# Characterisation and Molecular Analysis of an Unusual Chimeric Methicillin Resistant *Staphylococcus Aureus* Strain and its Bacteriophages

**DOI:** 10.3389/fgene.2021.723958

**Published:** 2021-11-18

**Authors:** Sindy Burgold-Voigt, Stefan Monecke, Alexandra Simbeck, Thomas Holzmann, Bärbel Kieninger, Elisabeth M. Liebler-Tenorio, Sascha D. Braun, Maximilian Collatz, Celia Diezel, Elke Müller, Wulf Schneider-Brachert, Ralf Ehricht

**Affiliations:** ^1^ Leibniz Institute of Photonic Technology (Leibniz-IPHT), Jena, Germany; ^2^ InfectoGnostics Research Campus, Jena, Germany; ^3^ Institute for Medical Microbiology and Virology, Dresden University Hospital, Dresden, Germany; ^4^ Department of Surgery, Asklepios Hospital Barmbeck, Hamburg, Germany; ^5^ Department of Infection Prevention and Infectious Diseases, University Hospital Regensburg, Regensburg, Germany; ^6^ Friedrich-Loeffler-Institute (Federal Research Institute for Animal Health), Institute of Molecular Pathogenesis, Jena, Germany; ^7^ Institute of Physical Chemistry, Friedrich Schiller University, Jena, Germany

**Keywords:** *Staphylococcus aureus*, trimethoprim, MRSA, bacteriophages, chromosomal replacement

## Abstract

In the context of microarray-based epidemiological typing of the clonal organism *Staphylococcus aureus*/MRSA*,* a strain was identified that did not belong to known clonal complexes. The molecular analysis by microarray-based typing yielded signals suggesting that it was a mosaic or hybrid strain of two lineages. To verify this result, the isolate was sequenced with both, short-read Illumina and long-read Nanopore technologies and analysed in detail. This supported the hypothesis that the genome of this strain, ST6610-MRSA-IVg comprised of segments originating from two different clonal complexes (CC). While the backbone of the strain’s genome, i.e., roughly 2 megabases, belongs to CC8, a continuous insert of 894 kb (approx. 30% of the genome) originated from CC140. Beside core genomic markers in the normal succession and orientation, this insert also included the *mecA* gene, coding for PbP2a and causing methicillin resistance, localised on an SCC*mec* IVg element. This particular SCC*mec* type was also previously observed in CC140 MRSA from African countries. A second conspicuous observation was the presence of the trimethoprim resistance gene *dfrG* within on a prophage that occupied an attachment site normally used by Panton-Valentine Leucocidin phages. This observation could indicate a role of large-scale chromosomal recombination in the evolution of *S. aureus* as well as a role of phages in the dissemination of antibiotic resistance genes.

## Introduction

*Staphylococcus aureus* is an abundant and extremely versatile bacterium that colonises, or infects 20–30% of any human population and also can be found in a wide range of wild or domestic animals. It can asymptomatically colonise its hosts, but it can also cause a wide variety of clinically relevant and sometimes fatal infections. These include skin and soft tissue infections of variable severity, osteomyelitis and septic arthritis, sepsis and endocarditis as well as pneumonia. It also can produce potent toxins and superantigens that might lead to toxigenic syndromes (scalded skin and toxic shock syndrome) or food intoxications. In recent decades, *S. aureus* acquired a wide range of resistance genes for different antimicrobial targets that complicate therapy: literally, an arms race took place between the development of new antibiotic compounds and the emergence of additional resistance modes. Most relevant are the *mecA* and *mecC* gene and their alleles which are localised on mobile SCC*mec* elements and serve as vehicles of a horizontal gene transfer between different lineages, and even different species, of staphylococci.

In general, the evolutionary success of *S. aureus* can be attributed to the modular organisation of its core, variable core and variable genome that allows a rapid evolution by means of shuffling and transfer of relevant genes.

The core genome with housekeeping genes engulfs genes that are essentially always present. Their order and orientation within the genome are as conserved as their sequences are. These genes evolve slowly by time-dependent accumulation of mutations. Seven of these genes are commonly used for a sequence-based typing scheme, the multilocus sequence typing (or MLST (Enright et al., 2000)) that allows assigning isolates to Sequence Types (ST) and combining related STs into larger phylogenetic clades, Clonal Complexes (CC). Some additional genes can be described as core-variable genes. This includes, for instance, genes for molecules that mediate adhesion to host structures. These genes are also conserved in both, position and sequence, but their sequences contain regions of high variability usually generated by repeating units that vary in number and sequence. The *spa* gene, commonly utilised for sequence-based so-called *spa*-typing, is one example for that class. Furthermore, there are genomic islands comprising fixed sets of genes that are present in some, but not all lineages of *S. aureus*.

*S. aureus* contains a number of mobile genetic elements, including SCC*mec* elements, plasmids, transposons, prophages and phage-like “pathogenicity islands” (PIs), that can transmit genetic information of evolutionary benefit, and of clinical relevance, i.e., genes encoding virulence factors or antibiotic resistance properties.

SCC*mec* elements typically include *mecA/C* genes (see above) and recombinase genes. They also can carry virulence factors (*tirS, psm-mec,* ACME) or other antibiotic resistance genes. There are also elements of this class that carry other payload such as fusidic acid (*fusC*) or heavy metal resistance genes instead, or in addition, to *mec* genes.

While plasmids and transposons carry very often resistance genes, most virulence factors are associated with prophages and *S. aureus* pathogenicity islands (SaPIs). Thus, these phages and SaPIs determine virulence and host specifity of *S. aureus* strains.

The bacteriophages of *S. aureus* are the tailed bacteriophages with double-stranded DNA genomes commonly belonging to the family *Siphoviridae* in the order *Caudovirales* (Dokland, 2019). In addition to transducing their own genes, prophages are also capable of transducing additional bacterial DNA. Many strains of *S. aureus* carry more than one prophage.

In addition, SaPIs are commonly found, averaging one per strain, although some strains might have three or more (Novick and Ram, 2017). SaPIs are ∼15-to 17-kb-chromosomal DNA fragments that are integrated into the bacterial chromosome and functionally related to bacteriophages (Novick and Subedi, 2007; Novick and Ram, 2016). They are transmitted horizontally, just like bacteriophages, but require the help of so-called helper-phages to do so. As molecular parasites, they have evolved mechanisms to take advantage of phage heads to spread their own genetic information, including virulence factors.

Beside these well-studied mechanisms of lateral or horizontal gene transfer, *S. aureus* might still have other options. A few strains show core genomic markers from distinct clonal complexes, or phylogenetic lineages. As these markers are considered non-motile, hybridisation or chimerism might be suspected, and affected regions might be as large as 20% of the entire genome. One of the first lineages for which this phenomenon was observed was CC239 that combines features of CC8 and CC30 (Robinson and Enright, 2004), resulting in a multi-resistant pandemic clone that was abundant for decades in hospitals around the globe. Another strain, ST2249, harbours CC239 DNA comprising fragments of both, CC8 and CC30 that is integrated into a CC45 genome (Nimmo et al., 2015). Further examples for chimeric strains are ST34/ST42 (where CC10 fragments are integrated into CC30 genomes (Robinson and Enright, 2004)), CC398 strains that harbour fragments of CC9 origin (Fetsch et al., 2017; Monecke et al., 2018), a CC80 strain incorporating two separate fragments of DNA from CC1 and an unknown donor strain (Gawlik et al., 2020), ST71 in which a region of unknown origin is integrated into a CC97 genome (Budd et al., 2015) as well as two MRSA strains (ST1048 and ST1774) from Hong Kong (Monecke et al., 2011). While there are several examples in which large scale genomic replacements or chimerism explain unusual features of strains that cannot be attributed to more conventional means of lateral gene transfer, the mechanism of transfer and integration of literally 100,000s nucleotides of core genome has yet to be elucidated.

In the present manuscript, we describe another strain of *S. aureus* for which chimerism was suspected based on its microarray profile. To test this hypothesis, the strain was sequenced using two different techniques. Sequencing also revealed the presence of a prophage carrying an antibiotic resistance gene (*dfrG*).

## Materials and Methods

### Patient History

The patient was a German national who lived in Africa. In 1999, he was treated for Non-Hodgkin lymphoma by stem cell transplantation. In addition, there was a history of several surgical interventions due to trauma and vascular conditions. In 2014, he was admitted to Regensburg University Hospital for a lower leg amputation due to late stage peripheral artery disease. The admission screening revealed MRSA colonisation with the isolate being stored and later subjected to molecular typing. After surgery, the patient became septic, and endocarditis was diagnosed. MRSA was detected in blood cultures. He was treated with vancomycin and rifampicin and survived that episode.

### Culture and Identification

The MRSA isolate was recovered by culture on a selective medium (Chrom ID MRSA, bioMérieux Marcy I’Etoile). Identification was achieved by linear matrix-assisted laser desorption ionisation–time of flight mass spectrometry (MALDI-TOF) (Bruker; Billerica, MA, United States). The isolate was stored frozen for subsequent genotyping.

### Antibiotic Susceptibility Testing

Antimicrobial susceptibility tests were performed using the Vitek 2 system (bioMérieux, Nürtingen, Germany) and the test card AST-P608, according to manufacturer’s instructions and guidelines of the European Committee on Antimicrobial Susceptibility Testing (EUCAST).

In addition, MICs for Trimethoprim/Sulfamethoxazole and Trimethoprim were determined using MIC Test Strips (bestbion dx, Köln, Germany/Liofilchem, Roseto degli Abruzzi, Italy), i.e., an agar diffusion technique that uses stripes impregnated with a gradient of an antibiotic compound in order to allow direct reading of the MIC. MIC Test Strips were used according to manufacturer’s instructions on Mueller Hinton agar (Roth, Karlsruhe, Germany).

### Amplification, Labeling and Microarray Hybridisation

The isolate was characterised using the DNA microarray-based assay from the Interarray *S. aureus* kit (fzmb GmbH, Bad Langensalza, Germany). Primer and probe sequences have been previously described in detail (Monecke et al., 2008; Monecke et al., 2011). In addition, a second array with probes mainly for the characterisation of the SCC*mec* element was used (Monecke et al., 2016). Protocols and procedures were in accordance with the manufacturer’s instructions. In brief, *S. aureus* was grown overnight on Columbia blood agar, harvested and enzymatically lysed prior to DNA preparation. The DNA was used in a multiplexed primer elongation incorporating biotin-16-dUTP. Amplicons were stringently hybridised to the microarray, washed and incubated with a horseradish-peroxidase-streptavidin conjugate. Hybridisations were detected by adding a precipitating dye. An image of the microarray was taken for further analysis.

### Phage Preparation and Isolation of Phage DNA

Phage induction was performed as described previously (Raya and H’bert, 2009). In summary, an overnight culture of the bacteria was inoculated in 2xTY medium and cultured at 37°C until the middle of the exponential growth phase. Mitomycin C (Roche, Basel, Switzerland) was added to a final concentration of 0.5 μg/ml, and cultivation was continued at 30°C until OD 600 nm decreased by 0.05.

The lysate was centrifuged at 4°C and 3,000 x g, the supernatant was neutralised with 0.1 N NaOH and filtered with a 0.20 µm cellulose acetate (CA) membrane filter (Sartorius, Göttingen, Germany). To sequence phage DNA, it was extracted from the phage filtrate. For this purpose, the phage filtrate was first treated with 10 μg/ml DNase I (Sigma Aldrich, Steinheim, Germany) and 10 μg/ml RNAse (Qiagen, Hilden, Germany) for 1 h at 37°C. This was followed by treatment with 20 mM EDTA, 50 μg/ml proteinase K, and 0.2% SDS and another incubation for 1 h at 65°C and 300rpm. Then, a phenol chloroform extraction was performed as described previously (Hagemann, 1990). For better separation of the phases, phase lock gel light tubes (Quantabio, Beverly, United States) were applied in each step. The isolated DNA was concentrated for 25 min at room temperature and 1,400 rpm in a SpeedVac vacuum concentrator (Eppendorf, Hamburg, Germany), and the final concentration was measured using the Qubit 4 fluorometer (ThermoFisher Scientific, Waltham, United States) according to the manufacturer’s instructions.

### Whole-Genome Sequencing by Illumina

DNA libraries were prepared with Nextera DNA Flex Library Prep and Nextera DNA CD Indexes (Illumina, San Diego, CA, United States). All libraries were sequenced on an Illumina Miniseq (Illumina, San Diego, CA, United States) using MiniSeq High Output Reagent Kit (300-cycles; Illumina, San Diego, CA, United States). The paired-end raw reads were imported into SeqSphere + software (Version 7.1.0, Ridom GmbH, Münster, Germany). Two independently prepared and sequenced samples were combined and quality checked. SeqSpere + uses the SKESA algorithm to assemble the raw reads and several output parameters were analysed to assure high quality sequences. The final assembly statics revealed a contig count of 130, N50 of 63.157, an assembled base count of 2.846.348 bases (2.8 Mbases), and an average genome coverage of 127. The cgMLST percentage of good targets was 98.4 and there was no evidence of contamination.

### Sequencing of Chromosomal and Phage DNA Applying Oxford Nanopore Technology

Oxford Nanopore Technology (ONT) sequencing of the isolated bacteria and phage DNA was performed using two different MinION flow cells (FLO-MIN106D for the isolated bacteria DNA, and FLO-FLG001 for phage DNA, both containing an R9.4.1 pore). Library preparations were done using the 1D genomic DNA by ligation kit (SQK-LSK109, ONT), and, additionally for the bacterial DNA, the native barcoding expansion kit (EXP-NBD104, ONT) following manufacturer’s instructions [protocol versions: GDE_9,063_v109_revX_Aug 14, 2019 (phage), NBE_9,065_v109_revR_Aug 14, 2019 (bacteria)]. Protocols were used by default with two minor exceptions: 1) the g-Tube shearing step was omitted and 2) incubation times of DNA repair and end preparation (dA tailing) were doubled. The flow cells were loaded with total amount of 120 ng of bacterial DNA, and 100 ng of phage DNA (measured by Qubit 4 Fluorometer; ThermoFisher Scientific, Waltham, United States). The sequencing ran for 48 h using the MinKNOW software version 20.06.5.

### Bioinformatic Analysis of Sequencing Data

For both data sets (bacteria and phage DNA) the guppy basecaller (v4.4.2, Oxford Nanopore Technologies, Oxford, United Kingdom) translated and trimmed the MinION raw data (fast5) into quality tagged sequence reads (4,000 reads per fastq-file). To get a smaller, better subset of reads, Filtlong (v0.2.0) was used with a median read quality of 15 and a minimum read length of 10,000 bp. Flye (v2.8.3) was used to assemble this subset of reads to high quality contigs (parameter: min-overlap 1,000 bp, --meta and --plasmids). Then, a racon-medaka pipeline (four times racon v1.4.3; once medaka v1.2.0) was applied for polishing. For medaka, the model r941_min_high_g360 was used. Beside the racon-medaka-polishing, the sequences were additionally polished using Illumina sequence data by pilon (v1.23). Pilon polishing was repeated until no more SNPs, indels and polyA/T were detected in the flye based assembly (about 4–5 times). The NCBI Prokaryotic Genome Annotation Pipeline (PGAP version 2021-01-11. build5132) was used for annotation all assembled contigs.

To search for excisionases in the bacterial and viral genomes, all coding excisionase sequences from European Nucleotide Archive (ENA) were downloaded (Download date April 20, 2021 https://www.ebi.ac.uk/ena/browser/text-search?query=excisionase). The 350 sequences were used for a BLAST analysis to find homologous genes potentially coding for excisionases.

### Phage Detection by Transmission Electron Microscopy

Drops of phage suspension were placed on a plate of dental wax. 300-mesh copper grids that had been filmed with formvar, coated with carbon and hydrophilised by glow discharge were floated on the drops for 30 min. Then grids were briefly rinsed in drops of distilled water and the excess liquid was drained. Finally, one grid of each preparation was contrasted on a drop of 1% phosphotungstic acid and one on a drop of 1% uranyl acetate for 1 min. After draining excess fluid and drying, grids were examined by transmission electron microscopy (Tecnai 12, FEI Deutschland GmbH, Dreieich, Germany) at 80 KV. Micrographs were taken using a TEMCAM FX416 (TVIPS, Gauting, Germany) and phage particles phages were measured using the EM-Measure software (TVIPS).

### Other Isolates Used for Comparison

Six isolates were used for comparison that have been collected in 2011 in Uganda from patients with skin and soft tissue infections (Monecke et al., 2013). These included a CC140-MRSA-IV isolate that was used for comparison of hybridisation profiles facilitating a presumptive identification of CC140 as donor of a part of the strain´s genome. In addition, five CC8-MSSA isolates were selected for further characterisation and *dfrG* PCR because they resembled the other part of the study strain´s genome also with regard to the carriage of mobile genetic elements.

### PCR for the Detection of *dfrG*

**FIGURE 1:**
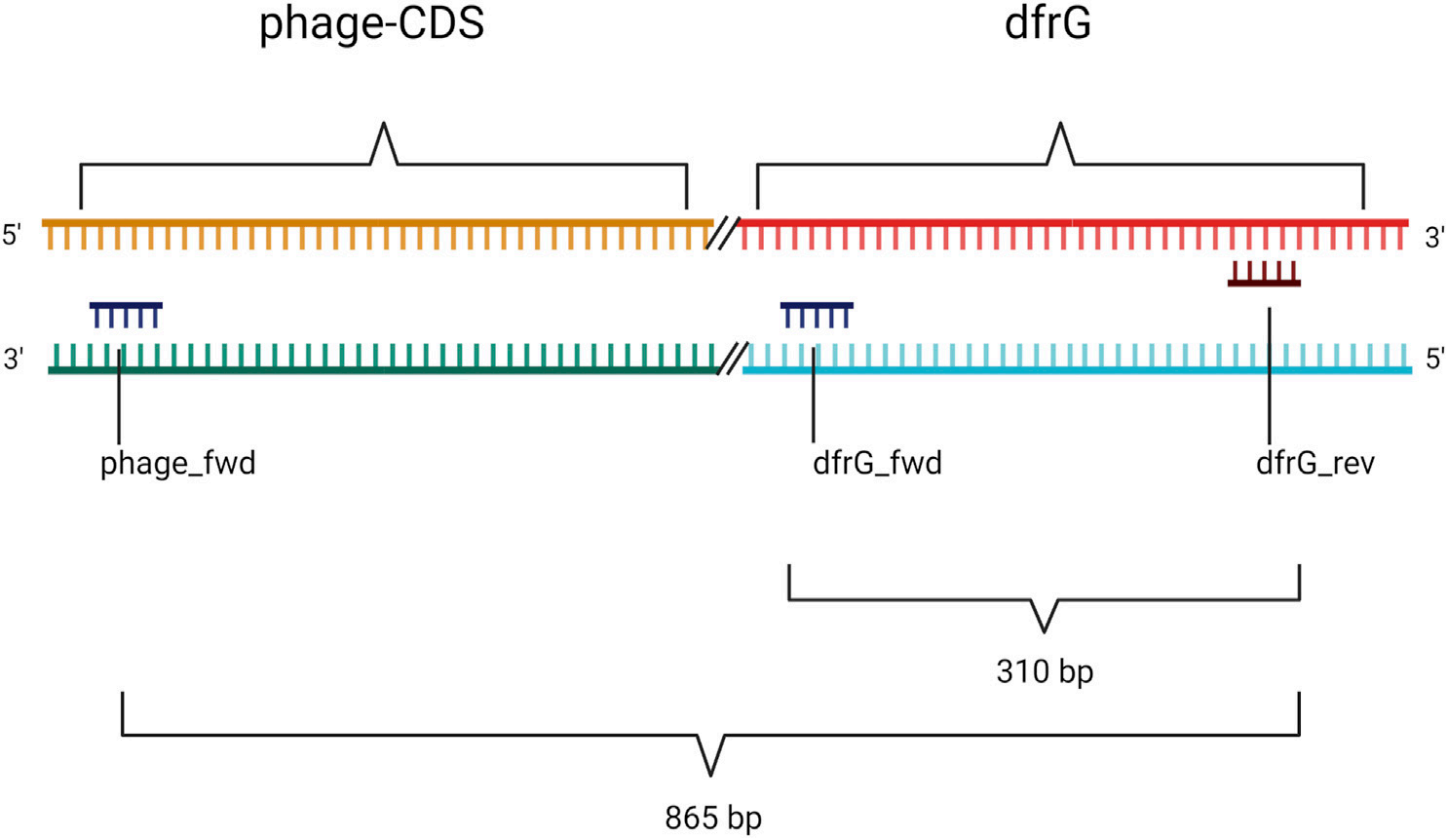
Non-scale arrangement of designated primers for PCR to detect local proximity between the phage-CDS and the *dfrG* gene. Created with BioRender.com.

Primers (Table 1) were designed for amplification of a sequence in the *dfrG* gene and for a sequence in the adjacent phage gene, hereafter referred to as “phage-CDS”. To detect the presence of the *dfrG* gene in the phage genome, the forward primer “phage-CDS” was combined with the reverse primer for *dfrG*, resulting, in the presence of *dfrG* in the phage, in an amplification product of 865 bp (MSSA476, BX571857 [1,022,819..1,022,983]) (Figure 1).

**TABLE 1:** Primer for detecting the local proximity of the *dfrG* and *phage-CDS* genes in the bacterial chromosome and the isolated phage-DNA.

Set	Primer name	Sequence (5′-3′)	Target gene	References sequence	Size of PCR product
**A**	dfrG_fwd	CAA​AGG​GAC​ATC​CGA​TAA​TA	*dfrG*	CP010526 [2,474,600..2,475,097]	310 bp
	dfrG_rev	AAT​ACC​TCA​TTC​CAT​TCC​TC	*dfrG*	CP010526 [2,474,600..2,475,097]	
**B**	phage_fwd	TTC​ACC​AAC​ATT​CGA​AGA​TA	Phage-CDS-*dfrG*	BX571857 [1,022,819..1,022,983]	865 bp
	dfrG_rev	AAT​ACC​TCA​TTC​CAT​TCC​TC	Phage-CDS-*dfrG*	CP010526 [2,474,600..2,475,097]	

Each PCR was performed with 0.1 U/µL Taq polymerase (Genaxxon BioScience GmbH, Ulm, Germany), 0.2 µM of the respective primers, 200 µM dNTPs (Genaxxon BioScience GmbH, Ulm, Germany), and 2.5 mM MgCl_2_ (Genaxxon BioScience GmbH, Ulm, Germany) in a final volume of 20 µL in 0.2 µL PCR tubes (STARLAB Int. GmbH, Hamburg, Germany). The PCR program was set in a Mastercycler® gradient device (Eppendorf, Hamburg, Germany) as follows: 95°C for 2 min, followed by 40 cycles of denaturation at 94°C for 30 s, annealing at 54°C for 45 s, elongation at 72°C for 1 min, followed by a final extension at 72°C for 10 min.

PCR products were checked by non-denaturing gel electrophoresis in a 2% agarose gel at 100 V/45 min 5 µL of a 50 bp-1.5 kbp Quantias Pro DNA ladder (Biozym, Hamburg, Germany) was used as a marker. The gel was analysed using a redTM Personal Gel Imaging System (Alpha Innotec, Kasendorf, Germany).

## Results

### MLST, Array-Based and *spa* Typing, Susceptibility Testing

The *S. aureus* isolate RGB-095930 was subjected to microarray-based analysis during an epidemiological study on MRSA in the Regensburg university hospital (Bavaria, Germany). It yielded an unusual and not yet known hybridisation pattern prompting further investigation. Array data as well as MLST and *spa* showed a combination of features previously seen in CC8 strains as well as in an African isolate (Monecke et al., 2013) assigned to CC140 (Supplementary File S1).

The allele of *arcC* (*arcC*-43) matched CC140 while five other MLST genes (*aroE-*3, *glpF*-1, gmk-1, *tpi*-4, *yqil*-3) were in concordance to CC8. The remaining MLST gene, *pta-*796 was a single nucleotide variant of an allele known to be associated with CC/ST8 (*pta*-4). Thus, the isolate was assigned to ST6610, a sequence type seen in two isolates from Tanzania (pubmlst.org/bigsdb?designation_operator1 = = &db = pubmlst_saureus_isolates&submit = 1&page = query&designation_value1 = 6,610&order = id&designation_field1 = s_1_ST&set_id = 0 (Kumburu et al., 2018)).

The *spa* gene had a Ridom profile of t293 (08-16-02-25-51-51-51-51). This does not match typical CC8 profiles such as t008 (11-19-12-21-17-34-24-34-22-25) but is related to the *spa* types of the other ST6610 isolates mentioned above (t296: 08-16-02-25-51-51-51) and to CC140 (t957, 08-16-02-25-51-51 (Berglund et al., 2009)).

**FIGURE 2:**
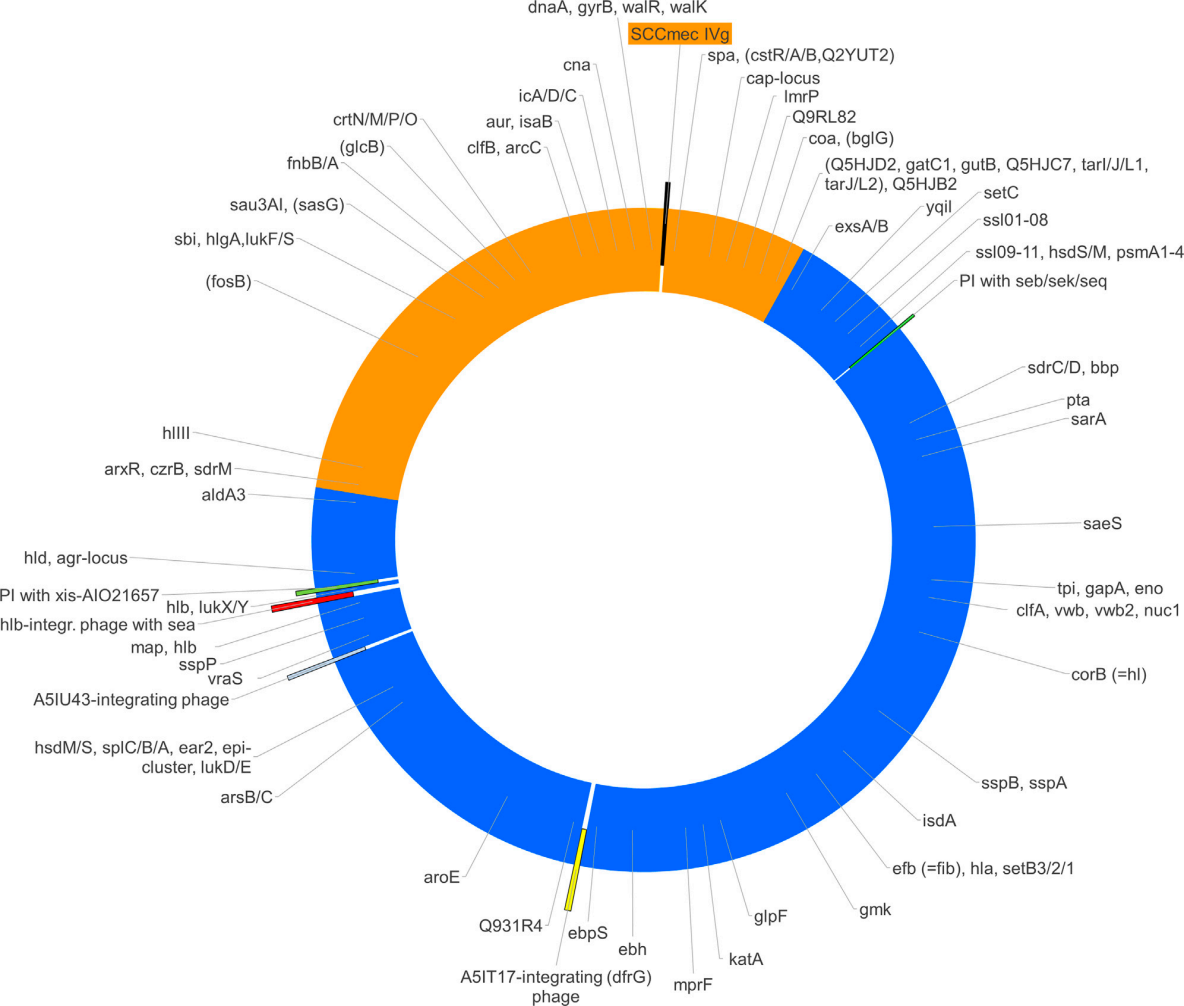
The figure shows a schematic representation of the chromosome of *S. aureus* RGB-095930 including the regions of different provenance (CC8, blue; CC140, orange), the localisations of mobile genetic elements (SCC*mec*, phages and pathogenicity islands, PI) as well as the approximate positions of relevant genes. Several of the genes targeted by the array have conserved positions within the *S. aureus* genome. If presence, absence or distinct alleles correlated with affiliation to one of the suspected parent lineages, the hybridisation profile can be used to determine the provenance of the part of the genome in which such a gene was localized.

The analysis of array data (Supplementary File S1) showed that the gene *cna,* which is absent in CC8, was present in the isolate as well as in CC140. Conversely, *sasG,* Q2G1R6/*cstB* and *fosB* were absent, as they are in CC140 rather than in CC8. For several genes (*aur, clfB, fnbA, isaB, hysA* genes, *sdrM,* and Q9RL82), alleles were recognised by array that were known to be associated with CC140. Other alleles (*agr* group I, *bbp, clfA, ebpS, efB, isdA, lukY, mprF, sdrC, sdrD, ssl* genes, *vwb*) as well as the presence of *ear2* and *esxB* were in accordance to CC8. When mapping the presence or absence as well as allelic variants over the known positions of the genes in question, a contiguous stretch of roughly one third of the strain´s genome appeared to originate from a CC140-like parent being integrated into a CC8 backbone genome (see Figure 2 and Supplementary File S1).

The isolate carried enterotoxin genes *sea, seb, sek* and *seq*, the staphylokinase gene *sak* and *scn* encoding a staphylococcal complement inhibitor. Panton-Valentine leukocidin genes (*lukF/S-PV*) were absent.

Susceptibility tests showed resistance towards beta-lactams, aminoglycosides (i.e., gentamicin and tobramycin), macrolides/lincosamides and trimethoprim which corresponded to the array-based detection of *mecA* as part of an SCC*mec* IVg element*, aacA-aphD, ermC* and *dfrG* respectively. In addition, resistance to fluoroquinolones and co-trimoxazole was noted (and genome sequencing revealed mutations in *gyrA, grlA* and *folP* that previously have been described to be associated with these properties; see Supplementary File S2 (Wang et al., 1998; Nurjadi et al., 2021)).

### Characterisation of the Strain’s Core Genome

**FIGURE 3:**
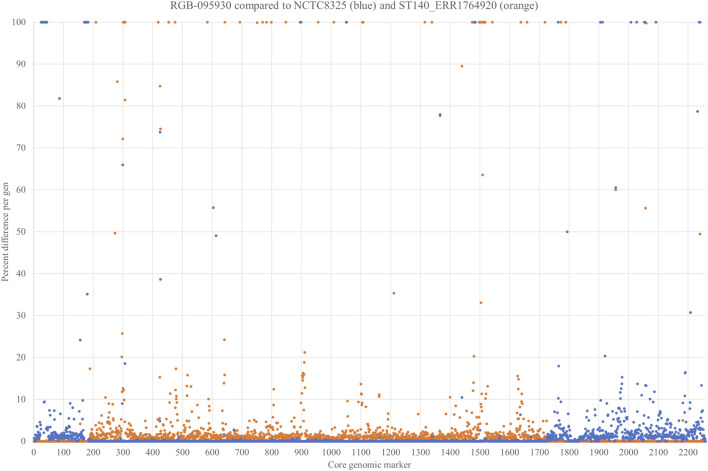
SNP analysis comparing 2,259 non-motile genes of RGB-095930, NCTC8325 GenBank CP000253.1 and Kenyaseq6547225, SAMEA4394796 (https://www.ncbi.nlm.nih.gov/sra/ERR1764920). For each single gene, the number of differences (Supplementary File S5) to the parent strains was counted and expressed as percentage. For genes that were present in one, but absent in the other strain, this percentage was set as 100%.

In order to prove our hypothesis of a chimeric strain and to characterise the boundaries of the integration more in detail, the strain was sequenced using Illumina and Oxford Nanopore technologies (GenBank accession number CP077098.1; Supplementary File S3). Non-motile genes with invariable positions within the *S. aureus* genome were selected (*n* = 2,259), and their respective sequences were compared to the CC8 reference sequence of Strain NCTC8325 GenBank CP000253.1 as well as to the genome sequence of Kenyaseq6547225, SAMEA4394796 (https://www.ncbi.nlm.nih.gov/sra/ERR1764920), an Illumina-sequenced MRSA from Kenya assigned to CC140 (Supplementary File S4). For each single gene, the number of nucleotides different than in the parent strains was counted and expressed as percentage of the length of the respective gene (Supplementary File S5). For genes that were present in one, but absent in the other strain, this percentage was set as 100%. Percentages were plotted over the positions in the genome (Figure 3). Despite some artefacts caused by technical issues (for instance the fact that only half of *cna* was found on one contig in ERR1764920), this procedure clearly showed that a part of the genome ranging from Q5HJB2, gene number (on the *x*-axis of Figure 3) 0,188 to *aldA3*, gene number 1725, was of CC8 origin while the remaining part of the genome originated from CC140. In addition to the SNP analysis, the presence or absence of genomic islands corroborates this notion. The CC140-derived part of the RGB-095930 genome, and the CC140 reference sequence concordantly lacked genomic islands and variable genes *cstR-GI + cstA-GI + cstB-GI*, Q5HJT2+Q6GD34 + A6QD75 + A6QD76 + A8YZ18, Q7A890 + Q2YUT2, *bglG* + Q5HJD2+Q5HJD1-*gatB1*+Q2G2C8-*gatC1+gutB +* Q5HJC7+Q5HJC6+*tarI1+tarJ1+tarL1*, *tarJ2+tarL2+tarS, fosB, glxK1, pnbA, sasG + sarT + sarU, glcB* and Q5HCK6+*padR* that all are present in CC8. Instead, the CC140-derived part of the RGB-095930 genome, and the CC140 harboured several genes absent from CC8, C2G7A0+Q6GKL3+C2G798 + D2N3E0+Q6GKL1 as well as *cna*. RGB-095930 matched CC8, but differed from CC140, in carrying *esxB, ssl10+hsdS-ssl + ssl11, dck, sdrD + bbp, yxxF, vwb2, yuzD, trfB, sspA, arsC, splF*, Q2FXC0-*ear*2, the lantibiotic epidermin biosynthesis cluster and a couple of other genes.

**FIGURE 4:**
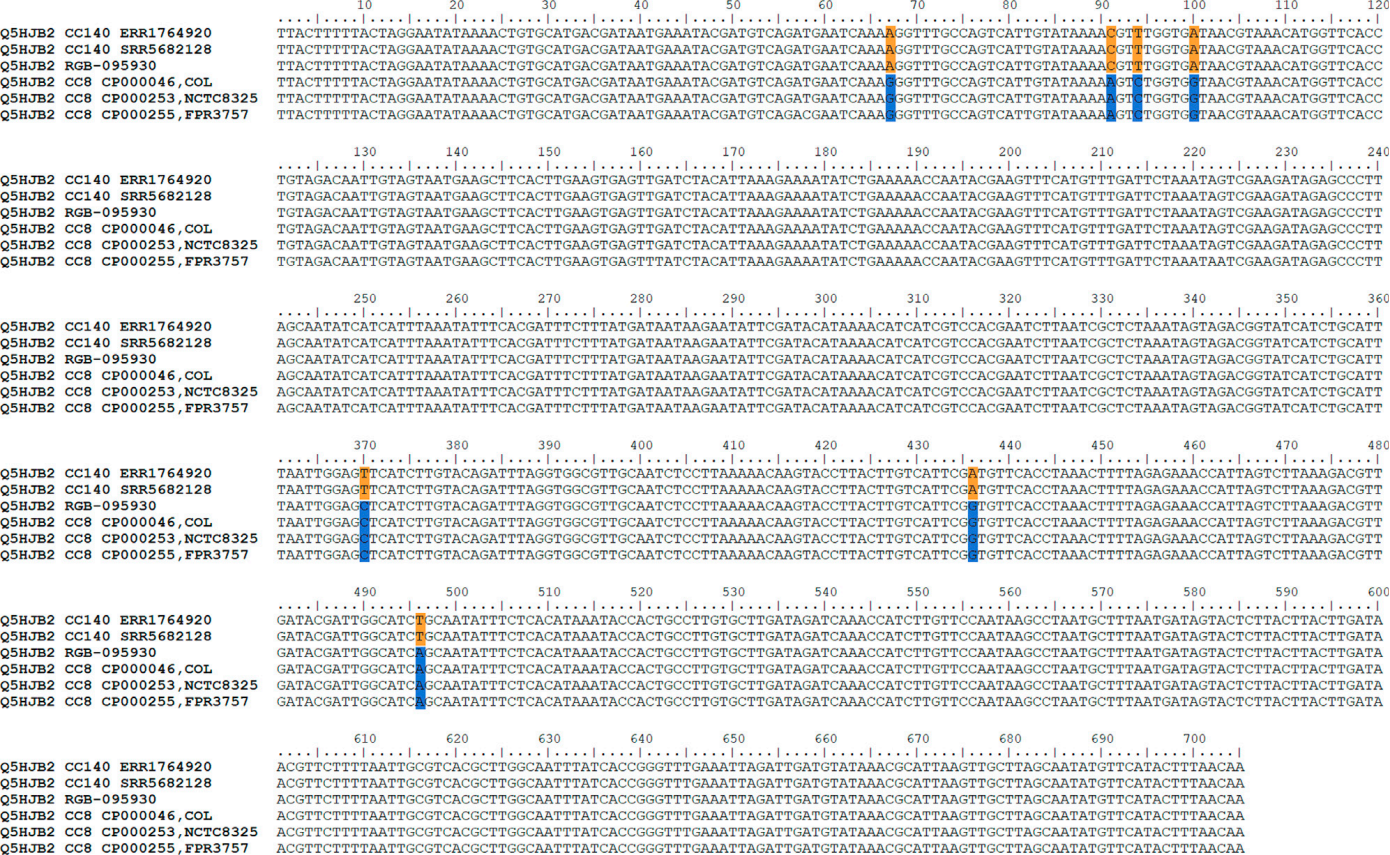
Alignment showing Q5HJB2 sequences of RGB-095930 as well as of CC8 and CC140 strains. Note that RGB-095930 shares SNPs with CC140 in the first part of the sequence but with CC8 in the second. CC8-specific SNPs are indicated in blue, CC140 ones in orange.

The gene encoding the putative GntR family transcriptional regulator Q5HJB2 (position 188 on the *x*-axis of Figure 3; or positions 279,625..280,329 in the genome), showed 0.4% difference to CC140 and 0.6% difference to CC8 while the gene before (*lrgB*) was identical to CC140 and the gene after (Q1YAD5-*ptsG3*) identical to CC8. Thus, it was assumed that the recombination breakpoint was localised within the gene, and indeed, its first 100 nucleotides lacked four SNPs characteristic for CC8 while its second half lacked three SNPs observed in CC140 ERR1764920 (Figure 4). Thus, it can be assumed that the recombination breakpoint was situated between position 100 and 370 of that gene.

The second recombination breakpoint appears to be situated between the end of *aldA3* and the beginning of *arxR* (position 1725 to 1726 on the *x*-axis of Figure 3; or between positions 2,290,040 and 2,290,278 of the genome). The *aldA3* gene of RGB-095930 differs in 0.98% of its sequence from the one in CC140 ERR1764920 and is completely identical to the one in NCTC8325. The intergenic region between *aldA3* and *arxR* differed in 0.84% compared to CC140 (2 mismatches at a length of 237 nt) and 0% to NCTC8325, while for *arxR*, the RGB-095930 allele is identical to CC140 but differs in 0.89% compared to NCTC8325.

The analysis of the cgMLST2208 provided on the MLST website (see pubmlst.org/bigsdb?db = pubmlst_saureus_seqdef&page = sequenceQuery and (Jolley et al., 2018)) and the comparison to NCTC8325, CP000253.1 and Kenyaseq6547225, ERR1764920, SAMEA4394796 also showed partial similarities to CC8 and CC140, respectively, with recombination breakpoints being situated around SAUR0261 (SAR_RS01305), or positions 279,625..280,329, and between SAUR2305 (SAR_RS11525) and SAUR2306 (SAR_RS11530), *i.e.,* between positions 2,290,040 and 2,290,278 (Supplementary File S6).

In conclusion, the observations indicate an integration of a segment that originates from CC140 into a backbone of CC8. As originally assumed, *sdrM,* Q9RL82, *cna, spa, arcC* as well as *orfX* and the SCC*mec* element are part of that inserted segment. Its size is about 894,000 nucleotides. Given a total genome size of 2,904,556 nucleotides, this equals roughly 30.8%.

### Mobile Genetic Elements in RGB-095930

Mobile genetic elements in RGB-095930 include a SCC*mec* element within the CC140-derived fragment of the genome as well as two Staphylococcal pathogenicity islands (SaPIs) and three full prophages that are situated in part of the genome that originates from CC8 (see Figure 2).

The SCC*mec* element (pos. 35,635..57,512; Supplementary File S7; Supplementary Table S1) belongs to SCC*mec* type IVg. The actual sequence in ERR1764920 is very similar to the one detected in RGB-095930. The only significant difference is a deletion of approximately 1,000 nt in RGB-095930 truncating both, delta *mecR*1 (from 978 down to 377 nt) and the IS1272 transposase, as well as removing a fragment of a type I restriction-modification system endonuclease present in ERR1764920.

**FIGURE 5:**
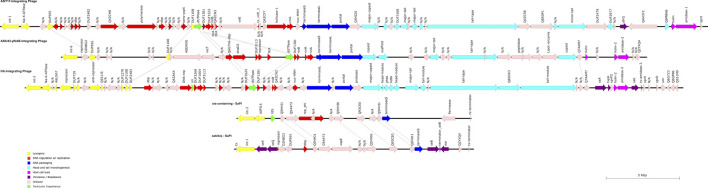
Schematic representation of the three Siphoviridae phages and the two SaPIs of RGB-095930. The directions, lengths, and positions of aligned genes (see also Supplementary Tables S2–S6) are shown by arrows. The colours represent the typical functional modules of *S. aureus* Siphoviridae: Lysogeny, DNA regulation und replication, DNA packaging, head and tail morphogenesis, and host cell lysis. Potential virulence factors are usually located downstream of the lysis module or, less commonly, inserted between lysogenesis and DNA metabolism (Deghorain and Van Melderen, 2012).

The first SaPI was named *Seb/k/q*-SaPI (pos. 862,265..878,144; Supplementary File S7; Supplementary Table S2; Figure 5). It is integrated in the CC8-derived part of the strain´s genome, between *metQ*1 (gene encoding methionine ABC transporter locus 1, substrate-binding protein) and *csbD-*L1 (gene encoding stress response protein, locus 1). This is the same integration site as in COL, CP000046, and the island carries the same genes in the same order and orientation as in COL. Thus, this pathogenicity island carries enterotoxin genes *seb, sek* and *seq*. Otherwise it is similar also to SaPI1 as described by Novick & Ram (Novick and Ram, 2016) in carrying homologs of phage integrase (*int*) and terminase small subunit (*terS)*. This SaPI does not contain an excisionase gene (*xis*). Just like the SaPI1, the *seb/k/q*-SaPI also contains the *ear* gene, coding for extracellular β-lactamase homolog.

A second SaPI (*xis*-SaPI, pos. 2,175,073..2,190,956; Supplementary File S7; Supplementary Table S3; Figure 5) is situated about 8,500 nt downstream of *hlb* and the *hlb*-integrating phage from which it is separated by several core genomic genes. It contains homologs of a phage integrase (*int*), a replication initiator (*rep-phi*), a terminase small subunit (*terS*) and, notably, an excisionase gene (*xis-*AIO21657).

**FIGURE 6:**
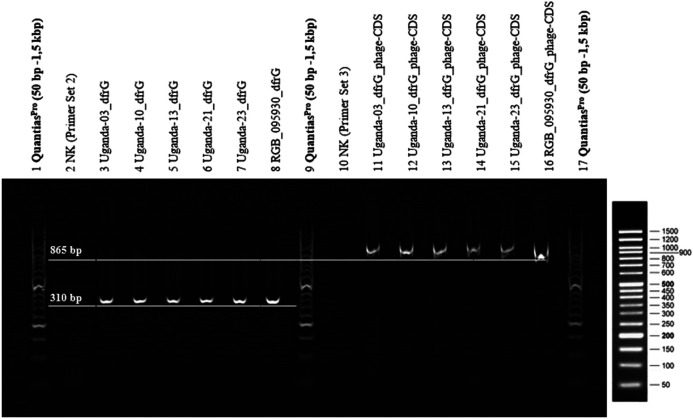
2% agarose gel (TBE-buffer/GelRed 1:10,000), 1x LoadingDye (Qiagen), Quantias Pro DNA Marker (50 bp-1.5 kbp), 100 V, 45 min. PCR products of primer set A, for the detection of *dfrG* (lane 2-8) and primer set B, for the detection of *dfrG* in the phage genome (lanes 10-16).

One prophage (pos. 1,538,924..1,586,638; Supplementary File S7; Supplementary Table S4; Figure 5) is integrated into A5IT17, encoding a putative protein. This prophage carried genes *dfrG* (encoding a dihydrofolate reductase associated with trimethoprim resistance) and Q4H3Y2, encoding an insertion element protein. The isolate was indeed resistant to trimethoprim as demonstrated by MIC Strip test (Supplementary File S2). The localisation of *dfrG* within the prophage was additionally demonstrated by PCR, ruling out a possible assembly artefact (see below, Figure 6).

A second prophage (pos. 2,015,849..2,055,180; Supplementary File S7; Supplementary Table S5; Figure 5) is integrated into A5IU43-*yfkAB*. This is a common integration site, also used in CC8 strains such as NCTC8325.

A third prophage (pos. 2,122,120..2,166,499; Supplementary File S7; Supplementary Table S6; Figure 5) interrupts the *hlb* gene which is also an integration site frequently used by *S. aureus* phages. It carries the enterotoxin A gene, the staphylokinase gene *sak* and a gene encoding the staphylococcal complement inhibitor, *scn.*

All three prophages lack *xis* genes. Nevertheless, at least one of them was inducible by addition of Mitomycin C. When Oxford Nanopore sequencing phage DNA and assembling sequence reads into contigs, one full, circular contig was observed with a coverage (or depth) of 1,014 that completely matched the entire sequence of the A5IU43-*yfkAB* integrating phage. Another contig contained a circular sequence corresponding to the complete *seb/sek/seq*-SaPI, albeit at a low coverage of 34. Additional non-circular contigs, with respective coverages between 3 and 12, contained various fragments of the other phages together with adjacent chromosomal sequences (Supplementary Files S8A,B).

### Presence of *dfrG* as Part of Prophage in Other Isolates

The presence of the *dfrG* gene as well as its linkage to the neighbouring phage gene were detected in RGB-095930 and five Ugandan CC8-MSSA strains. PCRs yielded products of the expected lengths (310 nt for the former, 865 nt for the latter), The results of the PCR are shown in Figure 6.

### Phage Morphology

**FIGURE 7:**
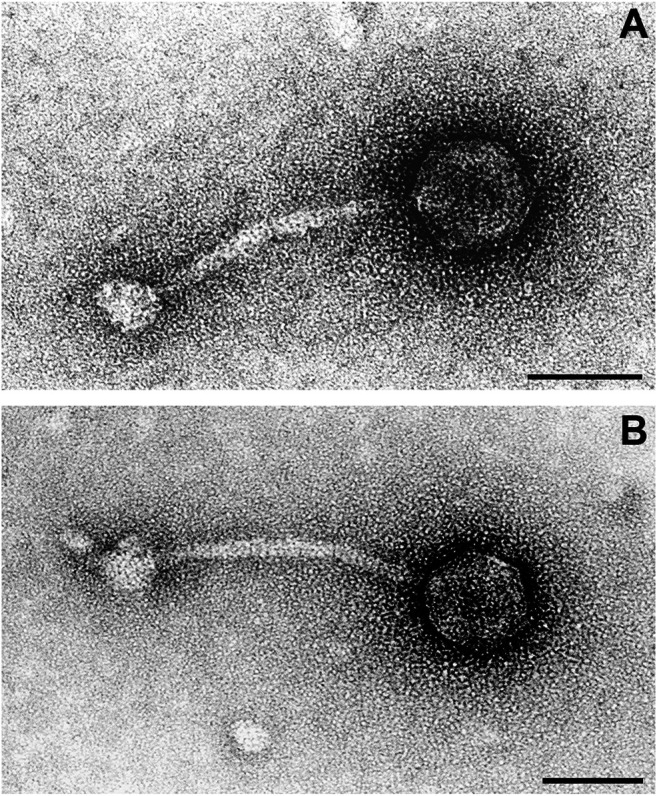
Transmission electron microscope image of two phage particles resulting from Mitomycin C treatment of bacterial strain RGB-095930. In particle **(A)** details of the icosahedral head, in particle **(B)** details of the tail and baseplate are more clearly depicted. Negative contrast preparation, contrasted with uranyl acetate. Bars = 50 nm.

There was a moderate number of phages in the preparation. One to two phage particles were detected per quadrant of the 300-mesh grid after Mitomycin C treatment. Phages were well contrasted with uranyl acetate and with phosphotungstic acid. All phages had a uniform morphology (Figure 7).

Phages were characterised by icosahedral, isometric heads with an average diameter of 50 ± 2 nm (range 44–58 nm). The thin (average diameter 8 ± 1 nm, range 8–10 nm), non-contractile tails were straight or curved, on average 130 ± 5 nm (range 122–140 nm) long and built of stacked discs. Tails terminated in a round to polygonal baseplate with an average diameter of 25 ± 2 nm (range 20–28 nm).

Based on these morphological features, phages were classified as members of the family *Siphoviridae* in the order *Caudovirales*.

## Discussion

The strain described herein presented with several interesting features shedding light on the evolution of *S. aureus*.

The most conspicuous one was the presence of a huge insert of CC140 provenance into a CC8 backbone. This insert spanned approximately 894,000 nucleotides, or roughly 31% of the strain´s genome. On one side, insertion bisected a gene (encoding Q5HJB2) while on the other side, the rift was localised in the intergenic region (between *aldA3* and *arxR*). The mechanism of such a recombination is currently unknown, and the insert is too large to be carried by phages.

Two isolates recently submitted to the MLST database are closely related to the strain described herein. They also represent ST6610 (MLST profile *arcC*-43, *aroE*-3, *glpF*-1, *gmk*-1, *pta-*796, *tpi*-4, *yqil*-3), their *spa* type was closely related (t296; 08-16-02-25-51-51-51) and their predicted hybridisation pattern (Supplementary File S1) was basically identical. Both originate from invasive infections affecting patients treated in a Tertiary Care Hospital in Moshi, Kilimanjaro region, Tanzania (Kumburu et al., 2018).

One of the two parental lineages, CC140, is a lineage that could indicate a connection to Africa where the patient used to live, or to North-western Europe. The authors observed that lineage in Uganda (Monecke et al., 2013), one genome sequence originated from Kenya (see above), another one from Khartoum, Sudan ((Ali et al., 2019); SAMN06894057). Both sequences include SCC*mec* IVg elements. In fact, this was one of the features drawing attention on CC140 as putative donor as SCC*mec* IVg is to our knowledge only present in few lineages, including CC59 and CC140. There are only few other reported observations of CC140. A corresponding *spa* type (t957, 08-16-02-25-51-51) was observed once in Uganda, twice in Sweden and once in Norway (https://spa.ridom.de/spa-t957.shtml) while the only entry in the MLST database came from Norway (https://pubmlst.org/bigsdb?db=pubmlst_saureus_isolates&page=info&id=1070). Other MLST-based identifications were reported from Sweden (Berglund et al., 2009) and Denmark (Bartels et al., 2015).

The backbone genome of RGB-095930 can be described as CC8 with a *sea*-positive *hlb* converting phage and a pathogenicity island carrying *seb, sek* and *seq*. CC8-MSSA matching that description have been frequently observed by the authors in Uganda (Monecke et al., 2013) while there are also other observations from Africa (Aiken et al., 2014; Shittu et al., 2015). MRSA with that toxin profile have been observed by the authors in patients repatriated from Mozambique and Zimbabwe (Monecke et al., 2016), and this strain is known to be brought from South Africa to West Australia ((Murphy et al., 2019); GenBank CP029166.1).

Thus, the most closely related strains described elsewhere (Kumburu et al., 2018) as well as both parental lineages, CC8 and CC140 appear to be linked to Africa. Unfortunately, no details on the exact travel history of the patient, on possible medications or exposures during his travels were recorded, mostly because his condition at hospital admission was clearly not related to foreign travel.

While it is currently impossible to further reconstruct the emergence of the CC8xCC140 chimera, it can be said that this recombination might have impacted the resulting chimeric strain. The transfer event removed *sasG,* encoding *S.taphylococcus aureus* surface protein G which stimulates adherence to desquamated nasal epithelial cells. It introduced the collagen-adhesion factor *cna* and the SCC*mec* IVg element. This is a rather rare variant of SCC*mec* IV previously observed in CC1 (Berglund et al., 2009; Monecke et al., 2020), CC30 (Berglund et al., 2009), CC45 (Berglund et al., 2009), CC59 (GenBank CP003604.1), CC361 (McManus et al., 2015) and CC140 (both genome sequences mentioned and (Berglund et al., 2009)).

The second conspicuous feature was the presence of the trimethoprim resistance gene *dfrG* within a prophage. The phage occupied the integration site usually used by PVL prophages, interrupting the gene encoding a conserved hypothetic protein, A5IT17. The *dfrG* gene was accompanied by a gene encoding an insertion element protein Q4H3Y2. It appears to be always linked to *dfrG*. Both genes together have been previously found as part of plasmids (AB205645.1), pathogenicity islands (CP010526.1) or integrated elsewhere into the genome (CP006838.1, FN433596.1) but to the best of our knowledge, not yet in prophages. In the studied strain, both genes together were found on a prophage integrating into A5IT17, encoding a putative protein. It frequently serves as integration site for prophages carrying the genes of the Panton-Valentine leucocidin (*lukF/S*-PV). However, in this strain, PVL genes were absent. The presence of *dfrG* was associated with resistance to trimethoprim and -together with mutations in *folP*- also to trimethoprim/sulfamethoxazole (Supplementary File S2). As the integration site of the prophage, the A5IT17 gene, is situated in the CC8-derived part of the strain´s genome, five *sea/seb/sek/seq*-positive CC8-MSSA from Uganda (see (Monecke et al., 2013) and above) were investigated and, indeed, yielded a positive PCR for *dfrG* and the neighbouring phage protein, suggesting 1) a presence of *dfrG* on a prophage in these isolates as well as 2) a close relationship of these African CC8 isolates to the CC8 part of the study strain´s genome. A possible phage-borne transmission of an antibiotic resistance gene could represent a public health problem, and thus it should be investigated which factors, or which medicaments, might cause a liberation of the *dfrG* phage and thus a transmission of *dfrG* to other *S. aureus* strains. It should also be investigated to which extend the recent proliferation of *dfrG* among *S. aureus* from Africa (Nurjadi et al., 2014; Coelho et al., 2017; Vandendriessche et al., 2017) might be related to the circulation of such phages.

Treatment of RGB-095930 with Mitomycin C resulted in the release of phage particles belonging to the family *Siphoviridae* based on their ultrastructural morphology. The rather uniform size and shape of heads, tails and base plates did not indicate the presence of different phages. We did Oxford Nanopore sequencing of the purified phage DNA in order to determine which of the strain´s prophages have been liberated. The sequence of A5IU43-*yfkAB*-integrating phage could be identified completely, as a ring closure, in a single contig and with much higher coverage than the other sequence fragments present. Based on the high coverage and the uniform microscopic appearance we assume that this phage was released most efficiently.

The other contigs obtained from the sequencing of the phage preparation included the complete *seb/sek/seq*-SaPI and diverse fragments encompassing incomplete fragments of the other prophages accompanied by chromosomal sequences (see Supplementary File S7). Neither the SaPI nor phages sequences excised after Mitomycin C treatment contained a known excisionase (*xis*) gene supposedly necessary for the excision of the prophage genome from the host genome.

The only known *xis* gene in the entire genome was detected within a SaPI (see Supplementary File S7; Supplementary Table S3, AIO21657). This particular *xis* gene is present in *S. aureus* H19-ST10, ACSS01000057 [34,506..34,823:RC] or in ATCC2592, CP009361.1 [2,051,151..2,051,468]. In the latter strain, it is also part of a SaPI which is situated at the same localisation as in RGB-095930, between *ktrB* and *groL*.

In all three prophages, a dUTPase gene was detected that was described by Cervera-Alamar (Cervera-Alamar et al., 2018) as one of the derepressor proteins for the stl-SaPI repressor. Furthermore, the *hlb*-integrating phage additionally possessed the *sri* gene, which can also act as a derepressor for the SaPI repressor. Because of the structural similarity of the SaPIs to the one described by Novick (Novick and Ram, 2016), it might be suspected that *sri* acted as a derepressor (Novick and Ram, 2017) leading to SaPI excision and to the onset of SaPI DNA replication. It cannot be ruled out, that other yet un-recognised derepressors (Cervera-Alamar et al., 2018) might be present in the prophage genomes hidden among the many uncharacterised “hypothetic proteins”. It can be assumed that the transcription of the *xis-*SaPI resulted in an excisionase that excised not only the associated SaPIs from the bacterial genome, but also the other SaPI and prophages, although with varying effectiveness. Indeed, it was previously demonstrated (Novick, 2003), that another SaPI, SaPI1 which does not encode any *xis* function, could be induced by the *xis* function of a phage, 80α. Thus, it was postulated that the excision was not sequence specific because the phage and SaPI1-att sites examined were unrelated (Novick, 2003). We hypothesise that in our case, conversely, a SaPI with a *xis* gene might help to induce phages without *xis* genes leading to an excision of prophages and SaPIs with different efficiencies. The greatest efficacy was clearly obtained for excision of the A5IU43-*yfkAB-*integrating phage. The reason for the different efficiency of excision of the mobile genetic elements in strain RGB-095930, as well as the detection of only partial sequences from the other two prophages, could be related to interference mechanisms between phages and SaPIs. Novick (Novick and Ram, 2017) described three mechanisms of SaPIs diverting the capsid production of phages to form smaller heads into which only the 15–17 kbp genome of SaPIs fits. However, postreplicative phage and SaPI DNA is packaged into these heads by the headful mechanism, with specificity being determined by terminase small subunits (TerS) of the phages and SaPIs that recognise phage and SaPI Pac sites, respectively, and differentially promote the packaging of phage and SaPIs DNA into procapsids of both sizes (Novick and Ram, 2017). Thus, smaller SaPI capsids, which can contain complete SaPI genomes or only about 1/3 of a phage genome, are also produced in addition to the normal-sized phage heads, which can contain both phage and SaPI genomes. From such a mixed population of capsids, phage and SaPI DNA was isolated and sequenced (Supplementary File S8).

Furthermore, sequences were detected that originate from chromosomal DNA from the regions adjacent to mobile genetic elements. This can be attributed to the ability of many bacteriophages (specialised transduction) to take up genetic material from their neighbourhood in addition to their own DNA and package it at a lower frequency upon entering the lytic cycle during excision from the host (Deghorain and Van Melderen, 2012; Dokland, 2019). The presence of chromosomal DNA fragments in the sequences of purified phage DNA could also be explained by lateral transduction. Here, chromosomal DNA is packaged that is located several thousand base pairs downstream of the prophage (Dokland, 2019) due to temperate phages that cannot detach from the chromosome after their induction but produce capsids into which only bacterial DNA is packaged (Gómez-Gómez et al., 2019). SaPIs are also capable of mediating generalised transduction (Novick and Ram, 2017). Like phages, homologs of SaPI Pac sites are scattered throughout the host genome and have highly variable efficiency, resulting in genes being transduced at different frequencies.

In summary, three practically relevant conclusions can be drawn from this study. First, evolution by large scale chromosomal replacements appear to be more common in the evolutionary history of *S. aureus* than previously appreciated as there is an increasing number of strains that can be described as chimeras combining genes and alleles from unrelated lineages (Robinson and Enright, 2004; Monecke et al., 2011; Budd et al., 2015; Nimmo et al., 2015; Fetsch et al., 2017; Monecke et al., 2018; Gawlik et al., 2020) although the mechanisms are not yet understood. Second, *xis* genes of SaPIs might enable the mobilisation of bacteriophages that do not carry XIS themselves. Third, phages can carry not only virulence factors but also resistance genes; and the issue of a possible role of bacteriophages in the spread of *dfrG* among African strains of *S. aureus* should further be investigated.

## Data Availability

The datasets presented in this study can be found in online repositories. The names of the repository/repositories and accession number(s) can be found below: www.ncbi.nlm.nih.gov/, PRJNA739061.
