# Supplementary material for: Characterisation and Molecular Analysis of an Unusual Chimeric Methicillin Resistant Staphylococcus Aureus Strain and its Bacteriophages
Source: Front Genet. 2021 Nov 18;12:723958. doi: 10.3389/fgene.2021.723958 (PMC8638950; doi:10.3389/fgene.2021.723958)
Supplement: Supplementary file 1 [file DataSheet7.PDF]

Table S1: The SCC*mec* IVg element in RGB-095930.

Gen ID	Description	Start in genome	End in genome	Length	Direction	Comments
**DR_SCC**	direct repeat of SCC	35,635	35,653	19		
***dcs*-L1**	downstream constant segment, locus 1	35,654	35,935	282		Identical to MW2, BA000033 [34169..34450]
**Q9XB68-*dcs***	putative protein	35,936	37,231	1296	FORWARD	Identical to COL, CP000046 [34372..35667] & MW2, BA000033 [34451..35746]
**Q7A213**	putative protein	37,646	37,885	240	FORWARD	Identical to COL, CP000046 [36082..36321] & MW2, BA000033 [36161..36400] & N315, BA000018 [36164..36403]
**IR_IS431**	inverted repeat of IS431	37,858	37,873	16	TRUNCATED	Identical to COL, CP000046 [36294..36309] & MW2, BA000033 [36373..36388]
**tnp-IS431**	transposase for IS431	37,917	38,591	675	REVERSE	
**Teg143**	trans-encoded RNA associated with tnpIS431	38,622	38,655	34	TRUNCATED	Identical to COL, CP000046 [37058..37091] & MW2, BA000033 [37137..37170]
**IR_IS431**	inverted repeat of IS431	38,632	38,647	16	TRUNCATED	Identical to COL, CP000046 [37068..37083] & MW2, BA000033 [37147..37162]
***mvaS*-SCC**	truncated 3-hydroxy-3-methylglutaryl CoA synthase	38,664	39,016	353	FORWARD-	Identical to COL, CP000046 [37100..37452] & MW2, BA000033 [37179..37531]
**Q5HJW6**	putative protein	39,114	39,344	231	FORWARD	Identical to COL, CP000046 [37550..37780] & MW2, BA000033 [37629..37859] & N315, BA000018 [42978..43208]
dru	SCC direct repeat units	39,254	39,691	438		
ugpQ	glycerophosphoryl diester phosphodiesterase associated with *mecA*	39,893	40,636	744	FORWARD	Identical to COL, CP000046 [38329..39072] & MW2, BA000033 [38288..39031] & N315, BA000018 [43717..44460]
ydeM	putative dehydratase	40,733	41,161	429	FORWARD	Identical to COL, CP000046 [39169..39597] & MW2, BA000033 [39128..39556]
**txbi_*mecA***	bidirectional rho-independent terminator of *mecA*	41,152	41,216	65		
mecA	penicillin binding protein 2a	41,207	43,213	2007	REVERSE	
***mecR1*-trunc**	methicillin resistance operon repressor 1, truncated	43,313	43,689	377	TRUNCATED-FORWARD	

Gen ID	Description	Start in genome	End in genome	Length	Direction	Comments
**tnp-IS1272**	transposase of insertion element IS1272	43,823	45,270	1448	TRUNCATED-REVERSE	
**Q9KX75-COL**	putative protein	45,321	45,827	507	REVERSE	Identical to COL, CP000046 [44617..45123]
**Q7A207**	putative protein	45,842	46,153	312	REVERSE	Identical to SA40, CP003604 [45892..46203:RC]
**Q7A206-delta**	putative protein, truncated copy	46,155	46,241	87	TRUNCATED-REVERSE	Identical to MW2, BA000033 [45410..45496]
**Q7A206**	putative protein	46,240	46,590	351	REVERSE	Identical to SA40, CP003604 [46290..46640:RC]
**UTR_*ccrB*-2-v2**	highly conserved 3'-untranslated region of *ccrB*	46,591	47,111	521		
ccrB-2	cassette chromosome recombinase B, type 2	47,112	48,740	1629	REVERSE	Identical to SA40, CP003604 [47162..48790:RC]
ccrA-2	cassette chromosome recombinase A, type 2	48,762	50,111	1350	REVERSE	Identical to SA40, CP003604 [48812..50161:RC]
cch-2	cassette chromosome helicase	50,345	52,138	1794	REVERSE	Identical to SA40, CP003604 [50395..52188:RC]
**DUF1413-2b**	putative protein associated with *cch*	52,138	52,431	294	REVERSE	Identical to SA40, CP003604 [52188..52481:RC]
**tnp-IS150**	transposase of IS150	52,666	52,980	315	FORWARD	Identical to SA40, CP003604 [52716..53030]
**tnp-A8YYY6**	transposase	52,992	53,807	816	FORWARD	Identical to SA40, CP003604 [53042..53857]
**Q6GD54**	putative protein	53,878	55,392	1515	REVERSE	Identical to SA40, CP003604 [53928..55442:RC]
**Q3YK51**	putative protein, marker for SCC*mec* IVg	55,554	57,302	1749	FORWARD	Identical to JCSC4796, AB266533 [15237..16985]
**DR_SCC**	direct repeat of SCC	57,494	57,512	19		

Table S2: The enterotoxin-carrying pathogenicity island in RGB-095930.

Gen ID	Description	Start in genome	End in genome	Length	Direction	Comments
**tx**	rho-independent terminator	862,265	862,314	50		
***int*-1_SaPI1**	bacteriophage integrase from SaPI1 and SaPI3	862,300	863,520	1221	REVERSE	Identical to COL, CP000046 [903491..904711:RC]
seK	enterotoxin K	863,609	864,337	729	REVERSE	Identical to NRS100, CP007539 [1478990..1479718]
seQ	enterotoxin Q	864,361	865,089	729	REVERSE	Identical to SA40, CP003604 [849105..849833:RC]
**repressor**	bacteriophage repressor	865,139	865,168	30	TRUNCATED-REVERSE	Identical to COL, CP000046 [906330..906359:RC]
**D2N8G3-nySaPI3**	lipoprotein	865,159	865,590	432	REVERSE	Identical to COL, CP000046 [906350..906781:RC]
**DUF955-SaPI3**	putative bacteriophagal protein	865,607	866,065	459	REVERSE	Identical to COL, CP000046 [906798..907256:RC]
**D0K398_phi**	putative DNA binding protein	866,858	867,130	273	FORWARD	Identical to COL, CP000046 [908049..908321]
**Q5HHJ1**	putative protein	867,493	867,813	321	FORWARD	
**O54473-nySa1**	putative protein	867,877	868,746	870	FORWARD	
vapE	bacteriophage virulence associated protein	868,796	870,220	1425	FORWARD	Identical to COL, CP000046 [909987..911411]
N/A	pathogenicity island protein from unclassified phage	870,521	870,883	363	FORWARD	
N/A	pathogenicity island protein from unclassified phage	870,885	871,169	285	FORWARD	
**Q5HHI6**	putative protein	871,166	871,807	642	FORWARD	Identical to COL, CP000046 [912357..912998]
**Q93CE0**	putative bacteriophagal protein	872,677	873,255	579	FORWARD	Identical to COL, CP000046 [913868..914446]
**Q5HHI1**	putative protein	874,072	874,413	342	FORWARD	Identical to COL, CP000046 [915263..915604]
**terminase-S**	terminase small subunit	874,410	874,979	570	FORWARD	Identical to SA40, CP003604 [859151..859720]

Gen ID	Description	Start in genome	End in genome	Length	Direction	Comments
seB	enterotoxin B	875,287	876,087	801	FORWARD	Identical to COL, CP000046 [916478..917278]
**txbi_*seB***	bidirectional rho-independent terminator of *seb*	876,115	876,169	55		
ear	enterotoxin-linked ampicillin resistance protein	876,165	876,722	558	REVERSE	
**Q2YXQ4_phi**	putative phage protein	877,415	877,615	201	FORWARD	Identical to COL, CP000046 [918606..918806]
**tx**	rho-independent terminator of orf Q2FII0	878,090	878,144	55		

Table S3: The ***xis***-carrying SaPI in RGB-095930.

Gen ID	Description	Start in genome	End in genome	Length	Direction	Comments
**txbi**	bidirectional rho-independent terminator of *ktrB*	2,175,073	2,175,118	46		
***per*-nySaPI2**	bacteriophage permease	2,176,140	2,177,075	936	FORWARD	Identical to H19-ST10, ACSS01000057 [22514..23449]
**terminase-S**	terminase small subunit	2,180,541	2,181,110	570	REVERSE	
**Q5HHI1**	putative protein	2,181,107	2,181,448	342	REVERSE	
N/A	hypothetic phage protein from unclassified DNA viruses	2,181,451	2,181,978	528	REVERSE	
**Q93CE0**	putative bacteriophagal protein	2,182,265	2,182,843	579	REVERSE	Identical to M0408, AIWO01000019 [9569..10147:RC]
**Q5HHI6**	putative protein	2,183,720	2,184,361	642	REVERSE	
pif	phage interference protein	2,184,358	2,184,738	381	REVERSE	Identical to H19-ST10, ACSS01000057 [30473..30853:RC]
***rep*_phi**	putative bacteriophage replication initiation protein	2,185,068	2,186,768	1701	REVERSE	
N/A	hypothetic phage protein from unclassified DNA viruses	2,185,690	2,185,986	297	FORWARD	
**O54473_phi**	phage DNA-replication protein	2,186,782	2,187,651	870	REVERSE	
**Q5HHJ1**	putative protein	2,187,715	2,188,035	321	REVERSE	
***xis*-AIO21657**	putative excisionase	2,188,383	2,188,700	318	REVERSE	Identical to H19-ST10, ACSS01000057 [34506..34823:RC]
**A0FIL5**	regulatory protein with helix turn helix motif	2,189,096	2,189,770	675	FORWARD	Identical to N315, BA000018 [2070412..2071086]
***int*-3_nySa4**	bacteriophage integrase	2,189,784	2,190,956	1173	FORWARD	Identical to Strain_6850, CP006706 [1987914..1989086]

Table S4: The A5IT17-integrating prophage in RGB-095930.

Gen ID	Description	Start in genome	End in genome	Length	Direction	Comments
sprX	noncoding small regulatory RNA	1,538,924	1,539,072	149		
**amidase-1**	bacteriophage amidase from *Siphoviridae*	1,539,282	1,540,736	1455	REVERSE	Identical to A9765, ACSN01000006 [85805..87259:RC]
***holA*-1**	holin from *Siphoviridae*	1,540,747	1,541,049	303	REVERSE	Identical to JKD6159, CP002114 [1517285..1517587:RC]
**Q9MBN6**	conserved hypothetic protein, SACOL0387	1,541,185	1,541,484	300	REVERSE	
**Q4H3Y2**	insertion element protein associated with *dfrG*	1,542,010	1,543,962	1953	FORWARD	
dfrG	dihydrofolate reductase	1,544,034	1,544,531	498	REVERSE	Identical to DAR4145, CP010526 [2474600..2475097]
N/A	hypothetic phage protein, SAS0951, contains the primer binding site for a PCR proving the presence of *dfrG* within the prophage (*Phage-CDS*, phage fwd)	1,544,815	1,544,979	165	REVERSE	Identical to MSSA476, BX571857 [1022819..1022983]
***mitp2*-DUF2977**	DUF2977 domain-containing protein, tail module of *Siphoviridae*, SACOL0385	1,544,972	1,545,361	390	REVERSE	
**DUF2479/ SLTorf488**	putative bacteriophagal protein	1,545,361	1,546,827	1467	REVERSE	Identical to CP007659 [1540263..1541729:RC]
***mitp*1**	minor structural protein, UG86_01670, tail module of *Siphoviridae*	1,546,827	1,548,737	1911	REVERSE	Identical to 93bS9, CP010952 [356210..358120]
N/A	hypothetic protein, SACOL0382	1,548,753	1,549,043	291	REVERSE	Identical to COL, CP000046 [391039..391329]
**Q8SDP1**	peptidase, UG86_01660	1,549,043	1,550,626	1584	REVERSE	
***holA*-Q4ZCS6**	conserved hypothetic protein, SACOL0380	1,550,635	1,551,459	825	REVERSE	Identical to COL, CP000046 [388623..389447]
**tail-fiber_phiSLT**	tail protein, UG86_01650	1,551,459	1,557,659	6201	REVERSE	
N/A	phage protein, UG86_01645	1,557,673	1,557,831	159	REVERSE	Identical to 93bS9, CP010952 [347116..347274]
N/A	hypothetic protein, SACOL0377	1,557,873	1,558,223	351	REVERSE	Identical to COL, CP000046 [381859..382209]
matp	prophage L54a, major tail protein, putative, SACOL0376	1,558,281	1,558,736	456	REVERSE	Identical to COL, CP000046 [381346..381801]

Gen ID	Description	Start in genome	End in genome	Length	Direction	Comments
N/A	hypothetic phage protein from *Sipho-/Triavirus*	1,558,770	1,558,970	201	REVERSE	Identical to Phage_42e, ORF083 [AY954955.1 [7227..7427]
matp1	tail protein from *Sipho-/Triavirus*	1,558,828	1,559,469	642	REVERSE	
**DUF3168**	hypothetic protein, UG86_01625, DUF3168 domain-containing protein, head module of *Siphoviridae*	1,559,504	1,559,899	396	REVERSE	
N/A	hypothetic phage protein from *Sipho-/Triavirus*	1,559,900	1,560,301	402	REVERSE	Identical to Phage_3A, ORF028 [AY954956.1 [5966..6367]
N/A	phage protein, UG86_01615	1,560,298	1,560,630	333	REVERSE	
N/A	hypothetic phage protein from *Sipho-/Triavirus*	1,560,931	1,561,041	111	REVERSE	
macp	major phage capsid protein	1,560,989	1,562,152	1164	REVERSE	
**Q5HIZ6**	bacteriophage prohead/Clp-protease from *Sipho-/Triavirus*	1,562,164	1,562,937	774	REVERSE	
**portal**	portal protein, UG86_01595	1,562,921	1,564,159	1239	REVERSE	
**terminase-L**	bacteriophage terminase, large subunit	1,564,164	1,565,855	1692	REVERSE	Identical to A9765, ACSN01000006 [107399..109090:RC]
**terminase-S**	prophage L54a, terminase, small subunit, putative, SACOL0366	1,565,845	1,566,150	306	REVERSE	Identical to COL, CP000046 [373932..374237]
***nuc*-HNH**	HNH-endonuclease	1,566,279	1,566,593	315	REVERSE	Identical to C427_ST42, ACSQ01000023 [22064..22378:RC]
***rinA*-Q4ZCF5**	phage transcriptional regulator/activator	1,566,750	1,567,187	438	REVERSE	Identical to C427_ST42, ACSQ01000023 [22535..22972:RC]
***dhlC*-1**	helicase 1 1359nt from *Siphoviridae*	1,567,200	1,568,558	1359	REVERSE	Identical to A9765, ACSN01000006 [110435..111793:RC]
***nuc*-Q4ZCF7**	hypothetic phage protein, SAS0927	1,568,548	1,568,838	291	REVERSE	Identical to MSSA476, BX571857 [998944..999234]
N/A	hypothetic protein, UG86_01560	1,568,981	1,569,070	90	FORWARD	Identical to 93bS9, CP010952 [335879..335968]
N/A	hypothetic phage protein from *Sipho-/Triavirus*	1,569,033	1,569,155	123	FORWARD	
**tx_*virE***	rho-independent terminator of *virE*	1,569,114	1,569,154	41		
virE	bacteriophage virulence-associated protein E	1,569,179	1,571626	2448	REVERSE	

Gen ID	Description	Start in genome	End in genome	Length	Direction	Comments
N/A	hypothetic phage protein, SAS0925	1,571,681	1,571,887	207	FORWARD	Identical to MSSA476, BX571857 [995895..996101]
**Q4ZCN3**	hypothetic protein, UG86_01550	1,571,914	1,572,114	201	REVERSE	Identical to 93bS9, CP010952 [332835..333035]
N/A	hypothetic phage protein from *Sipho-/Triavirus*	1,572,056	1,572,157	102	FORWARD	
N/A	hypothetic phage protein, SAS0923a	1,572,117	1,572,188	72	REVERSE	Identical to MSSA476, BX571857 [995594..995665]
rinB	Transcriptional-activator	1,572,182	1,572,334	153	REVERSE	Identical to JKD6159, CP002114 [1545432..1545584:RC]
**DUF1523**	replication-assoc-hypothetic-protein_SPV-80A_gp36	1,572,331	1,572,717	387	REVERSE	
**DUF1381**	DUF1381 domain-containing protein from *Siphoviridae*	1,572,714	1,572,920	207	REVERSE	
***dut*_phi**	dUTPase	1,572,957	1,573,487	531	REVERSE	Identical to A9765, ACSN01000006 [116192..116722:RC]
**DUF1108**	phage protein, UG86_01465	1,573,480	1,573,728	249	REVERSE	
N/A	hypothetic phage protein from *Sipho-/Phietavirus*	1,573,721	1,574,010	290	REVERSE	
sens-yopX	hypothetic sensor protein, replication module of *Siphoviridae*	1,574,026	1,574,373	348	REVERSE	Identical to COL, CP000046 [370100..370447]
N/A	hypothetic phage protein, SAS0915	1,574,370	1,574,774	405	REVERSE	Identical to MSSA476, BX571857 [992632..993036]
N/A	hypothetic phage protein, SAS0914	1,574,777	1,574,983	207	REVERSE	Identical to MSSA476, BX571857 [992423..992629]
N/A	conserved hypothetic protein, SACOL0351	1,574,997	1,575,239	243	REVERSE	
N/A/	phage protein, UG86_01500	1,575,245	1,575,499	255	REVERSE	
dbp	phage DNA polymerase or DNA binding protein, UG86_01495	1,575,499	1,575,900	402	REVERSE	Identical to 93bS9, CP010952 [329289..329690]
N/A	phiSLT ORF65-like protein, UG86_01490	1,575,900	1,576,085	186	REVERSE	Identical to 93bS9, CP010952 [329104..329289]
***pol*_phi12/ phi2638A**	DNA polymerase, UG86_01485	1,576,098	1,578,059	1962	REVERSE	
N/A	hypothetic protein, UG86_01480	1,578,118	1,578,675	558	REVERSE	Identical to 93bS9, CP010952 [326514..327071]

Gen ID	Description	Start in genome	End in genome	Length	Direction	Comments
**Q4ZCH8**	bacteriophagal protein, putative nuclease UG86_01475/SAS0907 from *Siphoviridae*	1,578,701	1,579,867	1167	REVERSE	
N/A	phage protein, UG86_01470	1,579,864	1,580,226	363	REVERSE	
**DUF2482**	DUF2482 family protein, ERS140266_RS12095	1,580,505	1,580,807	303	REVERSE	
N/A	phage protein, UG86_01455	1,581,210	1,581,371	162	REVERSE	Identical to 93bS9, CP010952 [324125..324286]
dbp	putative DNA-binding protein, SAS0902	1,581,383	1,581,646	264	REVERSE	Identical to MSSA476, BX571857 [986076..986339]
N/A	phage protein, UG86_01445	1,581,671	1,581,886	216	REVERSE	Identical to 93bS9, CP010952 [323610..323825]
N/A	hypothetic phage protein, SAS0900	1,581,933	1,582,172	240	FORWARD	
N/A	hypothetic phage protein, SAS0899	1,582,169	1,582,345	177	REVERSE	Identical to MSSA476, BX571857 [985377..985553]
N/A	putative phage regulatory protein, SAS0898	1,582,429	1,582,629	201	REVERSE	Identical to MSSA476, BX571857 [985093..985293]
N/A	putative phage regulatory protein, SAS0897	1,582,781	1,583,110	330	FORWARD	Identical to MSSA476, BX571857 [984612..984941]
**DUF955**	putative bacteriophagal protein	1,583,123	1,583,584	462	FORWARD	Identical to JKD6159, CP002114 [1555765..1556226]
N/A	putative lipoprotein, SAS0895	1,583,602	1,584,036	435	FORWARD	
**Na-K-ATPase**	Na-K-ATPase from *Sipho-/Triavirus*	1,584,693	1,585,307	615	REVERSE	
***int*-1**	integrase from *Siphoviridae*	1,585,433	1,586,638	1206	FORWARD	Identical to JKD6159, CP002114 [1558075..1559280]

Table S5: The A5IU43-*yfkAB*-integrating prophage in RGB-095930.

Gen ID	Description	Start in genome	End in genome	Length	Direction	Comments
**Q2YXQ4_phi**	putative phage protein	2,015,849	2,016,049	201	REVERSE	
N/A	hypothetic phage protein from *Sipho-/Phietavirus*	2,016,036	2,016,146	111	REVERSE	
N/A	hypothetic protein	2,016,218	2,016,370	153	REVERSE	
**amidase-2**	bacteriophage amidase from *Siphoviridae*	2,016,581	2,017,336	756	REVERSE	
hol-2	holin from *Sipho-/Biseptimavirus*	2,017,348	2,017,602	255	REVERSE	Identical to Strain_21202, AGRO01000005 [97..351]
N/A	hypothetic phage protein from *Sipho-/Phietavirus*	2,018,635	2,018,838	204	TRNC-FRWD	
hol	holin from *Sipho-/Phietavirus*	2,019,386	2,019,568	183	REVERSE	
**Q7A4M7_phi187**	putative bacteriophagal protein	2,019,762	2,020,136	375	REVERSE	
cap	capsid protein from *Sipho-/Phietavirus*	2,020,158	2,021,429	1272	REVERSE	
**Lipolytic-enzyme**	lipolytic enzyme from *Sipho-/Phietavirus*	2,021,446	2,022,489	1044	REVERSE	
N/A	hypothetic phage protein from *Sipho-/Phietavirus*	2,022,501	2,023,193	693	REVERSE	
**endopeptidase**	endopeptidase from *Sipho-/Phietavirus*	2,023,197	2,024,594	1398	REVERSE	Identical to Phage_187, ORF004 [AY954950.1 [13391..14788]
N/A	hypothetic phage protein from *Sipho-/Phietavirus*	2,023,744	2,023,893	150	TRNC-FRWD	Identical to Phage_187, ORF153 [AY954950.1 [14092..14241:RC]
N/A	hypothetic phage protein from *Sipho-/Phietavirus*	2,024,607	2,025,539	933	REVERSE	
***tmpM*-6-phi187**	phage tail tape measure protein	2,025,552	2,028,659	3108	REVERSE	
N/A	hypothetic phage protein from *Sipho-/Phietavirus*	2,026,294	2,026,455	162	TRNC-FRWD	Identical to Phage_187, ORF102 [AY954950.1 [11530..11691:RC]
N/A	hypothetic phage protein from *Sipho-/Phietavirus*	2,028,675	2,029,025	351	REVERSE	
N/A	hypothetic phage protein from *Sipho-/Phietavirus*	2,029,055	2,029,435	381	REVERSE	

Gen ID	Description	Start in genome	End in genome	Length	Direction	Comments
tmp	major tail protein from *Sipho-/Phietavirus*	2,029,494	2,030,147	654	REVERSE	
N/A	hypothetic phage protein from *Sipho-/Phietavirus*	2,030,165	2,030,560	396	REVERSE	
N/A	hypothetic phage protein from *Sipho-/Phietavirus*	2,031,221	2,031,607	387	REVERSE	
**Scaffold**	Scaffold from *Sipho-/Phietavirus*	2,033,033	2,033,656	624	REVERSE	
capS	capsid-S from *Sipho-/Phietavirus*	2,033,758	2,034,726	969	REVERSE	
**portal**	portal protein from *Siphoviridae*	2,034,710	2,036,155	1446	REVERSE	
**terminase-L**	terminase large subunit from *Sipho-/Phietavirus*	2,036,167	2,037,507	1341	REVERSE	
**terminase-S**	terminase small subunit from *Siphoviridae*	2,037,428	2,037,880	453	REVERSE	
rinA	RinA family transcriptional activator from *Siphoviridae*, locus tag SPV-80A_gp39	2,038,057	2,038,479	423	REVERSE	
rinM	ORF between *rinA* and *rinB*, replication module of *Siphoviridae*	2,038,503	2,038,649	147	REVERSE	
rinB	transcriptional activator	2,039,013	2,039,186	174	REVERSE	
**DUF1381**	DUF1381 domain-containing protein from *Siphoviridae*	2,039,183	2,039,389	207	REVERSE	
***dut*-phi**	dUTPase	2,039,426	2,039,959	534	REVERSE	
N/A	hypothetic phage protein from *Sipho-/Triavirus*	2,040,952	2,041,200	249	REVERSE	
rep	bacteriophage repressor/transcriptional regulator from *Sipho-/Triavirus*	2,041,561	2,041,828	268	REVERSE	
DUF3113	hypothetic phage protein, DUF3113 family protein, replication module of *Siphoviridae*	2,041,829	2,042,014	186	REVERSE	
dnaD2	bacteriophage DNA replication protein	2,042,655	2,043,539	885	REVERSE	
***ssbP*-Q6G7V6**	ssDNA-binding protein	2,043,569	2,044,039	471	REVERSE	
N/A	hypothetic phage protein, SAS1905	2,044,040	2,044,525	486	REVERSE	

Gen ID	Description	Start in genome	End in genome	Length	Direction	Comments
***recT*_phiNM3**	phage recombination protein from *Sipho-/Biseptimavirus*	2,044,738	2,045,658	921	REVERSE	Identical to 11819-97, CP003194 [2103073..2103993:RC]
**A0EWX0**	hypothetic phage protein, SAS1907	2,045,660	2,047,609	1950	REVERSE	
N/A	hypothetic phage protein, SAS1908	2,047,612	2,047,890	279	REVERSE	
**DUF1108**	phage protein, UG86_10455	2,047,884	2,048,144	261	FORWARD	
N/A	hypothetic phage protein from *Sipho-/Phietavirus*	2,048,960	2,049,070	111	FORWARD	
N/A	hypothetic Transcriptional regulator from *Sipho-/Phietavirus*	2,049,083	2,049,304	222	REVERSE	
N/A	hypothetic phage protein from *Sipho-/Phietavirus*	2,051,792	2,052,007	216	FORWARD	
N/A	hypothetic phage protein from *Sipho-/Phietavirus*	2,051,985	2,052,167	183	REVERSE	
N/A	conserved hypothetic phage protein, SAAV_2052	2,052,596	2,052,919	324	FORWARD	
**DUF955**	putative bacteriophagal protein	2,052,932	2,053,393	462	FORWARD	
**D2N8G3**	lipoprotein	2,053,409	2,053,840	432	FORWARD	
**repressor**	bacteriophage repressor	2,053,831	2,053,860	30	TRNC-FRWD	Identical to COL, CP000046 [906330..906359:RC]
***int*-6_Siph**	integrase	2,054,134	2,055,180	1047	FORWARD	

Table S6: The *hlb* integrating prophage in RGB-095930.

Gen ID	Description	Start in genome	End in genome	Length	Direction	Comments
**Q931M9**	putative membrane protein, assoc. with *hlb*-integr. phages/*Siphoviridae*	2,122,120	2,122,299	180	FORWARD	Identical to MW2, BA000033 [2046605..2046784]
**Q6GFB6**	putative membrane protein, assoc. with *hlb*-integr. phages/*Siphoviridae*	2,122,323	2,122,631	309	FORWARD	Identical to MW2, BA000033 [2046808..2047116]
**Q6G7Z3**	putative membrane protein, assoc. with *hlb*-integr. phages/*Siphoviridae*	2,122,679	2,122,870	192	FORWARD	Identical to MW2, BA000033 [2047164..2047355]
scn	staphylococcal complement inhibitor	2,122,923	2,123,273	351	REVERSE	Identical to 11819-97, CP003194 [2072409..2072759:RC]
sprD	small pathogenicity island RNA D	2,123,431	2,123,572	142		
N/A	hypothetic phage protein from *Sipho-/Triavirus*	2,123,986	2,124,117	132	REVERSE	
sprX	noncoding small regulatory RNA	2,124,321	2,124,472	152		
**tx_amidase3**	rho-independent terminator of amidase 3	2,124,557	2,124,612	56		
sak	staphylokinase	2,124,769	2,125,260	492	REVERSE	Identical to 11819-97, CP003194 [2074256..2074747:RC]
**amidase-2**	bacteriophage amidase from *Siphoviridae*	2,125,451	2,126,206	756	REVERSE	Identical to AB033232 [5502..6257]
hol-2	holin from *Sipho-/Biseptimavirus*	2,126,218	2,126,472	255	REVERSE	Identical to 930918, 3, ABFA01000076 [3294..3548:RC]
sprFG	small pathogenicity island RNA F and G	2,126,473	2,126,683	211		
txpA	toxin-antitoxin system	2,126,684	2,126,860	177	REVERSE	Identical to FSA084, CCEL01000012.1 [81196..81372]
seA	enterotoxin A	2,126,969	2,127,742	774	REVERSE	Identical to MW2, BA000033 [2051456..2052229:RC]
**Q7A4M7**	putative bacteriophagal protein from *Sipho-/Biseptimavirus*	2,128,115	2,128,489	375	REVERSE	Identical to N315, BA000018 [2012574..2012948:RC]
N/A	hypothetic phage protein, SAS1874	2,128,545	2,128,832	288	REVERSE	Identical to MSSA476, BX571857 [2032278..2032565:RC]
N/A	hypothetic phage protein, SAS1875	2,128,878	2,129,030	153	REVERSE	Identical to MSSA476, BX571857 [2032611..2032763:RC]
***pep*/minor**	phage tail peptidase/bacteriophage minor structural protein from *Sipho-/Biseptimavirus*	2,129,020	2,132,805	3786	REVERSE	

Gen ID	Description	Start in genome	End in genome	Length	Direction	Comments
**Q8SDK3**	putative bacteriophagal protein	2,132,821	2,134,311	1491	REVERSE	
***tmp*-1_phiPVL**	putative membrane protein, SAS1878	2,134,311	2,138,960	4650	REVERSE	
N/A	hypothetic phage protein, SAS1879	2,139,016	2,139,138	123	REVERSE	
N/A	hypothetic protein, UG86_10255	2,139,198	2,139,644	447	REVERSE	
**matp**	major phage tail protein, UG86_10260	2,139,711	2,140,664	954	REVERSE	
N/A	HK97 gp10 family phage protein, UG86_10270	2,141,042	2,141,419	378	REVERSE	
hdtA	head-tail adaptor protein, UG86_10275	2,141,419	2,141,754	336	REVERSE	
htcp	phage head-tail connector protein, UG86_10280	2,141,741	2,142,073	333	REVERSE	Identical to 93bS9, CP010952 [2055843..2056175:RC]
N/A	hypothetic protein, UG86_10285	2,142,082	2,142,240	159	REVERSE	Identical to 93bS9, CP010952 [2056184..2056342:RC]
macp	major capsid protein, SAS1887	2,142,276	2,143,523	1248	REVERSE	Identical to MSSA476, BX571857 [2046007..2047254:RC]
**protease**	putative prohead protease, SAS1888	2,143,611	2,144,207	597	REVERSE	Identical to MSSA476, BX571857 [2047342..2047938:RC]
**portal**	portal protein, UG86_10300	2,144,188	2,145,438	1251	REVERSE	Identical to 93bS9, CP010952 [2058290..2059540:RC]
N/A	hypothetic phage protein, SAS1890	2,145,444	2,145,644	201	REVERSE	Identical to MSSA476, BX571857 [2049175..2049375:RC]
**terminase-L**	putative terminase, large subunit, SAS1891	2,145,658	2,147,352	1695	REVERSE	
N/A	hypothetic phage protein, SAS1892	2,147,355	2,147,822	468	REVERSE	Identical to MSSA476, BX571857 [2051086..2051553:RC]
***nuc*-HNH**	HNH endonuclease, UG86_10320	2,147,951	2,148,304	354	REVERSE	Identical to 93bS9, CP010952 [2062054..2062407:RC]
N/A	hypothetic phage protein, SAS1894	2,148,311	2,148,763	453	REVERSE	Identical to MSSA476, BX571857 [2052042..2052494:RC]
N/A	hypothetic phage protein, SAS1895	2,148,878	2,149,348	471	REVERSE	Identical to MSSA476, BX571857 [2052609..2053079:RC]
**Q4ZCN3**	hypothetic protein, UG86_10340	2,149,371	2,149,571	201	REVERSE	

Gen ID	Description	Start in genome	End in genome	Length	Direction	Comments
rinB	transcriptional activator	2,149,571	2,149,720	150	REVERSE	Identical to A9765, ACSN01000033 [29345..29494:RC]
N/A	hypothetic phage protein from *Sipho-/Phietavirus*	2,149,720	2,150,106	387	REVERSE	
N/A	hypothetic phage protein from *Sipho-/Phietavirus*	2,150,096	2,150,332	237	REVERSE	
**DUF1381**	DUF1381 domain-containing protein from *Siphoviridae*	2,150,525	2,150,731	207	REVERSE	
dut	dUTPase from *Siphoviridae*	2,150,768	2,151,304	537	REVERSE	
**DUF1024**	DUF1024 family protein, replication module of *Siphoviridae*	2,151,297	2,151,545	249	REVERSE	
N/A	phage protein, UG86_01510	2,151,538	2,151,822	285	REVERSE	
N/A	hypothetic phage protein, SAS0915	2,152,456	2,152,860	405	REVERSE	Identical to MSSA476, BX571857 [992632..993036]
N/A	hypothetic protein, SACOL0352	2,152,863	2,153,069	207	REVERSE	
N/A	conserved hypothetic protein, SACOL0351	2,153,083	2,153,325	243	REVERSE	
N/A	phage protein, UG86_01500	2,153,331	2,153,585	255	REVERSE	
N/A	phage protein, UG86_10400	2,154,203	2,154,388	186	REVERSE	
**DUF1064**	DUF1064 domain-containing protein from *Siphoviridae*	2,154,393	2,154,797	405	REVERSE	
**DUF3269**	hypothetic protein, SACOL0345	2,154,808	2,155,029	222	REVERSE	
sri	staphylococcal replication inhibitor	2,155,042	2,155,200	159	REVERSE	Identical to A9765, ACSN01000051 [1999..2157:RC]
**DNA-*rep***	DNA-replication from *Sipho-/Phietavirus*	2,155,197	2,155,982	786	REVERSE	
**Q4ZAK4**	putative bacteriophagal protein	2,155,995	2,156,702	708	REVERSE	
N/A	hypothetic protein	2,156,767	2,157,048	282	FORWARD	
N/A	hypothetic phage protein from *Sipho-/Phietavirus*	2,15,7187	2,157,678	492	REVERSE	

Gen ID	Description	Start in genome	End in genome	Length	Direction	Comments
N/A	hypothetic phage protein from *Sipho-/Phietavirus*	2,157,671	2,157,858	188	REVERSE	
***ssbP*-3_phi11**	putative DNA binding protein from *Sipho-/Phietavirus*	2,157,871	2,158,425	555	REVERSE	Identical to ATCC25923, CP009361 [1549530..1550084:RC]
**DUF2483**	lysogeny-assoc. DUF2483 family protein from *Siphoviridae*	2,159,227	2,159,448	222	REVERSE	
**DUF1108**	lysogeny-assoc. DUF1108 family protein from *Siphoviridae*	2,159,458	2,159,718	261	REVERSE	Identical to Phage_88, ORF058 [AY954966.1 [32025..32285]
**DUF1270**	DUF1270 family protein, replication or lysogeny *module of Siphoviridae*	2,159,811	2,159,972	162	REVERSE	Identical to MSSA476, BX571857 [2060830..2060991:RC]
N/A	hypothetic phage protein, SAS1911	2,159,969	2,160,289	321	REVERSE	Identical to MSSA476, BX571857 [2060988..2061308:RC]
N/A	hypothetic phage protein, SAS1912	2,160,345	2,160,980	636	FORWARD	
N/A	hypothetic phage protein, SAS1912a	2,160,995	2,161,135	141	REVERSE	Identical to MSSA476, BX571857 [2062014..2062154:RC]
**Q931J5_phi**	NCTC13712_00761, Uncharacterized protein	2,161,166	2,161,363	198	REVERSE	Identical to NCTC13712, LR134304 [818444..818641]
ant	antirepressor	2,161,379	2,162,128	750	REVERSE	Identical to MW2, BA000033 [2083152..2083901:RC]
N/A	hypothetic phage protein, SAS1916	2,162,185	2,162,724	540	FORWARD	
N/A	hypothetic phage protein, SAS1917	2,162,748	2,163,029	282	REVERSE	Identical to MSSA476, BX571857 [2063763..2064044:RC]
**DUF739**	putative regulatory protein, SAS1918, DUF739 family protein, lysogeny module of *Siphoviridae*	2,163,026	2,163,250	225	REVERSE	Identical to MSSA476, BX571857 [2064041..2064265:RC]
**rep**	putative repressor, SAS1919	2,163,409	2,164,179	771	FORWARD	Identical to MSSA476, BX571857 [2064424..2065194]
**A6U3A7**	putative bacteriophagal protein, lysogeny-assoc. hypothetic protein from *Siphoviridae*, locus tag SPV-80A_gp05n	2,164,379	2,164,561	183	FORWARD	Identical to COL, CP000046 [356975..357157:RC]
N/A	hypothetic phage protein from *Sipho-/Triavirus*	2,164,618	2,164,743	126	FORWARD	Identical to Phage_47, ORF136 [AY954957.1 [25767..25892:RC]
**Na/K-ATPase**	Na/K ATPase	2,164,740	2,165,354	615	REVERSE	
***int*-2**	integrase from *Siphoviridae*	2,165,462	2,166,499	1038	FORWARD	Identical to Bmb9393, CP005288 [2209561..2210598]
