# Supplementary material for: Characterisation and Molecular Analysis of an Unusual Chimeric Methicillin Resistant Staphylococcus Aureus Strain and its Bacteriophages
Source: Front Genet. 2021 Nov 18;12:723958. doi: 10.3389/fgene.2021.723958 (PMC8638950; doi:10.3389/fgene.2021.723958)
Supplement: Supplementary file 2 [file DataSheet2.PDF]

Supplemental File 2: Susceptibility tests, geno- and phenotypes of antibiotic resistance.

Class	Compound	MIC	Method	Interpretation	Detected genotype	Genotypes *not* detected
**Beta-Lactams**	Bencylpenicillin	>= 0.5 µg/mL	Vitek-2	R	*blaZ*, *mecA* as part of an SCC*mec* IVg element	*mecC*
	Oxacillin	>= 4 µg/mL	Vitek-2	R		
	Cefoxitin		Vitek-2	R		
**Aminoglycosides**	Gentamicin	>= 16 µg/mL	Vitek-2	R	*aacA-aphD*	-
	Tobramycin	>= 16 µg/mL	Vitek-2	R	*aacA-aphD*	*aadD*
**Fluoroquinolones**	Ciprofloxacin	>= 8 µg/mL	Vitek-2	R	mutations **S84L** in *gyrA* and **S80F** in *grlA* (see Figures S5-1 and S5-2)	-
	Levofloxacin	>= 8 µg/mL	Vitek-2	R		
	Moxifloxacin	2	Vitek-2	R		
**Macrolides and lincosamides**	Erythromycin	>= 8 µg/mL	Vitek-2	R	*ermC*	*ermA, ermB, ermF, ermT, lnuA*
	Clindamycin	>= 8 µg/mL	Vitek-2	R		
	Inducible Clin.-resistance	Neg.				
**Oxazolidinone**	Linezolid	2 µg/mL	Vitek-2	S	-	*cfr*
**Glycopeptides**	Teicoplanin	2 µg/mL	Vitek-2	S	N/A	N/A
	Vancomycin	<= 0.5 µg/mL	Vitek-2	S	-	*vanA*
**Tetracyclines**	Tetracycline	<= 1 µg/mL	Vitek-2	S	(*tet38*)*	*tetK, tetL, tetM, tetM-O, tetM-S*
**Others**	Fosfomycin	<= 8 µg/mL	Vitek-2	S	(*fosB*)**	-
	Nitrofurantoin	<= 16 µg/mL	Vitek-2	S	N/A	N/A
	Fusidic acid	<= 0.5 µg/mL	Vitek-2	S	-	*fusB (far1), fusC*
	Mupirocin	<= 2 µg/mL	Vitek-2	S	-	*mupA, mupB*
	Rifampicin	<= 0.5 µg/mL	Vitek-2	S	N/A	N/A
**Sulfonamide plus dihydrofolate reductase inhibitors**	Co-trimoxazole	>= 320 µg/mL	Vitek-2	R	*dfrG* plus mutations **F17L** and **E208K** in the *folP* gene (see Figure S5-3)	*dfrA, dfrD, dfrK*
		> 32 µg/mL***	MIC strip test			
**Dihydrofolate reductase inhibitors**	Trimethoprim	> 32 µg/mL	MIC strip test	R	*dfrG*	*dfrA, dfrD, dfrK*

* Tet 38 can be found in any *S. aureus* and could thus even be regarded as a species marker. Although expression or overexpression might influence tetracycline susceptibility, the mere detection of the gene does not predict tetracycline resistance.

** In our experience (see for instance the Supplemental file to one of our earlier publications: https://doi.org/10.1371/journal.pone.0017936.s003), *fosB* is strictly linked to certain clonal complexes. It is always absent from CC1, CC10, CC22, CC45, ST49, CC59, CC80, CC93, CC97, CC130, CC140, CC188, CC395, CC398 and CC705, but it is always present in CC5, CC8, CC9, CC72 or CC772. Thus, we assume that a mere detection of that gene cannot be used to predict Fosfomycin resistance. This does not rule out that *fosB* might play a role in resistance for instance related to its expression, rather than to its mere presence, or that interactions or combinations with other genes might contribute.

*** 32 µg/mL is the highest concentration on the test stripe and there was no inhibition. Thus, this is *not* a contradiction to the higher value displayed by Vitek.

Figure S5-1: Amino acid sequences of *gyrA*, from RGB-095930, a CC8 and a CC140 reference sequence.

```
                                      10        20        30        40        50        60        70        80        90       100
gyrA|NCTC8325[7005:9668]      MAELPQSRINERNITSEMRESFLDYAMSVIVARALPDVRDGLKPVHRRILYGLNEQGMTPDKSYKKSARIVGDVMGKYHPHGDSSIYEAMVRMAQDFSYR
gyrA ERR1764920[20243:22904]  ....................................................................................................
gyrA RGB95930[7005:9665]      ...................................................................................L................

                                     110       120       130       140       150       160       170       180       190       200
gyrA|NCTC8325[7005:9668]      YPLVDGQGNFGSMDGDGAAAMRYTEARMTKITLELLRDINKDTIDFIDNYDGNEREPSVLPARFPNLLANGASGIAVGMATNIPPHNLTELINGVLSLSK
gyrA ERR1764920[20243:22904]  ....................................................................................................
gyrA RGB95930[7005:9665]      ....................................................................................................

                                     210       220       230       240       250       260       270       280       290       300
gyrA|NCTC8325[7005:9668]      NPDISIAELMEDIEGPDFPTAGLILGKSGIRRAYETGRGSIQMRSRAVIEERGGGRQRIVVTEIPFQVNKARMIEKIAELVRDKKIDGITDLRDETSLRT
gyrA ERR1764920[20243:22904]  ......T.............................................................................................
gyrA RGB95930[7005:9665]      ......T.............................................................................................

                                     310       320       330       340       350       360       370       380       390       400
gyrA|NCTC8325[7005:9668]      GVRVVIDVRKDANASVILNNLYKQTPLQTSFGVNMIALVNGRPKLINLKEALVHYLEHQKTVVRRRTQYNLRKAKDRAHILEGLRIALDHIDEIISTIRE
gyrA ERR1764920[20243:22904]  ....................................................................................................
gyrA RGB95930[7005:9665]      ....................................................................................................

                                     410       420       430       440       450       460       470       480       490       500
gyrA|NCTC8325[7005:9668]      SDTDKVAMESLQQRFKLSEKQAQAILDMRLRRLTGLERDKIEAEYNELLNYISELEAILADEEVLLQLVRDELTEIRDRFGDDRRTEIQLGGFEDLEDED
gyrA ERR1764920[20243:22904]  ....................................................................................................
gyrA RGB95930[7005:9665]      ....................................................................................................

                                     510       520       530       540       550       560       570       580       590       600
gyrA|NCTC8325[7005:9668]      LIPEEQIVITLSHNNYIKRLPVSTYRAQNRGGRGVQGMNTLEEDFVSQLVTLSTHDHVLFFTNKGRVYKLKGYEVPELSRQSKGIPVVNAIELENDEVIS
gyrA ERR1764920[20243:22904]  ....................................................................................................
gyrA RGB95930[7005:9665]      ....................................................................................................

                                     610       620       630       640       650       660       670       680       690       700
gyrA|NCTC8325[7005:9668]      TMIAVKDLESEDNFLVFATKRGVVKRSALSNFSRINRNGKIAISFREDDELIAVRLTSGQEDILIGTSHASLIRFPESTLRPLGRTATGVKGITLREGDE
gyrA ERR1764920[20243:22904]  ....................................................................................................
gyrA RGB95930[7005:9665]      ....................................................................................................

                                     710       720       730       740       750       760       770       780       790       800
gyrA|NCTC8325[7005:9668]      VVGLDVAHANSVDEVLVVTENGYGKRTPVNDYRLSNRGGKGIKTATITERNGNVVCITTVTGEEDLMIVTNAGVIIRLDVADISQNGRAAQGVRLIRLGD
gyrA ERR1764920[20243:22904]  ....................................................................................................
gyrA RGB95930[7005:9665]      ....................................................................................................

                                     810       820       830       840       850       860       870       880       890
gyrA|NCTC8325[7005:9668]      DQFVSTVAKVKEDAEDETNEDEQSTSTVSEDGTEQQREAVVNDETPGNAIHTEVIDSEENDEDGRIEVRQDFMDRVEEDIQQSSDE--E*--
gyrA ERR1764920[20243:22904]  ..............DE.XX......--........................A......V...........................DE.*
gyrA RGB95930[7005:9665]      ..............DE.XX......--........................A......V...........................DE.*
```

Figure S5-2: Amino acid sequences of *grlA*, from RGB-095930 and two CC8 reference sequences.

```
                                        10        20        30        40        50        60        70        80        90       100
                                ....|....|....|....|....|....|....|....|....|....|....|....|....|....|....|....|....|....|....|....|
grlA NCTC8325[1294197:1296599]  VSEIIQDLSLEDVLGDRFGRYSKYIIQERALPDVRDGLKPVQRRILYAMYSSGNTHDKNFRKSAKTVGDVIGQYHPHGDSSVYEAMVRLSQDWKLRHVLI
grlA RGB95930[1368306:1370708]  ...............................................................................F....................
grlA TCH1516[1388114:1390516]   ....................................................................................................

                                       110       120       130       140       150       160       170       180       190       200
                                ....|....|....|....|....|....|....|....|....|....|....|....|....|....|....|....|....|....|....|....|
grlA NCTC8325[1294197:1296599]  EMHGNNGSIDNDPPAAMRYTEAKLSLLAEELLRDINKETVSFIPNYDDTTLEPMVLPSRFPNLLVNGSTGISAGYATDIPPHNLAEVIQATLKYIDNPDI
grlA RGB95930[1368306:1370708]  ....................................................................................................
grlA TCH1516[1388114:1390516]   ....................................................................................................

                                       210       220       230       240       250       260       270       280       290       300
                                ....|....|....|....|....|....|....|....|....|....|....|....|....|....|....|....|....|....|....|....|
grlA NCTC8325[1294197:1296599]  TVNQLMKYIKGPDFPTGGIIQGIDGIKKAYESGKGRIIVRSKVEEETLRNGRKQLIITEIPYEVNKSSLVKRIDELRADKKVDGIVEVRDETDRTGLRIA
grlA RGB95930[1368306:1370708]  ....................................................................................................
grlA TCH1516[1388114:1390516]   ....................................................................................................

                                       310       320       330       340       350       360       370       380       390       400
                                ....|....|....|....|....|....|....|....|....|....|....|....|....|....|....|....|....|....|....|....|
grlA NCTC8325[1294197:1296599]  IELKKDVNSESIKNYLYKNSDLQISYNFNMVAISDGRPKLMGIRQIIDSYLNHQIEVVANRTKFELDNAEKRMHIVEGLIKALSILDKVIELIRSSKNKR
grlA RGB95930[1368306:1370708]  ....................................................................................................
grlA TCH1516[1388114:1390516]   ....................................................................................................

                                       410       420       430       440       450       460       470       480       490       500
                                ....|....|....|....|....|....|....|....|....|....|....|....|....|....|....|....|....|....|....|....|
grlA NCTC8325[1294197:1296599]  DAKENLIEVYEFTEEQAEAIVMLQLYRLTNTDIVALEGEHKELEALIKQLRHILDNHDALLNVIKEELNEIKKKFKSERLSLIEAEIEEIKIDKEVMVPS
grlA RGB95930[1368306:1370708]  ....................................................................................................
grlA TCH1516[1388114:1390516]   ....................................................................................................

                                       510       520       530       540       550       560       570       580       590       600
                                ....|....|....|....|....|....|....|....|....|....|....|....|....|....|....|....|....|....|....|....|
grlA NCTC8325[1294197:1296599]  EEVILSMTRHGYIKRTSIRSFNASGVEDIGLKDGDSLLKHQEVNTQDTVLVFTNKGRYLFIPVHKLADIRWKELGQHVSQIVPIEEDEVVINVFNEKDFN
grlA RGB95930[1368306:1370708]  ....................Y...............................................................................
grlA TCH1516[1388114:1390516]   ....................................................................................................

                                       610       620       630       640       650       660       670       680       690       700
                                ....|....|....|....|....|....|....|....|....|....|....|....|....|....|....|....|....|....|....|....|
grlA NCTC8325[1294197:1296599]  TDAFYVFATQNGMIKKSTVPLFKTTRFNKPLIATKVKENDDLISVMRFEKDQLITVITNKGMSLTYNTSELSDTGLRAAGVKSINLKAEDFVVMTEGVSE
grlA RGB95930[1368306:1370708]  ....................................................................................................
grlA TCH1516[1388114:1390516]   ....................................................................................................

                                       710       720       730       740       750       760       770       780       790       800
                                ....|....|....|....|....|....|....|....|....|....|....|....|....|....|....|....|....|....|....|....|
grlA NCTC8325[1294197:1296599]  NDTILMATQRGSLKRISFKILQVAKRAQRGITLLKELKKNPHRIVAAHVVTGEHSQYTLYSKSNEEHGLINDIHKSEQYTNGSFIVDTDDFGEVIDMYIS
grlA RGB95930[1368306:1370708]  ....................................................................................................
grlA TCH1516[1388114:1390516]   ....................................................................................................
```

Figure S5-3: Amino acid sequences of *folP*, from RGB-095930 and two CC8 reference sequences.

```
                                      10        20        30        40        50        60        70        80        90       100
                              ....|....|....|....|....|....|....|....|....|....|....|....|....|....|....|....|....|....|....|....|
folP NCTC8325 [487922:488725] MTKTKIMGILNVTPDSFSDGGKFNNVESAINRVKAMIDEGADIIDVGGVSTRPGHEMVSLEEEMNRVLPVVEAIVGFDVKISVDTFRSEVAEACLKLGVD
folP RGB095930 [522547:523350] ................L...................................................................................
folP Newman [541867:542670]    ................L...................................................................................

                                     110       120       130       140       150       160       170       180       190       200
                              ....|....|....|....|....|....|....|....|....|....|....|....|....|....|....|....|....|....|....|....|
folP NCTC8325 [487922:488725] MINDQWAGLYDHRMFQIVAKYDAEIILMHNGNGNRDEPVVEEMLTSLLAQAHQAKIAGIPSNKIWLDPGIGFAKTRNEEAEVMARLDELVATEYPVLLAT
folP RGB095930 [522547:523350] ....................................................................................................
folP Newman [541867:542670]    ....................................................................................................

                                     210       220       230       240       250       260
                              ....|....|....|....|....|....|....|....|....|....|....|....|....|...
folP NCTC8325 [487922:488725] SRKRFTKEMMGYDTTPVERDEVTAATTAYGIMKGVRAVRVHNVELNAKLAKGIDFLKENENARHNLS*
folP RGB095930 [522547:523350] .......K...........................................................*
folP Newman [541867:542670]    .......K...........................................................*
```

Figure S5-4: Copy of the original Vitek-2 protocol

bioMerieux-Kunde:
Systemnr.:

Laborbefund

Gedruckt am 30.01.2021 01:57 CET
Gedruckt von: labsuper

***** Angewandter Alarm *****

Referenznummer: Regensburg_ID95930-1

Kartentyp: AST-P608 Testgerät: 000015F14408 (13294)

Keimzahl:
Gewählter Keim: Staphylococcus aureus

**Kommentare:**	AES DETEKTIERTE FUER DIE BETALACTAM-AB DEN PHAENOTYP PBP MODIFIKATION (mecA), DIES ENTSPRICHT MRSA. SEIT DEM 1.7.2009 IST DER NACHWEIS VON MRSA IN BK UND LIQUOR ALS NACHWEIS EINER INFEKTION MELDEPFLICHTIG GEM. IFSG PAR. 7.

**Infos zur Identifizierung**				
**Gewählter Keim**	Staphylococcus aureus			
	**Eingegeben:**	29.01.2021 02:49 CET	**Von:**	labsuper
**Meldungen zur Analyse:** Low-Level-Resistenz - die MHK von 2, 4, 32, 64 für Mupirocin deckt den gesamten Intermediär-Bereich (MHK 2-256) ab.				

Infos zur Resistenz	**Karte:** AST-P608		**Chargenbez:** 4881414403	**Verfallsdatum:**	10.10.2021 13:00 CEST
	**Beendet:** 29.01.2021 11:48 CET		**Status:** Fertig	**Analysen-Dauer:**	9,00 Std.
**Antibiotikum**	**MHK**	**Interpretation**	**Antibiotikum**	**MHK**	**Interpretation**
Cefoxitin-Screen	POS	+	Linezolid	2	S
Benzylpenicillin	>= 0,5	R	Teicoplanin	2	S
Oxacillin	>= 4	R	Vancomycin	<= 0,5	S
Gentamicin	>= 16	R	Tetracyclin	<= 1	S
Tobramycin	>= 16	R	Fosfomycin	<= 8	S
Ciprofloxacin	>= 8	R	Nitrofurantoin	<= 16	S
Levofloxacin	>= 8	R	Fusidinsäure	<= 0,5	S
Moxifloxacin	2	R	Mupirocin	<= 2	S
Induzierbare Clindamycin Resistenz	NEG	-	Rifampicin	<= 0,5	S
Erythromycin	>= 8	R	Trimethoprim/Sulfamethoxazol	>= 320	R
Clindamycin	>= 8	R			

+= Abgeleitete Antibiotika *= AES modifiziert **= Anwender modifiziert

---

Installierte VITEK 2 Systems Version: 07.01
MHK-Interpretationsrichtlinie: EUCAST 2014 + CLSI 2014 D
Therapeutische Interpretationsrichtlinie: DEUTSCHLAND PHENOTYPIC 2014

Bezeichnung des AES-Parametersets: EUCAST/CLSI + PHAENOTYPISCH 2014 D
Letzte Änderung der AES-Parameter: 25.11.2014 12:06 CET
