# Supplementary material for: Characterisation and Molecular Analysis of an Unusual Chimeric Methicillin Resistant Staphylococcus Aureus Strain and its Bacteriophages
Source: Front Genet. 2021 Nov 18;12:723958. doi: 10.3389/fgene.2021.723958 (PMC8638950; doi:10.3389/fgene.2021.723958)
Supplement: Supplementary file 5 [file DataSheet6.PDF]

Locus	Start position in RGB-095930	End position in RGB-095930	Allele in NCTC8325, CP000253.1	Allele in RGB-095930	Allele in ST140_ERR1764920
SAUR0001 (SAR_RS00005)	517	1878	10	324	324
SAUR0002 (SAR_RS00010)	2156	3289	13	29	29
SAUR0003 (SAR_RS00015)	3679	3915	1	1	1
SAUR0004 (SAR_RS00020)	3912	5024	7	292	292
SAUR0005 (SAR_RS00025)	5034	6968	15	430	430
SAUR0006 (SAR_RS00030)	7005	9665	517	494	
SAUR0007 (SAR_RS00035)	9750	10562	9	216	216
SAUR0008 (SAR_RS00040)	10887	12401	16	516	516
SAUR0009 (SAR_RS00045)	12778	14064	8	324	324
SAUR0010 (SAR_RS00050)			9		
SAUR0011 (SAR_RS00055)	15405	15734	11	186	186
SAUR0012 (SAR_RS00060)			264		
SAUR0013 (SAR_RS00065)	17358	18296	13	329	
SAUR0014 (SAR_RS00070)	18311	20278	13	558	558
SAUR0015 (SAR_RS00075)	20275	20721	97	155	155
SAUR0016 (SAR_RS00080)	20753	22153	25	411	411
SAUR0017 (SAR_RS00085)	22433	23716	12	278	278
SAUR0020 (SAR_RS00100)	24913	25614	7	121	121
SAUR0021 (SAR_RS00105)	25627	27453	15	632	632
SAUR0022 (SAR_RS00110)	27446	28780	16	494	494
SAUR0023 (SAR_RS00115)	28781	29569		222	222
SAUR0024 (SAR_RS00120)	31463	32263	16	212	212
SAUR0025 (SAR_RS00125)			245		
SAUR0026 (SAR_RS00130)	35174	35653	48	365	365
SAUR0065 (SAR_RS00325)					
SAUR0106 (SAR_RS00530)	91291	91764	11	171	171
SAUR0107 (SAR_RS00535)	92026	93618	16	435	435
SAUR0109 (SAR_RS00545)	95835	96587	11	232	232
SAUR0110 (SAR_RS00550)	96956	97954	13	311	311
SAUR0111 (SAR_RS00555)			7		
SAUR0112 (SAR_RS00560)	98962	99954	9	262	262
SAUR0113 (SAR_RS00565)	100185	101165	5	242	242
SAUR0114 (SAR_RS00570)	101162	102172	12	287	287
SAUR0115 (SAR_RS00575)			14		
SAUR0116 (SAR_RS00580)	103940	105196	15	408	408
SAUR0117 (SAR_RS00585)	105186	106922	34	487	487
SAUR0118 (SAR_RS00590)	106903	108681	22	532	532
SAUR0119 (SAR_RS00595)	108656	109432	10	251	251
SAUR0120 (SAR_RS00600)	109432	110634	21	355	355
SAUR0121 (SAR_RS00605)	110638	111402	13	243	243
SAUR0122 (SAR_RS00610)	111340	111531	2	4	4
SAUR0124 (SAR_RS00620)	112914	113690	8	212	212
SAUR0125 (SAR_RS00625)			7		
SAUR0126 (SAR_RS00630)	114034	115005	12	183	183
SAUR0127 (SAR_RS00635)	114968	115657	94	172	172
SAUR0128 (SAR_RS00640)	115867	117033	13	418	194
SAUR0129 (SAR_RS00645)	117014	118252	40	23	23
SAUR0130 (SAR_RS00650)	118242	119672	40	438	438
SAUR0131 (SAR_RS00655)	119940	120539	9	4	4
SAUR0133 (SAR_RS00665)	121824	122564	11	226	226
SAUR0134 (SAR_RS00670)	122682	122906	6	36	36
SAUR0135 (SAR_RS00675)	122815	123522	6	265	265
SAUR0136 (SAR_RS00680)	123529	124881	12	374	185
SAUR0138 (SAR_RS00690)	125652	126830	10	325	325
SAUR0139 (SAR_RS00695)	126961	127776	12	195	195
SAUR0140 (SAR_RS00700)	127773	128573	14	273	273

Locus	Start position in RGB-095930	End position in RGB-095930	Allele in NCTC8325, CP000253.1	Allele in RGB-095930	Allele in ST140_ERR1764920
SAUR0141 (SAR_RS00705)	128575	129348	12	314	314
SAUR0142 (SAR_RS00710)	129562	130518	9	333	
SAUR0143 (SAR_RS00715)	130747	132291	14	574	574
SAUR0144 (SAR_RS00720)	132342	133877	13	522	522
SAUR0147 (SAR_RS00735)	136369	138978	413	755	755
SAUR0148 (SAR_RS00740)	139322	139990	10	113	113
SAUR0149 (SAR_RS00745)	140006	140692	15	10	10
SAUR0150 (SAR_RS00750)	140695	141459	14	150	150
SAUR0151 (SAR_RS00755)	141479	143302	27	4	4
SAUR0152 (SAR_RS00760)	143292	144320	199	30	30
SAUR0153 (SAR_RS00765)	144333	145442	13	404	404
SAUR0154 (SAR_RS00770)	145446	146570	15	341	341
SAUR0159 (SAR_RS00795)	150692	151897	17	214	214
SAUR0160 (SAR_RS00800)	151908	152465	9	4	4
SAUR0161 (SAR_RS00805)	152465	153352	14	159	159
SAUR0162 (SAR_RS00810)	153406	154668	13	4	
SAUR0163 (SAR_RS00815)	154745	155890	250	417	417
SAUR0164 (SAR_RS00820)	155955	156281	3	14	14
SAUR0165 (SAR_RS00825)	156288	156671	10	1	1
SAUR0166 (SAR_RS00830)	157098	158585	13	436	436
SAUR0167 (SAR_RS00835)	159233	160192	13	4	4
SAUR0168 (SAR_RS00840)	160254	160457	6	4	4
SAUR0169 (SAR_RS00845)	160637	161149	4	1	1
SAUR0170 (SAR_RS00850)			28		
SAUR0171 (SAR_RS00855)	162246	163220	37	4	4
SAUR0172 (SAR_RS00860)	163217	163978	10	4	4
SAUR0173 (SAR_RS00865)	163988	165022	124	11	11
SAUR0176 (SAR_RS00880)	167184	168434	9	23	23
SAUR0177 (SAR_RS00885)	168881	176056	49	1928	1928
SAUR0178 (SAR_RS00890)	176069	176713	12	24	24
SAUR0179 (SAR_RS00895)	177041	177535	10	196	196
SAUR0180 (SAR_RS00900)					
SAUR0181 (SAR_RS00905)	178568	179809	158	407	
SAUR0182 (SAR_RS00910)	179821	180852	182	300	300
SAUR0183 (SAR_RS00915)			6		
SAUR0184 (SAR_RS00920)	184309	185664	11	357	357
SAUR0185 (SAR_RS00925)	185944	186501	159	209	209
SAUR0186 (SAR_RS00930)	186568	188208	29	542	542
SAUR0187 (SAR_RS00935)	188480	190525	17	534	534
SAUR0188 (SAR_RS00940)	191110	192165	15	249	249
SAUR0189 (SAR_RS00945)	192165	193061	10	240	240
SAUR0190 (SAR_RS00950)	193073	194527	12	529	529
SAUR0191 (SAR_RS00955)	194530	195405	11	158	158
SAUR0193 (SAR_RS00965)	196662	199451	17	844	844
SAUR0195 (SAR_RS00975)	202781	204373	14	631	
SAUR0196 (SAR_RS00980)	204375	205808	13	588	588
SAUR0197 (SAR_RS00985)	205814	206977	16	427	427
SAUR0198 (SAR_RS00990)	206994	208769	15	587	587
SAUR0199 (SAR_RS00995)	208807	210813		215	215
SAUR0200 (SAR_RS01000)	212089	212715	12	257	257
SAUR0201 (SAR_RS01005)	212924	213502	11	239	239
SAUR0202 (SAR_RS01010)	213885	214982	9	346	346
SAUR0203 (SAR_RS01015)	214995	216266	8	438	438
SAUR0204 (SAR_RS01020)	216269	217537	9	4	4
SAUR0205 (SAR_RS01025)	217539	218378	7	4	4
SAUR0206 (SAR_RS01030)	218650	219726	15	409	409

Locus	Start position in RGB-095930	End position in RGB-095930	Allele in NCTC8325, CP000253.1	Allele in RGB-095930	Allele in ST140_ERR1764920
SAUR0207 (SAR_RS01035)	219751	220791	7	348	348
SAUR0208 (SAR_RS01040)	220846	221814	9	295	295
SAUR0209 (SAR_RS01045)	222174	222668	1	1	1
SAUR0210 (SAR_RS01050)	222901	224280	11	367	367
SAUR0211 (SAR_RS01055)	224639	225397	14	4	4
SAUR0212 (SAR_RS01060)	225390	226946	13	499	499
SAUR0213 (SAR_RS01065)	226943	227911	11	354	354
SAUR0214 (SAR_RS01070)	228499	230748	16	426	426
SAUR0215 (SAR_RS01075)	230771	231526	3	229	229
SAUR0216 (SAR_RS01080)					
SAUR0217 (SAR_RS01085)	231848	233611	294	518	518
SAUR0218 (SAR_RS01090)	233775	234119	7	145	145
SAUR0220 (SAR_RS01100)	236473	236580	11	1	1
SAUR0221 (SAR_RS01105)			3		
SAUR0222 (SAR_RS01110)	236878	238062	181	228	228
SAUR0223 (SAR_RS01115)	238092	240353	21	818	
SAUR0224 (SAR_RS01120)	240540	241751	17	445	
SAUR0225 (SAR_RS01125)	241863	243368	13	555	555
SAUR0226 (SAR_RS01130)	243394	244956	11	603	373
SAUR0228 (SAR_RS01140)	246137	247366	14	7	7
SAUR0229 (SAR_RS01145)			14		
SAUR0230 (SAR_RS01150)			3		
SAUR0231 (SAR_RS01155)	252289	252462	6	14	14
SAUR0232 (SAR_RS01160)	252487	253632	106	347	347
SAUR0233 (SAR_RS01165)			10		
SAUR0234 (SAR_RS01170)	254205	255158	14	333	333
SAUR0235 (SAR_RS01175)			2		2
SAUR0236 (SAR_RS01180)	255479	257008	11	588	
SAUR0237 (SAR_RS01185)	257370	258305	8	412	412
SAUR0238 (SAR_RS01190)					
SAUR0255 (SAR_RS01275)	272099	272773	8	32	32
SAUR0256 (SAR_RS01280)	273020	274774	10	444	444
SAUR0257 (SAR_RS01285)	274777	275517	11	118	118
SAUR0258 (SAR_RS01290)	275630	276073	1	1	1
SAUR0259 (SAR_RS01295)	276066	276767	11	20	20
SAUR0261 (SAR_RS01305)	279625	280329	9	179	4
SAUR0262 (SAR_RS01310)	280478	281269	77	77	
SAUR0263 (SAR_RS01315)	281285	282721	17	17	244
SAUR0265 (SAR_RS01325)	283155	283916	11	287	136
SAUR0266 (SAR_RS01330)	283935	284069	6	6	
SAUR0267 (SAR_RS01335)	284167	285081	11	11	4
SAUR0268 (SAR_RS01340)	285109	285513	8	8	
SAUR0269 (SAR_RS01345)	285528	286409	13	341	172
SAUR0270 (SAR_RS01350)	286641	287639	16	16	36
SAUR0273 (SAR_RS01365)	290416	291792	105	515	
SAUR0274 (SAR_RS01370)	292026	293018	247	15	
SAUR0275 (SAR_RS01375)	293344	294294	13	365	
SAUR0276 (SAR_RS01380)	294346	295005	12	12	
SAUR0277 (SAR_RS01385)	295019	295939	13	13	
SAUR0278 (SAR_RS01390)	295936	297102	12	12	
SAUR0279 (SAR_RS01395)	297170	298693	12	12	
SAUR0280 (SAR_RS01400)	300381	301274	11	11	82
SAUR0281 (SAR_RS01405)	301522	301815	4	4	4
SAUR0282 (SAR_RS01410)	301899	304928	16	16	
SAUR0284 (SAR_RS01420)	305358	305600	2	2	2
SAUR0285 (SAR_RS01425)	305613	306947	12	12	

Locus	Start position in RGB-095930	End position in RGB-095930	Allele in NCTC8325, CP000253.1	Allele in RGB-095930	Allele in ST140_ERR1764920
SAUR0286 (SAR_RS01430)					
SAUR0295 (SAR_RS01475)	317642	318142		72	
SAUR0296 (SAR_RS01480)	317642	318142		28	
SAUR0297 (SAR_RS01485)					
SAUR0298 (SAR_RS01490)					
SAUR0299 (SAR_RS01495)					
SAUR0300 (SAR_RS01500)	324312	324710	12	12	
SAUR0301 (SAR_RS01505)	324926	325750	11	11	50
SAUR0302 (SAR_RS01510)	325987	327294	5	5	
SAUR0303 (SAR_RS01515)	327878	328768	8	8	
SAUR0304 (SAR_RS01520)	328785	329018	1	1	
SAUR0305 (SAR_RS01525)	329017	330066	11	11	15
SAUR0306 (SAR_RS01530)	330079	330756	11	11	
SAUR0307 (SAR_RS01535)	330964	331995	11	11	
SAUR0308 (SAR_RS01540)	332338	333456	11	11	
SAUR0309 (SAR_RS01545)	333431	334354	10	10	
SAUR0310 (SAR_RS01550)					97
SAUR0311 (SAR_RS01555)	335690	337222	313	313	206
SAUR0312 (SAR_RS01560)	337262	338143	9	9	21
SAUR0313 (SAR_RS01565)	338301	339161	8	8	
SAUR0314 (SAR_RS01570)	339439	340239	13	13	
SAUR0315 (SAR_RS01575)	340379	341047	12	17	
SAUR0316 (SAR_RS01580)	341173	342486	13	13	
SAUR0317 (SAR_RS01585)	342904	344976	30	30	402
SAUR0318 (SAR_RS01590)	345218	346045	7	7	79
SAUR0319 (SAR_RS01595)					64
SAUR0320 (SAR_RS01600)	347413	347505	27	27	12
SAUR0321 (SAR_RS01605)	347619	348620	223	393	
SAUR0322 (SAR_RS01610)	348654	348986	7	7	1
SAUR0323 (SAR_RS01615)	348987	349787	7	9	40
SAUR0324 (SAR_RS01620)	349777	350721	24	24	
SAUR0325 (SAR_RS01625)	350699	351721	66	66	
SAUR0327 (SAR_RS01635)	353154	354500	11	11	
SAUR0328 (SAR_RS01640)	354515	354799	6	6	2
SAUR0329 (SAR_RS01645)	354801	355244	8	8	4
SAUR0330 (SAR_RS01650)	355249	357204	11	411	
SAUR0331 (SAR_RS01655)	357416	357835	7	7	4
SAUR0332 (SAR_RS01660)	357942	359297	19	19	
SAUR0334 (SAR_RS01670)	359924	361282	24	16	4
SAUR0335 (SAR_RS01675)	361590	362516	9	9	
SAUR0336 (SAR_RS01680)	362530	363591	9	9	
SAUR0337 (SAR_RS01685)	363605	364171	8	8	132
SAUR0338 (SAR_RS01690)	364231	365226	9	9	5
SAUR0340 (SAR_RS01700)	365700	366245	82	82	
SAUR0341 (SAR_RS01705)	366513	367367	10	10	
SAUR0342 (SAR_RS01710)	367364	368593	6	6	367
SAUR0343 (SAR_RS01715)	368574	370286	237	237	
SAUR0344 (SAR_RS01720)	370378	370983	83	83	4
SAUR0345 (SAR_RS01725)	371096	371311	6	6	3
SAUR0346 (SAR_RS01730)	371420	371809	70	70	
SAUR0347 (SAR_RS01735)	372050	372253	48	48	12
SAUR0348 (SAR_RS01740)	372250	372963	83	83	125
SAUR0349 (SAR_RS01745)	372988	373830	102	102	
SAUR0350 (SAR_RS01750)	373830	374459	139	11	4
SAUR0352 (SAR_RS01760)	376367	376525	1	1	18
SAUR0353 (SAR_RS01765)	376498	377679	9	383	

Locus	Start position in RGB-095930	End position in RGB-095930	Allele in NCTC8325, CP000253.1	Allele in RGB-095930	Allele in ST140_ERR1764920
SAUR0355 (SAR_RS01775)	378557	380785	13	13	4
SAUR0356 (SAR_RS01780)	380782	382623	17	17	5
SAUR0357 (SAR_RS01785)	382592	383752	9	9	4
SAUR0358 (SAR_RS01790)	383749	384852	16	16	4
SAUR0359 (SAR_RS01795)	385516	386361	24	24	4
SAUR0360 (SAR_RS01800)	386518	387399	103	103	108
SAUR0361 (SAR_RS01805)	387429	387632	26	26	1
SAUR0362 (SAR_RS01810)	387644	388741	204	204	
SAUR0363 (SAR_RS01815)	388827	389018	4	4	9
SAUR0364 (SAR_RS01820)	389262	389558	1	1	2
SAUR0366 (SAR_RS01830)	390134	390376	1	1	1
SAUR0391 (SAR_RS01955)					
SAUR0392 (SAR_RS01960)	393505	393873	8	8	
SAUR0393 (SAR_RS01965)	394054	394626	11	11	
SAUR0394 (SAR_RS01970)	394764	395027	8	8	
SAUR0395 (SAR_RS01975)	395327	395578	37	37	7
SAUR0396 (SAR_RS01980)	395617	395826	8	8	5
SAUR0397 (SAR_RS01985)	396004	396585	8	8	20
SAUR0398 (SAR_RS01990)	396651	397034	18	18	4
SAUR0399 (SAR_RS01995)	397289	397915	9	9	
SAUR0401 (SAR_RS02005)	398360	399883	11	539	
SAUR0402 (SAR_RS02010)	399899	400468	8	8	4
SAUR0403 (SAR_RS02015)	400974	401729	14	14	
SAUR0404 (SAR_RS02020)	401810	403198	35	582	350
SAUR0405 (SAR_RS02025)	403283	403384	1	1	32
SAUR0406 (SAR_RS02030)	404175	405131	10	10	24
SAUR0407 (SAR_RS02035)	405249	405911	11	44	4
SAUR0408 (SAR_RS02040)	406054	406461	8	8	1
SAUR0409 (SAR_RS02045)	406974	407552	8	8	
SAUR0410 (SAR_RS02050)	407552	408820	16	16	
SAUR0411 (SAR_RS02055)	408858	410324	11	431	
SAUR0412 (SAR_RS02060)	410349	411890	12	12	
SAUR0417 (SAR_RS02085)	414568	414720	17	17	3
SAUR0418 (SAR_RS02090)	415244	415603	43	43	
SAUR0419 (SAR_RS02095)	415622	416467	27	27	
SAUR0420 (SAR_RS02100)	416939	417619	21	21	
SAUR0421 (SAR_RS02105)	417905	418600	58	58	
SAUR0426 (SAR_RS02130)	422591	423286	20	20	
SAUR0427 (SAR_RS02135)					8
SAUR0428 (SAR_RS02140)	425772	426455	25	25	138
SAUR0433 (SAR_RS02165)					
SAUR0434 (SAR_RS02170)	433504	433812	9	9	
SAUR0440 (SAR_RS02200)					
SAUR0442 (SAR_RS02210)					
SAUR0443 (SAR_RS02215)	444022	444345	9	7	4
SAUR0444 (SAR_RS02220)	444364	444678	3	3	25
SAUR0445 (SAR_RS02225)	444968	445162	15	15	5
SAUR0446 (SAR_RS02230)	445347	446549	18	18	
SAUR0448 (SAR_RS02240)	448137	449621	79	79	
SAUR0449 (SAR_RS02245)	449634	452339	56	56	
SAUR0450 (SAR_RS02250)	452501	452863	7	7	52
SAUR0451 (SAR_RS02255)					
SAUR0452 (SAR_RS02260)	453546	454220	9	9	
SAUR0453 (SAR_RS02265)	454257	454991	10	10	
SAUR0454 (SAR_RS02270)			13		
SAUR0455 (SAR_RS02275)	455447	456784	197	9	

Locus	Start position in RGB-095930	End position in RGB-095930	Allele in NCTC8325, CP000253.1	Allele in RGB-095930	Allele in ST140_ERR1764920
SAUR0456 (SAR_RS02280)					4
SAUR0457 (SAR_RS02285)	457899	459041	11	61	
SAUR0458 (SAR_RS02290)	459335	460360	9	9	
SAUR0459 (SAR_RS02295)	460364	461023	8	302	159
SAUR0460 (SAR_RS02300)	461060	461902	24	24	80
SAUR0462 (SAR_RS02310)	463423	463692	4	4	1
SAUR0463 (SAR_RS02315)	463841	464236	9	9	3
SAUR0464 (SAR_RS02320)	464226	464711	7	7	4
SAUR0465 (SAR_RS02325)	464880	465662	13	13	130
SAUR0466 (SAR_RS02330)	465659	466771	10	10	29
SAUR0467 (SAR_RS02335)	466892	467776	6	6	
SAUR0468 (SAR_RS02340)	467957	472456	23	279	
SAUR0469 (SAR_RS02345)	472474	473937	300	9	
SAUR0471 (SAR_RS02355)	474769	476196	9	9	484
SAUR0472 (SAR_RS02360)	476260	477900	22	17	4
SAUR0473 (SAR_RS02365)	477925	478653	10	10	4
SAUR0474 (SAR_RS02370)	479053	479154	9	9	3
SAUR0475 (SAR_RS02375)	479292	479816	6	6	96
SAUR0476 (SAR_RS02380)	479885	481582	10	10	
SAUR0477 (SAR_RS02385)	481672	481989	2	2	1
SAUR0478 (SAR_RS02390)	481996	482592	9	9	
SAUR0484 (SAR_RS02420)	489356	490693	10	10	
SAUR0485 (SAR_RS02425)	490695	491312	7	7	135
SAUR0486 (SAR_RS02430)	491340	491669	4	4	1
SAUR0487 (SAR_RS02435)	491788	491964	4	4	1
SAUR0488 (SAR_RS02440)	491883	492809	5	5	195
SAUR0489 (SAR_RS02445)	492810	493613	7	7	4
SAUR0490 (SAR_RS02450)	493630	493977	1	1	1
SAUR0491 (SAR_RS02455)	494251	494976	9	9	
SAUR0492 (SAR_RS02460)	494969	495217	1	68	1
SAUR0493 (SAR_RS02465)	495219	496058	12	12	
SAUR0494 (SAR_RS02470)	496083	496238	1	1	1
SAUR0495 (SAR_RS02475)	496343	498316	12	12	
SAUR0496 (SAR_RS02480)	498347	499120	8	8	1
SAUR0497 (SAR_RS02485)	499287	499823	6	6	1
SAUR0498 (SAR_RS02490)	499834	500727	8	8	165
SAUR0499 (SAR_RS02495)	500827	501090	1	1	1
SAUR0500 (SAR_RS02500)	501400	502248	14	14	130
SAUR0501 (SAR_RS02505)	502262	503086	5	5	2
SAUR0502 (SAR_RS02510)	503103	503483	10	10	
SAUR0503 (SAR_RS02515)	503555	503857	2	2	
SAUR0504 (SAR_RS02520)	504047	504178	3	3	2
SAUR0505 (SAR_RS02525)	504201	505553	266	11	221
SAUR0506 (SAR_RS02530)	505700	506665	8	8	97
SAUR0507 (SAR_RS02535)	506723	506833	1	1	1
SAUR0508 (SAR_RS02540)	506815	507468	5	5	
SAUR0509 (SAR_RS02545)	507779	508351	9	9	4
SAUR0510 (SAR_RS02550)	508351	511857	19	19	
SAUR0511 (SAR_RS02555)	511847	513373	9	9	265
SAUR0512 (SAR_RS02560)			11		
SAUR0513 (SAR_RS02565)	514562	514825	6	6	1
SAUR0514 (SAR_RS02570)	514843	515235	7	7	3
SAUR0515 (SAR_RS02575)	515340	515741	5	5	4
SAUR0516 (SAR_RS02580)	515921	517216	9	17	
SAUR0517 (SAR_RS02585)	517221	517760	1	1	4
SAUR0518 (SAR_RS02590)	518017	520110	9	10	301

Locus	Start position in RGB-095930	End position in RGB-095930	Allele in NCTC8325, CP000253.1	Allele in RGB-095930	Allele in ST140_ERR1764920
SAUR0519 (SAR_RS02595)	520339	521220	10	10	170
SAUR0520 (SAR_RS02600)	521399	522331	9	9	
SAUR0521 (SAR_RS02605)	522547	523350	88	93	10
SAUR0522 (SAR_RS02610)	523328	523693	5	5	
SAUR0523 (SAR_RS02615)	523690	524166	7	7	
SAUR0524 (SAR_RS02620)	524560	524652	2	2	
SAUR0525 (SAR_RS02625)	524705	526192	13	13	
SAUR0541 (SAR_RS02705)	538426	539808	8	8	
SAUR0542 (SAR_RS02710)	539912	540799	9	9	
SAUR0543 (SAR_RS02715)	540803	541363	6	6	3
SAUR0544 (SAR_RS02720)					
SAUR0545 (SAR_RS02725)	541571	542785	8	277	
SAUR0546 (SAR_RS02730)	542943	543404	6	6	1
SAUR0547 (SAR_RS02735)	543423	543989	12	7	1
SAUR0548 (SAR_RS02740)	543979	544986	9	9	
SAUR0549 (SAR_RS02745)	545000	547456	13	697	
SAUR0550 (SAR_RS02750)	547384	547542	4	6	
SAUR0551 (SAR_RS02755)	547941	549305	10	10	
SAUR0552 (SAR_RS02760)	549330	550403	13	13	200
SAUR0553 (SAR_RS02765)	550960	552414	11	11	
SAUR0554 (SAR_RS02770)	552843	553484	5	5	13
SAUR0555 (SAR_RS02775)	553468	554868	14	14	4
SAUR0556 (SAR_RS02780)	554918	555265	6	6	
SAUR0557 (SAR_RS02785)	555273	556019	8	8	4
SAUR0558 (SAR_RS02790)	556019	556543	7	7	4
SAUR0559 (SAR_RS02795)	556624	557193	11	11	5
SAUR0560 (SAR_RS02800)	557507	557689	1	5	1
SAUR0561 (SAR_RS02805)	557702	558250	4	4	4
SAUR0562 (SAR_RS02810)	558431	558853	2	2	2
SAUR0563 (SAR_RS02815)					
SAUR0564 (SAR_RS02820)	560025	560525	1	1	1
SAUR0565 (SAR_RS02825)	560568	560936	5	5	3
SAUR0566 (SAR_RS02830)	561111	561719	8	302	23
SAUR0567 (SAR_RS02835)	561934	565485	18	18	651
SAUR0568 (SAR_RS02840)	565622	569245	13	13	
SAUR0569 (SAR_RS02845)	569382	569636	1	1	
SAUR0570 (SAR_RS02850)	569734	570147	1	1	1
SAUR0571 (SAR_RS02855)	570213	570683	5	101	10
SAUR0572 (SAR_RS02860)	570806	572887	10	10	
SAUR0573 (SAR_RS02865)	573104	574288	8	8	
SAUR0574 (SAR_RS02870)	574570	575745	15	15	
SAUR0575 (SAR_RS02875)	575916	577103	9	9	
SAUR0576 (SAR_RS02880)	577369	578247	14	14	
SAUR0577 (SAR_RS02885)	578407	580044	11	11	
SAUR0578 (SAR_RS02890)	580258	581223	7	7	4
SAUR0579 (SAR_RS02895)	581558	582634	8	312	18
SAUR0580 (SAR_RS02900)	582884	583567	7	7	13
SAUR0581 (SAR_RS02905)	583683	584345	8	8	85
SAUR0582 (SAR_RS02910)	584338	584955	4	4	92
SAUR0583 (SAR_RS02915)	585022	585492	6	6	79
SAUR0584 (SAR_RS02920)	585639	586508	9	9	185
SAUR0585 (SAR_RS02925)	586529	587095	4	4	132
SAUR0586 (SAR_RS02930)	587526	590369		988	
SAUR0588 (SAR_RS02940)	598840	600312	58	41	
SAUR0589 (SAR_RS02945)	600436	601926	10	10	
SAUR0590 (SAR_RS02950)	602208	603086	9	9	65

Locus	Start position in RGB-095930	End position in RGB-095930	Allele in NCTC8325, CP000253.1	Allele in RGB-095930	Allele in ST140_ERR1764920
SAUR0591 (SAR_RS02955)	603099	603764	7	58	17
SAUR0592 (SAR_RS02960)	603778	604137	8	8	4
SAUR0593 (SAR_RS02965)	604416	605174	171	6	4
SAUR0594 (SAR_RS02970)	605251	605883	46	6	
SAUR0595 (SAR_RS02975)	605885	606433	8	8	4
SAUR0596 (SAR_RS02980)	606546	607193	158	158	159
SAUR0597 (SAR_RS02985)	607695	609095	10	444	
SAUR0598 (SAR_RS02990)	609639	611015	11	11	
SAUR0599 (SAR_RS02995)	611017	612156	11	522	259
SAUR0600 (SAR_RS03000)	612131	612496	7	7	4
SAUR0601 (SAR_RS03005)	612499	612768	49	11	4
SAUR0602 (SAR_RS03010)	612969	613136	2	2	2
SAUR0603 (SAR_RS03015)	613643	614473	8	8	
SAUR0604 (SAR_RS03020)	614657	615313	9	9	4
SAUR0605 (SAR_RS03025)	615314	615694	11	11	10
SAUR0606 (SAR_RS03030)	615827	616195	7	7	4
SAUR0607 (SAR_RS03035)	616316	617800	15	425	264
SAUR0608 (SAR_RS03040)	617942	618394	68	68	45
SAUR0609 (SAR_RS03045)	618407	619171	9	9	4
SAUR0610 (SAR_RS03050)	619720	620472	10	10	129
SAUR0611 (SAR_RS03055)	620640	621626	11	339	
SAUR0612 (SAR_RS03060)	621629	622465	8	8	4
SAUR0613 (SAR_RS03065)	623052	623972	9	57	
SAUR0614 (SAR_RS03070)	623977	624960	8	329	160
SAUR0615 (SAR_RS03075)	624973	626049	9	9	169
SAUR0616 (SAR_RS03080)	626226	626567	3	3	71
SAUR0617 (SAR_RS03085)	626619	627941	35	14	
SAUR0618 (SAR_RS03090)	627928	628368	34	34	
SAUR0619 (SAR_RS03095)	628426	628527	5	5	5
SAUR0620 (SAR_RS03100)	628993	630402	99	88	
SAUR0623 (SAR_RS03115)	639557	640183	31	31	
SAUR0625 (SAR_RS03125)	640453	641391	8	8	
SAUR0626 (SAR_RS03130)	641602	642138	2	2	
SAUR0627 (SAR_RS03135)	642307	642783	1	1	
SAUR0628 (SAR_RS03140)	642823	644118	9	9	
SAUR0629 (SAR_RS03145)	644161	644667	8	8	5
SAUR0630 (SAR_RS03150)	645166	646176	10	80	177
SAUR0631 (SAR_RS03155)	646251	646358	3	3	
SAUR0632 (SAR_RS03160)	646451	646867	4	4	55
SAUR0633 (SAR_RS03165)	646864	648525	13	430	
SAUR0634 (SAR_RS03170)	648914	649549	21	21	4
SAUR0635 (SAR_RS03175)	649928	650815	10	10	165
SAUR0637 (SAR_RS03185)	651968	652687	5	5	14
SAUR0638 (SAR_RS03190)	652671	653471	10	10	4
SAUR0639 (SAR_RS03195)	653607	654113	5	5	4
SAUR0640 (SAR_RS03200)	654275	654991	8	8	
SAUR0641 (SAR_RS03205)	655160	655948	8	8	4
SAUR0642 (SAR_RS03210)	656199	656573	1	1	1
SAUR0643 (SAR_RS03215)	657521	658450	84	84	
SAUR0644 (SAR_RS03220)	658645	658869	7	7	36
SAUR0645 (SAR_RS03225)	658886	659089	40	40	1
SAUR0646 (SAR_RS03230)	659248	659808	57	52	2
SAUR0647 (SAR_RS03235)	659827	662229	101	101	
SAUR0648 (SAR_RS03240)	662216	662641	40	58	2
SAUR0649 (SAR_RS03245)	662638	662982	27	27	2
SAUR0650 (SAR_RS03250)	662972	664468	6	6	232

Locus	Start position in RGB-095930	End position in RGB-095930	Allele in NCTC8325, CP000253.1	Allele in RGB-095930	Allele in ST140_ERR1764920
SAUR0651 (SAR_RS03255)	664469	664951	8	8	
SAUR0652 (SAR_RS03260)	664948	665250	2	2	4
SAUR0653 (SAR_RS03265)					
SAUR0654 (SAR_RS03270)	666002	668044	12	12	4
SAUR0658 (SAR_RS03290)	668938	669867	142	3	4
SAUR0659 (SAR_RS03295)	669864	670700	7	7	5
SAUR0660 (SAR_RS03300)	670694	671437	3	3	3
SAUR0661 (SAR_RS03305)	671559	672203	7	7	
SAUR0662 (SAR_RS03310)	672283	673035	9	9	4
SAUR0663 (SAR_RS03315)	673271	674035	9	9	4
SAUR0664 (SAR_RS03320)	674096	674890	10	10	4
SAUR0665 (SAR_RS03325)	675237	676049	9	9	4
SAUR0666 (SAR_RS03330)	676148	677251	235	10	
SAUR0667 (SAR_RS03335)	677248	678309	13	13	183
SAUR0668 (SAR_RS03340)	678372	678770	3	3	
SAUR0669 (SAR_RS03345)	678887	680182	12	12	
SAUR0670 (SAR_RS03350)	680603	682330	11	11	
SAUR0671 (SAR_RS03355)	682689	683918	12	364	114
SAUR0672 (SAR_RS03360)	684173	684265	1	1	1
SAUR0673 (SAR_RS03365)	684443	685276	9	9	
SAUR0674 (SAR_RS03370)	685558	686355	7	7	
SAUR0675 (SAR_RS03375)	686391	687395	7	7	
SAUR0676 (SAR_RS03380)	687392	688408	7	7	24
SAUR0677 (SAR_RS03385)	688642	689610	9	9	21
SAUR0678 (SAR_RS03390)	689652	690236	6	6	
SAUR0679 (SAR_RS03395)	690229	690591	5	5	4
SAUR0680 (SAR_RS03400)	690707	691204	2	2	3
SAUR0681 (SAR_RS03405)	691660	692727	10	10	4
SAUR0682 (SAR_RS03410)	692878	693921		81	
SAUR0683 (SAR_RS03415)	694086	694274	5	5	
SAUR0684 (SAR_RS03420)	694250	694678	49	49	50
SAUR0685 (SAR_RS03425)	694887	695393	12	12	5
SAUR0686 (SAR_RS03430)	695506	696429	7	7	4
SAUR0687 (SAR_RS03435)	696445	697119	36	36	
SAUR0688 (SAR_RS03440)	697112	698152	74	74	2
SAUR0689 (SAR_RS03445)	698296	699057	95	95	2
SAUR0690 (SAR_RS03450)	699047	700936	228	228	151
SAUR0691 (SAR_RS03455)	701661	702278	6	6	3
SAUR0692 (SAR_RS03460)	702294	703301	9	9	4
SAUR0693 (SAR_RS03465)	703889	704686	6	6	4
SAUR0694 (SAR_RS03470)	705047	705691	8	221	119
SAUR0695 (SAR_RS03475)	706388	706598	2	2	
SAUR0696 (SAR_RS03480)	706595	708538	18	18	
SAUR0697 (SAR_RS03485)	708684	709043	3	3	2
SAUR0698 (SAR_RS03490)	709233	709949	7	7	18
SAUR0699 (SAR_RS03495)	709949	710422	8	8	4
SAUR0701 (SAR_RS03505)	710775	711419	6	6	1
SAUR0702 (SAR_RS03510)	711535	712401	14	14	4
SAUR0703 (SAR_RS03515)	712809	714029	10	16	4
SAUR0704 (SAR_RS03520)					
SAUR0710 (SAR_RS03550)	1891543	1892832	108	465	
SAUR0740 (SAR_RS03700)	715429	715872	7	7	4
SAUR0741 (SAR_RS03705)	715939	716334	11	11	
SAUR0742 (SAR_RS03710)	716472	716771	8	8	4
SAUR0743 (SAR_RS03715)	716851	717393	9	9	145
SAUR0744 (SAR_RS03720)	717489	718055	7	7	4

Locus	Start position in RGB-095930	End position in RGB-095930	Allele in NCTC8325, CP000253.1	Allele in RGB-095930	Allele in ST140_ERR1764920
SAUR0745 (SAR_RS03725)	718057	718515	7	7	2
SAUR0746 (SAR_RS03730)	718518	719201	9	9	4
SAUR0747 (SAR_RS03735)	719373	720248	11	11	129
SAUR0748 (SAR_RS03740)	720467	722098	12	12	
SAUR0749 (SAR_RS03745)	722095	723768	13	13	
SAUR0750 (SAR_RS03750)	723895	724338	6	6	1
SAUR0751 (SAR_RS03755)	724565	725491	8	8	130
SAUR0752 (SAR_RS03760)	725594	726502	11	11	4
SAUR0753 (SAR_RS03765)	726537	726824	6	6	3
SAUR0754 (SAR_RS03770)			22		452
SAUR0755 (SAR_RS03775)	728746	730119	99	7	3
SAUR0756 (SAR_RS03780)	730274	730558	5	5	2
SAUR0757 (SAR_RS03785)	730555	731172	5	5	2
SAUR0758 (SAR_RS03790)	731358	731780	9	9	12
SAUR0759 (SAR_RS03795)	731989	733155	168	168	95
SAUR0760 (SAR_RS03800)	733448	733900	8	8	4
SAUR0761 (SAR_RS03805)	734074	734556	18	18	
SAUR0762 (SAR_RS03810)	734809	735570	10	10	133
SAUR0763 (SAR_RS03815)	735567	736487	6	6	168
SAUR0764 (SAR_RS03820)			199		
SAUR0765 (SAR_RS03825)					
SAUR0766 (SAR_RS03830)	738759	739940	71	8	17
SAUR0767 (SAR_RS03835)	740161	741510	11	294	
SAUR0768 (SAR_RS03840)	741732	742571	8	8	
SAUR0769 (SAR_RS03845)	742703	743686	10	10	
SAUR0770 (SAR_RS03850)	744832	745887	16	16	3
SAUR0771 (SAR_RS03855)	745887	746573	2	2	2
SAUR0772 (SAR_RS03860)	746548	747021	7	7	3
SAUR0773 (SAR_RS03865)	747363	747803	10	10	
SAUR0775 (SAR_RS03875)	748766	749479	9	9	
SAUR0776 (SAR_RS03880)	749483	749902	8	8	
SAUR0777 (SAR_RS03885)	749904	750572	5	5	17
SAUR0778 (SAR_RS03890)	750923	751516	9	9	4
SAUR0779 (SAR_RS03895)	751500	752651	9	9	218
SAUR0780 (SAR_RS03900)	752651	753259	6	6	4
SAUR0781 (SAR_RS03905)	753325	753531	8	8	4
SAUR0782 (SAR_RS03910)	753619	754329	9	260	4
SAUR0783 (SAR_RS03915)	754313	755317	13	13	
SAUR0784 (SAR_RS03920)	755791	757731	11	11	
SAUR0785 (SAR_RS03925)	757873	758031	3	3	4
SAUR0786 (SAR_RS03930)	758008	759885	27	16	
SAUR0787 (SAR_RS03935)	759900	761678	9	15	
SAUR0788 (SAR_RS03940)	761900	762877	11	11	
SAUR0789 (SAR_RS03945)	762870	764384	12	12	
SAUR0790 (SAR_RS03950)	764624	765682	9	9	4
SAUR0791 (SAR_RS03955)	766033	766575	8	12	
SAUR0792 (SAR_RS03960)	767255	768172	8	8	
SAUR0793 (SAR_RS03965)	768263	768364	8	8	
SAUR0794 (SAR_RS03970)	768442	769947	8	8	
SAUR0795 (SAR_RS03975)	770288	770788	5	5	14
SAUR0796 (SAR_RS03980)	770808	771674	6	6	
SAUR0797 (SAR_RS03985)	772475	772873	2	2	
SAUR0798 (SAR_RS03990)	772836	774941	9	9	
SAUR0799 (SAR_RS03995)	775059	776030	6	6	4
SAUR0800 (SAR_RS04000)					
SAUR0801 (SAR_RS04005)	776568	777539	7	7	

Locus	Start position in RGB-095930	End position in RGB-095930	Allele in NCTC8325, CP000253.1	Allele in RGB-095930	Allele in ST140_ERR1764920
SAUR0802 (SAR_RS04010)	777526	778482	14	9	
SAUR0803 (SAR_RS04015)	778479	779240	7	7	4
SAUR0804 (SAR_RS04020)	779359	780387	11	11	144
SAUR0805 (SAR_RS04025)	780704	781018	7	7	63
SAUR0806 (SAR_RS04030)	781036	781959	9	9	
SAUR0807 (SAR_RS04035)	782086	782604	7	7	14
SAUR0808 (SAR_RS04040)	782724	783602	6	6	4
SAUR0809 (SAR_RS04045)	783756	784076	5	5	3
SAUR0810 (SAR_RS04050)	786007	787131	9	9	
SAUR0811 (SAR_RS04055)	787320	788546	10	10	31
SAUR0812 (SAR_RS04060)	788560	789054	9	9	10
SAUR0813 (SAR_RS04065)	789072	789833	9	9	25
SAUR0814 (SAR_RS04070)	790029	791099	12	12	6
SAUR0815 (SAR_RS04075)	791417	792472	9	9	22
SAUR0816 (SAR_RS04080)	792642	793283	10	10	
SAUR0818 (SAR_RS04090)			16		
SAUR0819 (SAR_RS04095)	795732	796406	11	11	
SAUR0820 (SAR_RS04100)	796467	797039	1	1	1
SAUR0821 (SAR_RS04105)	797453	799984	10	10	
SAUR0822 (SAR_RS04110)					
SAUR0823 (SAR_RS04115)	800417	801409	11	11	
SAUR0825 (SAR_RS04125)	801818	802657	9	9	4
SAUR0826 (SAR_RS04130)	802831	803481	9	9	4
SAUR0827 (SAR_RS04135)	803478	803714	5	5	4
SAUR0828 (SAR_RS04140)	803983	805968		12	
SAUR0829 (SAR_RS04145)	805976	808822	12	12	
SAUR0831 (SAR_RS04155)	809594	810526	7	7	
SAUR0832 (SAR_RS04160)	810532	811371	37	43	
SAUR0833 (SAR_RS04165)	811379	811864	8	8	26
SAUR0834 (SAR_RS04170)	811872	813311	17	12	
SAUR0835 (SAR_RS04175)	813378	814313	11	11	
SAUR0836 (SAR_RS04180)	815249	816160	12	12	
SAUR0837 (SAR_RS04185)	816157	817152	8	8	4
SAUR0838 (SAR_RS04190)	817262	818206	9	9	4
SAUR0840 (SAR_RS04200)	818779	819366	8	8	4
SAUR0842 (SAR_RS04210)	819609	820511	10	10	189
SAUR0843 (SAR_RS04215)	821214	821843	11	11	4
SAUR0844 (SAR_RS04220)	822603	823616	8	14	4
SAUR0845 (SAR_RS04225)	823669	824679	7	7	1
SAUR0846 (SAR_RS04230)	824818	826008	10	10	28
SAUR0847 (SAR_RS04235)	826130	826891	158	8	4
SAUR0848 (SAR_RS04240)	826894	828411	10	17	
SAUR0849 (SAR_RS04245)	828541	829845	9	9	
SAUR0850 (SAR_RS04250)	830185	830643	9	9	18
SAUR0851 (SAR_RS04255)	830710	830943	3	3	3
SAUR0852 (SAR_RS04260)	831059	831799	120	120	80
SAUR0853 (SAR_RS04265)	831833	834205	232	241	151
SAUR0854 (SAR_RS04270)	834227	834691	6	6	
SAUR0856 (SAR_RS04280)	836690	837418	3	3	
SAUR0857 (SAR_RS04285)	837857	838009	7	7	
SAUR0858 (SAR_RS04290)	838017	838538	8	8	
SAUR0859 (SAR_RS04295)	838677	839207	11	11	
SAUR0862 (SAR_RS04310)	844351	845373	108	368	
SAUR0864 (SAR_RS04320)	846519	847205	9	9	
SAUR0865 (SAR_RS04325)	847562	847762	1	1	1
SAUR0866 (SAR_RS04330)	848258	848476	5	5	

Locus	Start position in RGB-095930	End position in RGB-095930	Allele in NCTC8325, CP000253.1	Allele in RGB-095930	Allele in ST140_ERR1764920
SAUR0867 (SAR_RS04335)	848538	848822	8	8	
SAUR0868 (SAR_RS04340)	848910	849479	10	10	4
SAUR0869 (SAR_RS04345)	849667	849855	5	5	3
SAUR0870 (SAR_RS04350)	849884	850144	3	3	1
SAUR0871 (SAR_RS04355)	850485	850688	1	1	44
SAUR0872 (SAR_RS04360)	850685	850921	3	3	49
SAUR0873 (SAR_RS04365)	851146	851727	6	8	4
SAUR0874 (SAR_RS04370)	851800	852417	10	10	
SAUR0875 (SAR_RS04375)	852572	853066	1	1	4
SAUR0876 (SAR_RS04380)	853465	853887	8	8	4
SAUR0877 (SAR_RS04385)	854035	854751	10	10	4
SAUR0878 (SAR_RS04390)	854834	855373	11	7	4
SAUR0879 (SAR_RS04395)	855523	855843	5	5	2
SAUR0880 (SAR_RS04400)	855987	856343	2	2	
SAUR0881 (SAR_RS04405)	856501	856881	7	7	3
SAUR0882 (SAR_RS04410)					1
SAUR0883 (SAR_RS04415)	857060	857938	12	12	
SAUR0884 (SAR_RS04420)	858680	859066	6	6	12
SAUR0885 (SAR_RS04425)	859059	859355	6	6	4
SAUR0886 (SAR_RS04430)	859605	860630	10	10	
SAUR0887 (SAR_RS04435)	860623	861318	9	9	
SAUR0888 (SAR_RS04440)	861336	862157	10	10	
SAUR0889 (SAR_RS04445)	878446	878640	2	73	2
SAUR0890 (SAR_RS04450)	878873	879724	9	15	
SAUR0891 (SAR_RS04455)	880052	880813	8	8	200
SAUR0892 (SAR_RS04460)	880911	882218	11	11	
SAUR0893 (SAR_RS04465)	882333	883574	12	121	33
SAUR0894 (SAR_RS04470)			2		4
SAUR0895 (SAR_RS04475)	884167	885564	12	12	
SAUR0896 (SAR_RS04480)	885754	885888	4	4	
SAUR0897 (SAR_RS04485)	885894	886208	7	7	73
SAUR0898 (SAR_RS04490)	886573	887613	12	12	
SAUR0899 (SAR_RS04495)	887627	888694	45	45	
SAUR0900 (SAR_RS04500)	888907	889755	7	307	4
SAUR0901 (SAR_RS04505)	889768	890595	73	262	74
SAUR0902 (SAR_RS04510)	890622	891941	23	527	
SAUR0903 (SAR_RS04515)	892025	892942	10	10	
SAUR0904 (SAR_RS04520)	893072	893455	8	8	1
SAUR0905 (SAR_RS04525)	893534	893800	4	4	4
SAUR0907 (SAR_RS04535)	894332	895111	6	6	
SAUR0908 (SAR_RS04540)	895144	896103	12	12	
SAUR0909 (SAR_RS04545)	896357	896467	1	1	1
SAUR0910 (SAR_RS04550)	896515	896667	1	1	1
SAUR0911 (SAR_RS04555)	896683	898140	10	10	263
SAUR0912 (SAR_RS04560)	898137	899351	8	8	
SAUR0913 (SAR_RS04565)	899369	899605	1	1	1
SAUR0914 (SAR_RS04570)	899602	900777	8	8	
SAUR0915 (SAR_RS04575)	901040	901282	3	3	1
SAUR0916 (SAR_RS04580)	901383	901706	6	6	
SAUR0917 (SAR_RS04585)	901765	902829	8	9	
SAUR0918 (SAR_RS04590)	903146	903382	1	12	1
SAUR0919 (SAR_RS04595)	903395	903754		6	3
SAUR0920 (SAR_RS04600)	904208	905416	5	392	13
SAUR0921 (SAR_RS04605)	905547	907022	13	13	
SAUR0922 (SAR_RS04610)	907433	908749	9	9	224
SAUR0923 (SAR_RS04615)	908768	909142	9	9	2

Locus	Start position in RGB-095930	End position in RGB-095930	Allele in NCTC8325, CP000253.1	Allele in RGB-095930	Allele in ST140_ERR1764920
SAUR0924 (SAR_RS04620)	909198	910352	11	92	4
SAUR0925 (SAR_RS04625)	910601	910957	6	6	4
SAUR0926 (SAR_RS04630)	910935	911228	4	4	3
SAUR0927 (SAR_RS04635)	911228	911707	7	7	
SAUR0928 (SAR_RS04640)	911709	913205	10	10	
SAUR0929 (SAR_RS04645)	913198	913539	6	6	4
SAUR0930 (SAR_RS04650)	913539	913967	4	4	13
SAUR0931 (SAR_RS04655)	913960	916365	9	9	
SAUR0932 (SAR_RS04660)	916496	916879	8	8	4
SAUR0933 (SAR_RS04665)	916943	917536	7	7	19
SAUR0934 (SAR_RS04670)	917952	918329	2	2	3
SAUR0935 (SAR_RS04675)	918691	919818	13	45	278
SAUR0936 (SAR_RS04680)	920126	921316	11	11	203
SAUR0937 (SAR_RS04685)	921425	922669	9	9	186
SAUR0938 (SAR_RS04690)	923116	924045	10	10	208
SAUR0939 (SAR_RS04695)	924287	925666	13	13	
SAUR0940 (SAR_RS04700)	925656	926861	9	9	
SAUR0941 (SAR_RS04705)	927212	928543	25	14	4
SAUR0942 (SAR_RS04710)	928868	929443	18	18	105
SAUR0943 (SAR_RS04715)	929448	929972	188	188	
SAUR0944 (SAR_RS04720)	929988	930563	20	20	
SAUR0945 (SAR_RS04725)	930723	934199	12	12	
SAUR0946 (SAR_RS04730)	934200	937853	15	1047	
SAUR0947 (SAR_RS04735)	938019	938921	8	8	4
SAUR0948 (SAR_RS04740)	939250	939639	70	70	4
SAUR0950 (SAR_RS04750)	939810	941126	10	81	
SAUR0951 (SAR_RS04755)	941178	942002	9	9	
SAUR0952 (SAR_RS04760)	942116	942424	4	4	2
SAUR0953 (SAR_RS04765)	942971	944785	14	648	
SAUR0954 (SAR_RS04770)	946062	948671	235	19	
SAUR0955 (SAR_RS04775)	948730	949599	10	10	4
SAUR0959 (SAR_RS04795)	952574	953089	7	7	
SAUR0961 (SAR_RS04805)	954091	954276	2	2	2
SAUR0962 (SAR_RS04810)	954571	955512	7	7	
SAUR0963 (SAR_RS04815)	955524	956768	9	9	
SAUR0964 (SAR_RS04820)	956823	957194	7	7	1
SAUR0965 (SAR_RS04825)	957437	958363	10	10	1
SAUR0966 (SAR_RS04830)	958363	959433	13	13	301
SAUR0967 (SAR_RS04835)	959450	960532	14	14	
SAUR0968 (SAR_RS04840)	960522	961463	175	175	94
SAUR0969 (SAR_RS04845)	961482	963137	25	25	
SAUR0980 (SAR_RS04900)	968974	969963	6	6	161
SAUR0981 (SAR_RS04905)	970258	970653	1	1	1
SAUR0982 (SAR_RS04910)	971024	971743	9	9	4
SAUR0983 (SAR_RS04915)			15		
SAUR0984 (SAR_RS04920)	972897	974705	12	12	4
SAUR0985 (SAR_RS04925)	975165	975971	13	13	5
SAUR0986 (SAR_RS04930)	975994	976359	6	6	4
SAUR0987 (SAR_RS04935)	976463	977056	10	10	4
SAUR0988 (SAR_RS04940)	977242	977589	7	7	4
SAUR0989 (SAR_RS04945)	977606	978241	7	7	
SAUR0990 (SAR_RS04950)	978258	979067	10	10	
SAUR0991 (SAR_RS04955)	979064	979918	12	12	4
SAUR0992 (SAR_RS04960)	979939	981324	13	13	
SAUR0993 (SAR_RS04965)	981334	983178	11	11	
SAUR0996 (SAR_RS04980)	985848	987416	22	16	

Locus	Start position in RGB-095930	End position in RGB-095930	Allele in NCTC8325, CP000253.1	Allele in RGB-095930	Allele in ST140_ERR1764920
SAUR0997 (SAR_RS04985)	987558	988316	10	10	
SAUR1000 (SAR_RS05000)	988510	989019	7	7	4
SAUR1001 (SAR_RS05005)	989132	990322	10	10	
SAUR1002 (SAR_RS05010)	990300	991475	12	12	9
SAUR1003 (SAR_RS05015)	991908	993389	351	351	
SAUR1004 (SAR_RS05020)	993382	993633	15	15	
SAUR1005 (SAR_RS05025)	993633	995195	9	9	
SAUR1006 (SAR_RS05030)	995497	996300	4	4	
SAUR1007 (SAR_RS05035)	996534	998843	25	14	
SAUR1008 (SAR_RS05040)	998860	1000218	11	11	212
SAUR1009 (SAR_RS05045)	1000357	1001868	10	10	4
SAUR1012 (SAR_RS05060)	1002355	1002924	7	7	5
SAUR1013 (SAR_RS05065)	1003134	1003352	5	5	
SAUR1014 (SAR_RS05070)	1003433	1004419	10	10	
SAUR1015 (SAR_RS05075)	1004618	1004794	1	1	1
SAUR1016 (SAR_RS05080)	1004809	1005411	7	7	1
SAUR1019 (SAR_RS05095)	1006755	1008719	11	11	
SAUR1020 (SAR_RS05100)	1008722	1009042	4	4	61
SAUR1021 (SAR_RS05105)	1009039	1009680	9	9	4
SAUR1022 (SAR_RS05110)	1009768	1009956	7	7	
SAUR1026 (SAR_RS05130)	1012200	1012556	2	2	36
SAUR1027 (SAR_RS05135)	1013033	1013992	7	7	
SAUR1028 (SAR_RS05140)	1014038	1014154	5	5	4
SAUR1029 (SAR_RS05145)	1014233	1014448	5	5	4
SAUR1030 (SAR_RS05150)	1014613	1015164	8	8	
SAUR1031 (SAR_RS05155)	1015216	1016154	15	8	
SAUR1032 (SAR_RS05160)	1016336	1017697	14	14	
SAUR1033 (SAR_RS05165)	1017684	1019357	7	7	
SAUR1034 (SAR_RS05170)	1019344	1020147	8	8	38
SAUR1035 (SAR_RS05175)	1020140	1020961	8	16	4
SAUR1036 (SAR_RS05180)	1021200	1021529	6	6	
SAUR1037 (SAR_RS05185)	1021567	1022748	11	11	
SAUR1038 (SAR_RS05190)	1022830	1023840	52	52	
SAUR1039 (SAR_RS05195)					
SAUR1040 (SAR_RS05200)	1024369	1025523	217	10	
SAUR1041 (SAR_RS05205)	1025719	1026651	7	7	
SAUR1042 (SAR_RS05210)			7		
SAUR1043 (SAR_RS05215)	1027327	1027437	3	3	2
SAUR1045 (SAR_RS05225)	1031505	1031939	6	6	4
SAUR1046 (SAR_RS05230)	1032094	1032564	14	14	4
SAUR1047 (SAR_RS05235)	1032612	1033829	199	399	
SAUR1048 (SAR_RS05240)	1034280	1035473	13	346	
SAUR1049 (SAR_RS05245)	1036010	1036300	2	2	1
SAUR1050 (SAR_RS05250)	1036297	1036902	1	1	1
SAUR1051 (SAR_RS05255)	1036892	1038880	16	16	4
SAUR1052 (SAR_RS05260)	1038880	1039980	11	11	
SAUR1053 (SAR_RS05265)					
SAUR1054 (SAR_RS05270)	1040552	1040869	1	1	1
SAUR1056 (SAR_RS05280)	1041674	1042534	10	309	
SAUR1057 (SAR_RS05285)	1042735	1043217	10	10	4
SAUR1058 (SAR_RS05290)	1043204	1044328	12	12	
SAUR1059 (SAR_RS05295)	1044332	1045036	7	7	4
SAUR1060 (SAR_RS05300)	1045036	1045299	4	4	1
SAUR1061 (SAR_RS05305)	1045301	1045972	11	11	4
SAUR1062 (SAR_RS05310)	1045965	1048154	11	11	
SAUR1063 (SAR_RS05315)	1048133	1049617	13	13	

Locus	Start position in RGB-095930	End position in RGB-095930	Allele in NCTC8325, CP000253.1	Allele in RGB-095930	Allele in ST140_ERR1764920
SAUR1064 (SAR_RS05320)	1049610	1050638	11	11	
SAUR1065 (SAR_RS05325)	1050641	1051207	9	9	
SAUR1066 (SAR_RS05330)	1051222	1052700	13	13	
SAUR1067 (SAR_RS05335)	1052722	1053969	12	12	
SAUR1068 (SAR_RS05340)	1054234	1055040	9	9	4
SAUR1069 (SAR_RS05345)	1055033	1056433	13	496	247
SAUR1070 (SAR_RS05350)	1056448	1057023	6	199	4
SAUR1072 (SAR_RS05360)	1057607	1057741	3	3	1
SAUR1073 (SAR_RS05365)	1057893	1059188	13	13	6
SAUR1074 (SAR_RS05370)	1059613	1060785	9	9	283
SAUR1075 (SAR_RS05375)			22		1
SAUR1076 (SAR_RS05380)	1061535	1061801	4	4	1
SAUR1077 (SAR_RS05385)	1061804	1063522	11	11	
SAUR1078 (SAR_RS05390)	1063759	1063992	6	6	4
SAUR1079 (SAR_RS05395)	1064190	1065551	10	10	4
SAUR1080 (SAR_RS05400)	1065548	1066567	9	9	148
SAUR1081 (SAR_RS05405)	1066700	1067362	8	8	4
SAUR1083 (SAR_RS05415)	1067711	1069408	8	8	
SAUR1084 (SAR_RS05420)	1069408	1069626	1	1	1
SAUR1085 (SAR_RS05425)	1069655	1069777	1	1	1
SAUR1086 (SAR_RS05430)	1070107	1070658	8	194	105
SAUR1087 (SAR_RS05435)	1071023	1071649	9	245	4
SAUR1088 (SAR_RS05440)	1071820	1072932	14	14	4
SAUR1089 (SAR_RS05445)	1072936	1073913	7	7	4
SAUR1090 (SAR_RS05450)	1074004	1075296	249	11	
SAUR1091 (SAR_RS05455)	1075300	1076706	14	7	4
SAUR1092 (SAR_RS05460)	1076874	1077149	4	4	2
SAUR1093 (SAR_RS05465)	1077293	1077832	6	6	4
SAUR1094 (SAR_RS05470)	1077845	1078939	7	7	
SAUR1095 (SAR_RS05475)	1078932	1079729	9	9	
SAUR1096 (SAR_RS05480)	1079735	1080544	11	260	148
SAUR1097 (SAR_RS05485)	1080544	1081617	12	12	
SAUR1098 (SAR_RS05490)	1081691	1082710	10	10	97
SAUR1099 (SAR_RS05495)	1082993	1083412	9	9	
SAUR1100 (SAR_RS05500)	1083494	1084846	12	12	114
SAUR1102 (SAR_RS05510)	1085801	1086628	9	9	4
SAUR1103 (SAR_RS05515)	1086782	1086973	3	3	1
SAUR1104 (SAR_RS05520)	1087075	1088922	13	478	4
SAUR1105 (SAR_RS05525)	1089062	1089229	4	4	1
SAUR1106 (SAR_RS05530)	1089231	1089713	8	8	4
SAUR1107 (SAR_RS05535)	1089854	1090129	4	4	4
SAUR1108 (SAR_RS05540)	1090443	1091669	9	9	4
SAUR1109 (SAR_RS05545)	1092223	1095675	37	17	
SAUR1110 (SAR_RS05550)	1095815	1096726	10	10	4
SAUR1111 (SAR_RS05555)	1097166	1098077	157	16	4
SAUR1112 (SAR_RS05560)	1098102	1098563	7	7	61
SAUR1113 (SAR_RS05565)	1098775	1098978	3	3	
SAUR1114 (SAR_RS05570)	1098890	1099927	10	20	102
SAUR1115 (SAR_RS05575)	1099943	1100377	9	9	4
SAUR1116 (SAR_RS05580)	1100440	1101366	122	122	
SAUR1117 (SAR_RS05585)	1102679	1102933	1	6	1
SAUR1118 (SAR_RS05590)	1102936	1103325	6	6	4
SAUR1119 (SAR_RS05595)	1103395	1103937	9	9	
SAUR1120 (SAR_RS05600)	1103939	1104421	3	3	8
SAUR1121 (SAR_RS05605)	1104483	1105622	6	6	
SAUR1122 (SAR_RS05610)	1105749	1106306	6	6	

Locus	Start position in RGB-095930	End position in RGB-095930	Allele in NCTC8325, CP000253.1	Allele in RGB-095930	Allele in ST140_ERR1764920
SAUR1123 (SAR_RS05615)	1106218	1106442	1	1	1
SAUR1124 (SAR_RS05620)	1106386	1106559		1	2
SAUR1125 (SAR_RS05625)	1106713	1108650	240	240	
SAUR1126 (SAR_RS05630)	1108853	1109905	45	45	
SAUR1127 (SAR_RS05635)	1110114	1110797	27	58	
SAUR1128 (SAR_RS05640)	1110797	1111873	11	11	
SAUR1129 (SAR_RS05645)	1111870	1112748	6	6	99
SAUR1130 (SAR_RS05650)	1112758	1113726		9	
SAUR1132 (SAR_RS05660)	1114541	1114864	10	10	6
SAUR1133 (SAR_RS05665)	1114901	1115008	4	4	
SAUR1134 (SAR_RS05670)	1115248	1115988	8	8	
SAUR1135 (SAR_RS05675)	1116369	1117427	10	16	
SAUR1136 (SAR_RS05680)	1117427	1119829	17	17	
SAUR1137 (SAR_RS05685)	1120000	1120938	7	7	
SAUR1138 (SAR_RS05690)	1121308	1121574		5	1
SAUR1139 (SAR_RS05695)	1121575	1122096	3	3	5
SAUR1140 (SAR_RS05700)	1122169	1123881	12	12	
SAUR1141 (SAR_RS05705)	1123891	1126239	13	13	
SAUR1142 (SAR_RS05710)	1126412	1126726	6	6	
SAUR1143 (SAR_RS05715)	1126789	1126884	9	9	17
SAUR1144 (SAR_RS05720)	1127050	1128831	12	12	4
SAUR1145 (SAR_RS05725)	1129155	1129769	7	7	17
SAUR1146 (SAR_RS05730)	1129821	1131587	10	430	
SAUR1147 (SAR_RS05735)	1131587	1132402	6	6	
SAUR1148 (SAR_RS05740)			7		
SAUR1149 (SAR_RS05745)	1134890	1135477	8	8	
SAUR1150 (SAR_RS05750)	1135470	1135973	6	6	81
SAUR1151 (SAR_RS05755)	1136101	1136268	14	14	3
SAUR1152 (SAR_RS05760)	1136462	1136791	85	85	
SAUR1155 (SAR_RS05775)	1138233	1138739	8	8	
SAUR1156 (SAR_RS05780)	1138998	1139495	6	6	
SAUR1157 (SAR_RS05785)	1139649	1139999	14	14	
SAUR1158 (SAR_RS05790)	1140821	1141006	20	20	1
SAUR1161 (SAR_RS05805)	1142338	1143297	16	16	4
SAUR1162 (SAR_RS05810)	1143970	1144116	2	2	
SAUR1163 (SAR_RS05815)	1144100	1144297	12	12	2
SAUR1165 (SAR_RS05825)	1144798	1145514	52	52	
SAUR1166 (SAR_RS05830)	1145622	1146347	37	37	
SAUR1167 (SAR_RS05835)	1146442	1147167	54	54	26
SAUR1168 (SAR_RS05840)	1147605	1148606	12	12	
SAUR1169 (SAR_RS05845)	1148629	1149561	11	11	260
SAUR1170 (SAR_RS05850)	1149733	1151289	15	15	
SAUR1171 (SAR_RS05855)	1151597	1151824	6	6	4
SAUR1172 (SAR_RS05860)	1152129	1153076	27	10	179
SAUR1173 (SAR_RS05865)	1153316	1153513	12	12	14
SAUR1175 (psmβ1)	1154425	1154559	4	4	1
SAUR1176 (SAR_RS05880)	1154883	1155569	7	7	
SAUR1177 (SAR_RS05885)	1155678	1156118	2	2	3
SAUR1178 (SAR_RS05890)	1156313	1157926		8	552
SAUR1179 (SAR_RS05895)	1158071	1158502	1	1	
SAUR1180 (SAR_RS05900)	1158518	1159453	7	7	25
SAUR1181 (SAR_RS05905)	1159467	1159868	1	1	1
SAUR1182 (SAR_RS05910)	1159849	1162083	14	861	
SAUR1183 (SAR_RS05915)	1162375	1163340	11	11	29
SAUR1184 (SAR_RS05920)	1163342	1164691	11	11	146
SAUR1185 (SAR_RS05925)	1164707	1166026	16	16	37

Locus	Start position in RGB-095930	End position in RGB-095930	Allele in NCTC8325, CP000253.1	Allele in RGB-095930	Allele in ST140_ERR1764920
SAUR1186 (SAR_RS05930)	1166132	1167544	9	9	4
SAUR1187 (SAR_RS05935)	1167577	1168749	12	12	4
SAUR1188 (SAR_RS05940)	1169009	1169800	9	9	4
SAUR1189 (SAR_RS05945)	1169818	1170492	11	11	4
SAUR1190 (SAR_RS05950)			2		2
SAUR1191 (SAR_RS05955)	1171064	1171354	6	6	4
SAUR1192 (SAR_RS05960)	1171437	1172243	8	8	4
SAUR1193 (SAR_RS05965)					
SAUR1194 (SAR_RS05970)	1173105	1175858	13	13	
SAUR1196 (SAR_RS05980)	1176148	1176945	11	11	
SAUR1197 (SAR_RS05985)	1177769	1178260	4	29	1
SAUR1198 (SAR_RS05990)	1178260	1179177	8	8	4
SAUR1199 (SAR_RS05995)	1180651	1181178	9	9	4
SAUR1200 (SAR_RS06000)	1181396	1182703	15	15	
SAUR1201 (SAR_RS06005)	1182731	1183612	12	12	
SAUR1202 (SAR_RS06010)	1183630	1184904	13	21	4
SAUR1203 (SAR_RS06015)	1184906	1186006	9	9	
SAUR1204 (SAR_RS06020)	1185999	1189172	12	943	
SAUR1205 (SAR_RS06025)	1189282	1189974	10	10	132
SAUR1206 (SAR_RS06030)	1189974	1190585	9	9	
SAUR1207 (SAR_RS06035)	1190615	1190827	5	7	1
SAUR1208 (SAR_RS06040)	1191265	1191666	8	8	3
SAUR1209 (SAR_RS06045)	1191929	1193626	315	13	
SAUR1210 (SAR_RS06050)	1193901	1194524	5	5	68
SAUR1211 (SAR_RS06055)	1194524	1194742	2	2	69
SAUR1212 (SAR_RS06060)	1194958	1196157	12	12	
SAUR1213 (SAR_RS06065)	1196157	1198565	13	13	
SAUR1214 (SAR_RS06070)	1199071	1200024	3	272	
SAUR1215 (SAR_RS06075)	1200187	1200390	1	1	3
SAUR1216 (SAR_RS06080)	1200689	1201177	8	8	4
SAUR1217 (SAR_RS06085)	1201170	1202105	12	12	4
SAUR1218 (SAR_RS06090)	1202102	1203409	25	15	
SAUR1219 (SAR_RS06095)	1203412	1204506	11	11	4
SAUR1220 (SAR_RS06100)	1204513	1205256	8	8	4
SAUR1221 (SAR_RS06105)	1205253	1207247	10	10	
SAUR1222 (SAR_RS06110)	1207475	1208350	8	277	
SAUR1223 (SAR_RS06115)	1208351	1208995	9	9	
SAUR1224 (SAR_RS06120)	1209002	1209643	1	1	4
SAUR1225 (SAR_RS06125)	1210024	1210212	1	1	1
SAUR1226 (SAR_RS06130)	1210655	1211029	1	1	1
SAUR1227 (SAR_RS06135)	1211044	1212690	14	14	
SAUR1228 (SAR_RS06140)	1212880	1214940	17	17	
SAUR1229 (SAR_RS06145)	1215158	1215715	5	5	1
SAUR1230 (SAR_RS06150)	1215720	1216706	12	12	28
SAUR1231 (SAR_RS06155)	1216699	1217625	13	459	37
SAUR1232 (SAR_RS06160)	1217618	1218352	7	7	4
SAUR1233 (SAR_RS06165)					
SAUR1234 (SAR_RS06170)	1218595	1218828	1	1	1
SAUR1235 (SAR_RS06175)	1218944	1219675	6	199	
SAUR1236 (SAR_RS06180)	1219822	1223388	16	1042	
SAUR1237 (SAR_RS06185)	1223388	1224638	247	13	
SAUR1238 (SAR_RS06190)	1224625	1224957	4	4	21
SAUR1239 (SAR_RS06195)	1224983	1226350	14	14	
SAUR1240 (SAR_RS06200)	1226785	1227060	1	1	1
SAUR1241 (SAR_RS06205)	1227248	1227751		3	
SAUR1242 (SAR_RS06210)	1227751	1228488	8	8	15

Locus	Start position in RGB-095930	End position in RGB-095930	Allele in NCTC8325, CP000253.1	Allele in RGB-095930	Allele in ST140_ERR1764920
SAUR1243 (SAR_RS06215)	1228591	1228941	1	1	
SAUR1244 (SAR_RS06220)	1229095	1229256	2	2	
SAUR1245 (SAR_RS06225)	1229185	1231791	14	14	
SAUR1246 (SAR_RS06230)	1232192	1233076	4	4	4
SAUR1247 (SAR_RS06235)	1233060	1233827	6	6	
SAUR1248 (SAR_RS06240)	1233936	1235102	11	11	4
SAUR1249 (SAR_RS06245)	1235124	1236032	18	18	32
SAUR1253 (SAR_RS06265)	1239874	1241943	14	14	290
SAUR1254 (SAR_RS06270)	1242099	1243406	9	9	
SAUR1255 (SAR_RS06275)	1248400	1249296	10	10	25
SAUR1256 (SAR_RS06280)	1249293	1249838	10	248	4
SAUR1257 (SAR_RS06285)	1249904	1251307	10	10	265
SAUR1258 (SAR_RS06290)	1251332	1252105	11	11	4
SAUR1259 (SAR_RS06295)	1252156	1252248	9	9	3
SAUR1260 (SAR_RS06300)	1252447	1253214		1	1
SAUR1261 (SAR_RS06305)	1253248	1253361	2	2	2
SAUR1262 (SAR_RS06310)	1253396	1254277	4	4	91
SAUR1263 (SAR_RS06315)	1254414	1255136	12	12	11
SAUR1264 (SAR_RS06320)	1255155	1255709	11	6	3
SAUR1265 (SAR_RS06325)	1256083	1256853	7	7	7
SAUR1266 (SAR_RS06330)	1256860	1257642	9	9	7
SAUR1267 (SAR_RS06335)	1257854	1259140	13	13	
SAUR1268 (SAR_RS06340)	1259160	1260863	15	582	
SAUR1269 (SAR_RS06345)	1261121	1265437	18	18	
SAUR1270 (SAR_RS06350)	1265727	1266194	1	1	4
SAUR1271 (SAR_RS06355)	1266215	1267390	11	11	196
SAUR1272 (SAR_RS06360)	1267411	1267695	2	2	43
SAUR1273 (SAR_RS06365)	1267692	1268009	3	3	67
SAUR1274 (SAR_RS06370)	1268014	1270131	16	16	338
SAUR1275 (SAR_RS06375)	1270517	1270867	1	1	1
SAUR1276 (SAR_RS06380)	1271036	1271953	113	113	61
SAUR1277 (SAR_RS06385)	1271968	1272939	10	10	176
SAUR1278 (SAR_RS06390)	1273054	1273323	1	1	
SAUR1279 (SAR_RS06395)	1273693	1275789	18	18	
SAUR1280 (SAR_RS06400)	1276025	1277698	8	8	381
SAUR1281 (SAR_RS06405)	1277954	1280323	13	13	
SAUR1282 (SAR_RS06410)	1280328	1281041	7	270	26
SAUR1283 (SAR_RS06415)	1281072	1282337	12	12	
SAUR1284 (SAR_RS06420)	1282337	1283623	18	13	
SAUR1285 (SAR_RS06425)	1283623	1284327	10	10	4
SAUR1286 (SAR_RS06430)	1284432	1285259	9	9	
SAUR1287 (SAR_RS06435)	1285278	1285670	1	1	3
SAUR1288 (SAR_RS06440)	1285704	1286282	2	2	2
SAUR1289 (SAR_RS06445)	1286509	1287660	10	42	
SAUR1290 (SAR_RS06450)	1287825	1288868	6	6	
SAUR1291 (SAR_RS06455)	1289222	1290781	14	388	115
SAUR1292 (SAR_RS06460)	1291078	1291293	1	1	4
SAUR1293 (SAR_RS06465)	1291467	1292264	9	9	
SAUR1294 (SAR_RS06470)	1292404	1294164	11	11	
SAUR1295 (SAR_RS06475)	1294165	1295031	13	7	
SAUR1296 (SAR_RS06480)	1295125	1295418	3	3	
SAUR1297 (SAR_RS06485)	1295552	1297096	12	12	4
SAUR1298 (SAR_RS06490)	1297097	1297462	6	6	4
SAUR1299 (SAR_RS06495)	1297489	1297980	8	8	3
SAUR1300 (SAR_RS06500)	1298283	1300901	26	725	
SAUR1302 (SAR_RS06510)	1302929	1303471	9	9	4

Locus	Start position in RGB-095930	End position in RGB-095930	Allele in NCTC8325, CP000253.1	Allele in RGB-095930	Allele in ST140_ERR1764920
SAUR1303 (SAR_RS06515)	1303936	1304754	7	60	128
SAUR1304 (SAR_RS06520)	1304883	1306379	26	12	212
SAUR1305 (SAR_RS06525)	1306537	1308210	10	10	209
SAUR1306 (SAR_RS06530)	1308360	1309274	10	10	96
SAUR1307 (SAR_RS06535)					
SAUR1308 (SAR_RS06540)	1310242	1310475	3	3	1
SAUR1309 (SAR_RS06545)	1310697	1311173	8	8	115
SAUR1310 (SAR_RS06550)	1311285	1312523	14	14	4
SAUR1311 (SAR_RS06555)	1312542	1313780	11	11	
SAUR1312 (SAR_RS06560)	1314026	1314391	1	1	1
SAUR1313 (SAR_RS06565)	1314410	1315750	182	11	
SAUR1352 (SAR_RS06760)	1326518	1327543	12	12	4
SAUR1354 (SAR_RS06770)	1328068	1329549	250	250	
SAUR1359 (SAR_RS06795)	1333139	1333327	6	6	
SAUR1360 (SAR_RS06800)	1333466	1333999	6	6	
SAUR1361 (SAR_RS06805)	1334142	1334996	8	8	4
SAUR1362 (SAR_RS06810)	1335327	1335902	8	8	8
SAUR1363 (SAR_RS06815)	1335956	1337338	226	12	
SAUR1364 (SAR_RS06820)	1337529	1338809	13	13	
SAUR1365 (SAR_RS06825)	1338815	1339876	16	16	200
SAUR1366 (SAR_RS06830)	1339878	1340792	24	24	
SAUR1367 (SAR_RS06835)	1340850	1341653	23	23	4
SAUR1368 (SAR_RS06840)	1341946	1342260	7	7	3
SAUR1369 (SAR_RS06845)	1342235	1342339	1	1	1
SAUR1370 (SAR_RS06850)	1342478	1343932	9	9	
SAUR1371 (SAR_RS06855)	1344136	1345653	12	515	
SAUR1372 (SAR_RS06860)	1345744	1345893	2	2	
SAUR1373 (SAR_RS06865)	1346347	1346616	2	2	2
SAUR1374 (SAR_RS06870)	1346773	1347750	13	375	
SAUR1375 (SAR_RS06875)	1347747	1347851	2	2	5
SAUR1376 (SAR_RS06880)	1347925	1348788	11	11	102
SAUR1377 (SAR_RS06885)	1349168	1349791	8	8	4
SAUR1378 (SAR_RS06890)	1349931	1350167	5	5	4
SAUR1379 (SAR_RS06895)	1350303	1350542	2	2	3
SAUR1380 (SAR_RS06900)	1350663	1352651	15	15	
SAUR1381 (SAR_RS06905)	1352929	1353171	1	1	2
SAUR1383 (SAR_RS06915)	1353350	1353817	5	5	
SAUR1384 (SAR_RS06920)	1353941	1355062	5	5	
SAUR1388 (SAR_RS06940)	1358166	1358528	5	5	4
SAUR1389 (SAR_RS06945)	1358732	1360378	10	10	258
SAUR1390 (SAR_RS06950)	1361270	1363975	55	18	
SAUR1391 (SAR_RS06955)	1364155	1364622	5	5	1
SAUR1392 (SAR_RS06960)	1364829	1365125	6	136	
SAUR1393 (SAR_RS06965)	1365501	1366109	8	8	1
SAUR1394 (SAR_RS06970)	1366315	1368306	19	683	352
SAUR1395 (SAR_RS06975)	1368306	1370708	40	84	
SAUR1396 (SAR_RS06980)	1370836	1370934	7	7	1
SAUR1397 (SAR_RS06985)	1370958	1372418	11	377	4
SAUR1398 (SAR_RS06990)	1372918	1373769	6	6	4
SAUR1399 (SAR_RS06995)	1373744	1373911	1	1	
SAUR1400 (SAR_RS07000)	1373904	1375112	8	8	
SAUR1401 (SAR_RS07005)	1375593	1378115	8	8	
SAUR1402 (SAR_RS07010)	1378318	1378827	10	10	
SAUR1403 (SAR_RS07015)	1378964	1379947	10	10	
SAUR1405 (SAR_RS07025)	1380035	1380220	3	3	3
SAUR1406 (SAR_RS07030)	1380368	1381630	7	7	

Locus	Start position in RGB-095930	End position in RGB-095930	Allele in NCTC8325, CP000253.1	Allele in RGB-095930	Allele in ST140_ERR1764920
SAUR1407 (SAR_RS07035)	1381767	1382858	311	10	261
SAUR1408 (SAR_RS07040)	1383023	1384054	6	8	
SAUR1409 (SAR_RS07045)	1384546	1385952	42	42	
SAUR1410 (SAR_RS07050)	1385949	1386515	57	57	
SAUR1411 (SAR_RS07055)	1386512	1387519	40	40	42
SAUR1412 (SAR_RS07060)	1387521	1388303	3	3	352
SAUR1413 (SAR_RS07065)	1388300	1388935	23	23	243
SAUR1414 (SAR_RS07070)	1388928	1390142	8	76	
SAUR1415 (SAR_RS07075)	1390135	1390863	121	121	
SAUR1416 (SAR_RS07080)	1391185	1392447	9	9	
SAUR1417 (SAR_RS07085)	1392466	1393725	9	9	
SAUR1419 (SAR_RS07095)					
SAUR1420 (SAR_RS07100)	1395820	1396521	12	12	4
SAUR1421 (SAR_RS07105)	1396514	1397287	6	6	171
SAUR1422 (SAR_RS07110)	1397274	1398104	18	18	
SAUR1423 (SAR_RS07115)	1398097	1399083	20	20	
SAUR1425 (SAR_RS07125)	1399387	1399731	2	2	4
SAUR1426 (SAR_RS07130)	1399936	1401750	8	8	
SAUR1427 (SAR_RS07135)	1401890	1402531	7	7	
SAUR1428 (SAR_RS07140)	1402538	1403389	5	5	
SAUR1429 (SAR_RS07145)	1403436	1404353	11	11	6
SAUR1430 (SAR_RS07150)	1404355	1405281	13	13	
SAUR1431 (SAR_RS07155)	1405472	1406455	10	10	
SAUR1432 (SAR_RS07160)	1407427	1408329	11	11	
SAUR1433 (SAR_RS07165)			370		182
SAUR1434 (SAR_RS07170)	1410937	1412142	18	10	
SAUR1435 (SAR_RS07175)	1412206	1413195	10	10	
SAUR1436 (SAR_RS07180)	1413197	1414084	7	380	
SAUR1437 (SAR_RS07185)	1414081	1414803	48	65	116
SAUR1438 (SAR_RS07190)			7		
SAUR1439 (SAR_RS07195)	1415692	1416843	11	11	
SAUR1440 (SAR_RS07200)	1416848	1417933	16	10	
SAUR1441 (SAR_RS07205)	1417923	1419188	5	5	
SAUR1442 (SAR_RS07210)	1419428	1419829	12	12	
SAUR1443 (SAR_RS07215)	1420026	1420226	1	1	1
SAUR1444 (SAR_RS07220)	1420261	1420449	1	1	1
SAUR1445 (SAR_RS07225)	1420397	1420705	1	1	4
SAUR1446 (SAR_RS07230)	1420868	1421137	4	4	1
SAUR1447 (SAR_RS07235)	1421162	1421785	10	10	
SAUR1448 (SAR_RS07240)	1421817	1422953	11	11	
SAUR1449 (SAR_RS07245)	1423966	1425309	13	13	33
SAUR1451 (SAR_RS07255)	1427434	1428225	7	7	132
SAUR1452 (SAR_RS07260)	1428406	1428609	3	3	3
SAUR1453 (SAR_RS07265)	1428638	1429447	9	402	
SAUR1454 (SAR_RS07270)	1430027	1431295	11	67	
SAUR1455 (SAR_RS07275)	1431309	1434107	15	199	
SAUR1456 (SAR_RS07280)	1434391	1435746	9	9	
SAUR1457 (SAR_RS07285)	1435743	1436402	6	6	
SAUR1458 (SAR_RS07290)	1436664	1436795	3	3	
SAUR1459 (SAR_RS07295)	1437931	1438545	9	9	4
SAUR1460 (SAR_RS07300)	1438562	1439632	9	9	100
SAUR1461 (SAR_RS07305)	1439644	1440153	8	8	21
SAUR1462 (SAR_RS07310)	1440553	1442043	12	12	
SAUR1463 (SAR_RS07315)	1441982	1442137	2	2	
SAUR1465 (SAR_RS07325)	1442235	1442456	5	5	10
SAUR1466 (SAR_RS07330)	1442456	1442956	7	7	13

Locus	Start position in RGB-095930	End position in RGB-095930	Allele in NCTC8325, CP000253.1	Allele in RGB-095930	Allele in ST140_ERR1764920
SAUR1467 (SAR_RS07335)	1442968	1443396	8	8	56
SAUR1468 (SAR_RS07340)	1443389	1443922	7	7	
SAUR1469 (SAR_RS07345)	1444008	1444847	8	8	4
SAUR1470 (SAR_RS07350)	1444862	1445341	9	9	4
SAUR1471 (SAR_RS07355)	1445541	1446497	10	10	4
SAUR1472 (SAR_RS07360)	1446921	1447358	6	6	
SAUR1473 (SAR_RS07365)	1447374	1448498	11	11	
SAUR1474 (SAR_RS07370)	1448536	1448787	6	6	
SAUR1475 (SAR_RS07375)	1448799	1448993	2	2	2
SAUR1476 (SAR_RS07380)	1449238	1449942	11	11	91
SAUR1477 (SAR_RS07385)					
SAUR1478 (SAR_RS07390)	1450483	1450884	38	38	40
SAUR1480 (SAR_RS07400)	1480340	1481731	21	15	
SAUR1481 (SAR_RS07405)	1481888	1483210	10	10	
SAUR1482 (SAR_RS07410)	1483241	1484281	11	11	
SAUR1483 (SAR_RS07415)	1484376	1485494	18	10	
SAUR1484 (SAR_RS07420)	1485969	1486847	11	311	
SAUR1486 (SAR_RS07430)					
SAUR1487 (SAR_RS07435)	1492157	1492489	7	7	1
SAUR1488 (SAR_RS07440)	1492573	1493718	27	15	7
SAUR1489 (SAR_RS07445)	1494369	1494713	4	4	1
SAUR1490 (SAR_RS07450)	1494727	1495290	1	1	1
SAUR1491 (SAR_RS07455)	1495283	1495633	10	10	4
SAUR1492 (SAR_RS07460)					10
SAUR1493 (SAR_RS07465)	1496173	1496799	7	7	
SAUR1494 (SAR_RS07470)					
SAUR1495 (SAR_RS07475)	1499545	1499886	8	8	4
SAUR1496 (SAR_RS07480)	1499891	1500550	2	2	2
SAUR1497 (SAR_RS07485)	1500540	1501226	11	11	
SAUR1498 (SAR_RS07490)	1502296	1503588	10	10	
SAUR1499 (SAR_RS07495)	1503910	1506603	31	28	
SAUR1500 (SAR_RS07500)	1506627	1507598	23	12	
SAUR1501 (SAR_RS07505)	1507585	1508787	10	10	
SAUR1502 (SAR_RS07510)	1508792	1509934	9	9	
SAUR1503 (SAR_RS07515)	1510175	1510492	9	9	8
SAUR1504 (SAR_RS07520)	1510829	1511509	8	8	3
SAUR1505 (SAR_RS07525)	1511581	1512168	57	57	143
SAUR1506 (SAR_RS07530)	1512158	1512733	15	11	127
SAUR1507 (SAR_RS07535)	1512747	1513991	12	12	
SAUR1508 (SAR_RS07540)	1513998	1515296	67	67	
SAUR1509 (SAR_RS07545)	1515306	1516370	287	200	4
SAUR1510 (SAR_RS07550)	1516396	1517562	169	169	
SAUR1511 (SAR_RS07555)	1518357	1518806	9	9	98
SAUR1512 (SAR_RS07560)	1518898	1519857	8	8	163
SAUR1513 (SAR_RS07565)	1519859	1520584	10	10	127
SAUR1514 (SAR_RS07570)	1520587	1521159	7	7	3
SAUR1515 (SAR_RS07575)	1521086	1521400	8	8	
SAUR1516 (SAR_RS07580)	1521590	1521862	1	1	1
SAUR1517 (SAR_RS07585)	1522033	1523031	11	11	
SAUR1518 (SAR_RS07590)	1523048	1524358	8	8	
SAUR1519 (SAR_RS07595)	1524580	1525755	10	10	314
SAUR1520 (SAR_RS07600)	1526012	1526203	5	5	5
SAUR1523 (SAR_RS07615)	1526467	1527126	8	15	4
SAUR1524 (SAR_RS07620)	1527203	1528171	14	14	
SAUR1525 (SAR_RS07625)	1528286	1529272	8	8	
SAUR1526 (SAR_RS07630)	1529433	1529519	10	10	

Locus	Start position in RGB-095930	End position in RGB-095930	Allele in NCTC8325, CP000253.1	Allele in RGB-095930	Allele in ST140_ERR1764920
SAUR1528 (SAR_RS07640)	1531287	1532666	10	10	
SAUR1529 (SAR_RS07645)	1532656	1533609	9	367	4
SAUR1530 (SAR_RS07650)	1533717	1533965	2	2	2
SAUR1531 (SAR_RS07655)	1534071	1534616	5	5	
SAUR1604 (SAR_RS08020)					
SAUR1605 (SAR_RS08025)	1589875	1591626	12	12	
SAUR1606 (SAR_RS08030)	1591607	1592332	4	4	2
SAUR1607 (SAR_RS08035)	1592465	1593202	7	7	130
SAUR1608 (SAR_RS08040)	1593195	1593737	71	71	49
SAUR1609 (SAR_RS08045)	1593730	1594461	6	9	
SAUR1610 (SAR_RS08050)	1594553	1595059	9	9	135
SAUR1611 (SAR_RS08055)	1595137	1596024	10	10	4
SAUR1612 (SAR_RS08060)	1596073	1596522	5	5	1
SAUR1613 (SAR_RS08065)	1596627	1597169	5	258	4
SAUR1614 (SAR_RS08070)	1597251	1598159	10	10	
SAUR1615 (SAR_RS08075)	1598375	1598623	16	16	4
SAUR1617 (SAR_RS08085)	1599536	1600351	17	11	
SAUR1618 (SAR_RS08090)	1600456	1601376	10	10	
SAUR1619 (SAR_RS08095)	1601689	1603173	10	10	
SAUR1621 (SAR_RS08105)	1604352	1606001	10	10	
SAUR1622 (SAR_RS08110)	1606017	1607036	32	32	
SAUR1623 (SAR_RS08115)	1607037	1607264	15	7	
SAUR1624 (SAR_RS08120)	1607230	1607601	9	9	
SAUR1627 (SAR_RS08135)	1607820	1609226	10	89	
SAUR1628 (SAR_RS08140)	1609294	1610427	52	11	
SAUR1629 (SAR_RS08145)	1610690	1610869	4	4	48
SAUR1630 (SAR_RS08150)	1610996	1611976	10	10	
SAUR1631 (SAR_RS08155)	1611990	1612427	8	8	8
SAUR1632 (SAR_RS08160)	1612778	1614052	18	84	
SAUR1634 (SAR_RS08170)	1615048	1616040	12	12	
SAUR1635 (SAR_RS08175)	1616056	1617477	13	486	
SAUR1636 (SAR_RS08180)	1617628	1619307	23	12	
SAUR1637 (SAR_RS08185)	1619323	1619775	2	2	9
SAUR1638 (SAR_RS08190)	1620207	1621088	11	11	
SAUR1639 (SAR_RS08195)	1621066	1621296	3	3	2
SAUR1640 (SAR_RS08200)	1621289	1622626	21	12	
SAUR1641 (SAR_RS08205)	1622643	1623032	2	2	4
SAUR1642 (SAR_RS08210)	1623092	1623454	1	1	1
SAUR1643 (SAR_RS08215)	1623469	1624824	14	14	
SAUR1644 (SAR_RS08220)	1624824	1625288	6	6	4
SAUR1645 (SAR_RS08225)	1625762	1626319	6	6	4
SAUR1646 (SAR_RS08230)	1626345	1627406	8	8	4
SAUR1647 (SAR_RS08235)	1627511	1628092	2	2	4
SAUR1648 (SAR_RS08240)	1628106	1628324	5	5	3
SAUR1649 (SAR_RS08245)	1628388	1629218	10	12	
SAUR1650 (SAR_RS08250)	1629376	1629762	2	2	1
SAUR1651 (SAR_RS08255)	1630127	1631599	14	20	4
SAUR1652 (SAR_RS08260)	1631592	1632938	12	12	4
SAUR1653 (SAR_RS08265)			8		4
SAUR1654 (SAR_RS08270)	1634208	1634732	9	9	4
SAUR1655 (SAR_RS08275)	1634722	1634865	1	1	1
SAUR1656 (SAR_RS08280)	1634965	1635339	25	25	4
SAUR1657 (SAR_RS08285)	1635380	1635679	6	6	1
SAUR1659 (SAR_RS08295)	1636090	1636401	5	5	77
SAUR1660 (SAR_RS08300)	1636415	1637485		12	
SAUR1661 (SAR_RS08305)	1637457	1638431	14	14	

Locus	Start position in RGB-095930	End position in RGB-095930	Allele in NCTC8325, CP000253.1	Allele in RGB-095930	Allele in ST140_ERR1764920
SAUR1662 (SAR_RS08310)	1638483	1639106	11	11	4
SAUR1663 (SAR_RS08315)	1639103	1639432	9	9	
SAUR1664 (SAR_RS08320)	1639432	1640418	12	12	
SAUR1665 (SAR_RS08325)	1640415	1640618	1	1	2
SAUR1666 (SAR_RS08330)	1640599	1642062		499	
SAUR1667 (SAR_RS08335)	1642074	1642667	7	7	
SAUR1668 (SAR_RS08340)	1642822	1642971	1	1	1
SAUR1669 (SAR_RS08345)			17		
SAUR1670 (SAR_RS08350)	1645280	1645879	5	5	3
SAUR1671 (SAR_RS08355)	1646155	1646565	9	9	
SAUR1672 (SAR_RS08360)	1646552	1647415	7	11	4
SAUR1673 (SAR_RS08365)	1647457	1648242	8	8	
SAUR1674 (SAR_RS08370)	1648368	1649258	9	9	
SAUR1675 (SAR_RS08375)	1649268	1650614	14	14	
SAUR1676 (SAR_RS08380)	1650728	1651828	9	9	4
SAUR1677 (SAR_RS08385)	1651831	1652508	10	10	4
SAUR1678 (SAR_RS08390)	1652639	1653745	7	7	
SAUR1679 (SAR_RS08395)	1653969	1655768	7	7	
SAUR1680 (SAR_RS08400)	1655829	1656647	9	9	88
SAUR1681 (SAR_RS08405)	1656658	1657149	7	7	2
SAUR1682 (SAR_RS08410)	1657616	1659007	9	9	4
SAUR1683 (SAR_RS08415)	1659157	1659909	160	11	5
SAUR1684 (SAR_RS08420)	1659931	1660830	19	11	4
SAUR1685 (SAR_RS08425)	1660831	1661235	3	3	2
SAUR1686 (SAR_RS08430)	1661246	1661590	6	6	1
SAUR1687 (SAR_RS08435)	1661593	1662060	9	9	10
SAUR1688 (SAR_RS08440)	1662061	1663008	10	10	
SAUR1689 (SAR_RS08445)	1663312	1664010	6	6	4
SAUR1690 (SAR_RS08450)	1664027	1665016	8	8	4
SAUR1691 (SAR_RS08455)	1665034	1665717	9	15	1
SAUR1692 (SAR_RS08460)	1665961	1666137	1	1	1
SAUR1693 (SAR_RS08465)	1666430	1667776	12	12	
SAUR1694 (SAR_RS08470)	1667783	1668535	11	11	
SAUR1695 (SAR_RS08475)	1668537	1669475	9	9	
SAUR1696 (SAR_RS08480)	1669479	1670618	10	10	
SAUR1697 (SAR_RS08485)	1670754	1672586	16	16	4
SAUR1698 (SAR_RS08490)	1672655	1673281	5	5	
SAUR1699 (SAR_RS08495)	1673313	1674290	11	11	4
SAUR1700 (SAR_RS08500)	1674391	1675515	233	11	
SAUR1701 (SAR_RS08505)	1676078	1677901	11	11	
SAUR1702 (SAR_RS08510)	1678247	1678498	1	1	1
SAUR1703 (SAR_RS08515)	1678543	1679517	12	12	
SAUR1704 (SAR_RS08520)	1679574	1681775	23	14	
SAUR1705 (SAR_RS08525)	1681780	1682241	2	2	4
SAUR1706 (SAR_RS08530)	1682333	1683010	10	10	
SAUR1707 (SAR_RS08535)	1683059	1683775	9	9	
SAUR1708 (SAR_RS08540)	1683778	1684131	8	8	26
SAUR1709 (SAR_RS08545)	1684132	1684716	12	12	
SAUR1710 (SAR_RS08550)	1684706	1685275	7	7	
SAUR1711 (SAR_RS08555)	1685278	1685568	9	9	1
SAUR1712 (SAR_RS08560)	1685572	1686378	13	344	92
SAUR1713 (SAR_RS08565)	1686392	1687492	15	15	
SAUR1714 (SAR_RS08570)	1687493	1688020	6	6	2
SAUR1715 (SAR_RS08575)	1688040	1688726	10	56	4
SAUR1716 (SAR_RS08580)	1688772	1688894	3	3	
SAUR1717 (SAR_RS08585)	1689040	1689309	2	2	

Locus	Start position in RGB-095930	End position in RGB-095930	Allele in NCTC8325, CP000253.1	Allele in RGB-095930	Allele in ST140_ERR1764920
SAUR1718 (SAR_RS08590)	1689511	1689699	6	6	1
SAUR1719 (SAR_RS08595)					
SAUR1722 (SAR_RS08610)	1693518	1694747	6	6	
SAUR1723 (SAR_RS08615)	1694760	1695512	9	9	4
SAUR1724 (SAR_RS08620)	1695512	1696873	15	15	4
SAUR1725 (SAR_RS08625)	1696887	1697336	9	9	4
SAUR1726 (SAR_RS08630)	1697338	1698348	11	11	
SAUR1727 (SAR_RS08635)	1698338	1699072	9	333	
SAUR1728 (SAR_RS08640)	1699398	1699874	2	2	4
SAUR1729 (SAR_RS08645)	1699902	1700525	6	6	3
SAUR1730 (SAR_RS08650)	1700525	1701793	10	10	4
SAUR1731 (SAR_RS08655)	1701805	1702728	9	9	4
SAUR1732 (SAR_RS08660)	1702731	1703369	9	9	4
SAUR1733 (SAR_RS08665)	1703654	1703962	1	1	2
SAUR1734 (SAR_RS08670)	1703977	1704405	1	1	
SAUR1735 (SAR_RS08675)	1704409	1704669	4	4	1
SAUR1736 (SAR_RS08680)	1704732	1707362	14	42	4
SAUR1737 (SAR_RS08685)	1707705	1710182	15	757	
SAUR1738 (SAR_RS08690)	1710184	1710852	13	13	4
SAUR1739 (SAR_RS08695)	1711314	1712432		8	
SAUR1740 (SAR_RS08700)	1712433	1713575	10	10	4
SAUR1741 (SAR_RS08705)	1713887	1714900	251	60	35
SAUR1742 (SAR_RS08710)	1715137	1715283	5	5	
SAUR1744 (SAR_RS08720)	1715605	1716027		1	1
SAUR1745 (SAR_RS08725)	1716112	1717386	12	12	
SAUR1746 (SAR_RS08730)	1717547	1718320	7	272	84
SAUR1747 (SAR_RS08735)	1718320	1718448	19	19	
SAUR1748 (SAR_RS08740)	1718781	1720547	23	480	
SAUR1749 (SAR_RS08745)	1720563	1721825	9	9	
SAUR1750 (SAR_RS08750)	1722286	1723098	10	10	4
SAUR1751 (SAR_RS08755)	1723158	1723610	5	5	4
SAUR1752 (SAR_RS08760)	1723622	1725811	12	12	4
SAUR1753 (SAR_RS08765)	1726239	1726757	6	6	39
SAUR1754 (SAR_RS08770)	1726779	1729052	12	12	396
SAUR1755 (SAR_RS08775)	1729255	1731534	13	21	221
SAUR1756 (SAR_RS08780)	1731809	1732069	3	3	46
SAUR1757 (SAR_RS08785)	1732088	1733227		7	20
SAUR1758 (SAR_RS08790)	1733250	1734275	12	12	215
SAUR1759 (SAR_RS08795)	1734277	1735281	9	9	
SAUR1760 (SAR_RS08800)	1735313	1735915	9	9	
SAUR1761 (SAR_RS08805)	1735929	1736384	7	7	4
SAUR1762 (SAR_RS08810)	1736397	1737689	11	11	
SAUR1763 (SAR_RS08815)	1738044	1738328	1	1	1
SAUR1764 (SAR_RS08820)	1738340	1738660	4	4	1
SAUR1765 (SAR_RS08825)	1738666	1738974	1	1	1
SAUR1766 (SAR_RS08830)	1739141	1739671	11	11	
SAUR1778 (SAR_RS08890)	1749192	1749899	8	319	
SAUR1779 (SAR_RS08895)	1750001	1750111	4	4	
SAUR1780 (SAR_RS08900)	1750169	1751440	7	7	
SAUR1781 (SAR_RS08905)	1751453	1754083	14	14	
SAUR1782 (SAR_RS08910)	1754486	1755046	8	8	21
SAUR1783 (SAR_RS08915)	1755269	1756342	8	8	207
SAUR1784 (SAR_RS08920)					
SAUR1785 (SAR_RS08925)	1756538	1757824	8	8	310
SAUR1786 (SAR_RS08930)	1757872	1758846	10	10	169
SAUR1787 (SAR_RS08935)	1758849	1759517	10	10	121

Locus	Start position in RGB-095930	End position in RGB-095930	Allele in NCTC8325, CP000253.1	Allele in RGB-095930	Allele in ST140_ERR1764920
SAUR1788 (SAR_RS08940)	1759539	1760465	11	11	4
SAUR1789 (SAR_RS08945)	1760507	1761322	10	285	4
SAUR1790 (SAR_RS08950)	1761344	1762690	146	11	4
SAUR1791 (SAR_RS08955)	1762907	1763497	8	8	4
SAUR1792 (SAR_RS08960)	1763651	1764913	11	11	198
SAUR1793 (SAR_RS08965)	1765064	1766365	11	11	4
SAUR1794 (SAR_RS08970)	1766413	1766523	4	1	1
SAUR1795 (SAR_RS08975)	1766528	1767457	9	9	166
SAUR1796 (SAR_RS08980)	1767476	1768084	8	8	
SAUR1797 (SAR_RS08985)	1768161	1768262	1	1	1
SAUR1798 (SAR_RS08990)	1768226	1768582	5	5	3
SAUR1799 (SAR_RS08995)	1768629	1768829	2	2	1
SAUR1800 (SAR_RS09000)	1768858	1769385		7	4
SAUR1801 (SAR_RS09005)	1769614	1771107	165	165	
SAUR1802 (SAR_RS09010)	1771533	1773470	115	14	
SAUR1803 (SAR_RS09015)					
SAUR1804 (SAR_RS09020)	1773883	1774803	6	6	
SAUR1805 (SAR_RS09025)	1774803	1776203	14	14	
SAUR1806 (SAR_RS09030)	1776204	1776674	5	5	1
SAUR1807 (SAR_RS09035)	1776884	1777909	10	10	170
SAUR1808 (SAR_RS09040)	1778079	1778702	135	7	80
SAUR1809 (SAR_RS09045)	1778718	1779590	8	8	
SAUR1810 (SAR_RS09050)	1779606	1782236	19	802	
SAUR1811 (SAR_RS09055)	1782530	1784017	14	14	
SAUR1812 (SAR_RS09060)	1784516	1786180	9	9	
SAUR1813 (SAR_RS09065)					20
SAUR1814 (SAR_RS09070)	1786833	1787051	5	5	14
SAUR1815 (SAR_RS09075)	1787271	1788539	7	7	
SAUR1816 (SAR_RS09080)	1788588	1789709		8	
SAUR1817 (SAR_RS09085)	1790057	1791418	115	115	
SAUR1818 (SAR_RS09090)	1793743	1795500	9	9	4
SAUR1819 (SAR_RS09095)	1795522	1796490	12	12	4
SAUR1820 (SAR_RS09100)	1796759	1797703	8	8	
SAUR1821 (SAR_RS09105)	1797703	1798560	8	8	4
SAUR1822 (SAR_RS09110)	1798755	1799984	20	12	
SAUR1823 (SAR_RS09115)	1800434	1803631	76	69	
SAUR1824 (SAR_RS09120)	1803652	1804593	8	8	130
SAUR1825 (SAR_RS09125)	1804854	1806152	8	8	4
SAUR1826 (SAR_RS09130)	1806357	1806770	6	1	3
SAUR1827 (SAR_RS09135)	1807075	1807764	8	8	
SAUR1828 (SAR_RS09140)	1807939	1808994	10	10	4
SAUR1829 (SAR_RS09145)					
SAUR1830 (SAR_RS09150)	1809310	1810428	11	11	249
SAUR1831 (SAR_RS09155)	1810569	1811069	6	6	11
SAUR1832 (SAR_RS09160)	1811315	1812517	7	7	
SAUR1833 (SAR_RS09165)	1812605	1813552	8	8	
SAUR1834 (SAR_RS09170)	1813675	1814169	9	9	4
SAUR1835 (SAR_RS09175)	1814267	1815037	30	30	4
SAUR1836 (SAR_RS09180)	1815081	1816304	18	18	377
SAUR1837 (SAR_RS09185)	1816304	1817470	131	35	
SAUR1838 (SAR_RS09190)	1817607	1817741	3	3	
SAUR1839 (SAR_RS09195)	1817815	1819509		9	
SAUR1840 (SAR_RS09200)	1819646	1820110	134	9	92
SAUR1841 (SAR_RS09205)	1820354	1820956	4	4	4
SAUR1842 (SAR_RS09210)	1821029	1821283	3	3	
SAUR1843 (SAR_RS09215)	1821172	1821915	11	11	190

Locus	Start position in RGB-095930	End position in RGB-095930	Allele in NCTC8325, CP000253.1	Allele in RGB-095930	Allele in ST140_ERR1764920
SAUR1844 (SAR_RS09220)	1821995	1822441	7	175	4
SAUR1846 (SAR_RS09230)	1823702	1825306	13	614	
SAUR1847 (SAR_RS09235)	1825400	1825495	4	4	30
SAUR1848 (SAR_RS09240)	1825456	1826586	10	10	4
SAUR1849 (SAR_RS09245)	1826693	1828159	25	13	
SAUR1850 (SAR_RS09250)	1828287	1828904	10	10	
SAUR1851 (SAR_RS09255)	1829076	1830350	6	6	4
SAUR1852 (SAR_RS09260)	1830444	1831706	8	8	
SAUR1853 (SAR_RS09265)	1832088	1832993	11	11	
SAUR1855 (SAR_RS09275)			145		
SAUR1857 (SAR_RS09285)	1836217	1837884	21	10	
SAUR1859 (SAR_RS09295)	1840199	1840831	11	83	4
SAUR1860 (SAR_RS09300)	1840856	1842025	10	440	
SAUR1861 (SAR_RS09305)	1842123	1843112	10	10	4
SAUR1862 (SAR_RS09310)	1843185	1843298	3	3	1
SAUR1863 (SAR_RS09315)	1843652	1844743	17	9	3
SAUR1866 (SAR_RS09330)	1846875	1847366	5	5	4
SAUR1867 (SAR_RS09335)	1847440	1848753	10	10	
SAUR1868 (SAR_RS09340)	1848777	1852601	20	20	
SAUR1869 (SAR_RS09345)					
SAUR1870 (SAR_RS09350)	1853247	1854104	6	6	4
SAUR1871 (SAR_RS09355)	1854204	1854515	6	6	1
SAUR1872 (SAR_RS09360)	1854580	1855656	14	20	34
SAUR1873 (SAR_RS09365)	1855740	1856054	1	1	
SAUR1874 (SAR_RS09370)			11		4
SAUR1881 (SAR_RS09405)	1857491	1858135	9	16	4
SAUR1882 (SAR_RS09410)	1858150	1858941	6	6	4
SAUR1883 (SAR_RS09415)	1859491	1860339	11	11	
SAUR1884 (SAR_RS09420)	1860343	1861752	8	8	
SAUR1885 (SAR_RS09425)			7		1
SAUR1886 (SAR_RS09430)	1862881	1863576	11	11	
SAUR1887 (SAR_RS09435)	1863573	1865234	5	5	
SAUR1888 (SAR_RS09440)	1865641	1866909	5	5	50
SAUR1889 (SAR_RS09445)	1867026	1873586	1090	901	
SAUR1890 (SAR_RS09450)	1873912	1874223	39	39	
SAUR1891 (SAR_RS09455)	1874245	1876659	15	15	
SAUR1892 (SAR_RS09460)	1876950	1878131	13	13	
SAUR1893 (SAR_RS09465)	1878241	1879194	11	11	4
SAUR1894 (SAR_RS09470)	1879191	1879754	8	8	179
SAUR1895 (SAR_RS09475)	1879874	1880275	8	8	1
SAUR1896 (SAR_RS09480)	1883185	1884012	10	10	
SAUR1897 (SAR_RS09485)	1884015	1884131	3	3	
SAUR1898 (SAR_RS09490)	1884246	1885247	11	11	4
SAUR1899 (SAR_RS09495)	1885369	1885833	8	8	
SAUR1900 (SAR_RS09500)	1885846	1887027	15	15	
SAUR1901 (SAR_RS09505)	1887038	1887670	14	14	
SAUR1902 (SAR_RS09510)	1887677	1888720	10	10	4
SAUR1903 (SAR_RS09515)	1889201	1890703	27	12	
SAUR1904 (SAR_RS09520)	1891226	1891540	9	9	
SAUR1905 (SAR_RS09525)			74		
SAUR1906 (SAR_RS09530)	1893540	1894394	9	9	
SAUR1907 (SAR_RS09535)	1894670	1894894	5	5	
SAUR1908 (SAR_RS09540)	1895093	1895563	37	32	
SAUR1910 (SAR_RS09550)	1896106	1896549	64	64	4
SAUR1913 (SAR_RS09565)	1897920	1898633	7	7	142
SAUR1914 (SAR_RS09570)			7		115

Locus	Start position in RGB-095930	End position in RGB-095930	Allele in NCTC8325, CP000253.1	Allele in RGB-095930	Allele in ST140_ERR1764920
SAUR1915 (SAR_RS09575)	1900525	1900890	5	5	87
SAUR1916 (SAR_RS09580)	1900887	1901240	7	7	26
SAUR1917 (SAR_RS09585)			13		25
SAUR1918 (SAR_RS09590)	1905442	1906350	11	81	
SAUR1919 (SAR_RS09595)	1906475	1907668	8	8	
SAUR1920 (SAR_RS09600)	1908039	1909631	10	10	
SAUR1921 (SAR_RS09605)	1910627	1911397	144	144	
SAUR1922 (SAR_RS09610)	1911378	1911857	10	10	4
SAUR1923 (SAR_RS09615)	1911917	1912174	8	8	4
SAUR1924 (SAR_RS09620)	1912171	1913172	24	24	
SAUR1925 (SAR_RS09625)	1913177	1914655	6	6	
SAUR1926 (SAR_RS09630)	1914814	1915296	22	22	144
SAUR1927 (SAR_RS09635)	1915608	1916222	98	98	36
SAUR1928 (SAR_RS09640)	1916303	1917298	8	8	
SAUR1973 (SAR_RS09865)	1958009	1958563	11	6	4
SAUR1975 (SAR_RS09875)			81		
SAUR1976 (SAR_RS09880)	1960310	1961233	206	330	
SAUR1977 (SAR_RS09885)	1961291	1962328	9	88	
SAUR1978 (SAR_RS09890)					1
SAUR1979 (SAR_RS09895)	1962591	1963094	121	121	4
SAUR1980 (SAR_RS09900)	1963218	1964441	9	9	232
SAUR1981 (SAR_RS09905)	1964434	1965174	10	10	25
SAUR1982 (SAR_RS09910)	1965308	1965730	8	8	21
SAUR1983 (SAR_RS09915)	1965872	1966237	8	8	
SAUR1984 (SAR_RS09920)	1966970	1967527	6	6	6
SAUR1985 (SAR_RS09925)	1967732	1968694	9	9	
SAUR1986 (SAR_RS09930)	1968815	1969756	11	11	4
SAUR1987 (SAR_RS09935)	1969753	1972689	10	791	
SAUR1988 (SAR_RS09940)	1972679	1973875	11	11	104
SAUR1989 (SAR_RS09945)					
SAUR1990 (SAR_RS09950)	1974647	1974991	1	1	1
SAUR1991 (SAR_RS09955)	1975060	1976184	18	75	
SAUR1992 (SAR_RS09960)	1976363	1976827	7	1	1
SAUR1993 (SAR_RS09965)	1977183	1977806	7	7	20
SAUR1994 (SAR_RS09970)	1977828	1978940	10	10	167
SAUR1995 (SAR_RS09975)	1979103	1979924	11	11	4
SAUR1996 (SAR_RS09980)	1980387	1981772	8	8	
SAUR1997 (SAR_RS09985)	1981968	1982363	8	8	75
SAUR1999 (SAR_RS09995)	1983140	1983292	5	5	1
SAUR2000 (SAR_RS10000)	1983317	1983916	7	7	112
SAUR2001 (SAR_RS10005)	1984075	1984545	5	5	2
SAUR2002 (SAR_RS10010)			11		
SAUR2003 (SAR_RS10015)	1985828	1986550	9	9	160
SAUR2004 (SAR_RS10020)	1986543	1988000	13	13	
SAUR2005 (SAR_RS10025)	1988258	1989319	10	10	4
SAUR2036 (SAR_RS10180)	1999193	1999639	1	1	2
SAUR2037 (SAR_RS10185)	1999736	2000686	10	10	
SAUR2038 (SAR_RS10190)	2000692	2001147	9	193	17
SAUR2039 (SAR_RS10195)	2001228	2002517	10	10	
SAUR2040 (SAR_RS10200)	2002810	2003904	6	6	4
SAUR2042 (SAR_RS10210)	2004095	2005831	13	625	4
SAUR2043 (SAR_RS10215)	2006122	2006664	6	6	4
SAUR2044 (SAR_RS10220)	2006966	2008003	11	11	4
SAUR2045 (SAR_RS10225)	2008155	2009132	8	8	
SAUR2046 (SAR_RS10230)	2009236	2009466	8	8	
SAUR2047 (SAR_RS10235)	2009393	2010229	10	10	

Locus	Start position in RGB-095930	End position in RGB-095930	Allele in NCTC8325, CP000253.1	Allele in RGB-095930	Allele in ST140_ERR1764920
SAUR2048 (SAR_RS10240)	2010238	2011755	8	8	
SAUR2049 (SAR_RS10245)	2011770	2012084	8	8	4
SAUR2050 (SAR_RS10250)	2012062	2012880	8	8	172
SAUR2051 (SAR_RS10255)	2013141	2013950	9	9	
SAUR2052 (SAR_RS10260)	2014291	2014806	7	171	
SAUR2053 (SAR_RS10265)	2014937	2015098	1	4	1
SAUR2054 (SAR_RS10270)					
SAUR2055 (SAR_RS10275)	2056630	2057160	6	6	96
SAUR2056 (SAR_RS10280)	2057250	2058497	11	11	
SAUR2057 (SAR_RS10285)	2058509	2058715	3	3	1
SAUR2058 (SAR_RS10290)	2058856	2059320	6	6	3
SAUR2059 (SAR_RS10295)	2059327	2059602	4	4	4
SAUR2060 (SAR_RS10300)	2059880	2061097	11	11	
SAUR2061 (SAR_RS10305)	2061213	2061842	7	7	84
SAUR2062 (SAR_RS10310)	2061832	2062875	9	320	
SAUR2063 (SAR_RS10315)	2062872	2063573	10	10	21
SAUR2064 (SAR_RS10320)	2063588	2063974	5	5	12
SAUR2065 (SAR_RS10325)	2064191	2064949	7	7	
SAUR2067 (SAR_RS10335)	2066953	2067405	78	78	79
SAUR2068 (SAR_RS10340)	2067395	2067505	1	1	37
SAUR2069 (SAR_RS10345)	2067591	2068322	10	10	22
SAUR2070 (SAR_RS10350)	2068324	2069637		12	
SAUR2071 (SAR_RS10355)	2069929	2070429	6	6	75
SAUR2072 (SAR_RS10360)	2070511	2070603	1	1	1
SAUR2073 (SAR_RS10365)	2070818	2071372	9	9	102
SAUR2074 (SAR_RS10370)	2071440	2072510	9	72	22
SAUR2075 (SAR_RS10375)	2072757	2073287	8	8	4
SAUR2076 (SAR_RS10380)	2073457	2074818	11	11	
SAUR2077 (SAR_RS10385)	2074899	2075846	6	12	1
SAUR2078 (SAR_RS10390)	2076429	2076575	5	5	1
SAUR2079 (SAR_RS10395)	2076615	2078042	10	10	5
SAUR2080 (SAR_RS10400)	2078055	2079512	10	10	
SAUR2081 (SAR_RS10405)	2079514	2079816	2	2	1
SAUR2082 (SAR_RS10410)	2080183	2081721	198	16	
SAUR2083 (SAR_RS10415)	2081810	2083009	11	11	4
SAUR2084 (SAR_RS10420)	2083022	2085025	7	7	
SAUR2085 (SAR_RS10425)	2085029	2087221	14	14	
SAUR2086 (SAR_RS10430)	2087218	2087910	8	8	
SAUR2087 (SAR_RS10435)	2089588	2089890	2	2	2
SAUR2088 (SAR_RS10440)	2089999	2091294	8	8	
SAUR2089 (SAR_RS10445)	2092155	2093321	12	12	
SAUR2090 (SAR_RS10450)					3
SAUR2091 (SAR_RS10455)	2093970	2094143	5	5	45
SAUR2092 (SAR_RS10460)	2094124	2094726	6	6	111
SAUR2093 (SAR_RS10465)			9		
SAUR2094 (SAR_RS10470)	2095811	2097280	11	11	261
SAUR2095 (SAR_RS10475)	2097464	2098540	7	7	
SAUR2096 (SAR_RS10480)	2098560	2099354	24	14	
SAUR2098 (SAR_RS10490)	2099544	2101106	10	10	5
SAUR2099 (SAR_RS10495)	2101311	2102408	13	13	
SAUR2100 (SAR_RS10500)	2102780	2103340	3	3	
SAUR2101 (SAR_RS10505)	2103393	2104322	8	8	89
SAUR2102 (SAR_RS10510)	2104517	2104645	3	3	35
SAUR2103 (SAR_RS10515)	2104742	2106121	250	12	137
SAUR2104 (SAR_RS10520)	2106241	2107269	12	12	4
SAUR2105 (SAR_RS10525)	2107539	2107940	1	1	1

Locus	Start position in RGB-095930	End position in RGB-095930	Allele in NCTC8325, CP000253.1	Allele in RGB-095930	Allele in ST140_ERR1764920
SAUR2106 (SAR_RS10530)	2108513	2108686	2	2	1
SAUR2107 (SAR_RS10535)	2109137	2110177	13	13	216
SAUR2108 (SAR_RS10540)	2110234	2111076	7	7	124
SAUR2109 (SAR_RS10545)	2111073	2111630	11	8	
SAUR2110 (SAR_RS10550)	2111690	2112214	6	6	88
SAUR2111 (SAR_RS10555)	2112335	2112898	2	8	94
SAUR2112 (SAR_RS10560)	2112964	2113704	9	9	119
SAUR2113 (SAR_RS10565)	2113704	2114576	8	8	129
SAUR2114 (SAR_RS10570)	2114573	2115253	10	10	135
SAUR2115 (SAR_RS10575)	2115254	2116150	110	6	192
SAUR2116 (SAR_RS10580)			9		
SAUR2117 (SAR_RS10585)	2116791	2116967	26	26	1
SAUR2118 (SAR_RS10590)	2117210	2117308	2	2	1
SAUR2119 (SAR_RS10595)			10		
SAUR2122 (SAR_RS10610)	2121559	2121759	16	16	
SAUR2190 (SAR_RS10950)	2166556	2167380	17	325	
SAUR2191 (SAR_RS10955)	2167618	2168634	52	52	
SAUR2192 (SAR_RS10960)	2168656	2169711	52	324	20
SAUR2193 (SAR_RS10965)	2170146	2171369	232	45	
SAUR2194 (SAR_RS10970)	2173720	2175027	10	10	
SAUR2199 (SAR_RS10995)	2191025	2192641		137	157
SAUR2200 (SAR_RS11000)	2192717	2193001	9	9	1
SAUR2201 (SAR_RS11005)	2193176	2193919	103	103	4
SAUR2202 (SAR_RS11010)	2193944	2195125	75	389	
SAUR2204 (SAR_RS11020)	2196309	2197094	11	11	
SAUR2210 (SAR_RS11050)	2201186	2202145	12	12	184
SAUR2211 (SAR_RS11055)	2202142	2203626	13	13	375
SAUR2212 (SAR_RS11060)	2203775	2204725	8	8	150
SAUR2213 (SAR_RS11065)	2204908	2206158	23	23	4
SAUR2214 (SAR_RS11070)	2206366	2206590	6	6	4
SAUR2215 (SAR_RS11075)	2206650	2207729	10	10	
SAUR2216 (SAR_RS11080)	2208033	2208668	7	7	1
SAUR2217 (SAR_RS11085)			17		
SAUR2220 (SAR_RS11100)	2212923	2213948	11	11	138
SAUR2221 (SAR_RS11105)	2213941	2214405	5	5	1
SAUR2222 (SAR_RS11110)	2214378	2215040	8	8	19
SAUR2223 (SAR_RS11115)	2215021	2215515	9	9	4
SAUR2224 (SAR_RS11120)	2215520	2215675	6	6	72
SAUR2225 (SAR_RS11125)	2215993	2217681	21	11	
SAUR2226 (SAR_RS11130)	2217709	2219478	12	598	
SAUR2227 (SAR_RS11135)	2219478	2219732	6	6	1
SAUR2228 (SAR_RS11140)	2219869	2220873	9	9	196
SAUR2229 (SAR_RS11145)	2220903	2222432	13	13	
SAUR2230 (SAR_RS11150)	2222435	2223481	22	11	4
SAUR2231 (SAR_RS11155)	2223495	2224865	19	19	
SAUR2232 (SAR_RS11160)	2224866	2225438	112	112	
SAUR2233 (SAR_RS11165)	2225453	2226721	181	181	
SAUR2240 (SAR_RS11200)	2232963	2233418	8	8	
SAUR2241 (SAR_RS11205)	2233411	2235561	14	14	406
SAUR2242 (SAR_RS11210)	2235995	2236765	11	11	153
SAUR2243 (SAR_RS11215)	2236740	2237219	7	219	4
SAUR2244 (SAR_RS11220)	2237221	2237547	2	2	2
SAUR2245 (SAR_RS11225)	2237666	2238667		8	4
SAUR2246 (SAR_RS11230)	2238819	2239052	4	4	2
SAUR2247 (SAR_RS11235)	2239017	2239379	7	7	3
SAUR2248 (SAR_RS11240)	2239376	2239546	1	1	1

Locus	Start position in RGB-095930	End position in RGB-095930	Allele in NCTC8325, CP000253.1	Allele in RGB-095930	Allele in ST140_ERR1764920
SAUR2249 (SAR_RS11245)	2239631	2240779	7	7	
SAUR2250 (SAR_RS11250)	2240845	2241204	8	8	13
SAUR2251 (SAR_RS11255)	2241208	2241702	20	20	12
SAUR2252 (SAR_RS11260)					
SAUR2253 (SAR_RS11265)	2243262	2243741	9	9	
SAUR2254 (SAR_RS11270)	2243949	2244509	7	7	
SAUR2255 (SAR_RS11275)	2244529	2246556	13	13	
SAUR2256 (SAR_RS11280)	2246575	2248251	11	11	
SAUR2257 (SAR_RS11285)	2248275	2248385	3	3	1
SAUR2258 (SAR_RS11290)	2248523	2251180	21	30	
SAUR2259 (SAR_RS11295)	2251180	2251875	7	7	4
SAUR2260 (SAR_RS11300)	2252244	2253764	10	10	4
SAUR2261 (SAR_RS11305)	2254281	2255639	24	10	
SAUR2262 (SAR_RS11310)	2255654	2256724	6	6	4
SAUR2263 (SAR_RS11315)	2257042	2258244	8	8	1
SAUR2265 (SAR_RS11325)	2258508	2258645	1	1	1
SAUR2266 (SAR_RS11330)	2258773	2258982	5	5	4
SAUR2267 (SAR_RS11335)	2258994	2259287	7	7	4
SAUR2268 (SAR_RS11340)	2259453	2260937	9	9	
SAUR2269 (SAR_RS11345)	2260965	2261612	13	13	
SAUR2271 (SAR_RS11355)	2262425	2263297	9	9	4
SAUR2272 (SAR_RS11360)	2263383	2264024	7	63	4
SAUR2273 (SAR_RS11365)	2264026	2264817	10	19	4
SAUR2274 (SAR_RS11370)	2264801	2265631	10	10	4
SAUR2275 (SAR_RS11375)	2265624	2266313	214	167	128
SAUR2276 (SAR_RS11380)	2266413	2266538	3	3	2
SAUR2277 (SAR_RS11385)	2266699	2267394	10	10	
SAUR2278 (SAR_RS11390)	2267783	2268178	2	2	3
SAUR2279 (SAR_RS11395)	2268372	2268812	7	7	3
SAUR2280 (SAR_RS11400)	2268870	2269310	7	7	4
SAUR2281 (SAR_RS11405)	2269344	2270609		129	
SAUR2282 (SAR_RS11410)	2270720	2270953	2	2	2
SAUR2283 (SAR_RS11415)	2271085	2271177	4	4	3
SAUR2284 (SAR_RS11420)	2271515	2271919	4	4	4
SAUR2285 (SAR_RS11425)	2271939	2273351	11	11	206
SAUR2286 (SAR_RS11430)	2273373	2274239	5	5	1
SAUR2287 (SAR_RS11435)	2274270	2275778	11	11	4
SAUR2288 (SAR_RS11440)	2275800	2276339	9	9	4
SAUR2289 (SAR_RS11445)	2276339	2276860	2	2	2
SAUR2290 (SAR_RS11450)	2277058	2277270	1	1	1
SAUR2291 (SAR_RS11455)	2277313	2278041	7	7	3
SAUR2292 (SAR_RS11460)	2278062	2278415	6	6	4
SAUR2293 (SAR_RS11465)	2278582	2279709	80	93	263
SAUR2294 (SAR_RS11470)	2279733	2280362	9	9	4
SAUR2295 (SAR_RS11475)	2280390	2281628	2	500	
SAUR2296 (SAR_RS11480)	2281655	2282179	10	10	
SAUR2297 (SAR_RS11485)	2282286	2282705	7	7	4
SAUR2298 (SAR_RS11490)	2282702	2283748	7	7	
SAUR2299 (SAR_RS11495)	2283832	2284668	8	8	
SAUR2300 (SAR_RS11500)	2284655	2285731	6	6	
SAUR2301 (SAR_RS11505)	2285732	2286331	6	6	4
SAUR2302 (SAR_RS11510)	2286324	2286473	2	2	
SAUR2303 (SAR_RS11515)	2286678	2286932	1	1	1
SAUR2304 (SAR_RS11520)	2287050	2288366	17	8	
SAUR2305 (SAR_RS11525)	2288613	2290040	13	13	282
SAUR2306 (SAR_RS11530)	2290278	2290613	6	4	4

Locus	Start position in RGB-095930	End position in RGB-095930	Allele in NCTC8325, CP000253.1	Allele in RGB-095930	Allele in ST140_ERR1764920
SAUR2307 (SAR_RS11535)	2290702	2291961	10	6	6
SAUR2308 (SAR_RS11540)	2292427	2293287	8	299	299
SAUR2309 (SAR_RS11545)	2293505	2294026	7	3	3
SAUR2310 (SAR_RS11550)	2294135	2295745	23	456	
SAUR2311 (SAR_RS11555)	2296081	2296611	7	3	3
SAUR2312 (SAR_RS11560)	2296723	2297583	10	312	
SAUR2313 (SAR_RS11565)	2298008	2298811	10	5	5
SAUR2314 (SAR_RS11570)	2298941	2299612	1	232	232
SAUR2315 (SAR_RS11575)	2302038	2303231	10	459	459
SAUR2316 (SAR_RS11580)	2303231	2304415	11	478	478
SAUR2317 (SAR_RS11585)	2304724	2305194	7	67	67
SAUR2318 (SAR_RS11590)	2305346	2305696	5	122	122
SAUR2319 (SAR_RS11595)	2305710	2307011	15	494	494
SAUR2322 (SAR_RS11610)	2308267	2308977	8	4	4
SAUR2323 (SAR_RS11615)	2309098	2309541	7	3	3
SAUR2324 (SAR_RS11620)	2309741	2310154	10	4	4
SAUR2325 (SAR_RS11625)	2310612	2311985	11	4	4
SAUR2326 (SAR_RS11630)	2312657	2312887	4	3	3
SAUR2327 (SAR_RS11635)	2312918	2313856		28	28
SAUR2328 (SAR_RS11640)	2314290	2314955	10	5	5
SAUR2329 (SAR_RS11645)	2315107	2315427	5	3	3
SAUR2330 (SAR_RS11650)			9		308
SAUR2331 (SAR_RS11655)	2316675	2317766	11	364	364
SAUR2334 (SAR_RS11670)	2319864	2320721	153	276	276
SAUR2335 (SAR_RS11675)			79		
SAUR2336 (SAR_RS11680)	2321803	2323608	6	427	427
SAUR2337 (SAR_RS11685)	2324289	2325827	146	506	506
SAUR2338 (SAR_RS11690)	2325862	2327994		730	730
SAUR2339 (SAR_RS11695)	2328006	2328440	9	27	
SAUR2340 (SAR_RS11700)	2328440	2329546	19	397	397
SAUR2343 (SAR_RS11715)	2337535	2338890	13	5	5
SAUR2344 (SAR_RS11720)	2338917	2339849	8	309	309
SAUR2345 (SAR_RS11725)	2339851	2340660	9	235	5
SAUR2346 (SAR_RS11730)	2340849	2341757	9	4	4
SAUR2356 (SAR_RS11780)					
SAUR2357 (SAR_RS11785)	2347882	2348946		389	389
SAUR2358 (SAR_RS11790)	2349094	2350536	182	402	9
SAUR2359 (SAR_RS11795)	2350725	2351192	37	149	149
SAUR2361 (SAR_RS11805)			79		
SAUR2362 (SAR_RS11810)	2353538	2354725	10	226	226
SAUR2363 (SAR_RS11815)	2354769	2355284	13	4	4
SAUR2364 (SAR_RS11820)	2355461	2355721	64	4	4
SAUR2365 (SAR_RS11825)	2355853	2357223	12	383	383
SAUR2366 (SAR_RS11830)	2357459	2358427	11	4	4
SAUR2367 (SAR_RS11835)			6		
SAUR2368 (SAR_RS11840)	2359467	2360450	27	162	162
SAUR2369 (SAR_RS11845)	2360617	2360742	19	1	1
SAUR2370 (SAR_RS11850)	2360843	2361913	10	362	
SAUR2371 (SAR_RS11855)	2361920	2363677	15	585	585
SAUR2372 (SAR_RS11860)	2363664	2364857	13	404	404
SAUR2373 (SAR_RS11865)	2364959	2366935	137	704	704
SAUR2374 (SAR_RS11870)	2367096	2367605	7	15	15
SAUR2375 (SAR_RS11875)	2367668	2367907	5	4	4
SAUR2376 (SAR_RS11880)	2367920	2368456	6	1	1
SAUR2377 (SAR_RS11885)	2368631	2370193	13	275	275
SAUR2378 (SAR_RS11890)	2370501	2371508	94	221	221

Locus	Start position in RGB-095930	End position in RGB-095930	Allele in NCTC8325, CP000253.1	Allele in RGB-095930	Allele in ST140_ERR1764920
SAUR2379 (SAR_RS11895)	2371743	2372744	10	337	337
SAUR2380 (SAR_RS11900)	2373064	2373948	12	4	4
SAUR2381 (SAR_RS11905)	2374199	2375611	10	501	501
SAUR2382 (SAR_RS11910)	2375629	2377338	10	637	637
SAUR2383 (SAR_RS11915)	2377347	2377658	7	120	120
SAUR2384 (SAR_RS11920)	2377680	2378660	8	339	
SAUR2385 (SAR_RS11925)	2378664	2379596	13	451	
SAUR2386 (SAR_RS11930)	2379609	2380124	10	218	218
SAUR2387 (SAR_RS11935)	2380140	2380568	5	5	5
SAUR2388 (SAR_RS11940)			8		
SAUR2389 (SAR_RS11945)			8		
SAUR2394 (SAR_RS11970)	2383359	2384207	75	302	
SAUR2395 (SAR_RS11975)	2384277	2384693	11	23	23
SAUR2396 (SAR_RS11980)	2384936	2385103	2	4	4
SAUR2400 (SAR_RS12000)			34		
SAUR2401 (SAR_RS12005)			127		
SAUR2402 (SAR_RS12010)	2391306	2392970	344	592	592
SAUR2406 (SAR_RS12030)	2393927	2394319	2	1	1
SAUR2407 (SAR_RS12035)	2394339	2394776	2	2	2
SAUR2408 (SAR_RS12040)					
SAUR2409 (SAR_RS12045)	2395824	2396630	11	262	262
SAUR2410 (SAR_RS12050)	2396620	2397480	9	281	281
SAUR2411 (SAR_RS12055)	2397477	2398286	10	156	156
SAUR2414 (SAR_RS12070)	2398824	2399192	1	1	1
SAUR2415 (SAR_RS12075)	2399209	2400153	3	1	1
SAUR2416 (SAR_RS12080)	2400228	2400617	1	1	1
SAUR2417 (SAR_RS12085)	2400641	2401006	1	1	1
SAUR2418 (SAR_RS12090)	2401029	2401142	1	1	1
SAUR2419 (SAR_RS12095)	2401174	2401392	1	1	1
SAUR2420 (SAR_RS12100)	2401585	2402232	3	3	3
SAUR2421 (SAR_RS12105)	2402249	2403541	11	397	397
SAUR2422 (SAR_RS12110)	2403541	2403981	2	2	2
SAUR2423 (SAR_RS12115)	2403998	2404177	1	1	1
SAUR2424 (SAR_RS12120)	2404194	2404694	1	1	1
SAUR2425 (SAR_RS12125)	2404715	2405074	1	1	1
SAUR2426 (SAR_RS12130)	2405105	2405641	2	2	2
SAUR2427 (SAR_RS12135)	2405666	2406064	3	2	2
SAUR2428 (SAR_RS12140)	2406096	2406281	1	1	1
SAUR2429 (SAR_RS12145)	2406304	2406843	1	1	1
SAUR2430 (SAR_RS12150)	2406870	2407187	2	1	1
SAUR2431 (SAR_RS12155)	2407223	2407591	5	1	1
SAUR2432 (SAR_RS12160)	2407623	2407886	2	2	2
SAUR2433 (SAR_RS12165)	2407910	2408119	1	1	1
SAUR2434 (SAR_RS12170)	2408109	2408543	1	1	91
SAUR2435 (SAR_RS12175)	2408546	2409199	6	2	2
SAUR2436 (SAR_RS12180)	2409223	2409576	1	1	1
SAUR2437 (SAR_RS12185)	2409605	2409883	1	1	1
SAUR2438 (SAR_RS12190)	2409950	2410783	2	2	2
SAUR2439 (SAR_RS12195)	2410816	2411091	3	1	1
SAUR2440 (SAR_RS12200)	2411091	2411714	2	4	4
SAUR2441 (SAR_RS12205)	2411741	2412403	2	4	4
SAUR2442 (SAR_RS12210)	2412431	2412739	7	4	4
SAUR2443 (SAR_RS12215)	2413096	2413485	4	1	1
SAUR2444 (SAR_RS12220)	2413679	2415013	11	488	488
SAUR2445 (SAR_RS12225)	2415126	2417261	452	797	797
SAUR2446 (SAR_RS12230)	2417287	2417382	1	12	12

Locus	Start position in RGB-095930	End position in RGB-095930	Allele in NCTC8325, CP000253.1	Allele in RGB-095930	Allele in ST140_ERR1764920
SAUR2447 (SAR_RS12235)	2417448	2418335	11	330	330
SAUR2448 (SAR_RS12240)	2418516	2419379	8	22	22
SAUR2449 (SAR_RS12245)	2420092	2421000	8	22	22
SAUR2450 (SAR_RS12250)	2421133	2421297	5	1	1
SAUR2451 (SAR_RS12255)	2421542	2421859	4	114	
SAUR2452 (SAR_RS12260)	2422046	2425213	560	1014	1014
SAUR2453 (SAR_RS12265)	2425330	2426595	10	4	4
SAUR2454 (SAR_RS12270)	2426984	2427235	7	92	92
SAUR2455 (SAR_RS12275)	2427227	2427991	8	20	20
SAUR2456 (SAR_RS12280)			4		
SAUR2457 (SAR_RS12285)	2428586	2429797	2	5	5
SAUR2458 (SAR_RS12290)	2429899	2430249	6	1	1
SAUR2459 (SAR_RS12295)			5		
SAUR2460 (SAR_RS12300)					
SAUR2461 (SAR_RS12305)	2430885	2431907	7	20	20
SAUR2462 (SAR_RS12310)	2431927	2432526	8	22	22
SAUR2463 (SAR_RS12315)	2432533	2432766	6	12	12
SAUR2464 (SAR_RS12320)	2432772	2433218	68	4	4
SAUR2465 (SAR_RS12325)	2433232	2433717	7	4	4
SAUR2466 (SAR_RS12330)	2433714	2434973	24	454	279
SAUR2467 (SAR_RS12335)	2435036	2435530	9	148	148
SAUR2468 (SAR_RS12340)	2435532	2436038	8	95	95
SAUR2469 (SAR_RS12345)	2436068	2437072	12	312	312
SAUR2470 (SAR_RS12350)	2437240	2437845	7	4	4
SAUR2471 (SAR_RS12355)	2437846	2438517	8	4	4
SAUR2472 (SAR_RS12360)	2438531	2439313	10	244	4
SAUR2473 (SAR_RS12365)	2439603	2440400	10	3	3
SAUR2474 (SAR_RS12370)			6		
SAUR2475 (SAR_RS12375)	2441418	2441972	79	4	4
SAUR2476 (SAR_RS12380)			13		
SAUR2477 (SAR_RS12385)	2443434	2444342	11	4	4
SAUR2478 (SAR_RS12390)	2444555	2445709		262	262
SAUR2479 (SAR_RS12395)			138		
SAUR2480 (SAR_RS12400)	2447275	2447577	1	15	15
SAUR2481 (SAR_RS12405)	2447591	2448001	8	15	15
SAUR2482 (SAR_RS12410)	2447998	2449710	13	586	586
SAUR2483 (SAR_RS12415)	2449723	2450175	6	154	154
SAUR2484 (SAR_RS12420)	2450168	2450857	8	4	4
SAUR2485 (SAR_RS12425)	2450870	2451484	8	221	20
SAUR2486 (SAR_RS12430)	2451484	2452320	10	298	298
SAUR2487 (SAR_RS12435)	2452509	2452856	1	1	1
SAUR2488 (SAR_RS12440)					
SAUR2489 (SAR_RS12445)	2453284	2453610	8	4	4
SAUR2490 (SAR_RS12450)	2453674	2454417	15	4	4
SAUR2491 (SAR_RS12455)	2454443	2456398	12	673	673
SAUR2492 (SAR_RS12460)	2457008	2457817	13	4	4
SAUR2493 (SAR_RS12465)					
SAUR2494 (SAR_RS12470)	2458254	2459651	13	546	546
SAUR2495 (SAR_RS12475)	2459749	2460831	8	188	188
SAUR2496 (SAR_RS12480)	2461079	2461501	8	4	4
SAUR2497 (SAR_RS12485)	2461738	2462238	10	4	4
SAUR2498 (SAR_RS12490)					
SAUR2499 (SAR_RS12495)	2462601	2463554	8	323	323
SAUR2500 (SAR_RS12500)	2463646	2464770	20	4	
SAUR2502 (SAR_RS12510)	2465323	2466099	13	4	4
SAUR2503 (SAR_RS12515)	2466121	2466354	1	1	1

Locus	Start position in RGB-095930	End position in RGB-095930	Allele in NCTC8325, CP000253.1	Allele in RGB-095930	Allele in ST140_ERR1764920
SAUR2504 (SAR_RS12520)	2466649	2467122	79	5	5
SAUR2505 (SAR_RS12525)	2467122	2470076	425	259	259
SAUR2506 (SAR_RS12530)	2470682	2471629	8	190	190
SAUR2507 (SAR_RS12535)	2471762	2472559	10	163	163
SAUR2508 (SAR_RS12540)	2472943	2473635	8	220	
SAUR2509 (SAR_RS12545)	2473660	2474397	6	16	
SAUR2510 (SAR_RS12550)	2474446	2474775	6	4	4
SAUR2511 (SAR_RS12555)	2475221	2476093	6	217	217
SAUR2512 (SAR_RS12560)	2476472	2477851	10	205	205
SAUR2513 (SAR_RS12565)	2478129	2478485		95	95
SAUR2514 (SAR_RS12570)	2478482	2478667	1	1	1
SAUR2515 (SAR_RS12575)	2478763	2478951	2	2	2
SAUR2516 (SAR_RS12580)	2478965	2479600	7	247	247
SAUR2517 (SAR_RS12585)	2479675	2480592	11	23	23
SAUR2518 (SAR_RS12590)	2480860	2481390	3	1	1
SAUR2519 (SAR_RS12595)	2481456	2483060	25	470	
SAUR2520 (SAR_RS12600)	2483390	2484154	6	196	196
SAUR2521 (SAR_RS12605)	2484212	2484718	10	24	24
SAUR2523 (SAR_RS12615)			72		
SAUR2524 (SAR_RS12620)	2487482	2488363	9	333	333
SAUR2525 (SAR_RS12625)	2488472	2488660	7	4	4
SAUR2526 (SAR_RS12630)					
SAUR2527 (SAR_RS12635)	2489865	2491103	9	4	4
SAUR2528 (SAR_RS12640)	2491103	2492764	8	551	551
SAUR2529 (SAR_RS12645)	2492924	2493808	8	18	18
SAUR2531 (SAR_RS12655)	2494481	2495416	6	303	303
SAUR2532 (SAR_RS12660)	2495694	2496953	7	405	405
SAUR2533 (SAR_RS12665)	2497019	2497705	8	20	20
SAUR2534 (SAR_RS12670)	2497876	2498529	63	282	282
SAUR2535 (SAR_RS12675)	2498553	2498741	5	7	7
SAUR2536 (SAR_RS12680)	2498765	2499784	11	159	159
SAUR2537 (SAR_RS12685)	2499814	2500140	2	1	
SAUR2538 (SAR_RS12690)	2500358	2501590	8	442	442
SAUR2539 (SAR_RS12695)	2501583	2502482	9	24	24
SAUR2540 (SAR_RS12700)	2502713	2503360	6	105	105
SAUR2541 (SAR_RS12705)	2503759	2504367	9	4	4
SAUR2542 (SAR_RS12710)	2504704	2505912	35	376	
SAUR2543 (SAR_RS12715)	2505960	2507009	11	22	22
SAUR2544 (SAR_RS12720)	2507040	2507987	10	304	304
SAUR2545 (SAR_RS12725)	2508084	2508575	7	4	4
SAUR2546 (SAR_RS12730)			7		
SAUR2547 (SAR_RS12735)	2509063	2509983	9	293	293
SAUR2548 (SAR_RS12740)			134		16
SAUR2549 (SAR_RS12745)	2511076	2513007	240	151	
SAUR2550 (SAR_RS12750)	2513020	2513667	118	84	84
SAUR2551 (SAR_RS12755)	2513788	2514342	99	156	61
SAUR2553 (SAR_RS12765)	2516484	2517692		409	4
SAUR2554 (SAR_RS12770)	2517802	2517929	8	1	1
SAUR2555 (SAR_RS12775)	2517960	2519342	12	4	4
SAUR2556 (SAR_RS12780)	2519252	2519521	5	2	2
SAUR2557 (SAR_RS12785)	2519582	2520037		1	1
SAUR2558 (SAR_RS12790)	2520232	2521200	12	1	1
SAUR2559 (SAR_RS12795)	2521430	2522095	9	247	
SAUR2560 (SAR_RS12800)	2522095	2523150	10	392	26
SAUR2561 (SAR_RS12805)	2523286	2523960	9	19	19
SAUR2562 (SAR_RS12810)	2523953	2525326	11	443	443

Locus	Start position in RGB-095930	End position in RGB-095930	Allele in NCTC8325, CP000253.1	Allele in RGB-095930	Allele in ST140_ERR1764920
SAUR2563 (SAR_RS12815)	2525491	2525931	11	1	1
SAUR2564 (SAR_RS12820)	2525928	2526383	88	4	4
SAUR2568 (SAR_RS12840)	2526448	2527926	10	467	467
SAUR2569 (SAR_RS12845)	2528307	2529905	14	460	460
SAUR2570 (SAR_RS12850)	2530207	2531895	12	596	596
SAUR2571 (SAR_RS12855)	2532049	2532678	7	231	231
SAUR2572 (SAR_RS12860)	2532994	2533509	10	5	5
SAUR2573 (SAR_RS12865)	2533658	2534704	208	4	4
SAUR2574 (SAR_RS12870)			139		
SAUR2575 (SAR_RS12875)	2535707	2536741	8	327	327
SAUR2576 (SAR_RS12880)	2536752	2536868	5	52	52
SAUR2577 (SAR_RS12885)	2536973	2537404	1	1	1
SAUR2578 (SAR_RS12890)			5		
SAUR2579 (SAR_RS12895)	2537610	2538887	12	469	469
SAUR2580 (SAR_RS12900)	2539048	2539671	10	4	4
SAUR2581 (SAR_RS12905)	2539717	2540661	7	4	4
SAUR2582 (SAR_RS12910)	2540747	2542189		478	
SAUR2583 (SAR_RS12915)	2542372	2542755		14	14
SAUR2584 (SAR_RS12920)			23		737
SAUR2585 (SAR_RS12925)	2547751	2548173	9	136	136
SAUR2586 (SAR_RS12930)	2548451	2548807	80	10	10
SAUR2588 (SAR_RS12940)	2549188	2550465	9	169	169
SAUR2589 (SAR_RS12945)	2550647	2551012	8	4	4
SAUR2590 (SAR_RS12950)	2551409	2551855	6	290	1
SAUR2591 (SAR_RS12955)	2552063	2552500	8	153	153
SAUR2592 (SAR_RS12960)	2552608	2553777	12	383	383
SAUR2595 (SAR_RS12975)	2556229	2556882	6	162	162
SAUR2596 (SAR_RS12980)	2556905	2557939	10	251	
SAUR2597 (SAR_RS12985)	2557963	2558415	5	4	4
SAUR2598 (SAR_RS12990)	2558435	2559112	8	3	3
SAUR2599 (SAR_RS12995)	2559105	2559680	2	1	1
SAUR2600 (SAR_RS13000)			7		
SAUR2601 (SAR_RS13005)	2561222	2564911	27	906	906
SAUR2602 (SAR_RS13010)	2565218	2566195	60	28	28
SAUR2603 (SAR_RS13015)	2566186	2566500	7	17	17
SAUR2604 (SAR_RS13020)	2566504	2568909	32	565	565
SAUR2605 (SAR_RS13025)	2568955	2569707		54	
SAUR2606 (SAR_RS13030)	2569935	2570471	124	1	1
SAUR2607 (SAR_RS13035)	2570877	2571701	116	291	291
SAUR2609 (SAR_RS13045)	2572173	2572640	18	4	4
SAUR2610 (SAR_RS13050)	2572826	2574373	11	511	511
SAUR2613 (SAR_RS13065)			4		
SAUR2614 (SAR_RS13070)	2575455	2576054	9	176	176
SAUR2615 (SAR_RS13075)	2576073	2576435	1	9	9
SAUR2616 (SAR_RS13080)	2576690	2577940	209	411	411
SAUR2617 (SAR_RS13085)	2578037	2578768	3	174	174
SAUR2618 (SAR_RS13090)	2578765	2579484	7	116	116
SAUR2619 (SAR_RS13095)	2579465	2580244	9	242	242
SAUR2620 (SAR_RS13100)			10		
SAUR2621 (SAR_RS13105)	2581979	2582083	2	1	1
SAUR2622 (SAR_RS13110)	2582237	2582923	9	112	112
SAUR2623 (SAR_RS13115)	2583249	2584115	10	290	290
SAUR2624 (SAR_RS13120)			3		
SAUR2625 (SAR_RS13125)	2584675	2585988	6	336	336
SAUR2627 (SAR_RS13135)	2586523	2587452	10	4	4
SAUR2628 (SAR_RS13140)	2588020	2588967	10	332	332

Locus	Start position in RGB-095930	End position in RGB-095930	Allele in NCTC8325, CP000253.1	Allele in RGB-095930	Allele in ST140_ERR1764920
SAUR2629 (SAR_RS13145)	2588969	2589946		61	61
SAUR2630 (SAR_RS13150)	2589998	2590465	22	5	
SAUR2631 (SAR_RS13155)	2590476	2591168	9	241	241
SAUR2632 (SAR_RS13160)	2591179	2592294	11	4	4
SAUR2633 (SAR_RS13165)	2592272	2593279	9	4	4
SAUR2634 (SAR_RS13170)	2593281	2594639	10	416	416
SAUR2635 (SAR_RS13175)	2594617	2595303	8	154	154
SAUR2636 (SAR_RS13180)	2595358	2595525	4	46	46
SAUR2637 (SAR_RS13185)	2595525	2595668	6	1	1
SAUR2640 (SAR_RS13200)			7		
SAUR2642 (SAR_RS13210)			8		
SAUR2643 (SAR_RS13215)			8		
SAUR2644 (SAR_RS13220)	2602076	2602735	6	4	4
SAUR2645 (SAR_RS13225)	2602923	2604134	12	367	367
SAUR2646 (SAR_RS13230)	2604257	2604730	8	193	193
SAUR2647 (SAR_RS13235)	2604843	2605499	11	4	4
SAUR2648 (SAR_RS13240)	2605562	2606368	9	313	313
SAUR2649 (SAR_RS13245)	2606654	2608063	14	235	
SAUR2650 (SAR_RS13250)	2608201	2610279	11	518	518
SAUR2651 (SAR_RS13255)			3		
SAUR2652 (SAR_RS13260)	2610418	2612247	11	507	507
SAUR2654 (SAR_RS13270)	2612479	2613978	184	399	399
SAUR2655 (SAR_RS13275)	2614085	2614936	11	307	307
SAUR2657 (SAR_RS13285)	2615256	2616191	8	325	325
SAUR2658 (SAR_RS13290)	2616417	2617817	12	4	4
SAUR2659 (SAR_RS13295)	2618110	2618805	7	254	254
SAUR2660 (SAR_RS13300)	2618805	2619746	10	273	273
SAUR2661 (SAR_RS13305)	2619763	2620398	11	253	
SAUR2662 (SAR_RS13310)			11		
SAUR2667 (SAR_RS13335)	2625016	2626368	10	530	530
SAUR2668 (SAR_RS13340)			10		
SAUR2669 (SAR_RS13345)	2628013	2628789	123	283	4
SAUR2670 (SAR_RS13350)			132		
SAUR2671 (SAR_RS13355)	2629789	2630865	36	373	373
SAUR2672 (SAR_RS13360)	2631103	2631558	8	1	1
SAUR2673 (SAR_RS13365)	2631726	2633303	12	536	536
SAUR2674 (SAR_RS13370)	2633498	2634247	226	287	287
SAUR2675 (SAR_RS13375)	2634471	2635664	12	343	343
SAUR2676 (SAR_RS13380)	2635676	2636425	10	234	234
SAUR2677 (SAR_RS13385)	2636418	2637233	139	67	67
SAUR2678 (SAR_RS13390)	2637230	2638099	12	135	135
SAUR2679 (SAR_RS13395)	2638096	2639031	9	134	134
SAUR2680 (SAR_RS13400)	2639044	2640642	304	42	42
SAUR2681 (SAR_RS13405)	2640785	2642086	17	390	390
SAUR2682 (SAR_RS13410)	2642079	2642897	9	4	4
SAUR2683 (SAR_RS13415)	2642908	2643729	10	4	4
SAUR2685 (SAR_RS13425)					
SAUR2686 (SAR_RS13430)	2646349	2647887	8	495	495
SAUR2687 (SAR_RS13435)	2647999	2648421	10	4	4
SAUR2694 (SAR_RS13470)			176		
SAUR2695 (SAR_RS13475)	2653476	2653757	29	36	36
SAUR2696 (SAR_RS13480)	2653693	2653878	3	7	7
SAUR2697 (SAR_RS13485)	2654017	2654208	1	15	
SAUR2704 (SAR_RS13520)			32		
SAUR2705 (SAR_RS13525)	2661321	2661587	3	51	51
SAUR2706 (SAR_RS13530)			14		

Locus	Start position in RGB-095930	End position in RGB-095930	Allele in NCTC8325, CP000253.1	Allele in RGB-095930	Allele in ST140_ERR1764920
SAUR2707 (SAR_RS13535)	2662163	2663029	7	125	125
SAUR2719 (SAR_RS13595)	2681159	2681770	29	5	5
SAUR2723 (SAR_RS13615)			9		
SAUR2724 (SAR_RS13620)	2684622	2685392	7	252	252
SAUR2725 (SAR_RS13625)	2685898	2687862	225	493	493
SAUR2726 (SAR_RS13630)	2688206	2689285	12	230	230
SAUR2727 (SAR_RS13635)	2689390	2689983	25	4	4
SAUR2728 (SAR_RS13640)	2690005	2690973	15	264	264
SAUR2729 (SAR_RS13645)	2691051	2691485	10	4	4
SAUR2730 (SAR_RS13650)	2691712	2691996	21	3	3
SAUR2731 (SAR_RS13655)	2692171	2692977	9	332	332
SAUR2732 (SAR_RS13660)	2693097	2693768	11	230	230
SAUR2733 (SAR_RS13665)	2694038	2695030	10	347	347
SAUR2736 (SAR_RS13680)			10		
SAUR2737 (SAR_RS13685)	2697932	2698552	8	110	110
SAUR2738 (SAR_RS13690)	2698746	2699237	6	137	137
SAUR2739 (SAR_RS13695)	2699381	2699551	1	2	2
SAUR2740 (SAR_RS13700)			17		
SAUR2741 (SAR_RS13705)	2699729	2700628	11	240	19
SAUR2742 (SAR_RS13710)	2700642	2701322	8	2	2
SAUR2743 (SAR_RS13715)	2701325	2702368	11	4	4
SAUR2744 (SAR_RS13720)	2702916	2703119	5	3	3
SAUR2745 (SAR_RS13725)	2703275	2704237	10	143	143
SAUR2746 (SAR_RS13730)	2704319	2705287	12	347	347
SAUR2747 (SAR_RS13735)					
SAUR2748 (SAR_RS13740)	2705805	2706215	11	4	4
SAUR2749 (SAR_RS13745)			9		
SAUR2750 (SAR_RS13750)	2710029	2711768	25	478	478
SAUR2751 (SAR_RS13755)	2711808	2712497	10	159	159
SAUR2752 (SAR_RS13760)	2712490	2712885	6	129	129
SAUR2753 (SAR_RS13765)	2713098	2713976	9	244	244
SAUR2754 (SAR_RS13770)	2714143	2714361	1	1	1
SAUR2755 (SAR_RS13775)	2714750	2715181	9	139	139
SAUR2756 (SAR_RS13780)	2715321	2716601	146	8	8
SAUR2757 (SAR_RS13785)	2716856	2718022	8	322	322
SAUR2758 (SAR_RS13790)	2718193	2718714	52	193	193
SAUR2759 (SAR_RS13795)			62		
SAUR2764 (SAR_RS13820)	2726627	2727175	5	142	142
SAUR2765 (SAR_RS13825)	2727252	2727395	5	8	8
SAUR2766 (SAR_RS13830)	2727531	2729075	8	4	4
SAUR2767 (SAR_RS13835)	2729265	2729864	113	3	3
SAUR2768 (SAR_RS13840)	2730103	2730294	12	3	3
SAUR2769 (SAR_RS13845)	2730534	2732942	27	4	4
SAUR2770 (SAR_RS13850)	2733291	2733497	4	1	1
SAUR2771 (SAR_RS13855)	2733587	2734585	13	373	373
SAUR2772 (SAR_RS13860)	2734608	2735762	13	418	418
SAUR2773 (SAR_RS13865)	2736189	2737697	12	528	528
SAUR2774 (SAR_RS13870)	2737709	2738572	12	295	295
SAUR2775 (SAR_RS13875)	2738609	2739733	11	318	318
SAUR2776 (SAR_RS13880)	2739739	2741232	12	30	30
SAUR2777 (SAR_RS13885)	2741225	2741722	13	49	
SAUR2778 (SAR_RS13890)	2741888	2742655	7	256	256
SAUR2779 (SAR_RS13895)	2743017	2744828	12	445	445
SAUR2780 (SAR_RS13900)	2745503	2746204	12	285	285
SAUR2781 (SAR_RS13905)	2746811	2747866	12	355	355
SAUR2782 (SAR_RS13910)					

Locus	Start position in RGB-095930	End position in RGB-095930	Allele in NCTC8325, CP000253.1	Allele in RGB-095930	Allele in ST140_ERR1764920
SAUR2783 (SAR_RS13915)	2748258	2748845	140	229	229
SAUR2784 (SAR_RS13920)	2749223	2749543	73	131	131
SAUR2785 (SAR_RS13925)	2749763	2749993	4	4	4
SAUR2786 (SAR_RS13930)	2750295	2750672	10	144	144
SAUR2787 (SAR_RS13935)	2750689	2751510	8	4	4
SAUR2788 (SAR_RS13940)			14		
SAUR2789 (SAR_RS13945)			29		
SAUR2790 (SAR_RS13950)	2752509	2753213	13	267	4
SAUR2791 (SAR_RS13955)	2753311	2754321	230	398	
SAUR2792 (SAR_RS13960)	2754341	2755171	8	326	326
SAUR2793 (SAR_RS13965)	2755318	2756208	9	4	4
SAUR2794 (SAR_RS13970)	2756308	2757648		286	286
SAUR2795 (SAR_RS13975)	2757642	2758748	12	362	362
SAUR2796 (SAR_RS13980)	2759084	2759380	9	42	42
SAUR2797 (SAR_RS13985)	2759368	2759736	24	68	68
SAUR2798 (SAR_RS13990)	2759751	2760026	5	54	54
SAUR2799 (SAR_RS13995)	2760300	2761166	8	254	254
SAUR2800 (SAR_RS14000)	2761384	2762448	9	43	43
SAUR2801 (SAR_RS14005)	2762603	2762878	6	89	89
SAUR2802 (SAR_RS14010)	2763064	2763786		172	172
SAUR2803 (SAR_RS14015)	2764140	2764589	8	3	3
SAUR2806 (SAR_RS14030)	2765400	2767082	15	616	298
SAUR2807 (SAR_RS14035)	2767211	2767594	10	4	4
SAUR2808 (SAR_RS14040)	2767596	2768447	16	133	133
SAUR2809 (SAR_RS14045)	2768440	2769258	13	96	96
SAUR2810 (SAR_RS14050)	2769331	2770191	166	290	290
SAUR2811 (SAR_RS14055)	2770277	2770981	15	4	4
SAUR2812 (SAR_RS14060)	2771260	2772219	11	300	300
SAUR2813 (SAR_RS14065)	2772780	2772974	2	92	4
SAUR2814 (SAR_RS14070)	2772906	2774354		482	4
SAUR2815 (SAR_RS14075)	2774434	2775771	13	4	
SAUR2816 (SAR_RS14080)	2776025	2776441	7	4	4
SAUR2817 (SAR_RS14085)	2776564	2777454	121	101	101
SAUR2818 (SAR_RS14090)	2777646	2779142	10	4	4
SAUR2819 (SAR_RS14095)	2779569	2779769	4	1	1
SAUR2820 (SAR_RS14100)	2779829	2781427	18	483	483
SAUR2821 (SAR_RS14105)	2781621	2782064	14	23	23
SAUR2822 (SAR_RS14110)	2782344	2782556	1	1	1
SAUR2823 (SAR_RS14115)	2782834	2784543	11	423	423
SAUR2824 (SAR_RS14120)	2784804	2786294	10	383	383
SAUR2825 (SAR_RS14125)	2786363	2786458	1	5	5
SAUR2826 (SAR_RS14130)	2786547	2787110	17	101	101
SAUR2827 (SAR_RS14135)	2787279	2788901	15	4	4
SAUR2828 (SAR_RS14140)	2789033	2789194	117	14	14
SAUR2829 (SAR_RS14145)	2789210	2789320	5	6	6
SAUR2830 (SAR_RS14150)	2789419	2789955	11	17	17
SAUR2831 (SAR_RS14155)	2789952	2791802	11	261	261
SAUR2832 (SAR_RS14160)	2792049	2793419	24	197	197
SAUR2834 (SAR_RS14170)	2793908	2794516	37	3	3
SAUR2835 (SAR_RS14175)	2794582	2796462	27	305	305
SAUR2836 (SAR_RS14180)	2796653	2796745	5	1	1
SAUR2837 (SAR_RS14185)	2796853	2797350	7	1	1
SAUR2838 (SAR_RS14190)	2797360	2797611	10	173	173
SAUR2839 (SAR_RS14195)	2798002	2798115	16	1	1
SAUR2841 (SAR_RS14205)	2798784	2800784	17	646	646
SAUR2842 (SAR_RS14210)	2800781	2801536	12	282	282

Locus	Start position in RGB-095930	End position in RGB-095930	Allele in NCTC8325, CP000253.1	Allele in RGB-095930	Allele in ST140_ERR1764920
SAUR2843 (SAR_RS14215)	2801644	2802531	9	2	2
SAUR2844 (SAR_RS14220)	2802542	2803207	12	214	214
SAUR2845 (SAR_RS14225)	2803233	2803433	10	5	5
SAUR2846 (SAR_RS14230)	2803399	2803572	1	1	1
SAUR2848 (SAR_RS14240)					
SAUR2849 (SAR_RS14245)	2805338	2805793	8	1	1
SAUR2850 (SAR_RS14250)	2806037	2806798	9	268	268
SAUR2851 (SAR_RS14255)			466		
SAUR2852 (SAR_RS14260)	2809927	2810631	10	215	4
SAUR2853 (SAR_RS14265)			10		
SAUR2854 (SAR_RS14270)	2811688	2813118	16	412	
SAUR2855 (SAR_RS14275)	2813209	2814219	13	26	26
SAUR2856 (SAR_RS14280)	2814252	2815487	15	4	4
SAUR2857 (SAR_RS14285)			10		
SAUR2858 (SAR_RS14290)	2815835	2816284	6	113	113
SAUR2859 (SAR_RS14295)	2816652	2818181	182	543	
SAUR2861 (SAR_RS14305)					
SAUR2862 (SAR_RS14310)	2820075	2821949	12	487	487
SAUR2863 (SAR_RS14315)			23		
SAUR2864 (SAR_RS14320)	2823992	2824930	12	185	185
SAUR2865 (SAR_RS14325)	2825042	2828023	13	815	815
SAUR2866 (SAR_RS14330)	2828234	2830102	14	514	514
SAUR2867 (SAR_RS14335)	2830367	2830927	6	1	1
SAUR2868 (SAR_RS14340)	2831095	2832990	49	631	631
SAUR2869 (SAR_RS14345)	2833232	2834590	12	441	441
SAUR2870 (SAR_RS14350)	2834583	2836091	9	470	470
SAUR2871 (SAR_RS14355)	2836109	2838499	21	815	815
SAUR2873 (SAR_RS14365)	2839426	2840994	18	581	581
SAUR2874 (SAR_RS14370)					
SAUR2875 (SAR_RS14375)	2842548	2843759	31	396	396
SAUR2876 (SAR_RS14380)					
SAUR2877 (SAR_RS14385)	2850986	2851678	129	249	249
SAUR2879 (SAR_RS14395)	2852508	2852936	32	30	30
SAUR2880 (SAR_RS14400)			42		
SAUR2881 (SAR_RS14405)	2855166	2855525	11	81	81
SAUR2882 (SAR_RS14410)	2855610	2856221	7	204	204
SAUR2883 (SAR_RS14415)	2856300	2856776	19	178	178
SAUR2884 (SAR_RS14420)	2856779	2857279	9	4	4
SAUR2885 (SAR_RS14425)	2857514	2858281	11	275	275
SAUR2886 (SAR_RS14430)	2858278	2858970	14	4	4
SAUR2887 (SAR_RS14435)	2858987	2859649	6	266	266
SAUR2888 (SAR_RS14440)	2860496	2861056	8	92	
SAUR2889 (SAR_RS14445)	2861220	2862458	14	255	255
SAUR2890 (SAR_RS14450)	2862422	2862727	6	4	4
SAUR2891 (SAR_RS14455)	2862724	2863596	9	4	4
SAUR2892 (SAR_RS14460)	2863583	2864635	8	344	344
SAUR2893 (SAR_RS14465)	2865026	2867071	8	640	640
SAUR2895 (SAR_RS14475)	2868159	2868791	9	216	216
SAUR2896 (SAR_RS14480)	2868788	2869546		155	155
SAUR2897 (SAR_RS14485)	2869543	2870247	22	232	232
SAUR2898 (SAR_RS14490)			28		
SAUR2899 (SAR_RS14495)			42		
SAUR2900 (SAR_RS14500)	2871362	2872375	124	340	
SAUR2901 (SAR_RS14505)			101		
SAUR2902 (SAR_RS14510)	2873634	2874248	76	141	141
SAUR2903 (SAR_RS14515)	2874266	2875084	11	266	266

Locus	Start position in RGB-095930	End position in RGB-095930	Allele in NCTC8325, CP000253.1	Allele in RGB-095930	Allele in ST140_ERR1764920
SAUR2904 (SAR_RS14520)			6		
SAUR2910 (SAR_RS14550)	2876927	2877442	26	16	16
SAUR2911 (SAR_RS14555)			129		
SAUR2912 (SAR_RS14560)	2878807	2879763	13	304	304
SAUR2913 (SAR_RS14565)	2879917	2880555	68	65	65
SAUR2914 (SAR_RS14570)			18		
SAUR2916 (SAR_RS14580)			11		
SAUR2917 (SAR_RS14585)			21		237
SAUR2918 (SAR_RS14590)			21		
SAUR2919 (SAR_RS14595)	2888876	2889928	22	328	328
SAUR2920 (SAR_RS14600)	2890168	2890968	47	164	164
SAUR2921 (SAR_RS14605)	2891271	2891783	93	12	12
SAUR2922 (SAR_RS14610)	2892073	2892831	16	89	89
SAUR2923 (SAR_RS14615)	2892821	2894701	434	606	606
SAUR2929 (SAR_RS14645)	2896145	2896345	1	3	3
SAUR2930 (SAR_RS14650)			9		
SAUR2931 (SAR_RS14655)	2897292	2897687	4	6	6
SAUR2932 (SAR_RS14660)	2897755	2898108	6	16	16
SAUR2934 (SAR_RS14670)	2898663	2899502	9	235	235
SAUR2935 (SAR_RS14675)	2899545	2900264	11	4	4
SAUR2936 (SAR_RS14680)	2900264	2902141	14	441	441
SAUR2937 (SAR_RS14685)	2902208	2903587	19	360	360
SAUR2938 (SAR_RS14690)	2903731	2904078	4	4	4
SAUR2939 (SAR_RS14695)	2904205	2904342	2	2	2
