# Supplementary material for: Characterisation and Molecular Analysis of an Unusual Chimeric Methicillin Resistant Staphylococcus Aureus Strain and its Bacteriophages
Source: Front Genet. 2021 Nov 18;12:723958. doi: 10.3389/fgene.2021.723958 (PMC8638950; doi:10.3389/fgene.2021.723958)
Supplement: Supplementary file 7 [file DataSheet1.PDF]

STRAIN / ISOLATE	SPECIES MARKER							STAPHYLOXANTHIN BIOSYNTHESIS OPERON					REGULATORY GENES		
	Domain 1 of 23S-rRNA	glyceraldehyde 3-phosphate dehydrogenase, locus 1	katalase A	coagulase	thermostable extracellular nuclease	staphylococcal protein A	IgG-binding protein	dehydrosqualene synthase		dehydrosqualene desaturase	staphyloxanthin acyltransferase	diaponeurosporene oxidase	staphylococcal accessory regulator A	histidine protein kinase, sae locus	sensor protein
	rrnD1							crtM							
	rrnD1 (S. aureus)	gapA	katA	CoA	nuc1	spa	sbi	crtM-nonST93	crtM-ST93	crtN	crtO	crtP	sarA	saeS	vraS
**>CC8**		**POS**	**POS**	**POS**	**POS**	**POS**	**POS**	**POS**	**NEG**	**POS**	**POS**	**POS**	**POS**	**POS**	**POS**
NCTC8325 GenBank CP000253.1: Predicted Hybridisation Pattern	POS	POS	POS	POS	POS	POS	POS	POS	NEG	POS	POS	POS	AMB	POS	POS
NCTC 8325 = CIP 107700 = NARSA_77	POS	POS	POS	POS	POS	POS	POS	POS	NEG	POS	POS	POS	POS	POS	POS
COL GenBank CP000046.1: Predicted Hybridisation Pattern	POS	POS	POS	POS	POS	POS	POS	POS	NEG	POS	POS	POS	POS	POS	POS
COL	POS	POS	POS	POS	POS	POS	POS	POS	NEG	POS	POS	POS	POS	POS	POS
SVH7513 GenBank CP029166.1: Predicted Hybridisation Pattern	POS	POS	POS	POS	POS	POS	POS	POS	NEG	POS	POS	POS	POS	POS	POS
Uganda-03_643	POS	POS	POS	POS	POS	POS	POS	POS	NEG	POS	POS	POS	POS	POS	POS
Uganda-10_8347	POS	POS	POS	POS	POS	POS	POS	POS	NEG	POS	POS	POS	POS	POS	POS
Uganda-13_10674	POS	POS	POS	AMB	POS	POS	POS	POS	NEG	POS	POS	POS	POS	POS	POS
Uganda-21_12661	POS	POS	POS	POS	POS	POS	POS	POS	NEG	POS	POS	POS	POS	POS	POS
Uganda-23_12696	POS	POS	POS	POS	POS	POS	POS	POS	NEG	POS	POS	POS	POS	POS	POS
**>ST(43-3-1-1-4-4-3)**		**POS**	**POS**	**POS**	**POS**	**POS**	**POS**	**POS**	**NEG**	**POS**	**POS**	**POS**	**POS**	**POS**	**POS**
RGB-095930: Predicted Hybridisation Pattern	POS	POS	POS	POS	POS	POS	POS	POS	NEG	POS	POS	POS	POS	POS	POS
RGB-095930	POS	POS	POS	POS	POS	POS	POS	POS	NEG	POS	POS	POS	POS	POS	POS
ST6610_id-36080 (MLST Database): Predicted Hybr. Pattern	NEG	POS	POS	POS	POS	POS	POS	POS	NEG	POS	POS	POS	POS	POS	POS
ST6610_id-36082 (MLST Database): Predicted Hybr. Pattern	POS	POS	POS	POS	POS	POS	POS	POS	NEG	POS	POS	POS	POS	POS	POS
**>CC140**		**POS**	**POS**	**POS**	**POS**	**POS**	**POS**	**POS**	**NEG**	**POS**	**POS**	**POS**	**POS**	**POS**	**POS**
Kenyaseq6547225, ERR1764920: Predicted Hybridisation Pattern	POS	POS	POS	POS	POS	POS	POS	POS	NEG	POS	POS	POS	POS	POS	POS
SO-1977, SRR5682128: Predicted Hybridisation Pattern	POS	POS	POS	POS	POS	POS	POS	POS	NEG	POS	POS	POS	POS	POS	POS
Uganda-51_23201-2	POS	POS	POS	POS	POS	POS	POS	POS	NEG	POS	POS	POS	POS	POS	POS

STRAIN / ISOLATE	REGULATORY GENES																	
	accessory gene regulator allele I				accessory gene regulator allele II				accessory gene regulator allele III				accessory gene regulator allele IV			accessory gene regulator alleles from S. argenteus group		haemolysin delta
	agrI				agrII				agrIII				agrIV			agrV		
	agrI (total)	agrB-I	agrC-I	agrD-I	agrII (total)	agrB-II	agrC-II	agrD-II	agrIII (total)	agrB-III	agrC-III	agrD-III	agrIV (total)	agrB-IV	agrC-IV	agrV-ST1850	agrV-ST2198	hld
**>CC8**	**POS**	**POS**	**POS**	**POS**	**NEG**	**NEG**	**NEG**	**NEG**	**NEG**	**NEG**	**NEG**	**NEG**	**NEG**	**MB / VA**	**NEG**	**NEG**	**NEG**	**POS**
NCTC8325 GenBank CP000253.1: Predicted Hybridisation Pattern	POS	POS	POS	POS	NEG	NEG	NEG	NEG	NEG	NEG	NEG	NEG	NEG	NEG	NEG	NEG	NEG	POS
NCTC 8325 = CIP 107700 = NARSA_77	POS	POS	POS	POS	NEG	NEG	NEG	NEG	NEG	NEG	NEG	NEG	AMB	POS	NEG	NEG	NEG	POS
COL GenBank CP000046.1: Predicted Hybridisation Pattern	POS	POS	POS	POS	NEG	NEG	NEG	NEG	NEG	NEG	NEG	NEG	NEG	AMB	NEG	NEG	NEG	POS
COL	POS	POS	POS	POS	NEG	NEG	NEG	NEG	NEG	NEG	NEG	NEG	AMB	POS	NEG	NEG	NEG	POS
SVH7513 GenBank CP029166.1: Predicted Hybridisation Pattern	POS	POS	POS	POS	NEG	NEG	NEG	NEG	NEG	NEG	NEG	NEG	NEG	NEG	NEG	NEG	NEG	POS
Uganda-03_643	POS	POS	POS	POS	NEG	NEG	NEG	NEG	NEG	NEG	NEG	NEG	NEG	NEG	NEG	NEG	NEG	POS
Uganda-10_8347	POS	POS	POS	POS	NEG	NEG	NEG	NEG	NEG	NEG	NEG	NEG	NEG	NEG	NEG	NEG	NEG	POS
Uganda-13_10674	POS	POS	POS	POS	NEG	NEG	NEG	NEG	NEG	NEG	NEG	NEG	NEG	NEG	NEG	NEG	NEG	POS
Uganda-21_12661	POS	POS	POS	POS	NEG	NEG	NEG	NEG	NEG	NEG	NEG	NEG	NEG	NEG	NEG	NEG	NEG	POS
Uganda-23_12696	POS	POS	POS	POS	NEG	NEG	NEG	NEG	NEG	NEG	NEG	NEG	NEG	NEG	NEG	NEG	NEG	POS
**>ST(43-3-1-1-4-4-3)**	**POS**	**POS**	**POS**	**MB / VA**	**NEG**	**NEG**	**NEG**	**NEG**	**NEG**	**NEG**	**NEG**	**NEG**	**NEG**	**MB / VA**	**NEG**	**NEG**	**NEG**	**POS**
RGB-095930: Predicted Hybridisation Pattern	POS	POS	POS	AMB	NEG	NEG	NEG	NEG	NEG	NEG	NEG	NEG	NEG	NEG	NEG	NEG	NEG	POS
RGB-095930	POS	POS	POS	POS	NEG	NEG	NEG	NEG	NEG	NEG	NEG	NEG	AMB	POS	NEG	NEG	NEG	POS
ST6610_id-36080 (MLST Database): Predicted Hybr. Pattern	POS	POS	POS	AMB	NEG	NEG	NEG	NEG	NEG	NEG	NEG	NEG	NEG	NEG	NEG	NEG	NEG	POS
ST6610_id-36082 (MLST Database): Predicted Hybr. Pattern	POS	POS	POS	AMB	NEG	NEG	NEG	NEG	NEG	NEG	NEG	NEG	NEG	NEG	NEG	NEG	NEG	POS
**>CC140**	**NEG**	**MB / VA**	**POS**	**NEG**	**NEG**	**NEG**	**NEG**	**NEG**	**NEG**	**NEG**	**NEG**	**NEG**	**POS**	**POS**	**POS**	**NEG**	**NEG**	**POS**
Kenyaseq6547225, ERR1764920: Predicted Hybridisation Pattern	NEG	AMB	POS	NEG	NEG	NEG	NEG	NEG	NEG	NEG	NEG	NEG	POS	POS	POS	NEG	NEG	POS
SO-1977, SRR5682128: Predicted Hybridisation Pattern	NEG	AMB	POS	NEG	NEG	NEG	NEG	NEG	NEG	NEG	NEG	NEG	POS	POS	POS	NEG	NEG	POS
Uganda-51_23201-2	NEG	POS	POS	NEG	NEG	NEG	NEG	NEG	NEG	NEG	NEG	NEG	POS	POS	POS	NEG	NEG	POS

STRAIN / ISOLATE	METHICILLIN RESISTANCE AND SCCmec TYPING															
	Glycerophosphoryl diester phosphodiesterase. Accompanies mecA in nearly all SCCmec sequences.	Modified penicillin binding protein (PBP2a)	Truncated methicillin resistance operon repressor 1. Truncated mecR1 is present in SCCmec I, IV, V, VI,	Methicillin resistance operon repressor 1. Un-truncated sequence in SCCmec II, SCCmec III, SCCmec VIII	Methicillin-resistance regulatory protein. Present in SCCmec II (although absent from Irish SCCmec	Phenol soluble modulin from SCCmec	CsoR-like sulfur transferase-regulated genes B/metallo-beta-lactamase superfamily protein.	Methicillin resistance operon repressor 2, Homolog of xylose repressor. Located next to mec operon	Alternate gene encoding a modified penicillin binding protein . Present in, and characteristic for, SCCmec XI	Beta-lactamase from SCCmec XI	Plasmin-sensitive surface protein, prevents bacterial adhesion in vitro, located in SCC, close to mec operon. Subtyping	Truncated 3-hydroxy-3-methylglutaryl CoA synthase. Subtyping SCCmec I, II, IV, V	Putative protein next to dru	Putative protein	CsoR-like sulfur transferase-regulated genes B/metallo-beta-lactamase superfamily protein. Present in	Putative protein. Subtyping SCCmec I, SCCmec/ACME composites and SCCmec from WA40
	**ugpQ**	**mecA**	**delta_mecR1**	**mecR1**	**mecI**	**fudoh-PSM**	**cstB-SCC1 (ex Q2G1R6) (SCCmec II/III)**	**xylR/mecR2**	**mecC**	**blaZ (SCCmec XI) combined**	**plsSCC (COL)**	**mvaS-SCC**	**Q5HJW6**	**Q7A207**	**cstB-SCC2 (Q2G1R6)**	**Q9S0M4**
**>CC8**																
NCTC8325 GenBank CP000253.1: Predicted Hybridisation Pattern	NEG	NEG	NEG	NEG	NEG	NEG	NEG	NEG	NEG	NEG	NEG	NEG	NEG	NEG	NEG	NEG
NCTC 8325 = CIP 107700 = NARSA_77	NEG	NEG	NEG	NEG	NEG	NEG	NEG	NEG	neg	NEG	NEG	NEG	NEG	NEG	NEG	NEG
COL GenBank CP000046.1: Predicted Hybridisation Pattern	POS	POS	POS	NEG	NEG	NEG	NEG	NEG	NEG	NEG	POS	POS	POS	NEG	POS	POS
COL	POS	POS	POS	NEG	NEG	NEG	NEG	NEG	NEG	NEG	POS	POS	AMB	NEG	POS	POS
SVH7513 GenBank CP029166.1: Predicted Hybridisation Pattern	POS	POS	POS	NEG	NEG	NEG	NEG	NEG	NEG	NEG	NEG	POS	POS	AMB	NEG	NEG
Uganda-03_643	NEG	NEG	NEG	NEG	NEG	NEG	NEG	NEG	NEG	NEG	NEG	NEG	NEG	NEG	NEG	NEG
Uganda-10_8347	NEG	NEG	NEG	NEG	NEG	NEG	NEG	NEG	NEG	NEG	NEG	NEG	NEG	NEG	NEG	NEG
Uganda-13_10674	NEG	NEG	NEG	NEG	NEG	NEG	NEG	NEG	NEG	NEG	NEG	NEG	NEG	NEG	NEG	NEG
Uganda-21_12661	NEG	NEG	NEG	NEG	NEG	NEG	NEG	NEG	NEG	NEG	NEG	NEG	NEG	NEG	NEG	NEG
Uganda-23_12696	NEG	NEG	NEG	NEG	NEG	NEG	NEG	NEG	NEG	NEG	NEG	NEG	NEG	NEG	NEG	NEG
**>ST(43-3-1-1-4-4-3)**																
RGB-095930: Predicted Hybridisation Pattern	POS	POS	POS	NEG	NEG	NEG	NEG	NEG	NEG	NEG	NEG	POS	POS	NEG	NEG	NEG
RGB-095930	POS	POS	POS	NEG	NEG	NEG	NEG	NEG	NEG	NEG	NEG	POS	NEG	NEG	NEG	NEG
ST6610_id-36080 (MLST Database): Predicted Hybr. Pattern	POS	POS	POS	NEG	NEG	NEG	NEG	NEG	NEG	NEG	NEG	POS	POS	AMB	NEG	NEG
ST6610_id-36082 (MLST Database): Predicted Hybr. Pattern	POS	POS	POS	NEG	NEG	NEG	NEG	NEG	NEG	NEG	NEG	POS	POS	AMB	NEG	NEG
**>CC140**																
Kenyaseq6547225, ERR1764920: Predicted Hybridisation Pattern	POS	POS	POS	NEG	NEG	NEG	NEG	NEG	NEG	NEG	NEG	POS	POS	AMB	NEG	NEG
SO-1977, SRR5682128: Predicted Hybridisation Pattern	POS	POS	POS	NEG	NEG	NEG	NEG	NEG	NEG	NEG	NEG	POS	POS	AMB	NEG	NEG
Uganda-51_23201-2	POS	POS	POS	NEG	NEG	NEG	NEG	NEG	neg	NEG	NEG	POS	POS	POS	NEG	NEG

STRAIN / ISOLATE	kdpA-SCC	kdpB-SCC	kdpC-SCC	kdpD-SCC	kdpE-SCC	Q93IB7	D1GU38	D1GU38 (TW20)	Q933A2	D1GU55	F9JXC0 (full)	F9JXC0 (trunc)	B2Y834	B6VQU0	Q3YK51	ydhK (FPR3757)	hp1473_ydhK	hp1472_ydhK	C1PH94	DUF1958	Q4LAG7 (SCC consensus)	Q4LAG7 (SCCmecV, SO385)	Q4LAG7 (SCCfus 45394F/MSSA476)
	METHICILLIN RESISTANCE AND SCCmec TYPING																						
	Potassium-translocating ATPase A, chain 2	Potassium-transporting ATPase B, chain 1	Potassium-translocating ATPase C, chain 2	Sensor kinase protein	KDP operon transcriptional regulatory protein	LytTR domain DNA-binding regulator. Subtyping SCCmec III (present in, e.g., TW20 GenBank FN433596.1, but	Putative protein. Subtyping SCCmec III, identification of SCCmec VT, SCCmec ZH47, SCCmec VII because of an	Putative protein	Putative ADP-ribosyltransferase. Subtyping SCCmec III and SCCmec IX	Putative membrane protein. Subtyping SCCmec III, additional marker for SCCmec VII	KDP operon transcriptional regulatory protein		Abortive phage resistance protein. Subtyping SCCmec IV, i.e., identification of SCCmec IV A, G, c and SCCmec	Putative protein. Subtyping SCCmec IV, i.e., identification of SCCmec IVh/j	Putative protein. Subtyping SCCmec IV, i.e., identification of SCCmec IV g	Putative lipoprotein. Present in some composite elements comprising SCCmec and heavy metal resistance genes including the one in FPR3757, GenBank: CP000255.1			Putative protein	Putative protein. Subtyping SCCmec VT. Present, e.g., in PM1, GenBank BAFA but absent, e.g., in Strain 3957, GenBank	Putative protein located within SCCmec type V/SCCfus elements	Putative protein located within SCCmec type V elements	Putative protein located within SCCfus elements
							D1GU38				F9JXC0					ydhK					Q4LAG7		
**>CC8**																							
NCTC8325 GenBank CP000253.1: Predicted Hybridisation Pattern	NEG	NEG	NEG	NEG	NEG	NEG	NEG	NEG	NEG	NEG	NEG	NEG	NEG	NEG	NEG	NEG	NEG	NEG	NEG	NEG	NEG	NEG	NEG
NCTC 8325 = CIP 107700 = NARSA_77	NEG	NEG	NEG	NEG	NEG	NEG	NEG	NEG	NEG	NEG	NEG	NEG	NEG	NEG	NEG	NEG	NEG	NEG	NEG	NEG	NEG	NEG	NEG
COL GenBank CP000046.1: Predicted Hybridisation Pattern	NEG	NEG	NEG	NEG	NEG	NEG	NEG	NEG	NEG	NEG	NEG	NEG	NEG	NEG	NEG	NEG	NEG	NEG	NEG	NEG	NEG	NEG	NEG
COL	NEG	NEG	NEG	NEG	NEG	NEG	NEG	NEG	NEG	NEG	NEG	NEG	NEG	NEG	NEG	NEG	NEG	NEG	NEG	NEG	NEG	NEG	NEG
SVH7513 GenBank CP029166.1: Predicted Hybridisation Pattern	NEG	NEG	NEG	NEG	NEG	NEG	NEG	NEG	NEG	NEG	NEG	NEG	NEG	NEG	NEG	NEG	NEG	NEG	NEG	NEG	NEG	NEG	NEG
Uganda-03_643	NEG	NEG	NEG	NEG	NEG	NEG	NEG	NEG	NEG	NEG	NEG	NEG	NEG	NEG	NEG	NEG	NEG	NEG	NEG	NEG	NEG	NEG	NEG
Uganda-10_8347	NEG	NEG	NEG	NEG	NEG	NEG	NEG	NEG	NEG	NEG	NEG	NEG	NEG	NEG	NEG	NEG	NEG	NEG	NEG	NEG	NEG	NEG	NEG
Uganda-13_10674	NEG	NEG	NEG	NEG	NEG	NEG	NEG	NEG	NEG	NEG	NEG	NEG	NEG	NEG	NEG	NEG	NEG	NEG	NEG	NEG	NEG	NEG	NEG
Uganda-21_12661	NEG	NEG	NEG	NEG	NEG	NEG	NEG	NEG	NEG	NEG	NEG	NEG	NEG	NEG	NEG	NEG	NEG	NEG	NEG	NEG	NEG	NEG	NEG
Uganda-23_12696	NEG	NEG	NEG	NEG	NEG	NEG	NEG	NEG	NEG	NEG	NEG	NEG	NEG	NEG	NEG	NEG	NEG	NEG	NEG	NEG	NEG	NEG	NEG
**>ST(43-3-1-1-4-4-3)**																							
RGB-095930: Predicted Hybridisation Pattern	NEG	NEG	NEG	NEG	NEG	NEG	NEG	NEG	NEG	NEG	NEG	NEG	NEG	NEG	POS	NEG	NEG	NEG	NEG	NEG	NEG	NEG	NEG
RGB-095930	NEG	NEG	NEG	NEG	NEG	NEG	NEG	NEG	NEG	NEG	NEG	NEG	NEG	NEG	POS	NEG	NEG	NEG	NEG	NEG	NEG	NEG	NEG
ST6610_id-36080 (MLST Database): Predicted Hybr. Pattern	NEG	NEG	NEG	NEG	NEG	NEG	NEG	NEG	NEG	NEG	NEG	NEG	NEG	NEG	POS	NEG	NEG	NEG	NEG	NEG	NEG	NEG	NEG
ST6610_id-36082 (MLST Database): Predicted Hybr. Pattern	NEG	NEG	NEG	NEG	NEG	NEG	NEG	NEG	NEG	NEG	NEG	NEG	NEG	NEG	POS	NEG	NEG	NEG	NEG	NEG	NEG	NEG	NEG
**>CC140**																							
Kenyaseq6547225, ERR1764920: Predicted Hybridisation Pattern	NEG	NEG	NEG	NEG	NEG	NEG	NEG	NEG	NEG	NEG	NEG	NEG	NEG	NEG	POS	NEG	NEG	NEG	NEG	NEG	NEG	NEG	NEG
SO-1977, SRR5682128: Predicted Hybridisation Pattern	NEG	NEG	NEG	NEG	NEG	NEG	NEG	NEG	NEG	NEG	NEG	NEG	NEG	NEG	POS	NEG	NEG	NEG	NEG	NEG	NEG	NEG	NEG
Uganda-51_23201-2	NEG	NEG	NEG	NEG	NEG	NEG	NEG	NEG	NEG	NEG	NEG	NEG	NEG	NEG	POS	NEG	NEG	NEG	NEG	NEG	NEG	NEG	NEG

STRAIN / ISOLATE	METHICILLIN RESISTANCE AND SCCmec TYPING												
	Putative protein. Identification of SCCmec XI	Putative protein. Present in some SCCmec/fus composite elements such as, e.g., CMFT120, GenBank	Putative protein. Present in some composite SCC elements	CRISPR-associated endonuclease 1. Present in MSHR1132	CRISPR-associated endonuclease 1. Present in M06/0171, GenBank HE980450.1	SCC-associated fusidic acid resistance gene. Present alone in "SCCfus" elements or together with mecA in	Staphylococcal TIR-protein binding protein. Subtyping SCCfus because it is frequently, but not always, accompanying	Putative DNA methyltransferase. Subtyping SCCmecIV/fus composite elements	ACME total	Arginine deiminase	Ornithine carbamoyltransferase	Carbamate kinase	Arginine/ornithine antiporter
				cas1					ACME SCC locus				
	C5QAP8 (SCCmec XI)	Q8CU82	D3JD07	cas1 (MSHR1132)	cas1 (M06-171)	fusC (Q6GD50)	tirS	yeeA	ACME total	arcA-SCC	arcB-SCC	arcC-SCC	arcD-SCC
**>CC8**													
NCTC8325 GenBank CP000253.1: Predicted Hybridisation Pattern	NEG	NEG	NEG	NEG	NEG	NEG	NEG	NEG	NEG	NEG	NEG	NEG	NEG
NCTC 8325 = CIP 107700 = NARSA_77	NEG	NEG	NEG	NEG	NEG	NEG	NEG	NEG	NEG	NEG	NEG	NEG	NEG
COL GenBank CP000046.1: Predicted Hybridisation Pattern	NEG	NEG	NEG	NEG	NEG	NEG	NEG	NEG	NEG	NEG	NEG	NEG	NEG
COL	NEG	NEG	NEG	NEG	NEG	NEG	NEG	NEG	NEG	NEG	NEG	NEG	NEG
SVH7513 GenBank CP029166.1: Predicted Hybridisation Pattern	NEG	NEG	NEG	NEG	NEG	NEG	NEG	NEG	NEG	NEG	NEG	NEG	NEG
Uganda-03_643	NEG	NEG	NEG	NEG	NEG	NEG	NEG	NEG	NEG	NEG	NEG	NEG	NEG
Uganda-10_8347	NEG	NEG	NEG	NEG	NEG	NEG	NEG	NEG	NEG	NEG	NEG	NEG	NEG
Uganda-13_10674	NEG	NEG	NEG	NEG	NEG	NEG	NEG	NEG	NEG	NEG	NEG	NEG	NEG
Uganda-21_12661	NEG	NEG	NEG	NEG	NEG	NEG	NEG	NEG	NEG	NEG	NEG	NEG	NEG
Uganda-23_12696	NEG	NEG	NEG	NEG	NEG	NEG	NEG	NEG	NEG	NEG	NEG	NEG	NEG
**>ST(43-3-1-1-4-4-3)**													
RGB-095930: Predicted Hybridisation Pattern	NEG	NEG	NEG	NEG	NEG	NEG	NEG	NEG	NEG	NEG	NEG	NEG	NEG
RGB-095930	NEG	NEG	NEG	NEG	NEG	NEG	NEG	NEG	NEG	NEG	NEG	NEG	NEG
ST6610_id-36080 (MLST Database): Predicted Hybr. Pattern	NEG	NEG	NEG	NEG	NEG	NEG	NEG	NEG	NEG	NEG	NEG	NEG	NEG
ST6610_id-36082 (MLST Database): Predicted Hybr. Pattern	NEG	NEG	NEG	NEG	NEG	NEG	NEG	NEG	NEG	NEG	NEG	NEG	NEG
**>CC140**													
Kenyaseq6547225, ERR1764920: Predicted Hybridisation Pattern	NEG	NEG	NEG	NEG	NEG	NEG	NEG	NEG	NEG	NEG	NEG	NEG	NEG
SO-1977, SRR5682128: Predicted Hybridisation Pattern	NEG	NEG	NEG	NEG	NEG	NEG	NEG	NEG	NEG	NEG	NEG	NEG	NEG
Uganda-51_23201-2	NEG	NEG	NEG	NEG	NEG	NEG	NEG	NEG	NEG	NEG	NEG	NEG	NEG

STRAIN / ISOLATE	METHICILLIN RESISTANCE AND SCCmec TYPING																	
	Oligopeptide permease, channel-forming protein			Oligopeptide permease, channel-forming protein		Alcohol dehydrogenase, zinc-containing	Spermidine N-acetyltransferase. Usually associated with ACME or composite SCCmec/ACME elements	Cassette chromosome recombinase A, type 1	Cassette chromosome recombinase B, type 1	Cassette chromosome recombinase A, type 2	Cassette chromosome recombinase B, type 2	Cassette chromosome recombinase A, type 3	Cassette chromosome recombinase B, type 3	"Cassette chromosome recombinase AA"		Cassette chromosome recombinase C	Cassette chromosome recombinase A, type 4	Cassette chromosome recombinase B, type 4
	opp3B			opp3C		adhC	speG	ccrA/B-01		ccrA/B-02		ccrA/B-03		ccrAA/C			ccrA/B-04	
	opp3B	opp3B (FPR3757)	opp3B (C427)	opp3C (FPR3757)	opp3C (C427)			ccrA-1	ccrB-1	ccrA-2	ccrB-2	ccrA-3	ccrB-3	ccrAA (MRSAZH47)_probe 1	ccrAA (MRSAZH47)_probe 2	ccrC (85-2082)	ccrA-4	ccrB-4
**>CC8**																		
NCTC8325 GenBank CP000253.1: Predicted Hybridisation Pattern	NEG	NEG	NEG	NEG	NEG	NEG	NEG	NEG	NEG	NEG	NEG	NEG	NEG	NEG	NEG	NEG	NEG	NEG
NCTC 8325 = CIP 107700 = NARSA_77	NEG	NEG	NEG	NEG	NEG	NEG	NEG	NEG	NEG	NEG	NEG	NEG	NEG	NEG	NEG	NEG	NEG	NEG
COL GenBank CP000046.1: Predicted Hybridisation Pattern	NEG	NEG	NEG	NEG	NEG	NEG	NEG	POS	POS	NEG	NEG	NEG	AMB	NEG	NEG	NEG	NEG	NEG
COL	NEG	NEG	NEG	NEG	NEG	NEG	NEG	POS	POS	NEG	NEG	NEG	NEG	NEG	NEG	NEG	NEG	NEG
SVH7513 GenBank CP029166.1: Predicted Hybridisation Pattern	NEG	NEG	NEG	NEG	NEG	NEG	NEG	NEG	NEG	POS	POS	NEG	NEG	NEG	NEG	NEG	NEG	NEG
Uganda-03_643	NEG	NEG	NEG	NEG	NEG	NEG	NEG	NEG	NEG	NEG	NEG	NEG	NEG	NEG	NEG	NEG	NEG	NEG
Uganda-10_8347	NEG	NEG	NEG	NEG	NEG	NEG	NEG	NEG	NEG	NEG	NEG	NEG	NEG	NEG	NEG	NEG	NEG	NEG
Uganda-13_10674	NEG	NEG	NEG	NEG	NEG	NEG	NEG	NEG	NEG	NEG	NEG	NEG	NEG	NEG	NEG	NEG	NEG	NEG
Uganda-21_12661	NEG	NEG	NEG	NEG	NEG	NEG	NEG	NEG	NEG	NEG	NEG	NEG	NEG	NEG	NEG	NEG	NEG	NEG
Uganda-23_12696	NEG	NEG	NEG	NEG	NEG	NEG	NEG	NEG	NEG	NEG	NEG	NEG	NEG	NEG	NEG	NEG	NEG	NEG
**>ST(43-3-1-1-4-4-3)**																		
RGB-095930: Predicted Hybridisation Pattern	NEG	NEG	NEG	NEG	NEG	NEG	NEG	NEG	NEG	POS	POS	NEG	NEG	NEG	NEG	NEG	NEG	NEG
RGB-095930	NEG	NEG	NEG	NEG	NEG	NEG	NEG	NEG	NEG	POS	POS	NEG	NEG	NEG	NEG	NEG	NEG	NEG
ST6610_id-36080 (MLST Database): Predicted Hybr. Pattern	NEG	NEG	NEG	NEG	NEG	NEG	NEG	NEG	NEG	POS	POS	NEG	NEG	NEG	NEG	NEG	NEG	NEG
ST6610_id-36082 (MLST Database): Predicted Hybr. Pattern	NEG	NEG	NEG	NEG	NEG	NEG	NEG	NEG	NEG	POS	POS	NEG	NEG	NEG	NEG	NEG	NEG	NEG
**>CC140**																		
Kenyaseq6547225, ERR1764920: Predicted Hybridisation Pattern	NEG	NEG	NEG	NEG	NEG	NEG	NEG	NEG	NEG	POS	POS	NEG	NEG	NEG	NEG	NEG	NEG	NEG
SO-1977, SRR5682128: Predicted Hybridisation Pattern	NEG	NEG	NEG	NEG	NEG	NEG	NEG	NEG	NEG	POS	POS	NEG	NEG	NEG	NEG	NEG	NEG	NEG
Uganda-51_23201-2	NEG	NEG	NEG	NEG	NEG	NEG	NEG	NEG	NEG	POS	POS	NEG	NEG	NEG	NEG	NEG	NEG	NEG

STRAIN / ISOLATE	METHICILLIN RESISTANCE AND SCCmec TYPING															HEAVY METAL RESISTANCES	
	Located at the terminus of SCCmec directly next to orfX.This locus comprises the downstream constant segment	SCC integration site alternate to dcs														mercury resistance operon	
		Alternative SCC termini															
	Q9XB68-dcs	SCCmec Terminus 1	SCCmec Terminus 2	SCCmec Terminus 3	SCCmec Terminus 4	SCCmec Terminus 5	SCCmec Terminus 6	SCCmec Terminus 7	SCCmec Terminus 08	SCCmec Terminus 9	SCCmec Terminus 10	SCCmec Terminus 11	SCCmec Terminus 12	SCCmec Terminus 13	SCCmec Terminus 14	merA	merB
**>CC8**																	
NCTC8325 GenBank CP000253.1: Predicted Hybridisation Pattern	NEG	NEG	NEG	NEG	NEG	NEG	NEG	NEG	AMB	NEG	NEG	NEG	NEG	NEG	NEG	NEG	NEG
NCTC 8325 = CIP 107700 = NARSA_77	NEG	NEG	NEG	NEG	NEG	NEG	NEG	NEG	POS	NEG	NEG	NEG	NEG	NEG	NEG	NEG	NEG
COL GenBank CP000046.1: Predicted Hybridisation Pattern	POS	NEG	NEG	NEG	NEG	NEG	NEG	NEG	AMB	NEG	NEG	NEG	NEG	NEG	NEG	NEG	NEG
COL	POS	NEG	NEG	NEG	NEG	NEG	NEG	NEG	AMB	NEG	NEG	NEG	NEG	NEG	NEG	NEG	NEG
SVH7513 GenBank CP029166.1: Predicted Hybridisation Pattern	POS	NEG	NEG	NEG	NEG	NEG	NEG	NEG	AMB	NEG	NEG	NEG	NEG	NEG	NEG	NEG	NEG
Uganda-03_643	NEG	NEG	NEG	NEG	NEG	NEG	NEG	NEG	NEG	NEG	NEG	NEG	NEG	NEG	NEG	NEG	NEG
Uganda-10_8347	NEG	NEG	NEG	NEG	NEG	NEG	NEG	NEG	NEG	NEG	NEG	NEG	NEG	NEG	NEG	NEG	NEG
Uganda-13_10674	NEG	NEG	NEG	NEG	NEG	NEG	NEG	NEG	NEG	NEG	NEG	NEG	NEG	NEG	NEG	NEG	NEG
Uganda-21_12661	NEG	NEG	NEG	NEG	NEG	NEG	NEG	NEG	NEG	NEG	NEG	NEG	NEG	NEG	NEG	NEG	NEG
Uganda-23_12696	NEG	NEG	NEG	NEG	NEG	NEG	NEG	NEG	NEG	NEG	NEG	NEG	NEG	NEG	NEG	NEG	NEG
**>ST(43-3-1-1-4-4-3)**																	
RGB-095930: Predicted Hybridisation Pattern	POS	NEG	NEG	NEG	NEG	NEG	NEG	NEG	AMB	NEG	NEG	NEG	NEG	NEG	NEG	NEG	NEG
RGB-095930	POS	NEG	NEG	NEG	NEG	NEG	NEG	NEG	AMB	NEG	NEG	NEG	NEG	NEG	NEG	NEG	NEG
ST6610_id-36080 (MLST Database): Predicted Hybr. Pattern	POS	NEG	NEG	NEG	NEG	NEG	NEG	NEG	AMB	NEG	NEG	NEG	NEG	NEG	NEG	NEG	NEG
ST6610_id-36082 (MLST Database): Predicted Hybr. Pattern	POS	NEG	NEG	NEG	NEG	NEG	NEG	NEG	AMB	NEG	NEG	NEG	NEG	NEG	NEG	NEG	NEG
**>CC140**																	
Kenyaseq6547225, ERR1764920: Predicted Hybridisation Pattern	POS	NEG	NEG	NEG	NEG	NEG	NEG	NEG	AMB	NEG	NEG	NEG	NEG	NEG	NEG	NEG	NEG
SO-1977, SRR5682128: Predicted Hybridisation Pattern	POS	NEG	NEG	NEG	NEG	NEG	NEG	NEG	AMB	NEG	NEG	NEG	NEG	NEG	NEG	NEG	NEG
Uganda-51_23201-2	POS	NEG	NEG	NEG	NEG	NEG	NEG	AMB	AMB	NEG	NEG	NEG	NEG	NEG	NEG	NEG	NEG

STRAIN / ISOLATE	HEAVY METAL RESISTANCES																
	Multi copper oxidase	Copper exporting ATPase	Copper exporting ATPase	Multi copper oxidase	arsenical pump-driving ATPase	Putative dehydrogenase	trans-acting repressor of arsenic resistance operon	arsenical pump membrane protein							arsenate reductase		
	mco-plasmid	copA2-plasmi	copA2-SCC	mco-SCC	arsA	arsD	arsD2	arsB							arsC		
								arsB (SCC)			arsB (chromosomal)			arsB (plasmidic)	arsC (chromosomal)		arsC (SCC or plasmidic)
**>CC8**											**POS**	**POS**	**POS**		**POS**	**NEG**	
NCTC8325 GenBank CP000253.1: Predicted Hybridisation Pattern	NEG	NEG	NEG	NEG	NEG	NEG	NEG	NEG	NEG	NEG	POS	POS	POS	NEG	POS	NEG	NEG
NCTC 8325 = CIP 107700 = NARSA_77	NEG	NEG	NEG	NEG	NEG	NEG	NEG	NEG	NEG	NEG	POS	POS	POS	NEG	POS	POS	NEG
COL GenBank CP000046.1: Predicted Hybridisation Pattern	NEG	NEG	NEG	NEG	NEG	NEG	NEG	NEG	NEG	NEG	POS	POS	POS	NEG	POS	NEG	NEG
COL	NEG	NEG	NEG	NEG	NEG	NEG	NEG	NEG	NEG	NEG	POS	POS	POS	NEG	POS	POS	NEG
SVH7513 GenBank CP029166.1: Predicted Hybridisation Pattern	NEG	NEG	NEG	NEG	NEG	NEG	NEG	NEG	NEG	NEG	POS	POS	POS	NEG	POS	NEG	NEG
Uganda-03_643	NEG	NEG	NEG	NEG	NEG	NEG	NEG	NEG	NEG	NEG	POS	POS	POS	NEG	POS	NEG	NEG
Uganda-10_8347	NEG	NEG	NEG	NEG	NEG	NEG	NEG	NEG	NEG	NEG	POS	POS	POS	NEG	POS	NEG	NEG
Uganda-13_10674	NEG	NEG	NEG	NEG	NEG	NEG	NEG	NEG	NEG	NEG	POS	AMB	POS	NEG	POS	NEG	NEG
Uganda-21_12661	NEG	NEG	NEG	NEG	NEG	NEG	NEG	NEG	NEG	NEG	POS	POS	POS	NEG	POS	NEG	NEG
Uganda-23_12696	NEG	NEG	NEG	NEG	NEG	NEG	NEG	NEG	NEG	NEG	POS	POS	POS	NEG	POS	NEG	NEG
**>ST(43-3-1-1-4-4-3)**											**POS**	**POS**	**POS**		**POS**	**NEG**	
RGB-095930: Predicted Hybridisation Pattern	NEG	NEG	NEG	NEG	NEG	NEG	NEG	NEG	NEG	NEG	POS	POS	POS	NEG	POS	NEG	NEG
RGB-095930	NEG	NEG	NEG	NEG	NEG	NEG	NEG	NEG	NEG	NEG	POS	POS	POS	NEG	POS	NEG	NEG
ST6610_id-36080 (MLST Database): Predicted Hybr. Pattern	NEG	NEG	NEG	NEG	NEG	NEG	NEG	NEG	NEG	NEG	POS	POS	POS	NEG	POS	NEG	NEG
ST6610_id-36082 (MLST Database): Predicted Hybr. Pattern	NEG	NEG	NEG	NEG	NEG	NEG	NEG	NEG	NEG	NEG	POS	POS	POS	NEG	POS	NEG	NEG
**>CC140**											**POS**	**NEG**	**POS**		**NEG**	**NEG**	
Kenyaseq6547225, ERR1764920: Predicted Hybridisation Pattern	NEG	NEG	NEG	NEG	NEG	NEG	NEG	NEG	NEG	NEG	POS	NEG	POS	NEG	NEG	NEG	NEG
SO-1977, SRR5682128: Predicted Hybridisation Pattern	NEG	NEG	NEG	NEG	NEG	NEG	NEG	NEG	NEG	NEG	POS	NEG	POS	NEG	NEG	NEG	NEG
Uganda-51_23201-2	NEG	NEG	NEG	NEG	NEG	NEG	NEG	NEG	NEG	NEG	POS	NEG	POS	NEG	NEG	NEG	NEG

STRAIN / ISOLATE	HEAVY METAL RESISTANCES																		
	zink and cobalt transporter protein	cadmium and zinc resistance gene C, heavy metal translocating P-type ATPase. Frequently associated with	cadmium transporting ATPase				putative regulator of cadmium efflux		cadmium transport protein D								putative regulator of cadmium efflux		
			cadA				cadC		cadD								cadX (plasmid)		cadX (SCC)
	czrB	czrC	cadA (cons)	cadA (pTW20)	cadA (TN554)	cadA (Sepi)	cadC (TN554)	cadC (pI258)	cadD (total)	hp1548_cadD	hp1340_cadD	hp1339_cadD	hp1466_cadD	hp1547_cadD	cadD (R35)	cadD (Smas)	cadX (plasmid probe I)	cadX (plasmid probe II)	cadX (JCSC6943)
**>CC8**	**POS**																		
NCTC8325 GenBank CP000253.1: Predicted Hybridisation Pattern	POS	NEG	NEG	NEG	NEG	NEG	NEG	NEG	NEG	NEG	NEG	NEG	NEG	NEG	NEG	NEG	NEG	NEG	NEG
NCTC 8325 = CIP 107700 = NARSA_77	POS	NEG	NEG	NEG	NEG	NEG	NEG		NEG	NEG	NEG	NEG	NEG	NEG	NEG	NEG	NEG	NEG	NEG
COL GenBank CP000046.1: Predicted Hybridisation Pattern	POS	NEG	NEG	NEG	NEG	NEG	NEG	NEG	NEG	NEG	NEG	NEG	NEG	NEG	NEG	NEG	NEG	NEG	NEG
COL	POS	NEG	NEG	NEG	NEG	NEG	NEG	NEG	NEG	NEG	NEG	NEG	NEG	NEG	NEG	NEG	NEG	NEG	NEG
SVH7513 GenBank CP029166.1: Predicted Hybridisation Pattern	POS	NEG	NEG	NEG	NEG	NEG	NEG	NEG	POS	NEG	POS	POS	POS	NEG	NEG	NEG	POS	POS	NEG
Uganda-03_643	POS	NEG	NEG	NEG	NEG	NEG	NEG	NEG	NEG	NEG	NEG	NEG	NEG	NEG	NEG	NEG	NEG	NEG	NEG
Uganda-10_8347	POS	NEG	NEG	NEG	NEG	NEG	NEG	NEG	NEG	NEG	NEG	NEG	NEG	NEG	NEG	NEG	NEG	NEG	NEG
Uganda-13_10674	POS	NEG	NEG	NEG	NEG	NEG	NEG	NEG	NEG	NEG	NEG	NEG	NEG	NEG	NEG	NEG	NEG	NEG	NEG
Uganda-21_12661	POS	NEG	NEG	NEG	NEG	NEG	NEG	NEG	NEG	NEG	NEG	NEG	NEG	NEG	NEG	NEG	NEG	NEG	NEG
Uganda-23_12696	POS	NEG	NEG	NEG	NEG	NEG	NEG	NEG	NEG	NEG	NEG	NEG	NEG	NEG	NEG	NEG	NEG	NEG	NEG
**>ST(43-3-1-1-4-4-3)**	**POS**																		
RGB-095930: Predicted Hybridisation Pattern	POS	NEG	NEG	NEG	NEG	NEG	NEG	NEG	NEG	NEG	NEG	NEG	NEG	NEG	NEG	NEG	NEG	NEG	NEG
RGB-095930	POS	NEG	NEG	NEG	NEG	NEG	NEG	NEG	POS	NEG	POS	POS	POS	NEG	NEG	NEG	POS	POS	NEG
ST6610_id-36080 (MLST Database): Predicted Hybr. Pattern	POS	NEG	NEG	NEG	NEG	NEG	NEG	NEG	POS	NEG	POS	POS	POS	NEG	NEG	NEG	POS	POS	NEG
ST6610_id-36082 (MLST Database): Predicted Hybr. Pattern	POS	NEG	NEG	NEG	NEG	NEG	NEG	NEG	POS	NEG	POS	POS	POS	NEG	NEG	NEG	POS	POS	NEG
**>CC140**	**POS**																		
Kenyaseq6547225, ERR1764920: Predicted Hybridisation Pattern	POS	NEG	NEG	NEG	NEG	NEG	NEG	NEG	NEG	NEG	NEG	NEG	NEG	NEG	NEG	NEG	NEG	NEG	NEG
SO-1977, SRR5682128: Predicted Hybridisation Pattern	POS	NEG	NEG	NEG	NEG	NEG	NEG	NEG	NEG	NEG	NEG	NEG	NEG	NEG	NEG	NEG	NEG	NEG	NEG
Uganda-51_23201-2	POS	NEG	NEG	NEG	NEG	NEG	NEG	NEG	NEG	NEG	NEG	NEG	NEG	NEG	NEG	NEG	NEG	NEG	neg

STRAIN / ISOLATE	RESISTANCE : PENICILLINASE			RESISTANCE : MLS-ANTIBIOTICS															
	beta-lactamase	beta lactamase repressor (inhibitor)	beta-lactamase regulatory protein	rRNA adenine N-6-methyl-transferase, erythromycin/clindamycin resistance		erythro-mycin/clindamycin resistance	0,00	rRNA adenine methylase-like protein ErmGM	rRNA adenine N-6-methyltransferase	0,00	Lincosamid-Nucleotidyltransferase				lincosamide nucleotidyltransferase	lincosamide ABC transporter	lincosamide ABC transporter	energy-dependent efflux of erythro-mycin	
							ermC				linA/lnuA							msrA	
	blaZ	blaI	blaR	ermA	ermA-43	ermB	ermC	ermC-GM	ermF	ermT	linA/lnuA	hp_lnuA-831	hp_lnuA-832	hp_lnuA-833	lnuB	lsa-B	lsa-E	msrA	msrA (SarI)
**>CC8**																			
NCTC8325 GenBank CP000253.1: Predicted Hybridisation Pattern	NEG	NEG	NEG	NEG	NEG	NEG	NEG	NEG	NEG	NEG	NEG	NEG	NEG	NEG	NEG	NEG	NEG	NEG	
NCTC 8325 = CIP 107700 = NARSA_77	NEG	NEG	NEG	NEG	NEG	NEG	NEG	NEG	NEG	NEG	NEG	NEG	NEG	NEG	NEG	NEG	NEG	NEG	
COL GenBank CP000046.1: Predicted Hybridisation Pattern	NEG	NEG	NEG	NEG	NEG	NEG	NEG	NEG	NEG	NEG	NEG	NEG	NEG	NEG	NEG	NEG	NEG	NEG	
COL	NEG	NEG	NEG	NEG	NEG		NEG	NEG	NEG	NEG	NEG	NEG	NEG	NEG	NEG	NEG	NEG	NEG	
SVH7513 GenBank CP029166.1: Predicted Hybridisation Pattern	POS	POS	POS	NEG	NEG	NEG	POS	NEG	NEG	NEG	NEG	NEG	NEG	NEG	NEG	NEG	NEG	NEG	NEG
Uganda-03_643	POS	POS	POS	NEG	NEG	NEG	NEG	NEG	NEG	NEG	NEG	NEG	NEG	NEG	NEG	NEG	NEG	NEG	NEG
Uganda-10_8347	POS	POS	POS	NEG	NEG	NEG	NEG	NEG	NEG	NEG	NEG	NEG	NEG	NEG	NEG	NEG	NEG	NEG	NEG
Uganda-13_10674	POS	POS	POS	NEG	NEG	NEG	NEG	NEG	NEG	NEG	NEG	NEG	NEG	NEG	NEG	NEG	NEG	NEG	NEG
Uganda-21_12661	POS	POS	POS	NEG	NEG	NEG	NEG	NEG	NEG	NEG	NEG	NEG	NEG	NEG	NEG	NEG	NEG	NEG	NEG
Uganda-23_12696	POS	POS	POS	NEG	NEG	NEG	POS	NEG	NEG	NEG	NEG	NEG	NEG	NEG	NEG	NEG	NEG	NEG	NEG
**>ST(43-3-1-1-4-4-3)**																			
RGB-095930: Predicted Hybridisation Pattern	NEG	NEG	NEG	NEG	NEG	NEG	NEG	NEG	NEG	NEG	NEG	NEG	NEG	NEG	NEG	NEG	NEG	NEG	NEG
RGB-095930	POS	POS	POS	NEG	NEG	NEG	POS	NEG	NEG	NEG	NEG	NEG	NEG	NEG	NEG	NEG	NEG	NEG	NEG
ST6610_id-36080 (MLST Database): Predicted Hybr. Pattern	AMB	POS	POS	NEG	NEG	NEG	POS	NEG	NEG	NEG	NEG	NEG	NEG	NEG	NEG	NEG	NEG	NEG	NEG
ST6610_id-36082 (MLST Database): Predicted Hybr. Pattern	AMB	POS	POS	NEG	NEG	NEG	POS	NEG	NEG	NEG	NEG	NEG	NEG	NEG	NEG	NEG	NEG	NEG	NEG
**>CC140**																			
Kenyaseq6547225, ERR1764920: Predicted Hybridisation Pattern	POS	POS	POS	NEG	NEG	NEG	NEG	NEG	NEG	NEG	NEG	NEG	NEG	NEG	NEG	NEG	NEG	NEG	NEG
SO-1977, SRR5682128: Predicted Hybridisation Pattern	POS	POS	POS	NEG	NEG	NEG	NEG	NEG	NEG	NEG	NEG	NEG	NEG	NEG	NEG	NEG	NEG	NEG	NEG
Uganda-51_23201-2	POS	POS	POS	NEG	NEG	NEG	NEG	NEG	NEG	NEG	NEG	NEG	NEG	NEG	NEG	NEG	NEG	NEG	

STRAIN / ISOLATE	RESISTANCE : MLS-ANTIBIOTICS											RESISTANCE : AMINOGLYSOSIDES					
	macrolide efflux protein A	probable lysylphos phatidyl-glycerol synthetase	virginia-mycin A acetyltransferase	acetyl-transferase inactivating streptogramin A	ATP binding protein, streptogramin-A-resistance		streptogramin A ABC transporter	streptogramin A ABC transporter		streptogramin A ABC transporter	virginia-mycin B hydrolase	bifunctional enzyme Aac/Aph, gentamicin resistance	amino-glycoside adenyl-transferase,tobra mycin resistance	adenyltransferase AAd9		bleomycin resistance protein	aminoglycoside 6-adenylyltransferas e
														ant9			
	mefA	mph(C)	vat(A)	vat(B)	vga(A)	vga(A) (BM 3327)	vgaB	vgaC	vgaD	vgaE	vgb	aacA-aphD	aadD	ant9-C2944	ant9	ble	aad6
**>CC8**																	
NCTC8325 GenBank CP000253.1: Predicted Hybridisation Pattern	NEG	NEG	NEG	NEG	NEG	NEG	NEG	NEG	NEG	NEG	NEG	NEG	NEG	NEG	NEG	NEG	NEG
NCTC 8325 = CIP 107700 = NARSA_77	NEG	NEG	NEG	NEG	NEG	NEG	NEG	NEG	NEG	NEG	NEG	NEG	NEG	NEG	NEG	NEG	NEG
COL GenBank CP000046.1: Predicted Hybridisation Pattern	NEG	NEG	NEG	NEG	NEG	NEG	NEG	NEG	NEG	NEG	NEG	NEG	NEG	NEG	NEG	NEG	NEG
COL	NEG	NEG	NEG	NEG	NEG	NEG	NEG	NEG	NEG	NEG	NEG	NEG	NEG	NEG	NEG	NEG	NEG
SVH7513 GenBank CP029166.1: Predicted Hybridisation Pattern	NEG	NEG	NEG	NEG	NEG	NEG	NEG	NEG	NEG	NEG	NEG	NEG	NEG	NEG	NEG	NEG	NEG
Uganda-03_643	NEG	NEG	NEG	NEG	NEG	NEG	NEG	NEG	NEG	NEG	NEG	NEG	NEG	NEG	NEG	NEG	NEG
Uganda-10_8347	NEG	NEG	NEG	NEG	NEG	NEG	NEG	NEG	NEG	NEG	NEG	NEG	NEG	NEG	NEG	NEG	NEG
Uganda-13_10674	NEG	NEG	NEG	NEG	NEG	NEG	NEG	NEG	NEG	NEG	NEG	NEG	NEG	NEG	NEG	NEG	NEG
Uganda-21_12661	NEG	NEG	NEG	NEG	NEG	NEG	NEG	NEG	NEG	NEG	NEG	NEG	NEG	NEG	NEG	NEG	NEG
Uganda-23_12696	NEG	NEG	NEG	NEG	NEG	NEG	NEG	NEG	NEG	NEG	NEG	NEG	NEG	NEG	NEG	NEG	NEG
**>ST(43-3-1-1-4-4-3)**																	
RGB-095930: Predicted Hybridisation Pattern	NEG	NEG	NEG	NEG	NEG	NEG	NEG	NEG	NEG	NEG	NEG	POS	NEG	NEG	NEG	NEG	NEG
RGB-095930	NEG	NEG	NEG	NEG	NEG	NEG	NEG	NEG	NEG	NEG	NEG	POS	NEG	NEG	NEG	NEG	NEG
ST6610_id-36080 (MLST Database): Predicted Hybr. Pattern	NEG	NEG	NEG	NEG	NEG	NEG	NEG	NEG	NEG	NEG	NEG	POS	NEG	NEG	NEG	NEG	NEG
ST6610_id-36082 (MLST Database): Predicted Hybr. Pattern	NEG	NEG	NEG	NEG	NEG	NEG	NEG	NEG	NEG	NEG	NEG	POS	NEG	NEG	NEG	NEG	NEG
**>CC140**																	
Kenyaseq6547225, ERR1764920: Predicted Hybridisation Pattern	NEG	NEG	NEG	NEG	NEG	NEG	NEG	NEG	NEG	NEG	NEG	NEG	NEG	NEG	NEG	NEG	NEG
SO-1977, SRR5682128: Predicted Hybridisation Pattern	NEG	NEG	NEG	NEG	NEG	NEG	NEG	NEG	NEG	NEG	NEG	NEG	NEG	NEG	NEG	NEG	NEG
Uganda-51_23201-2	NEG	NEG	NEG	NEG	NEG	NEG	NEG	NEG	NEG	NEG	NEG	NEG	NEG	NEG	NEG	NEG	NEG

STRAIN / ISOLATE	RESISTANCE : AMINOGLYSOSIDES					RESISTANCE : MISCELLANEOUS GENES													
	aminoglycoside-3'-adenylyltransferase		streptomycin aminoglycoside 6-adenyltransferase		3'5'-aminog-lycoside phospho-transferase, neo-/ kanamycin resistance	strepto-thricine-acetyl-transferase	dihydro-folate reductase type 1	dihydrofolate reductase				fusidic acid resistence	mupirocin resistence protein	plasmidic isoleucyl-tRNA synthase	tetracycline resistance	Tetracycline resistance gene	tetracycline resistance		
	aadA		aadE					dfrG									tetM		
	aadA-var1	aadA-var2	aadE-C2944	aadE	aphA3	sat	dfrA	dfrG	dfrG-Tn559	dfrG-K	dfrG-D	far1	mupA	mupB	tetK	tetL	tetM (com-bined)	tetM-O	tetM-S
**>CC8**																			
NCTC8325 GenBank CP000253.1: Predicted Hybridisation Pattern	NEG	NEG	NEG	NEG	NEG	NEG	NEG	NEG	NEG	NEG	NEG	NEG	NEG	NEG	NEG	NEG	NEG	NEG	NEG
NCTC 8325 = CIP 107700 = NARSA_77	NEG	NEG	NEG	NEG	NEG	NEG	NEG	NEG	NEG	NEG	NEG	NEG	NEG	NEG	NEG	NEG	NEG	NEG	NEG
COL GenBank CP000046.1: Predicted Hybridisation Pattern	NEG	NEG	NEG	NEG	NEG	NEG	NEG	NEG	NEG	NEG	NEG	NEG	NEG	NEG	POS	NEG	NEG	NEG	NEG
COL	NEG	NEG	NEG	NEG	NEG	NEG	NEG	NEG	NEG	NEG	NEG	NEG	NEG	NEG	POS	NEG	NEG	NEG	NEG
SVH7513 GenBank CP029166.1: Predicted Hybridisation Pattern	NEG	NEG	NEG	NEG	NEG	NEG	POS	NEG	NEG	NEG	NEG	NEG	NEG	NEG	NEG	NEG	POS	NEG	NEG
Uganda-03_643	NEG	NEG	NEG	NEG	NEG	NEG	NEG	POS	AMB	AMB	AMB	NEG	NEG	NEG	POS	NEG	NEG	NEG	NEG
Uganda-10_8347	NEG	NEG	NEG	NEG	NEG	NEG	NEG	POS	AMB	AMB	AMB	NEG	NEG	NEG	NEG	NEG	NEG	NEG	NEG
Uganda-13_10674	NEG	NEG	NEG	NEG	NEG	NEG	NEG	POS	AMB	AMB	AMB	NEG	NEG	NEG	NEG	NEG	NEG	NEG	NEG
Uganda-21_12661	NEG	NEG	NEG	NEG	NEG	NEG	NEG	POS	AMB	AMB	AMB	NEG	NEG	NEG	POS	NEG	NEG	NEG	NEG
Uganda-23_12696	NEG	NEG	NEG	NEG	NEG	NEG	NEG	POS	AMB	AMB	AMB	NEG	NEG	NEG	POS	NEG	NEG	NEG	NEG
**>ST(43-3-1-1-4-4-3)**																			
RGB-095930: Predicted Hybridisation Pattern	NEG	NEG	NEG	NEG	NEG	NEG	NEG	POS	NEG	NEG	NEG	NEG	NEG	NEG	NEG	NEG	NEG	NEG	NEG
RGB-095930	NEG	NEG	NEG	NEG	NEG	NEG	NEG	POS	AMB	AMB	NEG	NEG	NEG	NEG	NEG	NEG	NEG	NEG	NEG
ST6610_id-36080 (MLST Database): Predicted Hybr. Pattern	NEG	NEG	NEG	NEG	NEG	NEG	NEG	POS	AMB	AMB	AMB	NEG	NEG	NEG	NEG	NEG	NEG	NEG	NEG
ST6610_id-36082 (MLST Database): Predicted Hybr. Pattern	NEG	NEG	NEG	NEG	NEG	NEG	NEG	POS	AMB	AMB	AMB	NEG	NEG	NEG	NEG	NEG	NEG	NEG	NEG
**>CC140**																			
Kenyaseq6547225, ERR1764920: Predicted Hybridisation Pattern	NEG	NEG	NEG	NEG	NEG	NEG	NEG	POS	AMB	AMB	AMB	NEG	NEG	NEG	NEG	NEG	POS	NEG	NEG
SO-1977, SRR5682128: Predicted Hybridisation Pattern	NEG	NEG	NEG	NEG	NEG	NEG	NEG	NEG	NEG	NEG	NEG	NEG	NEG	NEG	POS	NEG	POS	NEG	NEG
Uganda-51_23201-2	NEG	NEG	NEG	NEG	NEG	NEG	NEG	AMB	AMB	AMB	AMB	NEG	NEG	AMB	NEG	NEG	POS	NEG	NEG

STRAIN / ISOLATE	RESISTANCE : MISCELLANEOUS GENES																			
	chloramphenicol acetyltransferase						23S rRNA methyltransferase	chloramphenicol/florfenicol exporter	aminocyclitol acetyltransferase, confers apramycin resistance	metallothiol transferase				quaternary ammonium compound resistance protein A	quaternary ammonium compound resistance protein C					
	cat						cfr	fexA	apmA	fosB				qacA	qacC					
	cat	cat (pC221)	cat (pc223)	cat (pMC524)	cat (pSBK203R)	cat (Saga)	cfr	fexA	apmA	fosB	fosB (Sint)	fosB (plasmid 1)	fosB (plasmid 2)	qacA	qacC	qacC (cons)	qacC (equine)	qacC (SA5)	qacC (Ssap)	qacC (ST94)
**>CC8**																				
NCTC8325 GenBank CP000253.1: Predicted Hybridisation Pattern	NEG	NEG	NEG	NEG	NEG	NEG	NEG	NEG	NEG	POS	NEG	NEG	NEG	NEG	NEG	NEG	NEG	NEG	NEG	NEG
NCTC 8325 = CIP 107700 = NARSA_77	NEG	NEG	NEG	NEG	NEG	NEG	NEG	NEG	NEG	POS	NEG	NEG	NEG	NEG	NEG	NEG	NEG	NEG	NEG	NEG
COL GenBank CP000046.1: Predicted Hybridisation Pattern	NEG	NEG	NEG	NEG	NEG	NEG	NEG	NEG	NEG	POS	NEG	NEG	NEG	NEG	NEG	NEG	NEG	NEG	NEG	NEG
COL	NEG	NEG	NEG	NEG	NEG	NEG	NEG	NEG	NEG	POS	NEG	NEG	NEG	NEG	NEG	NEG	NEG	NEG	NEG	NEG
SVH7513 GenBank CP029166.1: Predicted Hybridisation Pattern	NEG	NEG	NEG	NEG	NEG	NEG	NEG	NEG	NEG	POS	NEG	NEG	NEG	NEG	NEG	NEG	NEG	NEG	NEG	NEG
Uganda-03_643	NEG	NEG	NEG	NEG	NEG	NEG	NEG	NEG	NEG	POS	NEG	NEG	NEG	NEG	NEG	NEG	NEG	NEG	NEG	NEG
Uganda-10_8347	NEG	NEG	NEG	NEG	NEG	NEG	NEG	NEG	NEG	POS	NEG	NEG	NEG	NEG	NEG	NEG	NEG	NEG	NEG	NEG
Uganda-13_10674	NEG	NEG	NEG	NEG	NEG	NEG	NEG	NEG	NEG	POS	NEG	NEG	NEG	NEG	NEG	NEG	NEG	NEG	NEG	NEG
Uganda-21_12661	NEG	NEG	NEG	NEG	NEG	NEG	NEG	NEG	NEG	POS	NEG	NEG	NEG	NEG	NEG	NEG	NEG	NEG	NEG	NEG
Uganda-23_12696	NEG	NEG	NEG	NEG	NEG	NEG	NEG	NEG	NEG	POS	NEG	NEG	NEG	NEG	NEG	NEG	NEG	NEG	NEG	NEG
**>ST(43-3-1-1-4-4-3)**																				
RGB-095930: Predicted Hybridisation Pattern	NEG	NEG	NEG	NEG	NEG	NEG	NEG	NEG	NEG	NEG	NEG	NEG	NEG	NEG	NEG	NEG	NEG	NEG	NEG	NEG
RGB-095930	NEG	NEG	NEG	NEG	NEG	NEG	NEG	NEG	NEG	NEG	NEG	NEG	NEG	NEG	POS	NEG	POS	NEG	NEG	NEG
ST6610_id-36080 (MLST Database): Predicted Hybr. Pattern	NEG	NEG	NEG	NEG	NEG	NEG	NEG	NEG	NEG	NEG	NEG	NEG	NEG	NEG	NEG	NEG	AMB	NEG	NEG	NEG
ST6610_id-36082 (MLST Database): Predicted Hybr. Pattern	NEG	NEG	NEG	NEG	NEG	NEG	NEG	NEG	NEG	NEG	NEG	NEG	NEG	NEG	NEG	NEG	AMB	NEG	NEG	NEG
**>CC140**																				
Kenyaseq6547225, ERR1764920: Predicted Hybridisation Pattern	NEG	NEG	NEG	NEG	NEG	NEG	NEG	NEG	NEG	NEG	NEG	NEG	NEG	NEG	NEG	NEG	NEG	NEG	NEG	NEG
SO-1977, SRR5682128: Predicted Hybridisation Pattern	NEG	NEG	NEG	NEG	NEG	NEG	NEG	NEG	NEG	NEG	NEG	NEG	NEG	NEG	NEG	NEG	NEG	NEG	NEG	NEG
Uganda-51_23201-2	NEG	NEG	NEG	NEG	NEG	neg	NEG	NEG	NEG	NEG	NEG	NEG	NEG	NEG	NEG	NEG	NEG	NEG	NEG	NEG

STRAIN / ISOLATE	RESISTANCE : MISCELLANEOUS GENES							VIRULENCE : TOX.SCHOCK.TOXIN			VIRULENCE : ENTEROTOXINS									
					vancomycin resistance gene	vancomycin resistance gene from enterococci and Clostridium	teicoplanin resistance gene from enterococci	toxic shock syndrome toxin 1			Enterotoxin A	Enterotoxin A, allele from strain 320E	Enterotoxin A, allele from strain N315 = Enterotoxin P	Enterotoxin B	Enterotoxin C	Enterotoxin D	Enterotoxin similar to EntD	Enterotoxin E	Enterotoxin H	Enterotoxin J
	smr							tst1			seA									
	smr (AL1)	smr (KACC16562)	smr (OJ82)	smr (VCU012)	vanA	vanB	vanZ	tst1 (consensus)	tst1 ("human" allele)	tst1 ("bovine" allele)	entA	entA (320E)	entA (N315) / entP	seB	seC	seD	seD2	seE	seH	seJ
**>CC8**																			NEG	
NCTC8325 GenBank CP000253.1: Predicted Hybridisation Pattern	NEG	NEG	NEG	NEG	NEG	NEG	NEG	NEG	NEG	NEG	NEG	NEG	NEG	NEG	NEG	NEG	NEG	NEG	NEG	NEG
NCTC 8325 = CIP 107700 = NARSA_77	NEG	NEG	NEG	NEG	NEG	NEG	NEG	NEG	NEG	NEG	NEG	NEG	NEG	NEG	NEG	NEG	NEG	NEG	NEG	NEG
COL GenBank CP000046.1: Predicted Hybridisation Pattern	NEG	NEG	NEG	NEG	NEG	NEG	NEG	NEG	NEG	NEG	NEG	NEG	NEG	POS	NEG	NEG	NEG	NEG	NEG	NEG
COL	NEG	NEG	NEG	NEG	NEG	NEG	NEG	NEG	NEG	NEG	NEG	NEG	NEG	POS	NEG	NEG	NEG	NEG	NEG	NEG
SVH7513 GenBank CP029166.1: Predicted Hybridisation Pattern	NEG	NEG	NEG	NEG	NEG	NEG	NEG	NEG	NEG	NEG	POS	NEG	NEG	POS	NEG	NEG	NEG	NEG	NEG	NEG
Uganda-03_643	NEG	NEG	NEG	NEG	NEG	NEG	NEG	NEG	NEG	NEG	POS	NEG	NEG	POS	NEG	NEG	NEG	NEG	NEG	NEG
Uganda-10_8347	NEG	NEG	NEG	NEG	NEG	NEG	NEG	NEG	NEG	NEG	POS	NEG	NEG	POS	NEG	NEG	NEG	NEG	NEG	NEG
Uganda-13_10674	NEG	NEG	NEG	NEG	NEG	NEG	NEG	NEG	NEG	NEG	POS	NEG	NEG	POS	NEG	NEG	NEG	NEG	NEG	NEG
Uganda-21_12661	NEG	NEG	NEG	NEG	NEG	NEG	NEG	NEG	NEG	NEG	POS	NEG	NEG	POS	NEG	NEG	NEG	NEG	NEG	NEG
Uganda-23_12696	NEG	NEG	NEG	NEG	NEG	NEG	NEG	NEG	NEG	NEG	POS	NEG	NEG	POS	NEG	NEG	NEG	NEG	NEG	NEG
**>ST(43-3-1-1-4-4-3)**																			NEG	
RGB-095930: Predicted Hybridisation Pattern	NEG	NEG	NEG	NEG	NEG	NEG	NEG	NEG	NEG	NEG	POS	NEG	NEG	POS	NEG	NEG	NEG	NEG	NEG	NEG
RGB-095930	NEG	NEG	NEG	NEG	NEG	NEG	NEG	NEG	NEG	NEG	POS	AMB	NEG	POS	NEG	NEG	NEG	NEG	NEG	NEG
ST6610_id-36080 (MLST Database): Predicted Hybr. Pattern	NEG	NEG	NEG	NEG	NEG	NEG	NEG	NEG	NEG	NEG	POS	NEG	NEG	POS	NEG	NEG	NEG	NEG	NEG	NEG
ST6610_id-36082 (MLST Database): Predicted Hybr. Pattern	NEG	NEG	NEG	NEG	NEG	NEG	NEG	NEG	NEG	NEG	POS	NEG	NEG	POS	NEG	NEG	NEG	NEG	NEG	NEG
**>CC140**																			NEG	
Kenyaseq6547225, ERR1764920: Predicted Hybridisation Pattern	NEG	NEG	NEG	NEG	NEG	NEG	NEG	NEG	NEG	NEG	NEG	NEG	NEG	NEG	NEG	NEG	NEG	NEG	NEG	NEG
SO-1977, SRR5682128: Predicted Hybridisation Pattern	NEG	NEG	NEG	NEG	NEG	NEG	NEG	POS	POS	NEG	NEG	NEG	NEG	NEG	POS	NEG	NEG	NEG	NEG	NEG
Uganda-51_23201-2	NEG	NEG	NEG	NEG	NEG	NEG	NEG	NEG	NEG	NEG	NEG	NEG	NEG	NEG	NEG	NEG	NEG	NEG	NEG	NEG

STRAIN / ISOLATE	Enterotoxin K	Enterotoxin L	Putative Enterotoxin N2	Enterotoxin Q	Enterotoxin R	Enterotoxin S	Enterotoxin T	Enterotoxin U2	Putative Enterotoxin	egc cluster	Enterotoxin G	Enterotoxin I	Enterotoxin M		Enterotoxin N			Enterotoxin O	Enterotoxin U and/or Y
															sen				
	seK	seL	seN2	seQ	seR	seS	seT	seU2	seW	egc (total)	selg	seli	selm	selm/i/v	seln (cons)	seln (other than RF122)	seln (argenteus)	selo	selu
**>CC8**										NEG	NEG	NEG	NEG		NEG	NEG	NEG	NEG	NEG
NCTC8325 GenBank CP000253.1: Predicted Hybridisation Pattern	NEG	NEG	NEG	NEG	NEG	NEG	NEG	NEG	NEG	NEG	NEG	NEG	NEG	NEG	NEG	NEG	NEG	NEG	NEG
NCTC 8325 = CIP 107700 = NARSA_77	NEG	NEG	NEG	NEG	NEG	NEG	NEG	NEG	NEG	NEG	NEG	NEG	NEG	NEG	NEG	NEG	NEG	NEG	NEG
COL GenBank CP000046.1: Predicted Hybridisation Pattern	POS	NEG	NEG	POS	NEG	NEG	NEG	NEG	NEG	NEG	NEG	NEG	NEG	NEG	NEG	NEG	NEG	NEG	NEG
COL	POS	NEG	NEG	POS	NEG	NEG	NEG	NEG	NEG	NEG	NEG	NEG	NEG	NEG	NEG	NEG	NEG	NEG	NEG
SVH7513 GenBank CP029166.1: Predicted Hybridisation Pattern	POS	NEG	NEG	POS	NEG	NEG	NEG	NEG	NEG	NEG	NEG	NEG	NEG	NEG	NEG	NEG	NEG	NEG	NEG
Uganda-03_643	POS	NEG	NEG	POS	NEG	NEG	NEG	NEG	NEG	NEG	NEG	NEG	NEG	NEG	NEG	NEG	NEG	NEG	NEG
Uganda-10_8347	POS	NEG	NEG	POS	NEG	NEG	NEG	NEG	NEG	NEG	NEG	NEG	NEG	NEG	NEG	NEG	NEG	NEG	NEG
Uganda-13_10674	POS	NEG	NEG	POS	NEG	NEG	NEG	NEG	NEG	NEG	NEG	NEG	NEG	NEG	NEG	NEG	NEG	NEG	NEG
Uganda-21_12661	POS	NEG	NEG	POS	NEG	NEG	NEG	NEG	NEG	NEG	NEG	NEG	NEG	NEG	NEG	NEG	NEG	NEG	NEG
Uganda-23_12696	POS	NEG	NEG	POS	NEG	NEG	NEG	NEG	NEG	NEG	NEG	NEG	NEG	NEG	NEG	NEG	NEG	NEG	NEG
**>ST(43-3-1-1-4-4-3)**										NEG	NEG	NEG	NEG		NEG	NEG	NEG	NEG	NEG
RGB-095930: Predicted Hybridisation Pattern	POS	NEG	NEG	POS	NEG	NEG	NEG	NEG	NEG	NEG	NEG	NEG	NEG	NEG	NEG	NEG	NEG	NEG	NEG
RGB-095930	POS	NEG	NEG	POS	NEG	NEG	NEG	NEG	NEG	NEG	NEG	NEG	NEG	NEG	AMB	NEG	NEG	NEG	NEG
ST6610_id-36080 (MLST Database): Predicted Hybr. Pattern	POS	NEG	NEG	POS	NEG	NEG	NEG	NEG	NEG	NEG	NEG	NEG	NEG	NEG	NEG	NEG	NEG	NEG	NEG
ST6610_id-36082 (MLST Database): Predicted Hybr. Pattern	POS	NEG	NEG	POS	NEG	NEG	NEG	NEG	NEG	NEG	NEG	NEG	NEG	NEG	NEG	NEG	NEG	NEG	NEG
**>CC140**										NEG	NEG	NEG	NEG		NEG	NEG	NEG	NEG	NEG
Kenyaseq6547225, ERR1764920: Predicted Hybridisation Pattern	NEG	NEG	NEG	NEG	NEG	NEG	NEG	NEG	NEG	NEG	NEG	NEG	NEG	NEG	NEG	NEG	NEG	NEG	NEG
SO-1977, SRR5682128: Predicted Hybridisation Pattern	NEG	POS	NEG	NEG	NEG	NEG	NEG	NEG	NEG	NEG	NEG	NEG	NEG	NEG	NEG	NEG	NEG	NEG	NEG
Uganda-51_23201-2	NEG	NEG	NEG	NEG	NEG	NEG	NEG	NEG	NEG	NEG	NEG	NEG	NEG	NEG	NEG	NEG	NEG	NEG	NEG

STRAIN / ISOLATE	ORF CM14		VIRULENCE : HLG AND LEUKOCIDINS															
	Enterotoxin-like protein ORF CM14		Haemolysin gamma / leukocidin, component B	Haemolysin gamma / leukocidin, component C		Haemolysin gamma, component A	intermedius group leukocidin		Panton Valentine leukocidin F component	Panton Valentine leukocidin S component	F component from ruminant leukocidin	S component from ruminant leukocidin	leukocidin D component	leukocidin E component	leukocidin/ haemolysin toxin family protein	leukocidin/haemolysin toxin family protein		
	ORF CM14			lukS												lukY		
	ORF CM14_ probe1	ORF CM14_ probe2	lukF	lukS	lukS (ST22+ ST45)	hlgA	lukF (int)	lukS (int)	lukF-PV	lukS-PV	lukF-PV (P83)	lukM	lukD	lukE	lukX	lukY	lukY (ST30+ST45)	lukY (ST1850)
**>CC8**	**NEG**	**NEG**	**POS**	**POS**	**MB / VA**	**POS**							**POS**	**POS**	**POS**	**POS**	**NEG**	**NEG**
NCTC8325 GenBank CP000253.1: Predicted Hybridisation Pattern	NEG	NEG	POS	POS	AMB	POS	NEG	NEG	NEG	NEG	NEG	NEG	POS	POS	POS	POS	NEG	NEG
NCTC 8325 = CIP 107700 = NARSA_77	NEG	NEG	POS	POS	AMB	POS	NEG		NEG	NEG	NEG	NEG	POS	POS	POS	POS	NEG	NEG
COL GenBank CP000046.1: Predicted Hybridisation Pattern	NEG	NEG	POS	POS	POS	POS	NEG	NEG	NEG	NEG	NEG	NEG	POS	POS	POS	POS	NEG	NEG
COL	NEG	NEG	POS	POS	AMB	POS	NEG	NEG	NEG	NEG	NEG	NEG	POS	POS	POS	POS	NEG	NEG
SVH7513 GenBank CP029166.1: Predicted Hybridisation Pattern	NEG	NEG	POS	POS	AMB	POS	NEG	NEG	NEG	NEG	NEG	NEG	POS	POS	POS	POS	NEG	NEG
Uganda-03_643	NEG	NEG	POS	POS	NEG	POS	NEG	NEG	NEG	NEG	NEG	NEG	POS	POS	POS	POS	NEG	NEG
Uganda-10_8347	NEG	NEG	POS	POS	NEG	POS	NEG	NEG	NEG	NEG	NEG	NEG	POS	POS	POS	POS	NEG	NEG
Uganda-13_10674	NEG	NEG	POS	POS	NEG	POS	NEG	NEG	NEG	NEG	NEG	NEG	POS	POS	POS	POS	NEG	NEG
Uganda-21_12661	NEG	NEG	POS	POS	NEG	POS	NEG	NEG	NEG	NEG	NEG	NEG	POS	POS	POS	POS	NEG	NEG
Uganda-23_12696	NEG	NEG	POS	POS	NEG	POS	NEG	NEG	NEG	NEG	NEG	NEG	POS	POS	POS	POS	NEG	NEG
**>ST(43-3-1-1-4-4-3)**	**NEG**	**NEG**	**POS**	**POS**	**MB / VA**	**POS**							**POS**	**POS**	**POS**	**POS**	**NEG**	**NEG**
RGB-095930: Predicted Hybridisation Pattern	NEG	NEG	POS	POS	AMB	POS	NEG	NEG	NEG	NEG	NEG	NEG	POS	POS	POS	POS	NEG	NEG
RGB-095930	NEG	NEG	POS	POS	POS	POS	NEG	NEG	NEG	NEG	NEG	NEG	POS	POS	POS	POS	NEG	NEG
ST6610_id-36080 (MLST Database): Predicted Hybr. Pattern	NEG	NEG	POS	POS	AMB	POS	NEG	NEG	NEG	NEG	NEG	NEG	POS	POS	POS	POS	NEG	NEG
ST6610_id-36082 (MLST Database): Predicted Hybr. Pattern	NEG	NEG	POS	POS	AMB	POS	NEG	NEG	NEG	NEG	NEG	NEG	POS	POS	POS	POS	NEG	NEG
**>CC140**	**NEG**	**NEG**	**POS**	**POS**	**MB / VA**	**POS**							**NEG**	**MB / VA**	**POS**	**NEG**	**POS**	**NEG**
Kenyaseq6547225, ERR1764920: Predicted Hybridisation Pattern	NEG	NEG	POS	POS	AMB	POS	NEG	NEG	NEG	NEG	NEG	NEG	NEG	AMB	POS	NEG	POS	NEG
SO-1977, SRR5682128: Predicted Hybridisation Pattern	NEG	NEG	POS	POS	AMB	POS	NEG	NEG	NEG	NEG	NEG	NEG	NEG	AMB	POS	NEG	POS	NEG
Uganda-51_23201-2	NEG	NEG	POS	POS	POS	POS	NEG	NEG	NEG	NEG	NEG	NEG	NEG	POS	POS	NEG	POS	NEG

STRAIN / ISOLATE	VIRULENCE : HAEMOLYSINS								VIRULENCE : HLB-CONV PHAGES		
	Putative membrane protein	Haemolysin alpha	Putative membrane protein		haemolysin beta				staphylo-kinase	chemotaxis-inhibiting protein (CHIPS)	Staphyl. Comple-ment inhibitor
			hlIII		hlb						
	corB (=hl)	hla	hlIII (cons)	hlIII (other than RF122)	hlb-probe 1	hlb-probe 2	hlb-probe 3	un-truncated hlb	sak	chp	scn
**>CC8**	**POS**	**POS**	**POS**	**POS**	**POS**	**POS**	**POS**				
NCTC8325 GenBank CP000253.1: Predicted Hybridisation Pattern	POS	POS	POS	POS	POS	POS	POS	POS	POS	POS	POS
NCTC 8325 = CIP 107700 = NARSA_77	POS	POS	POS	POS	POS	POS	POS	NEG	POS	POS	POS
COL GenBank CP000046.1: Predicted Hybridisation Pattern	POS	POS	POS	POS	POS	POS	POS	POS	NEG	NEG	NEG
COL	POS	POS	POS	POS	POS	POS	POS	POS	NEG	NEG	NEG
SVH7513 GenBank CP029166.1: Predicted Hybridisation Pattern	POS	POS	POS	POS	POS	POS	POS	POS	POS	NEG	POS
Uganda-03_643	POS	POS	POS	POS	POS	POS	POS	NEG	POS	NEG	POS
Uganda-10_8347	POS	POS	POS	POS	POS	POS	POS	NEG	POS	NEG	POS
Uganda-13_10674	POS	POS	POS	POS	POS	POS	POS	NEG	POS	NEG	POS
Uganda-21_12661	POS	POS	POS	POS	POS	POS	POS	NEG	POS	NEG	POS
Uganda-23_12696	POS	POS	POS	POS	POS	POS	POS	NEG	POS	NEG	POS
**>ST(43-3-1-1-4-4-3)**	**POS**	**POS**	**POS**	**POS**	**POS**	**POS**	**POS**				
RGB-095930: Predicted Hybridisation Pattern	POS	POS	POS	POS	POS	POS	POS	POS	POS	NEG	POS
RGB-095930	POS	POS	POS	POS	POS	POS	POS	NEG	POS	NEG	POS
ST6610_id-36080 (MLST Database): Predicted Hybr. Pattern	POS	POS	POS	POS	POS	POS	POS	POS	POS	NEG	POS
ST6610_id-36082 (MLST Database): Predicted Hybr. Pattern	POS	POS	POS	POS	POS	POS	POS	POS	POS	NEG	POS
**>CC140**	**POS**	**POS**	**POS**	**POS**	**NEG**	**NEG**	**NEG**				
Kenyaseq6547225, ERR1764920: Predicted Hybridisation Pattern	POS	POS	POS	POS	NEG	NEG	NEG	NEG	POS	NEG	POS
SO-1977, SRR5682128: Predicted Hybridisation Pattern	POS	POS	POS	POS	NEG	NEG	NEG	NEG	POS	NEG	POS
Uganda-51_23201-2	POS	POS	POS	POS	NEG	NEG	NEG	NEG	POS	NEG	POS

STRAIN / ISOLATE	VIRULENCE : OTHER FACTORS									VIRULENCE : PROTEASES		
	exfoliative toxin serotype A	exfoliative toxin serotype B	exfoliative toxin D	exfoliative toxin D2	epidermal cell differen-tiation inhibitor	epidermal cell differen-tiation inhibitor B	epidermal cell differen-tiation inhibitor C	virulence factor esxA	virulence factor esxB	aureolysin		
										aur		
	etA	etB	etD	etE / "etD2"	edinA	edinB	edinC	esxA	esxB	aur (cons)	aur (Other than MRSA252)	aur (MRSA252)
**>CC8**								**POS**	**POS**	**POS**	**POS**	**NEG**
NCTC8325 GenBank CP000253.1: Predicted Hybridisation Pattern	NEG	NEG	NEG	NEG	NEG	NEG	NEG	POS	POS	POS	POS	NEG
NCTC 8325 = CIP 107700 = NARSA_77	NEG	NEG	NEG	NEG	NEG	NEG	NEG	POS	POS	POS	POS	NEG
COL GenBank CP000046.1: Predicted Hybridisation Pattern	NEG	NEG	NEG	NEG	NEG	NEG	NEG	POS	POS	POS	POS	NEG
COL	NEG	NEG	NEG	NEG	NEG	NEG	NEG	POS	POS	POS	POS	NEG
SVH7513 GenBank CP029166.1: Predicted Hybridisation Pattern	NEG	NEG	NEG	NEG	NEG	NEG	NEG	POS	POS	POS	POS	NEG
Uganda-03_643	NEG	NEG	NEG	NEG	NEG	NEG	NEG	POS	POS	POS	POS	NEG
Uganda-10_8347	NEG	NEG	NEG	NEG	NEG	NEG	NEG	POS	POS	POS	POS	NEG
Uganda-13_10674	NEG	NEG	NEG	NEG	NEG	NEG	NEG	POS	POS	POS	POS	NEG
Uganda-21_12661	NEG	NEG	NEG	NEG	NEG	NEG	NEG	POS	POS	POS	POS	NEG
Uganda-23_12696	NEG	NEG	NEG	NEG	NEG	NEG	NEG	POS	POS	POS	POS	NEG
**>ST(43-3-1-1-4-4-3)**								**POS**	**POS**	**POS**	**NEG**	**POS**
RGB-095930: Predicted Hybridisation Pattern	NEG	NEG	NEG	NEG	NEG	NEG	NEG	POS	POS	POS	NEG	POS
RGB-095930	NEG	NEG	NEG	NEG	NEG	NEG	NEG	POS	POS	POS	NEG	POS
ST6610_id-36080 (MLST Database): Predicted Hybr. Pattern	NEG	NEG	NEG	NEG	NEG	NEG	NEG	POS	POS	POS	NEG	POS
ST6610_id-36082 (MLST Database): Predicted Hybr. Pattern	NEG	NEG	NEG	NEG	NEG	NEG	NEG	POS	POS	POS	NEG	POS
**>CC140**								**POS**	**NEG**	**POS**	**NEG**	**POS**
Kenyaseq6547225, ERR1764920: Predicted Hybridisation Pattern	NEG	NEG	NEG	NEG	NEG	NEG	NEG	POS	NEG	POS	NEG	POS
SO-1977, SRR5682128: Predicted Hybridisation Pattern	NEG	NEG	NEG	NEG	NEG	NEG	NEG	POS	NEG	POS	NEG	POS
Uganda-51_23201-2	NEG	NEG	NEG	NEG	NEG	NEG	NEG	POS	NEG	POS	NEG	POS

STRAIN / ISOLATE	VIRULENCE : PROTEASES							VIRULENCE : STAPHYLOCOCCAL SUPERANTIGEN/ENTEROTOXIN-LIKE GENES (SET/SSL)															
	serin- protease A	serin- protease B	serin- protease E	glutamylendopeptidase	Staphopain B, protease	Staphopain A (Staphylopain A), protease		Staphyl. exotoxin-like protein	Staphylococcal superantigen-like protein 1												Staphylococcal superantigen-like protein 2	
						sspP			ssl01												ssl02	
	splA	splB	splE	sspA	sspB	sspP (cons)	sspP (other than ST93)	setC / selX	set6-var1_11	set6-var2_11	set6-var1_12	set6-var2_12	set6-var4_11	ssl01-RF122	ssl01/set6 (COL)	ssl01/set6 (Mu50+ N315)	ssl01/set6 (MW2+ MSSA476)	ssl01/set6 (MRSA252)	ssl01/set6 (RF122)	ssl01/set6 (other alleles)	ssl02/set7	ssl02/set7 (MRSA252)
**>CC8**	POS	POS	POS	POS	POS	POS	POS	POS	POS	NEG	MB / VA	NEG	POS	NEG	POS	MB / VA	NEG	NEG	NEG	NEG	POS	MB / VA
NCTC8325 GenBank CP000253.1: Predicted Hybridisation Pattern	POS	POS	POS	POS	POS	POS	POS	POS	POS	NEG	AMB	NEG	POS	NEG	POS	AMB	NEG	NEG	NEG	NEG	POS	AMB
NCTC 8325 = CIP 107700 = NARSA_77	POS	POS	POS	POS	POS	POS	POS	POS	POS	NEG	AMB	NEG	POS	NEG	POS	NEG	NEG	NEG		NEG	POS	NEG
COL GenBank CP000046.1: Predicted Hybridisation Pattern	POS	POS	POS	POS	POS	POS	POS	POS	POS	NEG	POS	NEG	POS	NEG	POS	AMB	NEG	NEG	NEG	NEG	POS	AMB
COL	POS	POS	POS	POS	POS	POS	POS	POS	POS	NEG	POS	NEG	POS	NEG	POS	NEG	NEG	NEG	NEG	NEG	POS	NEG
SVH7513 GenBank CP029166.1: Predicted Hybridisation Pattern	POS	POS	POS	POS	POS	POS	POS	POS	POS	NEG	AMB	NEG	POS	NEG	POS	AMB	NEG	NEG	NEG	NEG	POS	AMB
Uganda-03_643	POS	POS	POS	POS	POS	POS	POS	POS	POS	NEG	NEG	NEG	POS	NEG	POS	AMB	NEG	NEG	NEG	NEG	POS	NEG
Uganda-10_8347	POS	POS	POS	POS	POS	POS	POS	POS	POS	NEG	NEG	NEG	POS	NEG	POS	AMB	NEG	NEG	NEG	NEG	POS	NEG
Uganda-13_10674	POS	POS	POS	POS	POS	POS	POS	POS	POS	NEG	NEG	NEG	POS	NEG	POS	AMB	NEG	NEG	NEG	NEG	POS	NEG
Uganda-21_12661	POS	POS	POS	POS	POS	POS	POS	POS	POS	NEG	NEG	NEG	POS	NEG	POS	AMB	NEG	NEG	NEG	NEG	POS	NEG
Uganda-23_12696	POS	POS	POS	POS	POS	POS	POS	POS	POS	NEG	NEG	NEG	POS	NEG	POS	AMB	NEG	NEG	NEG	NEG	POS	NEG
**>ST(43-3-1-1-4-4-3)**	POS	POS	POS	POS	POS	POS	POS	POS	POS	NEG	MB / VA	NEG	POS	NEG	POS	MB / VA	NEG	NEG	NEG	NEG	POS	NEG
RGB-095930: Predicted Hybridisation Pattern	POS	POS	POS	POS	POS	POS	POS	POS	POS	NEG	NEG	NEG	POS	NEG	POS	AMB	NEG	NEG	NEG	NEG	POS	NEG
RGB-095930	POS	POS	POS	POS	POS	POS	POS	POS	POS	AMB	POS	NEG	POS	NEG	POS	AMB	NEG	NEG	NEG	NEG	POS	AMB
ST6610_id-36080 (MLST Database): Predicted Hybr. Pattern	POS	POS	POS	POS	POS	POS	POS	POS	POS	NEG	AMB	NEG	POS	NEG	POS	AMB	NEG	NEG	NEG	NEG	POS	NEG
ST6610_id-36082 (MLST Database): Predicted Hybr. Pattern	POS	POS	POS	POS	POS	POS	POS	POS	POS	NEG	AMB	NEG	POS	NEG	POS	AMB	NEG	NEG	NEG	NEG	POS	NEG
**>CC140**	POS	MB / VA	NEG	POS	POS	POS	POS	POS	NEG	POS	POS	NEG	MB / VA	NEG	NEG	NEG	NEG	POS	NEG	NEG	MB / VA	MB / VA
Kenyaseq6547225, ERR1764920: Predicted Hybridisation Pattern	POS	AMB	NEG	POS	POS	POS	POS	POS	NEG	POS	POS	NEG	AMB	NEG	NEG	NEG	NEG	POS	NEG	NEG	AMB	AMB
SO-1977, SRR5682128: Predicted Hybridisation Pattern	POS	AMB	NEG	POS	POS	POS	POS	POS	NEG	POS	POS	NEG	AMB	NEG	NEG	NEG	NEG	POS	NEG	NEG	AMB	AMB
Uganda-51_23201-2	POS	POS	NEG	POS	POS	POS	POS	POS	NEG	POS	POS	NEG	POS	NEG	NEG	NEG	NEG	POS	NEG	NEG	AMB	POS

STRAIN / ISOLATE	VIRULENCE : STAPHYLOCOCCAL SUPERANTIGEN/ENTEROTOXIN-LIKE GENES (SET/SSL)																					
	Staphylococcal superantigen-like protein 3			Staphylococcal superantigen-like protein 4		Staphylococcal superantigen-like protein 5				Staphylococcal superantigen-like protein 6		Staphylococcal superantigen-like protein 7			Staphylococcal superantigen-like protein 8		Staphylococcal superantigen-like protein 9			Staphylococcal superantigen-like protein 10		
	ssl03			ssl04		ssl05				ssl06		ssl07			ssl08		ssl09			ssl10		
	ssl03/set8_ probe 1	ssl03/set8_ probe 2	ssl03/set8 (MRSA252, SAR0424)	ssl04/set9	ssl04/set9 (MRSA252, SAR0425)	ssl05/set3_ probe 1	ssl05/set3 (RF122, probe-611)	ssl05/set3_ probe 2 (612)	ssl05/set3 (MRSA252)	ssl06/set21	ssl06 (NCTC8325 +MW2)	ssl07/set1	ssl07/set1 (MRSA252)	ssl07/set1 (AF188836)	ssl08/set12 _probe 1	ssl08/set12 _probe 2	ssl09/set5_ probe 1	ssl09/set5_ probe 2	ssl09/set5 (MRSA252)	ssl10/set4	ssl10 (RF122)	ssl10/set4 (MRSA252)
**>CC8**	**POS**	**POS**	**NEG**	**POS**	**NEG**	**POS**	**MB / VA**	**POS**	**NEG**	**POS**	**POS**	**POS**	**MB / VA**	**NEG**	**POS**	**POS**	**POS**	**POS**	**NEG**	**POS**	**NEG**	**MB / VA**
NCTC8325 GenBank CP000253.1: Predicted Hybridisation Pattern	POS	POS	NEG	POS	NEG	POS	NEG	POS	NEG	POS	POS	POS	NEG	NEG	POS	POS	POS	POS	NEG	POS	NEG	NEG
NCTC 8325 = CIP 107700 = NARSA_77	POS	POS	NEG	POS	NEG	POS	NEG	POS	NEG	POS	POS	POS	AMB	NEG	POS	POS	POS	POS	NEG	POS	NEG	NEG
COL GenBank CP000046.1: Predicted Hybridisation Pattern	POS	POS	NEG	POS	NEG	NEG	NEG	NEG	NEG	NEG	NEG	NEG	NEG	NEG	NEG	NEG	POS	POS	NEG	POS	NEG	AMB
COL	POS	POS	NEG	POS	NEG	NEG	NEG	NEG	NEG	NEG	AMB	NEG	NEG	NEG	NEG	NEG	POS	POS	NEG	POS	NEG	NEG
SVH7513 GenBank CP029166.1: Predicted Hybridisation Pattern	POS	POS	NEG	POS	NEG	POS	NEG	POS	NEG	POS	POS	POS	NEG	NEG	POS	POS	POS	POS	NEG	POS	NEG	NEG
Uganda-03_643	POS	POS	NEG	POS	NEG	POS	NEG	POS	NEG	POS	POS	POS	AMB	NEG	POS	POS	POS	POS	NEG	POS	NEG	NEG
Uganda-10_8347	POS	POS	NEG	POS	NEG	POS	NEG	POS	NEG	POS	POS	POS	AMB	NEG	POS	POS	POS	POS	NEG	POS	NEG	NEG
Uganda-13_10674	POS	POS	NEG	POS	NEG	POS	NEG	POS	NEG	POS	POS	POS	AMB	NEG	POS	POS	POS	POS	NEG	POS	NEG	NEG
Uganda-21_12661	POS	POS	NEG	POS	NEG	POS	NEG	POS	NEG	POS	POS	POS	AMB	NEG	POS	POS	POS	POS	NEG	POS	NEG	NEG
Uganda-23_12696	POS	POS	NEG	POS	NEG	POS	NEG	POS	NEG	POS	POS	POS	AMB	NEG	POS	POS	POS	POS	NEG	POS	NEG	NEG
**>ST(43-3-1-1-4-4-3)**	**POS**	**POS**	**NEG**	**POS**	**NEG**	**POS**	**NEG**	**POS**	**NEG**	**POS**	**POS**	**POS**	**NEG**	**NEG**	**POS**	**POS**	**POS**	**POS**	**NEG**	**POS**	**NEG**	**NEG**
RGB-095930: Predicted Hybridisation Pattern	POS	POS	NEG	POS	NEG	POS	NEG	POS	NEG	POS	POS	POS	NEG	NEG	POS	POS	POS	POS	NEG	POS	NEG	NEG
RGB-095930	POS	POS	NEG	POS	NEG	POS	AMB	POS	NEG	POS	POS	POS	AMB	AMB	POS	POS	POS	POS	NEG	POS	AMB	NEG
ST6610_id-36080 (MLST Database): Predicted Hybr. Pattern	POS	POS	NEG	POS	NEG	POS	NEG	POS	NEG	POS	POS	POS	NEG	NEG	POS	POS	POS	POS	NEG	POS	NEG	NEG
ST6610_id-36082 (MLST Database): Predicted Hybr. Pattern	POS	POS	NEG	POS	NEG	POS	NEG	POS	NEG	POS	POS	POS	NEG	NEG	POS	POS	POS	POS	NEG	POS	NEG	NEG
**>CC140**	**NEG**	**NEG**	**NEG**	**NEG**	**MB / VA**	**NEG**	**NEG**	**NEG**	**POS**	**NEG**	**NEG**	**NEG**	**MB / VA**	**POS**	**NEG**	**NEG**	**NEG**	**NEG**	**POS**	**NEG**	**NEG**	**POS**
Kenyaseq6547225, ERR1764920: Predicted Hybridisation Pattern	NEG	NEG	NEG	NEG	POS	NEG	NEG	NEG	POS	NEG	NEG	NEG	NEG	POS	NEG	NEG	NEG	NEG	POS	NEG	NEG	POS
SO-1977, SRR5682128: Predicted Hybridisation Pattern	NEG	NEG	NEG	NEG	POS	NEG	NEG	NEG	POS	NEG	NEG	NEG	NEG	POS	NEG	NEG	NEG	NEG	POS	NEG	NEG	POS
Uganda-51_23201-2	NEG	NEG	NEG	NEG	POS	NEG	NEG	NEG	POS	NEG	AMB	AMB	POS	AMB	NEG	NEG	NEG	NEG	POS	AMB	NEG	POS

STRAIN / ISOLATE	VIRULENCE : STAPHYLOCOCCAL SUPERANTIGEN/ENTEROTOXIN-LIKE GENES (SET/SSL)									CAPSULE-ASSOCIATED GENES												
	Staphylococcal superantigene-like protein 11				Staphylococcal exotoxin-like protein, second locus					Capsule type 1	capsular poly-saccharide synthesis enzyme	O-antigen polymerase	capsular poly-saccharide biosyn-thesis protein	Capsule type 5	capsular poly-saccharide synthesis enzyme	O-antigen polymerase	capsular poly-saccharide biosyn-thesis protein	Capsule type 8	capsular poly-saccharide synthesis enzyme	capsular poly-saccharide biosyn-thesis protein	O-antigen poly-merase	capsular poly-saccharide biosyn-thesis protein
	ssl11				setB3		setB2		setB1	Capsule type 1				Capsule type 5				Capsule type 8				
	ssl11/set2 (COL)	ssl11/set2 (Mu50+ N315)	ssl11/set2 (MW2+ MSSA476)	ssl11/set2 (MRSA252)	setB3	setB3 (MRSA252)	setB2	setB2 (MRSA252)		cap 1	capH1	capJ1	capK1	cap 5	capH5	capJ5	capK5	cap 8	capH8	capI8	capJ8	capK8
**>CC8**	**POS**	**NEG**	**NEG**	**NEG**	**POS**	**NEG**	**POS**	**NEG**	**POS**					**POS**	**POS**	**POS**	**POS**	**NEG**	**NEG**	**NEG**	**NEG**	**NEG**
NCTC8325 GenBank CP000253.1: Predicted Hybridisation Pattern	POS	NEG	NEG	NEG	POS	NEG	POS	NEG	POS	NEG	NEG	NEG	NEG	POS	POS	POS	POS	NEG	NEG	NEG	NEG	NEG
NCTC 8325 = CIP 107700 = NARSA_77	POS	NEG	NEG	NEG	POS	NEG	POS	NEG	POS	NEG	NEG	NEG	NEG	POS	POS	POS	POS	NEG	NEG	NEG	NEG	NEG
COL GenBank CP000046.1: Predicted Hybridisation Pattern	POS	NEG	NEG	NEG	POS	NEG	POS	NEG	POS	NEG	NEG	NEG	NEG	POS	POS	POS	POS	NEG	NEG	NEG	NEG	NEG
COL	POS	NEG	NEG	NEG	POS	NEG	POS	NEG	POS	NEG	NEG	NEG	NEG	POS	POS	POS	POS	NEG	NEG	NEG	NEG	NEG
SVH7513 GenBank CP029166.1: Predicted Hybridisation Pattern	POS	NEG	NEG	NEG	POS	NEG	POS	NEG	POS	NEG	NEG	NEG	NEG	POS	POS	POS	POS	NEG	NEG	NEG	NEG	NEG
Uganda-03_643	POS	NEG	NEG	NEG	POS	NEG	POS	NEG	POS	NEG	NEG	NEG	NEG	POS	POS	POS	POS	NEG	NEG	NEG	NEG	NEG
Uganda-10_8347	POS	NEG	NEG	NEG	POS	NEG	POS	NEG	POS	NEG	NEG	NEG	NEG	POS	POS	POS	POS	NEG	NEG	NEG	NEG	NEG
Uganda-13_10674	POS	NEG	NEG	NEG	POS	NEG	POS	NEG	POS	NEG	NEG	NEG	NEG	POS	POS	POS	POS	NEG	NEG	NEG	NEG	NEG
Uganda-21_12661	POS	NEG	NEG	NEG	POS	NEG	POS	NEG	POS	NEG	NEG	NEG	NEG	POS	POS	POS	POS	NEG	NEG	NEG	NEG	NEG
Uganda-23_12696	POS	NEG	NEG	NEG	POS	NEG	POS	NEG	POS	NEG	NEG	NEG	NEG	POS	POS	POS	POS	NEG	NEG	NEG	NEG	NEG
**>ST(43-3-1-1-4-4-3)**	**POS**	**NEG**	**NEG**	**MB / VA**	**POS**	**NEG**	**POS**	**NEG**	**POS**					**POS**	**POS**	**POS**	**POS**	**NEG**	**NEG**	**NEG**	**NEG**	**NEG**
RGB-095930: Predicted Hybridisation Pattern	POS	NEG	NEG	NEG	POS	NEG	POS	NEG	POS	NEG	NEG	NEG	NEG	POS	POS	POS	POS	NEG	NEG	NEG	NEG	NEG
RGB-095930	POS	NEG	NEG	POS	POS	NEG	POS	AMB	POS	NEG	NEG	NEG	NEG	POS	POS	POS	POS	NEG	NEG	NEG	NEG	NEG
ST6610_id-36080 (MLST Database): Predicted Hybr. Pattern	POS	NEG	NEG	NEG	POS	NEG	POS	NEG	POS	NEG	NEG	NEG	NEG	POS	POS	POS	POS	NEG	NEG	NEG	NEG	NEG
ST6610_id-36082 (MLST Database): Predicted Hybr. Pattern	POS	NEG	NEG	NEG	POS	NEG	POS	NEG	POS	NEG	NEG	NEG	NEG	POS	POS	POS	POS	NEG	NEG	NEG	NEG	NEG
**>CC140**	**NEG**	**NEG**	**NEG**	**NEG**	**NEG**	**POS**	**NEG**	**POS**	**MB / VAR**					**POS**	**POS**	**POS**	**POS**	**NEG**	**NEG**	**NEG**	**NEG**	**NEG**
Kenyaseq6547225, ERR1764920: Predicted Hybridisation Pattern	NEG	NEG	NEG	NEG	NEG	POS	NEG	POS	AMB	NEG	NEG	NEG	NEG	POS	POS	POS	POS	NEG	NEG	NEG	NEG	NEG
SO-1977, SRR5682128: Predicted Hybridisation Pattern	NEG	NEG	NEG	NEG	NEG	POS	NEG	POS	AMB	NEG	NEG	NEG	NEG	POS	POS	POS	POS	NEG	NEG	NEG	NEG	NEG
Uganda-51_23201-2	NEG	NEG	NEG	NEG	NEG	POS	NEG	POS	POS	NEG	NEG	NEG	NEG	POS	POS	POS	POS	NEG	NEG	NEG	NEG	NEG

STRAIN / ISOLATE	icaA	icaC	icaD	bap	bbp	bbp (cons)	bbp (COL+MW2)	bbp (MRSA252)	bbp (Mu50)	bbp (RF122)	bbp (ST45)	clfA	clfA (cons)	clfA (COL+RF122)	clfA (MRSA252)	clfA (Mu50+MW2)	clfB	clfB (cons)	clfB (COL+Mu50)	clfB (MW2)	clfB (RF122)	cna
	BIOFILM-ASSOCIATED GENES				ADHAESION FACTORS / GENES ENCODING MICROBIAL SURFACE COMPONENTS RECOGNIZING ADHESIVE MATRIX MOLECULES (MSCRAMM GENES)																	
	intercellular adhesion protein A	intercellular adhesion protein C	biofilm PIA synthesis protein D	Surface protein involved in biofilm formation	Bone sialoprotein-binding protein							Clumping factor A					Clumping factor B					Collagen-binding adhesin
	icaA	icaC	icaD	bap	bbp							clfA					clfB					cna
**>CC8**	POS	POS	POS		POS	POS	POS	NEG	NEG	NEG	NEG	POS	POS	POS	MB / VA	MB / VA	POS	POS	POS	NEG	NEG	NEG
NCTC8325 GenBank CP000253.1: Predicted Hybridisation Pattern	POS	POS	POS	NEG	NEG	NEG	NEG	NEG	NEG	NEG	NEG	POS	POS	POS	NEG	AMB	POS	POS	POS	NEG	NEG	NEG
NCTC 8325 = CIP 107700 = NARSA_77	POS	POS	POS	NEG	NEG	NEG	NEG	NEG	NEG	NEG	NEG	POS	POS	POS	NEG	POS	POS	POS	POS	NEG	NEG	NEG
COL GenBank CP000046.1: Predicted Hybridisation Pattern	POS	POS	POS	NEG	POS	POS	POS	NEG	AMB	AMB	NEG	POS	POS	POS	NEG	AMB	POS	POS	POS	NEG	NEG	NEG
COL	POS	POS	POS	NEG	POS	POS	POS	NEG	NEG	NEG	NEG	POS	POS	POS	NEG	POS	POS	POS	POS	NEG	NEG	NEG
SVH7513 GenBank CP029166.1: Predicted Hybridisation Pattern	POS	POS	POS	NEG	POS	POS	POS	NEG	NEG	NEG	NEG	POS	POS	POS	NEG	AMB	POS	POS	POS	NEG	NEG	NEG
Uganda-03_643	POS	POS	POS	NEG	POS	POS	POS	NEG	NEG	NEG	NEG	POS	POS	POS	AMB	AMB	POS	POS	POS	NEG	NEG	NEG
Uganda-10_8347	POS	POS	POS	NEG	POS	POS	POS	NEG	NEG	NEG	NEG	POS	POS	POS	AMB	AMB	POS	POS	POS	NEG	NEG	NEG
Uganda-13_10674	POS	POS	POS	NEG	POS	POS	POS	NEG	NEG	NEG	NEG	POS	POS	POS	AMB	AMB	POS	POS	POS	NEG	NEG	NEG
Uganda-21_12661	POS	POS	POS	NEG	POS	POS	POS	NEG	NEG	NEG	NEG	POS	POS	POS	AMB	AMB	POS	POS	POS	NEG	NEG	NEG
Uganda-23_12696	POS	POS	POS	NEG	POS	POS	POS	NEG	NEG	NEG	NEG	POS	POS	POS	AMB	AMB	POS	POS	POS	NEG	NEG	NEG
**>ST(43-3-1-1-4-4-3)**	POS	POS	POS		POS	POS	POS	NEG	NEG	NEG	NEG	POS	POS	POS	NEG	MB / VA	POS	POS	NEG	NEG	MB / VA	POS
RGB-095930: Predicted Hybridisation Pattern	POS	POS	POS	NEG	POS	POS	POS	NEG	NEG	NEG	NEG	POS	POS	POS	NEG	AMB	POS	POS	NEG	NEG	NEG	POS
RGB-095930	POS	POS	POS	NEG	POS	POS	POS	NEG	AMB	NEG	NEG	POS	POS	POS	AMB	AMB	POS	POS	NEG	NEG	POS	POS
ST6610_id-36080 (MLST Database): Predicted Hybr. Pattern	POS	POS	POS	NEG	POS	POS	POS	NEG	NEG	NEG	NEG	POS	POS	POS	NEG	AMB	POS	POS	NEG	NEG	NEG	POS
ST6610_id-36082 (MLST Database): Predicted Hybr. Pattern	POS	POS	POS	NEG	POS	POS	POS	NEG	NEG	NEG	NEG	POS	POS	POS	NEG	AMB	POS	POS	NEG	NEG	NEG	POS
**>CC140**	POS	POS	POS		AMB / VA	MB / VA	NEG	NEG	NEG	NEG	MB / VA	POS	POS	NEG	NEG	POS	POS	POS	NEG	NEG	MB / VA	POS
Kenyaseq6547225, ERR1764920: Predicted Hybridisation Pattern	POS	POS	POS	NEG	NEG	NEG	NEG	NEG	NEG	NEG	NEG	POS	POS	NEG	NEG	POS	POS	POS	NEG	NEG	NEG	POS
SO-1977, SRR5682128: Predicted Hybridisation Pattern	POS	POS	POS	NEG	POS	POS	AMB	NEG	NEG	NEG	POS	POS	POS	NEG	NEG	POS	POS	POS	NEG	NEG	NEG	POS
Uganda-51_23201-2	POS	POS	POS	NEG	POS	POS	AMB	NEG	AMB	NEG	POS	POS	POS	NEG	AMB	POS	POS	POS	NEG	NEG	POS	POS

STRAIN / ISOLATE	ADHAESION FACTORS / GENES ENCODING MICROBIAL SURFACE COMPONENTS RECOGNIZING ADHESIVE MATRIX MOLECULES (MSCRAMM GENES)																					
	Cell wall associated fibronectin-binding protein	cell surface elastin binding protein					enolase	fibrinogen binding protein (19 kDa)		fibronectin-binding protein A						Fibronectin-binding protein B						
	ebh	ebpS						efb		fnbA						fnbB						
	ebh (cons)	ebpS	ebpS_probe 612	ebpS_probe 614	ebpS (01-1111)	ebpS (COL)	eno	efb / fib	efb / fib (MRSA252)	fnbA	fnbA (cons)	fnbA (COL)	fnbA (MRSA252)	fnbA (Mu50+MW2)	fnbA (RF122)	fnbB	fnbB (COL)	fnbB (COL+Mu50+MW2)	fnbB (Mu50)	fnbB (MW2)	fnbB (ST15)	fnbB (ST45-2)
**>CC8**	**POS**	**POS**	**POS**	**POS**	**NEG**	**POS**	**POS**	**POS**	**NEG**	**POS**	**POS**	**POS**	**NEG**	**NEG**	**NEG**	**POS**	**POS**	**MB / VA**	**NEG**	**NEG**	**NEG**	**NEG**
NCTC8325 GenBank CP000253.1: Predicted Hybridisation Pattern	POS	POS	POS	POS	NEG	POS	POS	POS	NEG	POS	POS	POS	NEG	NEG	NEG	POS	AMB	AMB	NEG	NEG	NEG	NEG
NCTC 8325 = CIP 107700 = NARSA_77	POS	POS	POS	POS	NEG	POS	POS	POS	NEG	POS	POS	POS	NEG	NEG	NEG	POS	POS	POS	NEG	NEG	NEG	NEG
COL GenBank CP000046.1: Predicted Hybridisation Pattern	POS	POS	POS	POS	NEG	POS	POS	POS	AMB	POS	POS	POS	NEG	NEG	NEG	POS	AMB	AMB	NEG	NEG	NEG	NEG
COL	POS	POS	POS	POS	NEG	POS	POS	POS	NEG	POS	POS	POS	NEG	NEG	NEG	POS	POS	POS	NEG	NEG	NEG	NEG
SVH7513 GenBank CP029166.1: Predicted Hybridisation Pattern	POS	POS	POS	POS	NEG	POS	POS	POS	NEG	POS	POS	POS	NEG	NEG	NEG	POS	AMB	AMB	NEG	NEG	NEG	NEG
Uganda-03_643	POS	POS	POS	POS	NEG	POS	POS	POS	NEG	POS	POS	POS	NEG	NEG	NEG	POS	AMB	POS	NEG	NEG	NEG	NEG
Uganda-10_8347	POS	POS	POS	POS	NEG	POS	POS	POS	NEG	POS	POS	POS	NEG	NEG	NEG	POS	POS	AMB	NEG	NEG	NEG	NEG
Uganda-13_10674	POS	POS	NEG	POS	NEG	POS	POS	POS	NEG	POS	POS	POS	NEG	NEG	NEG	POS	POS	AMB	NEG	NEG	NEG	NEG
Uganda-21_12661	POS	POS	AMB	POS	NEG	POS	POS	POS	NEG	POS	POS	POS	NEG	NEG	NEG	POS	AMB	POS	NEG	NEG	NEG	NEG
Uganda-23_12696	POS	POS	AMB	POS	NEG	POS	POS	POS	NEG	POS	POS	POS	NEG	NEG	NEG	POS	AMB	POS	NEG	NEG	NEG	NEG
**>ST(43-3-1-1-4-4-3)**	**POS**	**POS**	**POS**	**POS**	**NEG**	**POS**	**POS**	**POS**	**NEG**	**POS**	**POS**	**NEG**	**NEG**	**NEG**	**NEG**	**POS**	**NEG**	**MB / VA**	**NEG**	**NEG**	**NEG**	**MB / VA**
RGB-095930: Predicted Hybridisation Pattern	POS	POS	POS	POS	NEG	POS	POS	POS	NEG	POS	POS	NEG	NEG	NEG	NEG	POS	NEG	AMB	NEG	NEG	NEG	AMB
RGB-095930	POS	POS	POS	POS	NEG	POS	POS	POS	NEG	POS	POS	NEG	NEG	NEG	NEG	POS	NEG	AMB	NEG	NEG	NEG	POS
ST6610_id-36080 (MLST Database): Predicted Hybr. Pattern	POS	POS	POS	POS	NEG	POS	POS	POS	NEG	POS	POS	NEG	NEG	NEG	NEG	POS	NEG	AMB	NEG	NEG	NEG	AMB
ST6610_id-36082 (MLST Database): Predicted Hybr. Pattern	POS	POS	POS	POS	NEG	POS	POS	POS	NEG	POS	POS	NEG	NEG	NEG	NEG	POS	NEG	AMB	NEG	NEG	NEG	AMB
**>CC140**	**POS**	**POS**	**POS**	**POS**	**NEG**	**NEG**	**MB / VA**	**NEG**	**POS**	**POS**	**POS**	**NEG**	**NEG**	**NEG**	**NEG**	**POS**	**NEG**	**MB / VA**	**NEG**	**NEG**	**NEG**	**MB / VA**
Kenyaseq6547225, ERR1764920: Predicted Hybridisation Pattern	POS	POS	POS	POS	NEG	NEG	AMB	NEG	POS	POS	POS	NEG	NEG	NEG	NEG	POS	NEG	AMB	NEG	NEG	NEG	AMB
SO-1977, SRR5682128: Predicted Hybridisation Pattern	POS	POS	POS	POS	NEG	NEG	AMB	NEG	POS	POS	POS	NEG	NEG	NEG	NEG	POS	NEG	AMB	NEG	NEG	NEG	AMB
Uganda-51_23201-2	POS	POS	POS	POS	NEG	NEG	POS	NEG	POS	POS	POS	NEG	NEG	NEG	NEG	POS	NEG	AMB	NEG	NEG	NEG	POS

STRAIN / ISOLATE	ADHAESION FACTORS / GENES ENCODING MICROBIAL SURFACE COMPONENTS RECOGNIZING ADHESIVE MATRIX MOLECULES (MSCRAMM GENES)																				
	Major histocompatibility complex class II analog protein (=Extracellular adherence protein, eap)				Staphylococcus aureus surface protein G					Ser-Asp rich fibrinogen-/bone sialoprotein-binding protein C							Ser-Asp rich fibrinogen-/bone sialoprotein-binding protein D				
	map				sasG					sdrC							sdrD				
	map	map (COL)	map (MRSA252)	map (Mu50+MW2)	sasG	sasG (COL+Mu50)	sasG (MW2)	sasG (OtherThan252+122	sasX / sesI	sdrC	sdrC (cons)	sdrC (B1)	sdrC (COL)	sdrC (Mu50)	sdrC (MW2+MRSA252+RF122)	sdrC (OtherThan252+RF122)	sdrD	sdrD (cons)	sdrD (COL+MW2)	sdrD (Mu50)	sdrD (other)
**>CC8**	**POS**	**POS**	**NEG**	**NEG**	**POS**	**POS**	**NEG**	**POS**		**POS**	**POS**	**NEG**	**POS**	**NEG**	**NEG**	**POS**	**POS**	**POS**	**POS**	**NEG**	**NEG**
NCTC8325 GenBank CP000253.1: Predicted Hybridisation Pattern	POS	POS	NEG	NEG	POS	POS	NEG	POS	NEG	POS	POS	NEG	POS	NEG	NEG	POS	POS	POS	POS	NEG	NEG
NCTC 8325 = CIP 107700 = NARSA_77	POS	POS	NEG	NEG	POS	POS	NEG	POS	NEG	AMB	POS	NEG	POS	NEG	NEG	POS	POS	POS	POS	NEG	NEG
COL GenBank CP000046.1: Predicted Hybridisation Pattern	POS	POS	NEG	NEG	POS	POS	NEG	POS	NEG	POS	POS	NEG	POS	NEG	NEG	POS	POS	POS	POS	NEG	NEG
COL	POS	POS	NEG	NEG	POS	POS	NEG	POS	NEG	POS	POS	NEG	POS	NEG	NEG	POS	POS	POS	POS	NEG	NEG
SVH7513 GenBank CP029166.1: Predicted Hybridisation Pattern	POS	POS	NEG	NEG	POS	POS	NEG	POS	NEG	POS	POS	NEG	POS	NEG	NEG	POS	POS	POS	POS	NEG	NEG
Uganda-03_643	POS	POS	NEG	NEG	POS	POS	NEG	POS	NEG	POS	POS	NEG	POS	NEG	NEG	POS	POS	POS	POS	NEG	NEG
Uganda-10_8347	POS	POS	NEG	NEG	POS	POS	NEG	POS	NEG	POS	POS	NEG	POS	NEG	NEG	POS	POS	POS	POS	NEG	NEG
Uganda-13_10674	POS	POS	NEG	NEG	POS	POS	NEG	POS	NEG	POS	POS	NEG	POS	NEG	NEG	POS	POS	POS	POS	NEG	NEG
Uganda-21_12661	POS	POS	NEG	NEG	POS	POS	NEG	POS	NEG	POS	POS	NEG	POS	NEG	NEG	POS	POS	POS	POS	NEG	NEG
Uganda-23_12696	POS	POS	NEG	NEG	POS	POS	NEG	POS	NEG	POS	POS	NEG	POS	NEG	NEG	POS	POS	POS	POS	NEG	NEG
**>ST(43-3-1-1-4-4-3)**	**POS**	**POS**	**NEG**	**NEG**	**NEG**	**NEG**	**NEG**	**NEG**		**POS**	**POS**	**NEG**	**POS**	**NEG**	**NEG**	**POS**	**POS**	**POS**	**POS**	**NEG**	**NEG**
RGB-095930: Predicted Hybridisation Pattern	POS	POS	NEG	NEG	NEG	NEG	NEG	NEG	NEG	POS	POS	NEG	POS	NEG	NEG	POS	POS	POS	POS	NEG	NEG
RGB-095930	POS	POS	NEG	NEG	NEG	NEG	NEG	NEG	NEG	POS	POS	AMB	POS	NEG	NEG	POS	POS	POS	POS	NEG	NEG
ST6610_id-36080 (MLST Database): Predicted Hybr. Pattern	POS	POS	NEG	NEG	NEG	NEG	NEG	NEG	NEG	POS	POS	NEG	POS	NEG	NEG	POS	POS	POS	POS	NEG	NEG
ST6610_id-36082 (MLST Database): Predicted Hybr. Pattern	POS	POS	NEG	NEG	NEG	NEG	NEG	NEG	NEG	POS	POS	NEG	POS	NEG	NEG	POS	POS	POS	POS	NEG	NEG
**>CC140**	**MB / VAMB / VAMB / VA**			**NEG**	**NEG**	**NEG**	**NEG**	**NEG**		**POS**	**POS**	**NEG**	**NEG**	**MB / VAMB / VA**		**NEG**	**MB / VAMB / VA**		**NEG**	**NEG**	**NEG**
Kenyaseq6547225, ERR1764920: Predicted Hybridisation Pattern	NEG	NEG	AMB	NEG	NEG	NEG	NEG	NEG	NEG	POS	POS	NEG	NEG	NEG	NEG	NEG	NEG	NEG	NEG	NEG	NEG
SO-1977, SRR5682128: Predicted Hybridisation Pattern	NEG	NEG	AMB	NEG	NEG	NEG	NEG	NEG	NEG	POS	POS	NEG	NEG	NEG	NEG	NEG	POS	POS	NEG	AMB	NEG
Uganda-51_23201-2	POS	POS	POS	NEG	NEG	NEG	AMB	NEG	NEG	POS	POS	NEG	NEG	POS	POS	NEG	POS	POS	NEG	AMB	NEG

STRAIN / ISOLATE	ADHAESION FACTORS / MSCRAMM GENES						IMMUNOD.AG.B		DEFENSIN RESIST.		TRANSFERRIN BINDING PROT			PUTATIVE TRANSPORTER					TYPE I RESTRICTION-MODIFICATION SYSTEM, SINGLE SEQUENCE SPECIFICITY PROTEIN				
	van Willebrand factor binding protein						immunodominant antigen B		defensin resistance protein		transferrin-binding protein			hypothetical protein, similar to integral membrane protein LmrP					type I site-specific deoxyribo-nuclease subunit, 1st locus	type I site-specific deoxyribonuclease subunit, 2nd locus			
	vwb						isaB		mprF		isdA			lmrP					hsdS1	hsdS2			
	vwb	vwb (cons)	vwb (COL+MW2)	vwb (MRSA252)	vwb (Mu50)	vwb (RF122)	isaB	isaB (MRSA252)	mprF (COL+MW2)	mprF (Mu50+252)	isdA (cons)	isdA (MRSA252)	isdA (Other Than MRSA252)	lmrP (OtherThanRF122)	lmrP (OtherThanRF122)	lmrP (RF122)	lmrP (RF122)	lmrP-pWBG762	hsdS1-RF122	hsdS2-ST5+ST8	hsdS2-MW2+476	hsdS2-RF122	hsdS2-MRSA252
**>CC8**	**POS**	**POS**	**POS**	**NEG**	**NEG**	**NEG**	**POS**	**MB / VA**	**POS**	**MB / VA**	**POS**	**NEG**	**POS**	**POS**	**POS**	**NEG**	**NEG**			**POS**	**NEG**	**NEG**	**NEG**
NCTC8325 GenBank CP000253.1: Predicted Hybridisation Pattern	POS	POS	POS	NEG	NEG	NEG	POS	NEG	POS	NEG	POS	NEG	POS	POS	POS	NEG	NEG	NEG	NEG	POS	NEG	NEG	NEG
NCTC 8325 = CIP 107700 = NARSA_77	POS	POS	POS	NEG	NEG	NEG	POS	AMB	POS	NEG	POS	NEG	POS	POS	POS	NEG	NEG	NEG	NEG	POS	NEG	NEG	NEG
COL GenBank CP000046.1: Predicted Hybridisation Pattern	POS	POS	POS	NEG	NEG	NEG	POS	AMB	POS	AMB	POS	AMB	POS	POS	POS	NEG	NEG	NEG	NEG	POS	NEG	NEG	NEG
COL	POS	POS	POS	NEG	NEG	NEG	POS	POS	POS	NEG	POS	NEG	POS	POS	POS	NEG	NEG	NEG	NEG	POS	NEG	NEG	NEG
SVH7513 GenBank CP029166.1: Predicted Hybridisation Pattern	POS	POS	POS	NEG	NEG	NEG	POS	NEG	POS	NEG	POS	NEG	POS	POS	POS	NEG	NEG	NEG	NEG	POS	NEG	NEG	NEG
Uganda-03_643	POS	POS	POS	NEG	NEG	NEG	POS	AMB	POS	AMB	POS	NEG	POS	POS	POS	NEG	NEG	NEG	NEG	NEG	NEG	NEG	NEG
Uganda-10_8347	POS	POS	POS	NEG	NEG	NEG	POS	AMB	POS	AMB	POS	NEG	POS	POS	POS	NEG	NEG	NEG	NEG	POS	NEG	NEG	NEG
Uganda-13_10674	POS	POS	POS	NEG	NEG	NEG	POS	AMB	POS	NEG	POS	NEG	POS	POS	POS	NEG	NEG	NEG	NEG	POS	NEG	NEG	NEG
Uganda-21_12661	POS	POS	POS	NEG	NEG	NEG	POS	AMB	POS	NEG	POS	NEG	POS	POS	POS	NEG	NEG	NEG	NEG	POS	NEG	NEG	NEG
Uganda-23_12696	POS	POS	POS	NEG	NEG	NEG	POS	AMB	POS	NEG	POS	NEG	POS	POS	POS	NEG	NEG	NEG	NEG	POS	NEG	NEG	NEG
**>ST(43-3-1-1-4-4-3)**	**POS**	**POS**	**POS**	**NEG**	**NEG**	**NEG**	**NEG**	**POS**	**POS**	**NEG**	**POS**	**NEG**	**POS**	**POS**	**POS**	**NEG**	**NEG**			**POS**	**NEG**	**NEG**	**NEG**
RGB-095930: Predicted Hybridisation Pattern	POS	POS	POS	NEG	NEG	NEG	NEG	POS	POS	NEG	POS	NEG	POS	POS	POS	NEG	NEG	NEG	NEG	POS	NEG	NEG	NEG
RGB-095930	POS	POS	POS	NEG	NEG	NEG	NEG	POS	POS	AMB	POS	AMB	POS	POS	POS	NEG	NEG	NEG	NEG	POS	NEG	NEG	AMB
ST6610_id-36080 (MLST Database): Predicted Hybr. Pattern	POS	POS	POS	NEG	NEG	NEG	NEG	POS	POS	NEG	POS	NEG	POS	POS	POS	NEG	NEG	NEG	NEG	POS	NEG	NEG	NEG
ST6610_id-36082 (MLST Database): Predicted Hybr. Pattern	POS	POS	POS	NEG	NEG	NEG	NEG	POS	POS	NEG	POS	NEG	POS	POS	POS	NEG	NEG	NEG	NEG	POS	NEG	NEG	NEG
**>CC140**	**POS**	**POS**	**NEG**	**NEG**	**NEG**	**NEG**	**NEG**	**POS**	**NEG**	**MB / VA**	**POS**	**POS**	**NEG**	**POS**	**POS**	**NEG**	**NEG**			**NEG**	**NEG**	**NEG**	**NEG**
Kenyaseq6547225, ERR1764920: Predicted Hybridisation Pattern	POS	POS	NEG	NEG	NEG	NEG	NEG	POS	NEG	POS	POS	POS	NEG	POS	POS	NEG	NEG	NEG	NEG	NEG	NEG	NEG	NEG
SO-1977, SRR5682128: Predicted Hybridisation Pattern	POS	POS	NEG	NEG	NEG	NEG	NEG	POS	NEG	POS	POS	POS	NEG	POS	POS	NEG	NEG	NEG	NEG	NEG	NEG	NEG	NEG
Uganda-51_23201-2	POS	POS	NEG	NEG	NEG	NEG	NEG	POS	NEG	POS	POS	POS	AMB	POS	POS	NEG	NEG		NEG	NEG	AMB	NEG	NEG

STRAIN / ISOLATE	TYPE I RESTRICTION-MODIFICATION SYSTEM, SINGLE SEQUENCE SPECIFICITY PROTEIN								MISCELLANEOUS GENES															
	type I site-specific deoxyribonuclease subunit, 3rd locus					type I site-specific deoxyribonuclease subunit, unknown locus			Putative protein	Multidrug resistance transporter	Putative protein	major facilitator superfamily transporter	Putative protein				type II restriction-modification system endonuclease					acetyltransferase, GNAT family, "Argenteus/ST1850-like"		Putative bacteriocin biosynthesis associated protein
	hsdS3					hsdSx			ear2 = Q2FXC0	Q2YUB3	Q7A4X2	Q931R4 (CC5, CC15, CC30, CC97, CC188, ST1850)	Q9RL82			Q2G1R6-genomic island / cstB	sau				sau96I	G7ZRU6	ycjY	sagD
	hsdS3-AllOtherThanRF122+252	hsdS3-ST8+ST1+RF122	hsdS3-Mu50+N315	hsdS3-CC51+252	hsdS3-MRSA252	hsdSx-CC25	hsdSx-CC15	hsdSx-etd					Q9RL82 (consensus)	Q9RL82 (CC8)	Q9RL82-CC10/361		sau3AI	sauUSI	sauRF122	sauSO385	sau96I	G7ZRU6	ycjY = C5Q1F1 ("Argenteus/ST1850-like", CC12, CC361, CC398)	
**>CC8**	**POS**	**POS**	**NEG**	**NEG**	**NEG**	**POS**	**NEG**	**NEG**	**POS**	**NEG**	**NEG**	**NEG**	**POS**	**POS**	**NEG**	**POS**	**NEG**	**POS**	**NEG**	**NEG**	**NEG**	**NEG**	**NEG**	**NEG**
NCTC8325 GenBank CP000253.1: Predicted Hybridisation Pattern	POS	POS	NEG	NEG	NEG	POS	NEG	NEG	POS	NEG	NEG	NEG	POS	POS	NEG	POS	NEG	POS	NEG	NEG	NEG	NEG	NEG	NEG
NCTC 8325 = CIP 107700 = NARSA_77	POS	POS	NEG	NEG	NEG	POS	NEG	NEG	POS	NEG	NEG	NEG	POS	POS	NEG	POS	NEG	POS	NEG	NEG	NEG	NEG	NEG	NEG
COL GenBank CP000046.1: Predicted Hybridisation Pattern	POS	POS	NEG	NEG	NEG	POS	NEG	NEG	POS	NEG	NEG	NEG	POS	POS	AMB	POS	NEG	POS	NEG	NEG	NEG	NEG	NEG	NEG
COL	POS	POS	NEG	NEG	NEG	POS	NEG	NEG	POS	NEG	NEG	NEG	POS	POS	NEG	POS	NEG	POS	NEG	NEG	NEG	NEG	NEG	NEG
SVH7513 GenBank CP029166.1: Predicted Hybridisation Pattern	POS	POS	NEG	NEG	NEG	POS	NEG	NEG	POS	NEG	NEG	NEG	POS	POS	NEG	POS	NEG	POS	NEG	NEG	NEG	NEG	NEG	NEG
Uganda-03_643	POS	POS	NEG	NEG	NEG	POS	NEG	NEG	POS	NEG	NEG	NEG	POS	POS	NEG	POS	NEG	POS	NEG	NEG	NEG	NEG	NEG	NEG
Uganda-10_8347	POS	POS	NEG	NEG	NEG	POS	NEG	NEG	POS	NEG	NEG	NEG	POS	POS	NEG	POS	NEG	POS	NEG	NEG	NEG	NEG	NEG	NEG
Uganda-13_10674	POS	POS	NEG	NEG	NEG	POS	NEG	NEG	POS	NEG	NEG	NEG	POS	POS	NEG	POS	NEG	POS	NEG	NEG	NEG	NEG	NEG	NEG
Uganda-21_12661	POS	POS	NEG	NEG	NEG	POS	NEG	NEG	POS	NEG	NEG	NEG	POS	POS	NEG	POS	NEG	POS	NEG	NEG	NEG	NEG	NEG	NEG
Uganda-23_12696	POS	POS	NEG	NEG	NEG	POS	NEG	NEG	POS	NEG	NEG	NEG	POS	POS	NEG	POS	NEG	POS	NEG	NEG	NEG	NEG	NEG	NEG
**>ST(43-3-1-1-4-4-3)**	**POS**	**POS**	**NEG**	**NEG**	**NEG**	**POS**	**NEG**	**NEG**	**POS**	**NEG**	**NEG**	**NEG**	**NEG**	**NEG**	**POS**	**NEG**	**NEG**	**POS**	**NEG**	**NEG**	**NEG**	**NEG**	**POS**	**NEG**
RGB-095930: Predicted Hybridisation Pattern	POS	POS	NEG	NEG	NEG	POS	NEG	NEG	POS	NEG	NEG	NEG	NEG	NEG	POS	NEG	NEG	POS	NEG	NEG	NEG	NEG	POS	NEG
RGB-095930	POS	POS	NEG	NEG	NEG	POS	NEG	NEG	POS	NEG	NEG	NEG	AMB	NEG	POS	NEG	NEG	POS	NEG	NEG	NEG	NEG	POS	NEG
ST6610_id-36080 (MLST Database): Predicted Hybr. Pattern	POS	POS	NEG	NEG	NEG	POS	NEG	NEG	POS	NEG	NEG	NEG	AMB	NEG	POS	NEG	NEG	POS	NEG	NEG	NEG	NEG	POS	NEG
ST6610_id-36082 (MLST Database): Predicted Hybr. Pattern	POS	POS	NEG	NEG	NEG	POS	NEG	NEG	POS	NEG	NEG	NEG	AMB	NEG	POS	NEG	NEG	POS	NEG	NEG	NEG	NEG	POS	NEG
**>CC140**	**NEG**	**POS**	**NEG**	**NEG**	**NEG**	**MB / VA**	**MB / VA**	**NEG**	**NEG**	**NEG**	**NEG**	**NEG**	**MB / VA**	**NEG**	**POS**	**NEG**	**NEG**	**POS**	**NEG**	**NEG**	**NEG**	**NEG**	**POS**	**NEG**
Kenyaseq6547225, ERR1764920: Predicted Hybridisation Pattern	NEG	POS	NEG	NEG	NEG	AMB	NEG	NEG	NEG	NEG	NEG	NEG	AMB	NEG	POS	NEG	NEG	POS	NEG	NEG	NEG	NEG	POS	NEG
SO-1977, SRR5682128: Predicted Hybridisation Pattern	NEG	POS	NEG	NEG	NEG	AMB	NEG	NEG	NEG	NEG	NEG	NEG	AMB	NEG	POS	NEG	NEG	POS	NEG	NEG	NEG	NEG	POS	NEG
Uganda-51_23201-2	AMB	POS	NEG	NEG	NEG	POS	POS	NEG	NEG	AMB	NEG	NEG	POS	NEG	POS	NEG	NEG	POS	NEG	NEG	NEG	NEG	POS	NEG

STRAIN / ISOLATE	MISCELLANEOUS GENES											HYALURONATE LYASE							
	TetR family regulator		Multidrug resistance transporter						Multidrug resistance transporter			Hyaluronate lyase, variable first / second locus			Hyaluronate lyase, second locus				
	G7ZTC1		sdrM						Q2YUB3			hysA1			hysA2				
	G7ZTC1	G7ZTC1-argenteus	sdrM / tetEfflux	sdrM (cons)	hp_sdrM-801	hp_sdrM-802	sdrM (CC30)	sdrM (argenteus)	Q2YUB3 (RF122)	Q2YUB3 (Swar)	Q2YUB3 (Sepi)	hysA1 (MRSA252)	hysA1 (MRSA252+RF122) and/or hysA2 (cons)	hysA1 (MRSA252+RF122) and/or hysA2 (COL+USA300)	hysA2 (All Other Than MRSA252)	hysA2 (COL+USA300+NCTC8325)	hysA2 (All Other Than COL+USA300+NCTC8325)	hysA2 (All Other Than COL+USA300+NCTC8325)	hysA2 (MRSA252)
**>CC8**	**NEG**	**NEG**	**POS**	**NEG**	**POS**	**MB / VA**	**NEG**	**NEG**	**NEG**	**NEG**	**NEG**	**NEG**	**POS**	**MB / VA**	**POS**	**POS**	**NEG**	**NEG**	**NEG**
NCTC8325 GenBank CP000253.1: Predicted Hybridisation Pattern	NEG	NEG	POS	NEG	POS	NEG	NEG	NEG	NEG	NEG	NEG	NEG	POS	NEG	POS	POS	NEG	NEG	NEG
NCTC 8325 = CIP 107700 = NARSA_77	NEG	NEG	POS	POS	POS	POS	NEG	NEG	NEG	NEG	NEG	NEG	POS	POS	POS	POS	NEG	NEG	NEG
COL GenBank CP000046.1: Predicted Hybridisation Pattern	NEG	NEG	POS	NEG	POS	AMB	NEG	NEG	NEG	NEG	NEG	NEG	POS	AMB	POS	POS	NEG	NEG	NEG
COL	NEG	NEG	POS	POS	POS	POS	NEG	NEG	NEG	NEG	NEG	NEG	POS	POS	POS	POS	NEG	NEG	NEG
SVH7513 GenBank CP029166.1: Predicted Hybridisation Pattern	NEG	NEG	POS	NEG	POS	NEG	NEG	NEG	NEG	NEG	NEG	NEG	POS	NEG	POS	POS	NEG	NEG	NEG
Uganda-03_643	NEG	NEG	POS	POS	POS	AMB	NEG	NEG	NEG	NEG	NEG	NEG	POS	POS	POS	POS	NEG	NEG	NEG
Uganda-10_8347	NEG	NEG	POS	NEG	POS	NEG	NEG	NEG	NEG	NEG	NEG	NEG	POS	POS	POS	POS	NEG	NEG	NEG
Uganda-13_10674	NEG	NEG	POS	NEG	POS	NEG	NEG	NEG	NEG	NEG	NEG	NEG	POS	POS	NEG	POS	NEG	NEG	NEG
Uganda-21_12661	NEG	NEG	POS	NEG	POS	NEG	NEG	NEG	NEG	NEG	NEG	NEG	POS	POS	POS	POS	NEG	NEG	NEG
Uganda-23_12696	NEG	NEG	POS	NEG	POS	NEG	NEG	NEG	NEG	NEG	NEG	NEG	POS	POS	POS	POS	NEG	NEG	NEG
**>ST(43-3-1-1-4-4-3)**	**POS**	**NEG**	**POS**	**MB / VA**	**NEG**	**NEG**	**POS**	**NEG**	**NEG**	**NEG**	**NEG**	**NEG**	**POS**	**NEG**	**MB / VA**	**NEG**	**POS**	**MB / VA**	**NEG**
RGB-095930: Predicted Hybridisation Pattern	POS	NEG	POS	NEG	NEG	NEG	POS	NEG	NEG	NEG	NEG	NEG	POS	NEG	POS	NEG	POS	NEG	NEG
RGB-095930	POS	NEG	POS	POS	NEG	NEG	POS	NEG	NEG	NEG	NEG	NEG	POS	NEG	NEG	NEG	POS	POS	NEG
ST6610_id-36080 (MLST Database): Predicted Hybr. Pattern	POS	NEG	POS	NEG	NEG	NEG	POS	NEG	NEG	NEG	NEG	NEG	POS	NEG	POS	NEG	POS	AMB	NEG
ST6610_id-36082 (MLST Database): Predicted Hybr. Pattern	POS	NEG	POS	NEG	NEG	NEG	POS	NEG	NEG	NEG	NEG	NEG	POS	NEG	POS	NEG	POS	AMB	NEG
**>CC140**	**POS**	**NEG**	**POS**	**MB / VA**	**NEG**	**NEG**	**POS**	**NEG**	**NEG**	**NEG**	**NEG**	**NEG**	**POS**	**NEG**	**MB / VA**	**NEG**	**POS**	**MB / VA**	**NEG**
Kenyaseq6547225, ERR1764920: Predicted Hybridisation Pattern	POS	NEG	POS	NEG	NEG	NEG	POS	NEG	NEG	NEG	NEG	NEG	POS	NEG	POS	NEG	POS	AMB	NEG
SO-1977, SRR5682128: Predicted Hybridisation Pattern	POS	NEG	POS	NEG	NEG	NEG	POS	NEG	NEG	NEG	NEG	NEG	POS	NEG	POS	NEG	POS	AMB	NEG
Uganda-51_23201-2	POS	NEG	POS	POS	NEG	NEG	POS	NEG	NEG	NEG	NEG	NEG	POS	NEG	NEG	NEG	POS	POS	NEG
