# Supplementary material for: Characterisation and Molecular Analysis of an Unusual Chimeric Methicillin Resistant Staphylococcus Aureus Strain and its Bacteriophages
Source: Front Genet. 2021 Nov 18;12:723958. doi: 10.3389/fgene.2021.723958 (PMC8638950; doi:10.3389/fgene.2021.723958)
Supplement: Supplementary file 8 [file DataSheet5.PDF]

Number/ Position	Gene	RGB-095930 compared to ERR1764920	RGB-095930 compared to NCTC8325
1	dnaA	0.00	0.73
2	dnaN	0.00	0.71
3	yaaA	0.00	0.00
4	recF	0.00	0.09
5	gyrB	0.00	0.31
6	gyrA	0.04	1.57
7	nnrD	0.00	0.60
8	hutH	0.00	1.32
9	serS	0.00	0.39
10	azlC	0.00	3.58
11	azlD	0.00	0.91
12	metX	0.00	3.51
13	yybS	0.11	1.49
14	gdpP	0.00	1.27
15	rplI	0.00	2.21
16	dnaC	0.00	1.07
17	purA	0.00	0.62
18	walR	0.00	0.57
19	walK	0.00	0.99
20	walH	0.00	1.87
21	walI	0.00	4.56
22	walJ	0.00	1.37
23	sasH	0.00	3.88
24	orfX	0.00	2.92
25	C2G7A0	0.00	100.00
26	Q6GKL3	0.00	100.00
27	C2G798	0.00	100.00
28	D2N3E0	0.00	100.00
29	Q6GKL1	0.00	100.00
30	cstR-GI	0.00	100.00
31	cstA-GI	0.00	100.00
32	cstB-GI	0.00	100.00
33	sqr	0.00	100.00
34	dusC	0.00	2.03
35	A6TXM6	0.00	9.31
36	A6QD71	0.00	9.43
37	Q5HJT2	0.00	100.00
38	Q6GD34	0.00	100.00
39	A6QD75	0.00	100.00
40	A6QD76	0.00	100.00
41	A8YZ18	0.00	100.00
42	Q6GKK6	0.16	2.94
43	Q7A890	0.00	100.00
44	Q2YUT2	0.00	100.00
45	plc	0.00	3.34
46	Q8NYT6	0.00	3.31
47	Q8NYT5	0.08	2.63
48	norC	0.00	3.24
49	nptA	0.00	1.02
50	Q2YUS5	0.00	3.32
51	DUF1648	0.00	1.27
52	lctP-locus1	0.00	0.44
53	spa	0.00	7.37
54	sarS	0.00	0.80
55	sirC	0.00	6.21
56	sirB	0.00	3.41
57	sirA	0.00	0.40
58	sbnA-cysK1	0.00	0.71
59	sbnB	0.00	1.38
60	sbnC	0.00	1.94
61	sbnD	0.00	1.11
62	sbnE	0.00	1.55
63	sbnF	0.00	1.21
64	sbnG	0.00	0.90
65	sbnH	0.00	1.41
66	sbnI	0.00	0.39
67	Q5HJP3	0.00	2.07
68	butA	0.00	0.90
69	galE	0.00	7.30
70	wcaJ-tuaA	0.00	5.19
71	epsF	0.09	2.23
72	wzy	0.00	2.10
73	wzx	0.00	1.61
74	sodA-L1	0.00	0.83
75	sasD	0.00	1.10
76	Q5HJN3	0.00	1.72
77	deoD-L1	0.00	1.27
78	tet38	0.07	1.48
79	deoC-L1	0.00	1.36
80	deoB	0.00	0.76
81	phnE2	0.00	0.12
82	phnE1	0.00	0.37
83	phnC	0.00	1.29
84	phnB	0.10	1.15
85	Q5HJM4	0.00	0.84
86	cpdB	0.00	1.37
87	Q1Y4B9	0.00	81.80
88	adhE	0.00	0.73
89	capA-L1	0.00	0.90
90	capB-L1	0.00	6.55
91	capC-L1	0.00	1.57
92	capD	0.00	0.55
93	capE	0.00	0.97
94	capF	0.00	1.89
95	capG	0.00	0.89
96	capH5	0.00	0.80
97	capI5	0.00	1.35
98	capJ5	0.00	1.03
99	capK5	0.00	0.83
100	capL	0.00	1.41
101	capM	0.00	0.54
102	capN	0.00	2.03
103	capO	0.08	1.11
104	capP	0.00	1.45
105	isdI	0.00	0.92
106	ybaN	0.00	0.78
107	aldA1	0.00	0.87
108	czcD	0.00	2.71
109	Q5HJK0-srpF	0.00	0.78
110	tauB	0.00	3.23
111	tauA	0.00	2.26
112	tauC	0.00	1.31
113	Q5HJJ8	0.00	3.10
114	lmrP	0.00	1.36
115	ausA	0.00	1.39
116	ausB	0.00	0.78
117	Q5HJJ2	0.00	0.40
118	argB	0.00	5.45
119	argJ	0.08	3.62
120	argC	0.00	3.38
121	rocD1	0.08	2.28
122	brnQ1	0.00	1.25
123	ywoC2	0.00	9.04
124	ipdC	0.00	2.07
125	glcA-ptsG	0.00	1.42
126	DUF871	0.00	0.76
127	murQ	0.00	0.67
128	Q5HJI0	0.00	1.03
129	rpiRB	0.00	0.34
130	hsdR	0.00	0.75
131	Q9RL82	0.00	8.01
132	oppF2	0.06	2.01
133	oppB2	0.00	1.42
134	oppC2	0.00	1.89
135	oppA2	0.00	1.41
136	ggt	0.00	5.28
137	acpD	0.00	1.75
138	Q5HJG7	0.00	1.55
139	malK-ugpC	0.00	0.55
140	malE	0.00	0.71
141	malC	0.00	1.73
142	malD	0.00	0.24
143	yrbE	0.00	1.86
144	mviM	0.00	1.63

Number/ Position	Gene	RGB-095930 compared to ERR1764920	RGB-095930 compared to NCTC8325
145	iolE	0.00	1.03
146	Q7A1X1	0.00	0.00
147	uhpT	0.00	1.38
148	yesN	0.00	1.05
149	yesM	0.00	1.22
150	hptA	0.00	1.44
151	pflB	0.00	0.44
152	pflA	0.00	0.26
153	Q2YV51	0.00	7.12
154	glpQ1	0.00	1.42
155	Q2YV49	0.00	1.74
156	coa	4.17	24.15
157	fadA	0.00	1.35
158	fadB	0.04	1.24
159	fadD	0.08	0.74
160	fadE	0.00	2.19
161	fadX	0.06	1.73
162	prsW-prsS	0.00	1.22
163	nikA	0.00	1.29
164	DUF488	0.00	5.32
165	Q5HJD9	0.00	9.77
166	hmp	0.00	2.62
167	lctE	0.00	0.42
168	ptsIIBC	0.07	0.92
169	rihA	0.00	0.64
170	bglG	0.00	100.00
171	Q5HJD2	0.00	100.00
172	Q5HJD1-gatB1	0.00	100.00
173	Q2G2C8-gatC1	0.00	100.00
174	gutB	0.00	100.00
175	Q5HJC7	0.00	100.00
176	Q5HJC6	0.00	100.00
177	tarI1	0.00	100.00
178	tarJ1	0.00	100.00
179	tarL1	0.00	100.00
180	tarF	0.00	35.12
181	tarJ2	0.00	100.00
182	tarL2	0.00	100.00
183	tarS	0.00	100.00
184	lytS	0.00	0.34
185	lytR	0.00	0.54
186	lrgA	0.00	0.00
187	lrgB	0.00	0.57
188	Q5HJB2	0.43	0.57
189	Q1YAD5-ptsG3	17.30	0.00
190	bglA	0.90	0.00
191	rbsK	1.53	0.00
192	rbsD	1.73	0.00
193	rbsU	1.36	0.23
194	rbsR	2.00	0.00
195	yusP	2.83	0.22
196	yxeI	0.60	0.10
197	lytM	0.95	0.11
198	Q5HJ98-ybhF	1.67	0.00
199	Q5HJ97	0.65	0.00
200	Q5HJ96	0.69	0.00
201	Q5HJ95	1.25	0.00
202	ssaA	0.89	0.00
203	esxA	0.00	0.00
204	esaA	0.96	0.00
205	essA	0.22	0.00
206	esaB	0.00	0.00
207	essB	0.82	0.00
208	essC	3.85	0.04
209	esxB	100.00	0.00
210	Q1Y4S0	0.53	0.00
211	Q2G173	0.50	0.00
212	focA-L1-nirC	1.94	0.00
213	brnQ2	2.75	0.00
214	Q99WS0-sapS	1.12	0.00
215	Q2G168	1.43	0.00
216	A5IPH6	0.44	0.00
217	pfoR	0.68	0.00
218	Q5HJ57	0.63	0.00
219	psuG	1.52	0.00
220	nupC2	4.42	0.00
221	nanT	3.07	0.00
222	nanA	1.47	0.00
223	nanK	1.51	0.00
224	nanR	0.25	0.00
225	nanE	1.20	0.15
226	Q5HJ49	1.60	0.00
227	lip2	2.60	0.00
228	A5IPQ2	6.16	0.00
229	Q6GJZ4	4.20	0.00
230	limB1	1.50	0.20
231	gcvH1	0.60	0.00
232	UPF0189-ymdB	1.62	0.12
233	sir2	2.12	0.00
234	lplA1	3.42	0.00
235	ulaA	1.63	0.00
236	Q5HIW4	0.70	0.00
237	Q5HIW3	0.23	0.00
238	A8YZF7-licR2	1.43	0.05
239	mepR	1.67	0.00
240	mepA	1.47	0.00
241	mepB	10.43	0.00
242	glpT	0.81	0.15
243	mhqA-1	0.93	0.00
244	ssuD-luxA	1.79	0.00
245	ssuE	1.41	0.00
246	yeiH	1.81	0.00
247	rimL-ydaF	4.58	0.00
248	fepA	0.82	0.00
249	fepB	4.39	0.00
250	fepC	4.73	0.00
251	tatC	5.70	0.00
252	tatA	1.85	0.00
253	DUF1398	8.97	0.00
254	Q5HIU5	6.86	0.00
255	DUF3169	4.48	0.00
256	Q5HIU3	4.15	0.00
257	Q5HIU2	1.75	0.16
258	ltrA-L1	3.00	0.18
259	thlA-yqil	1.35	0.08
260	mdh	1.06	0.00
261	metE	1.03	0.00
262	metF	1.30	0.00
263	metC	2.24	0.00
264	metI	2.08	0.00
265	spo0J	2.36	0.00
266	ykuT	8.84	0.00
267	yyzM	5.39	0.00
268	yyaF	5.01	0.00
269	Q5HIT0	1.04	0.00
270	rpsF	0.34	0.00
271	ssb	0.20	0.00
272	rpsR	0.00	0.00
273	setC	49.67	0.00
274	Q2G106	2.17	0.00
275	Q1Y9M1	0.52	0.00
276	Q5HIS2	1.14	0.00
277	Q5HIS1	3.17	0.00
278	Q7A7J4	2.06	0.00
279	Q5HIR9	2.60	0.00
280	A5IPV8	0.48	0.00
281	Q6GJR9	85.83	0.00
282	ahpF	1.57	0.07
283	ahpC	0.18	0.00
284	nfrA	2.51	0.00
285	tcyP	2.09	0.14
286	Q5HIR2	2.09	0.00
287	Q5HIR1	0.90	0.15
288	yflT	3.68	0.00

Number/ Position	Gene	RGB-095930 compared to ERR1764920	RGB-095930 compared to NCTC8325
289	xpt	1.04	0.00
290	pbuX	0.79	0.00
291	guaB	1.16	0.07
292	guaA	1.82	0.00
293	Q2YVN4	3.08	0.51
294	Q5HIQ1	5.22	0.00
295	Q2G0Y0	2.48	0.00
296	ssl01	20.12	0.00
297	ssl02	11.82	0.00
298	ssl03	25.70	9.00
299	ssl04	72.13	65.93
300	ssl05	12.62	0.00
301	ssl06	100.00	0.00
302	ssl07	12.21	0.00
303	ssl08	100.00	0.00
304	ssl09	9.87	0.14
305	ssl10	100.00	0.00
306	hsdM-ssl	4.62	0.00
307	hsdS-ssl	81.44	18.56
308	ssl11	100.00	0.00
309	Q5HIM7	2.05	0.00
310	cobW1	1.75	0.00
311	psmA4	0.00	0.00
312	psmA3	0.00	0.00
313	psmA2	0.00	0.00
314	mpsA-nuoL	1.75	0.00
315	mpsB-ybcC	2.85	0.00
316	mpsC-ybcI	1.10	0.00
317	Q5HIM2	1.44	0.00
318	Q5HIM1	1.33	0.00
319	Q5HIM0-est1	1.36	0.00
320	Q5HIL8	1.27	0.07
321	mccA-var915	4.26	0.11
322	mccB	1.57	0.09
323	metN2	2.53	0.00
324	metP2	1.97	0.15
325	metQ2	2.73	0.00
326	aaa	3.78	0.00
327	Q5HIL1	0.74	0.00
328	nudG	2.02	0.00
329	bltD	1.65	0.00
330	yibF	1.53	0.00
331	yibE	1.71	0.00
332	gltC	2.71	0.00
333	gltB	0.69	0.02
334	gltD	1.02	0.07
335	treP	1.12	0.00
336	treC	1.58	0.12
337	treR	1.23	0.00
338	Q5HIK0	1.90	0.00
339	dnaX	1.47	0.00
340	yaaK	0.31	0.00
341	recR	1.01	0.00
342	yaaO	0.82	0.00
343	tmk	1.29	0.00
344	darA	0.30	0.00
345	holB	0.32	0.00
346	yaaT-tpl	0.87	0.00
347	yabA	0.00	0.00
348	yabB	0.96	0.00
349	yazA	0.40	0.40
350	rsmI	1.07	0.00
351	metS	0.81	0.00
352	tatP	0.78	0.00
353	rnmV	0.19	0.00
354	ksgA-rsmA	0.79	0.00
355	veg	0.00	0.00
356	ispE	0.94	0.00
357	purR	0.24	0.00
358	yabJ	1.57	0.00
359	spoVG	0.33	0.00
360	glmU	1.92	0.07
361	prs	0.41	0.00
362	rplY	0.31	0.00
363	pth	0.35	0.00
364	mfd	0.88	0.00
365	yabM	0.98	0.00
366	mazG	1.17	0.08
367	hslR	0.76	0.00
368	divIC	0.25	0.00
369	yabR	0.50	0.00
370	tilS	1.47	0.08
371	hpt	0.19	0.00
372	ftsH	0.29	0.05
373	hslO	0.45	0.00
374	cysK	0.75	0.00
375	folP	4.85	0.25
376	folB	1.64	0.00
377	folK	1.47	0.00
378	lysS_leader	3.23	0.00
379	lysS	0.54	0.00
380	pdxR	1.59	0.00
381	pdxS	0.68	0.00
382	pdxT	0.71	0.00
383	nupC1	0.82	0.08
384	ctsR	0.65	0.00
385	mcsA	0.88	0.18
386	mcsB	0.49	0.00
387	clpC	0.73	0.08
388	radA	0.95	0.00
389	pilT	1.68	0.00
390	gltX	0.96	0.00
391	cysE	0.31	0.00
392	cysS	1.36	0.00
393	mrnC	0.99	0.00
394	yacO	1.07	0.00
395	yacP	0.38	0.00
396	sigH	1.05	0.00
397	rpmG3	0.69	0.00
398	secE	0.55	0.55
399	nusG	0.00	0.00
400	rplK	0.00	0.00
401	rplA	0.43	0.00
402	rplJ	0.00	0.00
403	rplL	0.27	0.00
404	ybxB-rsmC	0.99	0.16
405	rpoB	0.59	0.00
406	rpoC	0.86	0.00
407	ybxF	0.39	0.00
408	rpsL	0.00	0.00
409	rpsG	0.85	0.21
410	efg	0.34	0.00
411	tuf	0.17	0.00
412	yhaA-amaA	3.49	0.00
413	kbl	1.60	0.00
414	hchA	1.37	0.00
415	araB	0.79	0.00
416	Q5HIC2	0.83	0.00
417	ilvE	5.20	0.09
418	ppaX1	0.58	0.00
419	dck	100.00	0.00
420	dgk	0.32	0.00
421	tadA	1.70	0.00
422	A5IQB3	0.80	0.00
423	azo1	5.47	0.00
424	sdrC	15.27	4.83
425	sdrD	84.74	73.78
426	bbp	74.51	38.61
427	sdgA	2.44	0.07
428	sdgB	1.74	0.00
429	folE2	0.91	0.00
430	bshB2	1.05	0.15
431	yojF	0.55	0.00
432	nagB	1.05	0.13

Number/ Position	Gene	RGB-095930 compared to ERR1764920	RGB-095930 compared to NCTC8325
433	hxlA	0.47	0.16
434	sis	0.91	0.00
435	Q2FJ68	4.01	0.00
436	proP	3.85	0.07
437	vraA	1.31	0.00
438	vraB	1.67	0.09
439	vraC	1.37	0.00
440	Q5HI98	2.59	0.37
441	thiD1	1.32	0.00
442	ung	0.61	0.00
443	ywdI	1.84	0.00
444	ywdK	0.54	0.00
445	yfnA2	0.81	0.07
446	DUF3815	5.30	0.00
447	DUF1212_L1	0.78	0.00
448	hemQ	0.27	0.00
449	pta	0.71	0.10
450	lipL	1.31	0.00
451	mvaK1	1.19	0.11
452	mvaD	1.63	0.10
453	mvaK2	0.56	0.00
454	DUF1450	100.00	0.00
455	ykgC	1.81	0.08
456	Q5HI81	0.68	0.00
457	Q5HI79	9.27	0.07
458	Q5HI67	2.02	0.00
459	Q5HI66	11.36	0.00
460	Q8NXU4	0.84	0.00
461	Q5HI65	4.55	0.00
462	ywhD	0.77	0.00
463	adhA	0.59	0.10
464	ywiB	0.93	0.00
465	argS	1.02	0.06
466	nth2	3.62	0.00
467	A5IQG3	0.90	0.00
468	yvrB	1.68	0.00
469	ppaX2	0.56	0.00
470	ydjP	1.12	0.00
471	Q5HI54	0.39	0.00
472	Q5HI53	1.53	0.00
473	Q5HI52	1.39	0.00
474	sarA	0.00	0.00
475	Q2YSV8	100.00	0.00
476	A6QET1	8.00	0.00
477	Q7A1N3	12.25	0.00
478	A5IQH4	17.29	0.18
479	mrpA	10.15	0.00
480	mrpB	10.80	0.23
481	mrpC	9.28	0.00
482	mrpD	6.41	0.00
483	mrpE	1.66	0.00
484	mrpF	4.95	0.00
485	mrpG	2.05	0.23
486	nhaK1	0.54	0.00
487	mntC	0.32	0.96
488	mntB	0.24	0.00
489	mntA	0.00	0.00
490	mntR	1.24	0.00
491	Q5HI33	0.40	0.00
492	tarA	0.78	0.00
493	tarH	0.63	0.00
494	tarG	0.36	0.00
495	tarB	1.36	0.09
496	tarX	1.98	0.00
497	tarD	1.00	0.00
498	pbpD	1.00	0.00
499	msbA1	1.27	0.00
500	nupG	0.98	0.08
501	yxkD	0.72	0.00
502	fhuC	0.75	0.00
503	fhuB	0.70	0.00
504	fhuG	1.28	0.00

Number/ Position	Gene	RGB-095930 compared to ERR1764920	RGB-095930 compared to NCTC8325
505	dakK	3.82	0.00
506	dakL	2.56	0.00
507	dakP	2.20	0.00
508	Q5HI15	0.40	0.00
509	Q5HI14	2.72	0.00
510	lip4	3.26	0.19
511	Q5HI12	0.47	0.00
512	Q5HI11	0.79	0.00
513	graX	0.87	0.00
514	graR	4.89	0.00
515	graS	13.16	0.00
516	vraF	10.89	0.00
517	vraG	15.77	0.00
518	ykaA-pitR	1.29	0.00
519	pitA	0.89	0.00
520	ssaA5	0.63	0.00
521	yetJ	0.93	0.16
522	rbf	4.79	0.00
523	sarX	0.70	0.00
524	yeeI	3.35	0.00
525	DUF985	1.48	0.00
526	A5IQM5	1.55	0.00
527	ccpE	1.73	0.00
528	A8Z196	1.15	0.08
529	Q2YSN2	13.09	0.00
530	Q2YSQ6	1.16	0.14
531	A5IQN0	0.90	0.00
532	A8YZU8	7.07	0.00
533	Q5HHZ0	2.00	0.00
534	ykkB	0.92	0.00
535	yvdD	1.41	0.00
536	yqxD	0.87	0.00
537	Q5HHY6	0.44	0.00
538	uppP	2.40	0.00
539	cydD	1.16	0.00
540	cydC	1.31	0.00
541	mgrA	0.45	0.00
542	cobW2	1.29	0.00
543	ycsN	0.88	0.00
544	Q5HHX9	1.04	0.00
545	yflS	1.35	0.13
546	phrB	2.47	0.07
547	Q2G0A5	1.05	0.00
548	Q5HHX6	0.97	0.00
549	Q5HHX5	1.89	0.00
550	norA	8.65	0.00
551	yedL	1.10	0.00
552	ybaK	2.48	0.00
553	fruR	1.31	0.00
554	fruB	1.85	0.00
555	fruA	5.60	0.05
556	nagA	1.95	0.08
557	corC-mpfA	2.15	0.15
558	yvgN1	0.36	0.00
559	csbB	1.83	0.00
560	saeS	0.47	0.00
561	saeR	0.00	0.00
562	saeQ	1.05	0.00
563	saeP	3.17	0.00
564	Q5HHW1	0.85	0.00
565	queE	0.98	0.00
566	queD	0.95	0.00
567	queC	0.30	0.00
568	pabA	0.67	0.00
569	pabB	1.30	0.00
570	pabC	1.31	0.00
571	Q6GIS6	2.90	0.00
572	ahs1	0.56	0.14
573	ahs2	1.00	0.00
574	ltaS	0.46	0.00
575	uup	1.38	0.05
576	recQ1	1.91	0.06

Number/ Position	Gene	RGB-095930 compared to ERR1764920	RGB-095930 compared to NCTC8325
577	opuBA	0.61	0.00
578	opuBB	1.52	0.00
579	hisC2	1.32	0.00
580	yorS	1.66	0.18
581	bmrU-dgkA1	1.85	0.00
582	dtpT	1.53	0.00
583	queF	1.20	0.00
584	yxxF	100.00	0.00
585	nrdI	0.50	0.00
586	nrdE	1.80	0.00
587	nrdF	2.67	0.00
588	sstA	10.08	0.00
589	sstB	4.28	0.10
590	sstC	8.40	0.00
591	sstD	7.39	0.00
592	Q5HHT3	3.17	0.00
593	murB	1.73	0.00
594	grpB	3.08	0.00
595	Q5HHT0	0.46	0.00
596	ytxJ	0.62	0.00
597	glxK2	1.24	0.00
598	pepT	1.30	0.00
599	Q5HHS6	0.81	0.00
600	DUF1212_L2	1.05	0.00
601	gdpS	0.75	0.00
602	tarO	1.42	0.00
603	Q5HHS2	0.78	0.00
604	degV1	1.04	55.71
605	comFA	2.12	0.09
606	comFC	1.33	0.00
607	yfiA	0.00	0.00
608	secA1	1.46	0.00
609	prfB	0.99	0.00
610	lysM	0.83	0.00
611	yfbR	0.61	0.00
612	DUF2198	0.84	0.00
613	uvrB	1.31	49.05
614	uvrA	1.55	0.00
615	hpr	0.64	0.00
616	lgt	1.19	0.12
617	yvoF	1.03	0.00
618	yvcD	1.32	0.07
619	trxB	0.75	0.00
620	yvcJ	0.77	0.00
621	mgfK-yvcK	0.80	0.00
622	whiA	1.06	0.00
623	clpP	1.19	0.00
624	yfcH	1.33	0.00
625	Q5HHP8	0.95	0.00
626	gapR	0.59	0.10
627	gapA	0.30	0.00
628	pgk	0.59	0.00
629	tpi	1.18	0.26
630	gpmI	1.58	0.07
631	eno	1.07	0.00
632	Q2YSE7	0.44	0.00
633	secG	0.00	0.00
634	est	7.29	0.00
635	rnr	4.34	0.04
636	ssrP	3.01	0.00
637	Q1YB79	0.27	0.00
638	Q5HHN0	2.68	0.00
639	Q1XZI1	1.13	0.00
640	clfA	13.87	1.61
641	vwb	24.27	0.06
642	emp	15.84	0.19
643	vwb2	100.00	0.00
644	nuc1	1.46	0.00
645	cspC-L1	0.00	0.00
646	Q1Y1Z8	4.11	0.00
647	Q2G007	1.40	0.00
648	Q1YB69	1.23	0.00

Number/ Position	Gene	RGB-095930 compared to ERR1764920	RGB-095930 compared to NCTC8325
649	Q6GIJ6	0.53	0.00
650	Q6GIJ5	0.38	0.00
651	DUF1250	0.49	0.00
652	Q6GIJ3	0.84	0.00
653	gpmA1-cobC	1.03	0.17
654	yisU1	1.78	0.00
655	Q5HHL4	0.40	0.00
656	ohrB	1.42	0.00
657	aroD	0.98	0.00
658	ntrA	0.74	0.19
659	trxA2	0.62	0.00
660	yusI	1.12	0.00
661	gcvH2	2.10	0.00
662	ywqG	1.48	0.00
663	yusF	1.03	0.00
664	yusE	1.01	0.00
665	metN1	0.88	0.00
666	metP1	1.15	0.00
667	metQ1	1.22	0.00
668	csbD-L1	0.51	0.51
669	DUF368	0.82	0.12
670	sufC	0.52	0.00
671	sufD	0.46	0.00
672	sufS	1.20	0.08
673	sufN	2.80	2.58
674	sufB	0.72	0.00
675	Q2YWM5	1.90	0.00
676	corB	0.77	0.00
677	npd	2.53	0.00
678	yunF	0.71	0.12
679	yunE	1.81	0.12
680	yunD	1.29	0.23
681	lipA	0.87	0.00
682	DUF1027	0.25	0.00
683	DUF3055	0.00	0.00
684	yutE	1.15	0.23
685	yutF	2.56	0.00
686	gyaR	1.56	0.00
687	dltX	0.00	0.00
688	dltA	0.75	0.00
689	dltB	0.41	0.00
690	dltC	0.00	0.00
691	dltD	1.02	0.00
692	nfuA	0.45	0.00
693	yuzD	100.00	0.00
694	yutJ-ndhF	1.22	0.09
695	yuzB	0.42	0.42
696	sufA	0.83	0.56
697	ndhC-yumB	0.17	0.08
698	pepZ	1.15	0.00
699	nhaC3	0.99	0.00
700	yuxO	1.33	0.00
701	Q5HHE0	0.95	0.09
702	mnhG	1.12	0.00
703	mnhF	0.68	0.00
704	mnhE	0.83	0.00
705	mnhD	0.87	0.00
706	mnhC	0.58	0.00
707	mnhB	0.23	0.00
708	mnhA	0.42	0.00
709	kapB	1.04	0.00
710	prsA1	1.35	0.00
711	yugI	0.53	0.00
712	namA	1.33	0.18
713	rocD2	1.26	0.00
714	gluD	0.48	0.00
715	glpQ2	0.65	0.00
716	argH	1.16	0.00
717	argG	1.41	0.00
718	pgi	0.53	0.08
719	yhjE	1.91	0.00
720	spsA	3.24	0.00

Number/ Position	Gene	RGB-095930 compared to ERR1764920	RGB-095930 compared to NCTC8325
721	spsB	2.26	0.00
722	rexB	1.41	0.00
723	rexA	0.74	0.03
724	Q5HHB6	0.44	0.00
725	UPF0344	4.87	0.00
726	cdr	0.53	0.08
727	yitU-yidA	1.45	0.00
728	sufT-paaD	0.65	0.00
729	Q5HHB1	0.94	0.17
730	clpB	0.65	0.04
731	Q5HHA9	1.03	0.00
732	Q5HHA5	2.33	0.00
733	eapH-2	0.00	0.00
734	A6U088	0.00	0.00
735	fabH	0.96	0.00
736	fabF	0.88	0.00
737	Q2YWV0	1.61	0.00
738	oppB	1.08	0.00
739	oppC	0.37	0.00
740	oppD	0.83	0.00
741	oppF	5.10	0.00
742	oppA	2.36	0.00
743	appA	2.62	0.17
744	appD	1.93	0.10
745	appF	2.04	0.00
746	appB	1.14	0.00
747	appC	0.79	0.00
748	trpS	4.65	0.00
749	spxA	0.00	0.00
750	trfA	0.83	0.00
751	trfB	99.90	0.10
752	pepF1	0.83	0.00
753	yjbH	0.50	0.00
754	yjbI	0.55	0.00
755	yjbK	0.84	0.00
756	yjbL	0.57	0.00
757	relQ	0.47	0.00
758	ppnK	0.74	0.00
759	rluE	1.75	0.00
760	mgtE	1.59	0.00
761	cpaA	0.92	0.00
762	fabI	1.17	0.13
763	UPF0118	0.46	0.00
764	yrbD	1.27	0.06
765	yjcH	2.11	0.00
766	UPF0477	0.59	0.00
767	ltaA	0.25	0.00
768	ugtP	0.17	0.00
769	murE	0.13	0.00
770	yueH	100.00	0.00
771	prfC	0.96	0.00
772	terC	0.75	0.00
773	htrA	1.77	0.04
774	ktrD	0.88	0.00
775	Q1XY52-yfkN3	1.19	0.00
776	comK	0.35	0.00
777	Q6GI58	1.32	0.00
778	lplA2	1.42	0.00
779	Q6GI56	0.00	0.00
780	Q5HH56	0.17	0.00
781	txpA-var1	4.63	0.00
782	Q7A194	3.37	0.00
783	yujE	100.00	0.00
784	yxeA	0.31	0.00
785	yujD	1.71	0.00
786	Q6GI49	3.44	0.00
787	Q6GI46	3.64	0.00
788	Q5HH47	3.54	0.00
789	shpA	2.56	0.00
790	Q7A191	1.85	0.00
791	Q6GAH2	1.81	0.00
792	menA	0.75	0.11

Number/ Position	Gene	RGB-095930 compared to ERR1764920	RGB-095930 compared to NCTC8325
793	menF	1.54	0.00
794	menD	3.76	0.00
795	menH	3.48	0.00
796	menB	0.49	0.12
797	sspC	0.61	0.00
798	sspB	3.21	0.00
799	sspA	100.00	0.00
800	aspC	0.95	0.09
801	ykrP	1.29	0.00
802	Q5HH32	0.95	0.48
803	atl	3.33	0.05
804	UPF0039	5.75	0.00
805	Q5HH29	2.76	0.00
806	tagV-lcpB	8.64	0.08
807	fmtA	12.40	0.17
808	qoxD	1.03	0.00
809	qoxC	0.00	0.00
810	qoxB	0.35	0.00
811	qoxA	0.36	0.00
812	iraE	0.00	0.00
813	folD	1.39	0.12
814	purE	1.04	0.00
815	purK	1.33	0.00
816	purC	4.40	0.00
817	purS	1.14	0.00
818	purQ	0.74	0.00
819	purL	2.88	0.00
820	purF	4.38	0.00
821	purM	1.07	0.00
822	purN	0.71	0.00
823	purH	1.01	0.00
824	purD	1.60	0.00
825	ykoC	1.61	0.00
826	ykoD	1.00	0.07
827	ykoE	1.22	0.17
828	graF	1.48	0.00
829	Q5HH05	1.62	0.00
830	ywbD	1.88	0.00
831	Q5HH03	3.50	0.18
832	ptsH	1.87	0.00
833	ptsI	1.11	0.00
834	nrdH	1.28	0.00
835	cydA	0.59	0.00
836	cydB	1.18	0.00
837	ktrA	0.45	0.00
838	rnjA	0.88	0.00
839	rnpZA	0.00	0.00
840	defB-def1	1.63	0.18
841	ykyA	0.80	0.16
842	pdhA	0.45	0.00
843	pdhB	1.02	0.00
844	pdhC	0.54	0.08
845	pdhD	0.21	0.07
846	UPF0223	0.72	0.00
847	puuR	0.74	0.00
848	potA	100.00	0.00
849	potB	1.50	0.00
850	potC	0.99	0.12
851	potD	1.02	0.00
852	Q5HGY1	0.49	0.00
853	mntH	0.96	0.00
854	yktB	0.49	0.00
855	suhB1	1.45	0.00
856	ylaF	0.52	0.00
857	typA	0.76	0.05
858	ylaL	0.62	0.00
859	ylaN	0.00	0.00
860	ftsW2	1.06	0.00
861	pycA	1.36	0.03
862	ctaA	0.88	0.00
863	ctaB	0.33	0.22
864	ctaM-yozB	1.08	0.00

Number/ Position	Gene	RGB-095930 compared to ERR1764920	RGB-095930 compared to NCTC8325
865	ylbC2	1.00	0.11
866	ylbF	1.61	0.00
867	yhdW	4.75	0.00
868	ylbG	0.39	0.39
869	Q5HGW1	1.03	0.00
870	rsmD	1.10	0.00
871	coaD	0.41	0.00
872	ylbM	1.84	0.00
873	ylbN-Q4L5E0	0.72	0.00
874	rpmF	0.57	0.57
875	isdB	3.50	0.00
876	isdA	5.63	0.00
877	isdC	3.36	0.15
878	isdD	1.58	0.00
879	isdE	0.34	0.00
880	isdF	1.24	0.10
881	srtB	0.95	0.00
882	isdG	0.31	0.00
883	spoU	1.08	0.00
884	pheS	0.76	0.09
885	pheT	1.83	0.00
886	rnhC	2.24	0.00
887	zapA	0.37	0.75
888	yshB	0.57	0.00
889	polX	1.11	0.00
890	mutS2	1.70	0.00
891	trxA1	1.59	0.00
892	uvrC	1.46	0.00
893	sdhC	0.81	0.00
894	sdhA	1.13	0.06
895	sdhB	0.49	0.00
896	murI	100.00	100.00
897	ntpA	3.40	0.00
898	ysnB	9.13	0.00
899	ecb	1.52	0.00
900	flr	100.00	0.00
901	Q2YXB9	1.78	0.00
902	efb	11.45	0.00
903	scc	15.67	0.00
904	A5IS45	14.52	0.00
905	Q6GHS5	15.26	0.00
906	A5IS47	16.24	0.00
907	hla	4.79	0.00
908	A6U0Y3	7.48	0.00
909	Q2FZB4	15.92	0.00
910	setB3	18.83	0.00
911	setB2	21.21	0.00
912	setB1	12.81	0.00
913	arcB-L1	1.90	0.00
914	arcC-L1	1.18	0.00
915	arcD-L1	1.41	0.00
916	A5IS57	1.75	0.00
917	per	2.53	0.11
918	psmB1	1.48	0.00
919	Q1Y2B3	1.29	0.00
920	Q931T2	0.45	0.00
921	bshC	1.36	0.49
922	mraZ	0.23	0.00
923	mraW-rsmH	0.32	0.00
924	ftsL	0.00	0.00
925	pbpA	1.07	0.04
926	mraY	1.14	0.00
927	murD	1.11	0.00
928	ftsQ	0.91	0.00
929	ftsA	0.64	0.00
930	ftsZ	0.09	0.00
931	ylmD	1.64	0.00
932	ylmE	0.44	0.00
933	sepF	0.18	0.18
934	ylmG	1.03	0.00
935	ylmH	0.87	0.00
936	divIVA	0.32	0.00

Number/ Position	Gene	RGB-095930 compared to ERR1764920	RGB-095930 compared to NCTC8325
937	ileS	0.69	0.00
938	PF00903-catE	0.63	0.00
939	lspA	0.41	0.20
940	ylyB	0.76	0.00
941	pyrR	0.76	0.00
942	pyrP	1.15	0.00
943	pyrB	0.57	0.00
944	pyrC	0.70	0.16
945	pyrAA	0.36	0.00
946	pyrAB	1.13	0.03
947	pyrF	1.73	0.00
948	pyrE	0.82	0.00
949	Q5HGM6	0.94	0.47
950	Q5HGM5	0.75	0.00
951	fbpA	1.24	0.18
952	gmk	0.48	0.00
953	rpoZ	0.46	0.00
954	coaBC	1.42	0.00
955	priA	0.91	0.00
956	Q5HGL9	100.00	0.10
957	Q5HGL8	1.43	0.00
958	defA-def2	1.23	0.00
959	fmt	1.71	0.00
960	rsmB-sun	1.83	0.15
961	rlmN	0.73	0.00
962	prpC-stp1	0.13	0.00
963	prkC-pknB	1.50	0.00
964	cpgA-engC	2.40	0.11
965	rpe	0.93	0.00
966	thiN	0.62	0.00
967	rpmB	0.00	0.00
968	yloU	0.00	0.00
969	yloV	0.36	0.00
970	recG	1.31	0.00
971	fapR	0.35	0.00
972	plsX	0.51	0.00
973	fabD	1.62	0.11
974	fabG1	0.27	0.00
975	acpP	0.00	0.00
976	rnc	0.68	0.14
977	smc	1.32	0.03
978	ftsY	0.80	0.08
979	ylxM	0.30	0.00
980	ffh	0.15	0.00
981	rpsP	0.00	0.00
982	rimM	0.60	0.40
983	trmD	0.54	0.00
984	rplS	0.57	0.00
985	yfhO	1.23	0.00
986	rbgA	0.00	0.00
987	rnhB	0.52	0.00
988	sucC	0.17	0.00
989	sucD	0.88	0.00
990	dprA	1.26	0.00
991	topA	0.77	0.00
992	trmFO-gid	1.07	0.00
993	xerC	1.00	0.00
994	hslV	0.37	0.18
995	hslU	0.36	0.00
996	codY	0.65	0.00
997	rpsB	0.00	0.13
998	tsf	0.34	0.00
999	pyrH	0.14	0.00
1000	frr	0.90	0.18
1001	uppS	0.00	0.00
1002	cdsA	0.64	0.00
1003	rseP	2.87	0.00
1004	proS	0.94	0.06
1005	polC	0.93	0.00
1006	rimP	0.21	0.00
1007	nusA	1.02	0.00
1008	ylxR	0.70	0.00

Number/ Position	Gene	RGB-095930 compared to ERR1764920	RGB-095930 compared to NCTC8325
1009	ylxQ	100.00	0.00
1010	infB	0.57	0.00
1011	rbfA	0.00	0.00
1012	truB	3.27	0.00
1013	ribF	0.51	0.00
1014	rpsO	0.37	0.00
1015	pnpA	0.81	0.00
1016	rnjB	0.42	0.00
1017	ftsK	0.63	0.00
1018	ymfC	0.84	0.14
1019	ymfF	0.79	0.00
1020	ymfH	0.93	0.08
1021	fabG2	0.71	0.00
1022	ymfK	0.97	0.00
1023	ymfM	0.25	0.00
1024	pgsA	0.00	0.00
1025	cinA	1.56	0.09
1026	recA	0.57	0.00
1027	rny	0.51	0.06
1028	Q5HGE4	0.46	0.00
1029	ymdB	0.63	0.00
1030	porA	0.40	0.00
1031	porB	0.12	0.12
1032	DUF77	0.68	0.00
1033	miaB	1.10	0.00
1034	ymcA	1.37	0.00
1035	thiW	0.61	0.00
1036	mutS	0.95	0.11
1037	mutL	0.85	0.00
1038	glpP	2.43	0.00
1039	glpF	0.73	0.12
1040	glpK	0.67	0.07
1041	glpD	0.99	0.00
1042	pldB2	1.53	0.00
1043	miaA	1.92	0.00
1044	hfq	0.85	0.00
1045	gpxA-L1	1.47	0.00
1046	ynbA	0.89	0.00
1047	ynbB	1.45	0.00
1048	glnR	0.00	0.00
1049	glnA	2.09	0.07
1050	A6U1C8	2.53	0.00
1051	Q2FYY4	100.00	100.00
1052	A5ISJ3	100.00	100.00
1053	Q2YXQ4	1.08	0.00
1054	A6QGL8	1.17	0.00
1055	A5ISJ9	9.60	0.00
1056	cls1	5.94	0.00
1057	Q99UF3	3.70	0.00
1058	nuc2	1.31	0.00
1059	Q5HG96	0.94	0.00
1060	Q5HG95	0.00	0.00
1061	thrA	0.58	0.07
1062	hom	0.70	0.00
1063	thrC	0.85	0.00
1064	thrB	2.51	0.00
1065	yxeH	2.36	0.00
1066	Q5HG88	0.95	0.00
1067	lysP2-Q5HG87	1.44	0.00
1068	katA	1.32	0.07
1069	rpmG2	1.33	0.00
1070	rpsN	0.00	0.00
1071	guaC	0.72	0.10
1072	ylbC1	2.66	0.00
1073	lexA	0.64	0.00
1074	Q5HG79	0.43	0.00
1075	DUF896	0.83	0.00
1076	tkt	1.11	0.00
1077	UPF0154	0.41	0.00
1078	ccdC	0.85	0.00
1079	sbcD	1.87	0.00
1080	sbcC	2.14	0.07

Number/ Position	Gene	RGB-095930 compared to ERR1764920	RGB-095930 compared to NCTC8325
1081	mscL	0.55	0.00
1082	opuD1	0.61	0.00
1083	acnA-citB	1.11	0.04
1084	yneP	0.64	0.00
1085	yneR	1.35	0.34
1086	plsY	0.99	0.00
1087	grlB	1.05	0.05
1088	grlA-parC	0.87	0.08
1089	alsT	2.53	0.07
1090	glcT	1.53	0.00
1091	yubA	0.66	0.00
1092	mprF	9.35	0.00
1093	msrA1	0.59	0.00
1094	lcpA-tagT	0.51	0.00
1095	dmpI	0.00	0.00
1096	uvrX	1.50	0.00
1097	tyrA	1.56	0.09
1098	yhfE	0.97	0.10
1099	trpE	2.84	0.00
1100	trpG	13.68	0.00
1101	trpD	11.31	0.00
1102	trpC	11.24	0.00
1103	trpF	8.65	0.00
1104	trpB	9.14	0.08
1105	trpA	100.00	0.00
1106	femA	0.95	0.00
1107	femB	1.19	0.00
1108	cof	100.00	0.13
1109	Q5HG42	0.42	0.00
1110	nikF	0.43	0.00
1111	nikD	1.29	0.00
1112	nikC	1.08	0.00
1113	nikB	0.71	0.00
1114	Q5HG37	0.87	0.00
1115	pepF2	0.99	0.00
1116	phoU	2.02	0.00
1117	pstB	8.22	0.00
1118	pstA	1.09	0.00
1119	pstC	1.29	0.00
1120	pstS	0.81	0.00
1121	cvfB	1.33	0.00
1122	ykpA	4.12	0.12
1123	lysC	1.24	0.08
1124	asd	4.04	0.00
1125	dapA	3.60	0.11
1126	dapB	2.90	0.14
1127	dapD	2.22	0.14
1128	hipO	0.95	0.00
1129	alr2	1.29	0.09
1130	lysA	2.76	0.00
1131	cspC-L2-msaB	0.00	0.00
1132	msaA	0.32	0.00
1133	acyP	0.37	0.00
1134	xpaC	1.43	0.00
1135	terA	0.97	0.00
1136	brnQ3	1.12	0.00
1137	cobT	0.95	0.00
1138	norQ	1.39	0.00
1139	yozC	0.00	0.00
1140	Q5HG08	0.74	0.12
1141	odhB	2.04	0.08
1142	odhA	1.14	0.07
1143	arlS	1.03	0.00
1144	arlR	1.36	0.00
1145	pgpB	1.14	0.00
1146	murG	1.12	0.00
1147	Q7A0W4	0.78	0.00
1148	ctpA	2.01	0.00
1149	yozE	0.45	0.00
1150	crr	1.80	0.00
1151	msrB	2.56	0.00
1152	msrA2	0.94	0.00

Number/ Position	Gene	RGB-095930 compared to ERR1764920	RGB-095930 compared to NCTC8325
1153	degV2	0.60	0.00
1154	folA	1.46	0.00
1155	thyA-chr	1.67	0.00
1156	cvfC4	1.14	0.00
1157	cvfC3	3.20	0.00
1158	cvfC2	1.59	0.00
1159	cvfC1	0.00	0.00
1160	Q5HFZ1-yhhQ	1.28	0.00
1161	rnhA	10.70	0.00
1162	ebh	11.13	0.77
1163	norB	1.92	0.07
1164	steT	3.33	0.00
1165	ilvA1	1.44	0.00
1166	ald1	2.06	0.09
1167	exo53	1.02	0.11
1168	Q2FGZ8	1.19	0.03
1169	piuB	1.86	0.00
1170	Q5HFY0	1.20	0.00
1171	ypsC	0.79	0.09
1172	gpsB	0.58	0.00
1173	ypsA	0.00	0.00
1174	yppE	2.56	0.00
1175	recU	0.80	0.00
1176	pbpB	0.69	0.00
1177	Q1YBU7	1.17	0.00
1178	nth1	0.00	0.00
1179	dnaD	1.60	0.00
1180	asnS	1.31	0.00
1181	dinG1	0.82	0.07
1182	birA	1.03	0.10
1183	cca-papS	1.50	0.00
1184	bshA	1.14	0.00
1185	ypjD	0.31	0.00
1186	yugP	0.42	0.00
1187	ypjA	2.55	0.00
1188	ypiB	1.91	0.17
1189	ypiA	0.96	0.00
1190	aroA	3.54	0.00
1191	aroB	5.26	0.19
1192	aroC	6.60	0.00
1193	ndk	1.33	0.00
1194	gerCC	1.46	0.00
1195	ubiE	1.10	0.00
1196	gerCA	0.70	0.00
1197	hup	0.00	0.00
1198	gpdA	1.50	0.00
1199	engA	0.76	0.00
1200	rpsA	0.43	0.00
1201	Q2FYF8	0.00	0.00
1202	cmk	0.91	0.15
1203	ansA	1.14	0.00
1204	ypdA	0.71	0.00
1205	ebpS	3.22	0.07
1206	recQ2	0.94	0.00
1207	A5IT10	0.21	0.10
1208	fer	0.00	0.00
1209	ribU	1.28	0.00
1210	A5IT17-2	1.93	0.00
1211	A5IT17-1	35.31	0.13
1212	srrB	1.43	0.00
1213	srrA	0.14	0.00
1214	rluB	1.76	0.00
1215	scpB	2.95	0.00
1216	scpA	0.55	0.14
1217	Q2FY75	1.78	0.00
1218	xerD	0.79	0.00
1219	fur	0.22	0.00
1220	nudF	1.47	0.18
1221	yhdN	0.88	0.00
1222	Q5HFS1	0.80	0.00
1223	yqjQ	1.06	0.00
1224	proC	1.47	0.12
1225	rnz	1.19	0.00
1226	zwf	0.88	0.00
1227	graE	0.46	0.00
1228	malA-yugT	0.97	0.00
1229	malR-kdgR	2.25	0.00
1230	Q5HFR3	2.15	0.00
1231	gnd	0.57	0.07
1232	Q5HFR1-pepT2	0.79	0.09
1233	yqjA	0.61	0.00
1234	yqiW	0.00	0.00
1235	bfmBB	2.82	0.08
1236	bfmBAB	0.91	0.00
1237	bfmBAA	1.41	0.00
1238	lpdA	0.77	0.07
1239	recN	1.19	0.06
1240	ahrC	0.44	0.00
1241	ispA	1.81	0.00
1242	xseB	0.43	0.00
1243	xseA	0.67	0.07
1244	nusB	0.26	0.00
1245	yqhY	0.00	0.00
1246	accC	0.88	0.00
1247	accB	1.08	0.00
1248	efp	0.72	0.00
1249	yqhT	0.56	0.00
1250	Q5HFM8	0.17	0.00
1251	Q5HFM7	0.46	0.00
1252	lipM	1.20	0.12
1253	yqhL	0.26	0.00
1254	gcvPB	1.70	0.07
1255	gcvPA	0.82	0.00
1256	gcvT	0.37	0.18
1257	aroK	0.95	0.00
1258	comGF	2.21	0.00
1259	comGE	1.00	0.00
1260	comGD	1.12	0.00
1261	comGC	0.96	0.00
1262	comGB	1.40	0.09
1263	comGA	0.82	0.00
1264	yqgX	0.64	0.00
1265	yqgV	0.61	0.00
1266	glk	0.51	0.00
1267	yqgQ	0.49	0.00
1268	gluP	0.82	0.20
1269	yqgN	0.74	0.00
1270	rpmG1	0.00	0.00
1271	pbpC	1.64	0.05
1272	sodA-L2	0.33	0.00
1273	zur	0.97	0.00
1274	znuB	0.48	0.12
1275	znuC	0.64	0.00
1276	nfo	0.79	0.00
1277	cshB	1.04	0.00
1278	ybgI	1.82	0.00
1279	trmK	1.47	0.00
1280	sigA	0.54	0.00
1281	dnaG-var1800	1.33	0.00
1282	yqfL	1.22	0.00
1283	ccpN	0.96	0.00
1284	glyS	0.22	0.00
1285	recO	1.46	0.13
1286	era	0.78	0.11
1287	cdd	0.49	0.00
1288	dgkA	0.58	0.00
1289	ybeY	0.21	0.00
1290	phoH	0.33	0.00
1291	Q5HFI8	0.43	0.00
1292	floA	0.40	0.00
1293	yqeZ	6.51	0.13
1294	rpsU	0.00	0.00
1295	mtaB	0.97	0.00
1296	rsmE	1.06	0.00

Number/ Position	Gene	RGB-095930 compared to ERR1764920	RGB-095930 compared to NCTC8325
1297	prmA	1.17	0.00
1298	dnaJ	1.23	0.00
1299	dnaK	0.98	0.00
1300	grpE	0.64	0.00
1301	hrcA	0.92	0.00
1302	hemN	1.90	0.09
1303	lepA	1.21	0.00
1304	rpsT	0.00	0.00
1305	holA	0.62	0.00
1306	comEC	1.54	0.05
1307	comEB	0.69	0.00
1308	comEA	2.33	0.00
1309	yqeM-cypM	0.98	0.00
1310	rsfS	0.56	0.00
1311	yqeK	1.20	0.00
1312	nadD	1.05	0.00
1313	yhbY	1.37	0.00
1314	aroE	1.86	0.12
1315	yqeH	100.00	0.00
1316	yqeG	0.38	0.00
1317	Q5HFG1	2.22	0.00
1318	entX	3.32	0.00
1319	ycsG	1.54	0.00
1320	ycsF	0.80	0.00
1321	accC2	0.81	0.00
1322	accB2	1.78	0.00
1323	kipA	1.98	0.00
1324	kipI	1.63	0.14
1325	greA	0.63	0.00
1326	udk	0.64	0.00
1327	yrrO	0.63	0.00
1328	yrrN	0.87	0.00
1329	yrrM	0.47	0.00
1330	yrzB	0.32	0.00
1331	yrrK	0.23	0.00
1332	yrzL	0.38	0.00
1333	alaS	0.91	0.04
1334	recD	1.01	0.08
1335	yrrB	1.20	0.00
1336	mnmA	1.52	0.09
1337	csd1	0.61	0.00
1338	limB2	1.58	0.20
1339	Q5HFD8	100.00	0.00
1340	csbD-L2	1.64	1.09
1341	cymR	0.00	0.24
1342	rarA	1.80	0.00
1343	yrvM	0.90	0.26
1344	aspS	1.19	0.11
1345	hisS	0.71	0.00
1346	lytH	0.57	0.00
1347	dtd	1.10	0.00
1348	relA	0.91	0.00
1349	apt	0.19	0.00
1350	recJ	0.75	0.00
1351	secDF	0.75	0.04
1352	yajC	0.38	0.00
1353	tgt	0.35	0.09
1354	queA	0.97	0.00
1355	ruvB	1.09	0.00
1356	ruvA	1.00	0.00
1357	pheB	0.87	0.00
1358	obgE	1.01	0.00
1359	rpmA	0.00	0.00
1360	DUF464	0.31	0.00
1361	rplU	0.00	0.00
1362	mreD	1.51	0.00
1363	mreC	1.19	0.00
1364	Q5HFB3	0.63	0.00
1365	Q5HFB2	2.46	0.00
1366	radC	78.02	77.73
1367	comC-outO	2.12	0.14
1368	folC	1.02	0.00

Number/ Position	Gene	RGB-095930 compared to ERR1764920	RGB-095930 compared to NCTC8325
1369	valS	1.14	0.00
1370	tag	1.07	0.00
1371	abrB	1.49	0.00
1372	gsaA	1.55	0.00
1373	hemB	0.72	0.00
1374	hemD	0.75	0.00
1375	hemC	0.86	0.00
1376	hemX	1.59	0.25
1377	hemA1	0.30	0.15
1378	engB	1.18	0.00
1379	clpX	0.63	0.00
1380	tig	1.00	0.00
1381	ysoA	0.86	0.00
1382	ymaB	0.66	0.00
1383	rplT	0.84	0.00
1384	rpmI	0.50	0.00
1385	infC	0.38	0.19
1386	lysP1	6.49	0.00
1387	thrS	1.24	0.05
1388	dnaI	0.54	0.00
1389	dnaB	1.21	0.00
1390	nrdR	0.42	0.00
1391	gapB	1.17	0.00
1392	coaE	1.12	0.16
1393	mutM	1.72	0.00
1394	polA	1.03	0.04
1395	Q2YTE9	1.34	0.00
1396	phoR	1.44	0.00
1397	phoP	0.99	0.14
1398	citC	1.73	0.00
1399	citZ	1.43	0.09
1400	aapA1	10.50	0.00
1401	pykA	0.85	0.00
1402	pfkA	0.31	0.00
1403	accA	1.38	0.00
1404	accD	0.70	0.00
1405	maeB	1.30	0.08
1406	dnaE	3.10	0.03
1407	nrnA	2.02	0.00
1408	ytoI	1.15	0.00
1409	uspA	0.24	0.24
1410	ytkL	0.29	0.00
1411	pepQ	1.52	0.00
1412	ald2	1.34	0.00
1413	yxiE	0.60	0.00
1414	ackA	6.40	0.00
1415	ytxK	1.27	0.00
1416	tpx	0.81	0.00
1417	ytnM	2.85	0.00
1418	thiI	2.53	0.00
1419	csd2	8.42	0.09
1420	ezrA	0.53	0.12
1421	ytsP-msrC	1.51	0.22
1422	rpsD	0.00	0.00
1423	ugpQ2	1.75	0.00
1424	osmC	2.01	0.22
1425	pucG	1.46	0.17
1426	serA	1.37	0.06
1427	Q5HF49	1.06	0.00
1428	nagE	1.23	0.07
1429	plsC	0.81	0.00
1430	htrC	1.18	0.00
1431	tyrS	1.11	0.00
1432	sgtA	0.88	0.00
1433	isdH-harA	5.69	0.11
1434	fhs	1.68	0.12
1435	acsA1	1.29	0.00
1436	acuA	1.42	0.16
1437	acuC	1.62	0.09
1438	ccpA	2.02	0.00
1439	aroA2	1.10	0.09
1440	Q99TC6	89.53	10.47

Number/ Position	Gene	RGB-095930 compared to ERR1764920	RGB-095930 compared to NCTC8325
1441	yoxC	0.20	0.00
1442	murC	0.61	0.00
1443	Q5HF33	2.07	0.00
1444	pheT2	0.50	0.00
1445	Q5HF31	0.47	0.00
1446	ytpP	0.32	0.00
1447	pepA1	1.49	0.09
1448	ytzB	1.28	0.00
1449	ytnP	2.25	0.12
1450	trmB	1.24	0.16
1451	ytmP	0.76	0.00
1452	daaA	1.06	0.00
1453	ytjP	1.13	0.00
1454	Q5HF22	1.18	0.24
1455	rsuA	1.29	0.00
1456	ytgP	7.28	0.00
1457	Q1Y9Y5	2.44	0.00
1458	sasC	3.64	0.03
1459	ytwF-moeB	4.49	0.00
1460	leuS	2.03	0.00
1461	yttB	1.35	0.00
1462	ytqA	0.73	0.00
1463	ytqB	4.08	0.00
1464	rot	1.00	0.00
1465	pldB1	0.85	0.00
1466	putA	0.50	0.00
1467	ribH	2.58	0.00
1468	ribA	1.18	0.00
1469	ribB	2.21	0.00
1470	ribD	0.96	0.00
1471	Q5HF04	1.73	0.07
1472	arsR	1.90	0.00
1473	arsB	6.81	0.15
1474	arsC	100.00	0.00
1475	sagB-gad	0.82	0.00
1476	Q5HEZ9	100.00	0.00
1477	sigS	12.10	0.42
1478	Q5HEZ7-comK	2.93	0.00
1479	Q5HEZ6	13.96	0.00
1480	Q5HEZ5	20.28	0.00
1481	tal	0.70	0.00
1482	Q7A500	100.00	100.00
1483	crcB1	0.45	0.00
1484	crcB2	3.39	0.00
1485	ytbE-yvgN	100.00	100.00
1486	nrd	1.21	0.11
1487	metK	1.34	0.00
1488	pckA	1.63	0.00
1489	ytmA	5.71	0.00
1490	rppH	0.83	0.00
1491	ytjA-yidD	1.55	0.00
1492	menC	2.89	0.00
1493	menE	2.64	0.00
1494	Q5HEY1	3.31	0.00
1495	Q5HEY0	3.99	0.00
1496	DUF4352	1.71	0.00
1497	Q5HEX8	5.74	0.00
1498	Q5HEX7	100.00	0.00
1499	A5ITW8	100.00	0.00
1500	hsdS-spl	100.00	0.17
1501	hsdM-spl	4.24	2.63
1502	splF	100.00	0.00
1503	splE	8.89	0.00
1504	splD1	33.06	0.00
1505	splC	8.19	0.14
1506	splB	11.34	0.00
1507	splA	7.20	0.00
1508	Q2FXC0-ear2	100.00	0.00
1509	epiG	63.57	0.00
1510	epiE	100.00	0.00
1511	epiF	100.00	0.14
1512	epiP	100.00	0.00
1513	epiD	100.00	0.00
1514	epiC	100.00	0.00
1515	epiB	100.00	0.00
1516	epiA2	100.00	0.69
1517	bsaX	100.00	0.00
1518	epiA1	100.00	0.00
1519	lukD	10.16	0.20
1520	lukE	2.03	0.11
1521	Q7A4X2	11.32	0.47
1522	ydeN	0.90	0.18
1523	hemY	4.14	0.07
1524	hemH	0.76	0.22
1525	hemE	1.54	0.10
1526	traP	13.10	0.00
1527	ecsB	2.04	0.00
1528	ecsA	1.21	0.00
1529	hit	0.95	0.00
1530	gvpP	1.91	0.00
1531	yhaJ	0.00	0.00
1532	prsA2	0.62	0.00
1533	cbf	0.53	0.00
1534	Q5HET2	0.72	0.03
1535	Q5HET1	0.84	0.00
1536	UPF0342	0.00	0.00
1537	UPF0754	1.69	0.09
1538	xdrA	0.00	0.22
1539	airR	0.48	0.00
1540	airS	1.17	0.00
1541	rluA2	1.46	0.00
1542	fumC-citG	100.00	0.00
1543	Q5HES3	1.77	0.00
1544	Q5HES1	1.31	0.00
1545	Q5HES0	1.00	0.00
1546	cspR-trmL	0.42	0.00
1547	queG	1.24	0.09
1548	artR	0.55	0.00
1549	artQ	0.96	0.00
1550	Q1Y9N4	0.85	0.00
1551	perR	0.22	0.00
1552	Q5HER2	1.89	0.00
1553	bcp	0.88	0.22
1554	gsaB	2.09	0.00
1555	DUF939_L1	0.91	0.00
1556	msbA2	1.21	0.06
1557	DUF402	0.18	0.00
1558	mutY	1.54	0.00
1559	DUF457	0.61	0.00
1560	tagG	1.79	0.00
1561	tagH	1.19	0.00
1562	yfhH	0.63	0.00
1563	recX	0.37	0.00
1564	sgtB	1.11	0.00
1565	pfpI	1.36	0.19
1566	Q5HEP8	0.62	0.62
1567	A5IU43-yfkAB	2.21	1.36
1568	A5IU44	1.13	0.00
1569	ampS	1.20	0.00
1570	Q5HEP4	0.48	0.00
1571	ptpA	0.65	0.00
1572	Q5HEP2	0.00	0.00
1573	rbn	1.15	0.00
1574	vraR	0.63	0.00
1575	vraS	0.86	0.10
1576	vraT	0.43	0.00
1577	vraU	0.52	0.00
1578	ampM	1.00	0.00
1579	DUF939_L2	2.03	0.00
1580	Q5HEN4	3.62	0.00
1581	gatD	1.37	0.00
1582	murT	0.68	0.08
1583	ftnA	0.80	0.00
1584	dinG2	0.90	0.00

Number/ Position	Gene	RGB-095930 compared to ERR1764920	RGB-095930 compared to NCTC8325
1585	dinB	1.03	0.09
1586	Q5HEM6	0.19	0.00
1587	rlmCD	0.66	0.00
1588	dagK	0.53	0.11
1589	gatB	0.84	0.00
1590	gatA	0.96	0.00
1591	gatC	0.33	0.00
1592	putP	0.45	0.13
1593	camS	0.58	0.00
1594	ligA	1.80	0.00
1595	pcrA	0.87	0.00
1596	pcrB	0.87	0.00
1597	yerC	0.00	0.00
1598	purB	1.16	0.00
1599	sspP	1.80	0.00
1600	sspS	1.83	0.00
1601	Q5HEL1	2.30	0.00
1602	UPF0316	0.83	0.00
1603	nadE	4.14	0.12
1604	nadC	0.82	0.00
1605	nos	0.37	0.00
1606	pdt	1.26	0.13
1607	sdcS	1.66	0.00
1608	Q5HEK3-pfbA	1.82	0.00
1609	pncA	0.53	0.00
1610	ppaC	0.43	0.00
1611	aldH	0.94	0.07
1612	Q5HEJ8	0.78	0.00
1613	Q5HEJ7	0.00	0.00
1614	A6U329	0.57	0.00
1615	A6U330	1.17	0.00
1616	Q5HEJ4	1.54	0.00
1617	DUF1700	0.72	0.18
1618	Q5HEJ2	0.57	0.00
1619	Q5HEJ1	1.06	0.18
1620	pmtD	1.35	0.00
1621	pmtC	0.57	0.00
1622	pmtB	0.73	0.00
1623	pmtA	3.01	0.11
1624	pmtR	2.62	0.26
1625	A6U340	6.78	0.00
1626	Q5HEI3	2.33	0.08
1627	map	15.53	0.10
1628	hlb	5.36	0.30
1629	lukX	12.45	0.00
1630	lukY	14.77	0.09
1631	dapE	5.39	0.16
1632	ktrB	1.53	0.00
1633	groL	2.72	0.06
1634	groS	3.16	0.00
1635	ydiL	8.20	0.00
1636	sdrH	3.65	6.35
1637	Q5HEG8	0.96	0.00
1638	mtnU-ramA	100.00	0.00
1639	hld	0.00	0.00
1640	agrB-Ia	9.47	0.00
1641	agrD-Iac	11.35	0.00
1642	agrC-Iabd	8.97	0.00
1643	agrA	1.81	0.00
1644	scrK-frk	1.35	0.00
1645	scrB	1.55	0.00
1646	scrR	1.05	0.00
1647	nrgA	5.36	0.00
1648	Q5HEF7-tusA	0.44	0.00
1649	yeeE-DUF395	1.86	0.00
1650	rex	0.31	0.00
1651	yheS	1.04	0.10
1652	mutS3	2.23	0.00
1653	tsaD	1.27	0.00
1654	rimI	1.08	0.00
1655	tsaB	1.06	0.00
1656	tsaE	1.15	0.00

Number/ Position	Gene	RGB-095930 compared to ERR1764920	RGB-095930 compared to NCTC8325
1657	ilvD	1.54	0.06
1658	ilvB	100.00	0.06
1659	ilvH	0.78	0.00
1660	ilvC	0.50	0.00
1661	leuA1	0.46	0.00
1662	leuB	0.96	0.10
1663	leuC	1.39	0.00
1664	leuD	4.89	0.00
1665	ilvA2	3.55	0.00
1666	sprL	2.63	0.00
1667	yhgF	0.88	0.00
1668	sigB	0.91	0.00
1669	rsbW	0.83	0.21
1670	rsbV	0.00	0.00
1671	rsbU	0.20	1.10
1672	mazF	0.55	0.00
1673	mazE	0.00	0.00
1674	alr1	1.04	0.00
1675	acpS	2.78	0.00
1676	A5IUL8	2.85	0.00
1677	DUF304	2.78	0.06
1678	Q9ZAH9	4.58	0.00
1679	kdpC-chr	2.32	0.00
1680	kdpB-chr	0.99	0.00
1681	kdpA-chr	1.37	0.00
1682	kdpF-chr	0.90	0.00
1683	kdpD-chr	1.39	0.04
1684	kdpE-chr	0.86	0.00
1685	cshA	1.05	0.00
1686	murF	1.32	0.07
1687	ddlA	0.47	0.00
1688	ftsW1-rodA	0.67	0.00
1689	Q5HEB5	0.00	0.00
1690	csoZ	1.43	0.00
1691	csoR	0.34	0.00
1692	cls2	1.14	0.00
1693	yedJ	1.70	0.00
1694	yidC	0.57	0.00
1695	thiE	2.49	0.16
1696	thiM	1.39	0.13
1697	thiD2	1.20	0.00
1698	tenA	2.46	0.29
1699	sceD	1.15	0.00
1700	Q5HEA3	0.25	0.00
1701	ywpF	0.45	0.00
1702	fabZ	0.68	0.00
1703	murA	2.92	0.24
1704	ywzB	0.00	0.00
1705	atpE	0.00	0.00
1706	atpD	0.28	0.00
1707	atpG	0.12	0.00
1708	atpA	0.20	0.00
1709	atpH	0.56	0.00
1710	atpF	0.00	0.00
1711	atpL	0.00	0.00
1712	atpB	0.14	0.00
1713	atpI	4.80	0.00
1714	mnaA	2.03	0.09
1715	upp	0.48	0.00
1716	glyA	0.48	0.16
1717	ywlG	0.95	0.00
1718	ptpB	1.19	0.00
1719	tsaC	100.00	0.00
1720	prmC	0.60	0.00
1721	prfA	0.65	0.00
1722	tdk	0.50	0.00
1723	rpmE	0.00	0.00
1724	rho	4.86	0.08
1725	aldA3	0.98	0.00
1726	arxR	0.00	0.89
1727	murZ	0.00	1.11
1728	fbaA	0.00	1.74

Number/ Position	Gene	RGB-095930 compared to ERR1764920	RGB-095930 compared to NCTC8325
1729	DUF2529	0.00	0.38
1730	pyrG	0.06	1.55
1731	rpoE	0.00	0.56
1732	Q5HE71	0.12	1.16
1733	coaW	0.00	0.75
1734	DUF2750	0.00	0.15
1735	A5IUS7	0.00	1.34
1736	hmrA	0.00	1.43
1737	luxS	0.00	1.06
1738	Q5HE65	0.00	0.28
1739	pdp	0.00	1.38
1740	deoC-L2	0.00	2.41
1741	deoD-L2	0.00	0.56
1742	dpsA	0.00	2.03
1743	DUF393	0.00	1.45
1744	Q5HE59	0.00	0.87
1745	Q7A0B3	0.00	2.16
1746	manA1	0.00	0.74
1747	yhfK1	0.00	1.80
1748	czrA	0.00	0.31
1749	czrB	0.10	1.94
1750	Q9ZB00	0.00	1.19
1751	ylmA	0.00	7.02
1752	glmS	0.00	1.16
1753	mtlA	0.00	3.64
1754	mtlR	0.00	1.17
1755	mtlF	0.23	0.46
1756	mtlD	0.00	1.45
1757	sasB	0.00	3.81
1758	glmM	0.00	0.74
1759	ybbR-cdaR	0.00	0.32
1760	cdaA-dacA	0.12	0.49
1761	argI-rocF	0.00	6.82
1762	salA	0.00	3.75
1763	ycnB	0.00	100.00
1764	sepA	0.00	10.26
1765	sdrM	0.00	17.93
1766	hlIII	0.00	4.97
1767	Q5HE34-urtF	0.00	3.20
1768	yvsG-ydjM	0.00	0.58
1769	ynzG	0.00	1.53
1770	Q5HE31	0.00	4.01
1771	htsC-fecD	0.00	0.93
1772	htsB	100.00	9.40
1773	htsA	0.00	2.95
1774	Q5HE27-sfnaC	0.09	1.40
1775	rhbC1	0.00	1.48
1776	Q5HE25	0.00	0.50
1777	rhbC2	0.00	1.21
1778	asp23	0.00	1.18
1779	DUF2273	0.00	0.42
1780	Q5HE21	0.00	2.55
1781	opuD2	0.00	3.13
1782	Q5HE19	0.00	3.17
1783	qorA	0.00	1.60
1784	DUF915	0.00	1.95
1785	lacG	0.00	1.56
1786	lacE	0.00	1.98
1787	lacF	0.00	1.92
1788	lacD	100.00	1.43
1789	lacC	100.00	1.61
1790	lacB	0.00	1.16
1791	lacA	0.00	0.00
1792	lacR	0.00	2.38
1793	cobB	0.00	1.50
1794	Q5HE05	0.00	50.00
1795	yvgN2	0.12	0.71
1796	adhR	0.00	0.96
1797	eap-L2-eapH-1	0.00	6.57
1798	alsD-L1	0.00	3.40
1799	alsS	0.00	3.60
1800	rpsI	0.00	0.25

Number/ Position	Gene	RGB-095930 compared to ERR1764920	RGB-095930 compared to NCTC8325
1801	rplM	0.00	0.00
1802	truA	0.00	2.61
1803	ecfT	0.00	1.24
1804	ecfA1	0.00	1.39
1805	ecfA2	0.00	1.23
1806	rplQ	0.00	0.00
1807	rpoA	0.00	0.21
1808	rpsK	0.00	0.00
1809	rpsM	0.00	0.00
1810	rpmJ	0.00	0.00
1811	infA	0.00	0.00
1812	adk	0.00	0.00
1813	secY1	0.00	0.15
1814	rplO	0.00	0.00
1815	rpmD	0.00	0.00
1816	rpsE	0.00	0.00
1817	rplR	0.00	0.00
1818	rplF	0.00	0.00
1819	rpsH	0.00	0.50
1820	rpsZ	0.00	0.00
1821	rplE	0.00	0.00
1822	rplX	0.00	0.31
1823	rplN	0.00	0.27
1824	rpsQ	0.00	0.00
1825	rpmC	0.00	0.00
1826	rplP	0.23	0.00
1827	rpsC	0.00	0.15
1828	rplV	0.00	0.00
1829	rpsS	0.00	0.00
1830	rplB	0.00	0.00
1831	rplW	0.00	0.36
1832	rplD	0.00	0.16
1833	rplC	0.00	0.30
1834	rpsJ	0.00	0.65
1835	Q5HDV6	0.00	0.26
1836	pbuG	0.00	1.05
1837	topB	0.00	0.98
1838	Q5HDV3	0.00	0.68
1839	glcU	0.00	0.46
1840	Q1Y7Y7	0.00	1.10
1841	Q5HDU8	0.31	0.94
1842	acrB	0.00	1.07
1843	fmhB	0.00	1.11
1844	Q5HDU5	0.00	1.05
1845	Q5HDU4	0.00	0.45
1846	ybfD	0.00	0.99
1847	sarV	0.00	0.28
1848	moaA	0.00	1.08
1849	mobA	0.00	0.83
1850	moaD	0.00	1.28
1851	moaE	0.00	0.89
1852	mobB	0.00	1.23
1853	moeA	0.08	1.51
1854	moaC	0.00	1.41
1855	moaB	0.00	2.96
1856	moeB	0.00	1.89
1857	modC	0.00	1.49
1858	modB	0.00	0.30
1859	modA	0.13	5.75
1860	fdhD	0.00	2.38
1861	Q5HDS6	0.00	7.26
1862	bioY	0.00	1.26
1863	rihB	0.00	1.06
1864	fhuD2	0.00	0.77
1865	caiA	0.00	2.49
1866	utp	0.00	4.97
1867	ureA	0.00	0.66
1868	ureB	0.00	0.73
1869	ureC	0.00	1.11
1870	ureE	0.00	1.77
1871	ureF	0.00	0.72
1872	ureG	0.16	0.49

Number/ Position	Gene	RGB-095930 compared to ERR1764920	RGB-095930 compared to NCTC8325
1873	ureD	0.00	0.96
1874	sarR	0.00	0.00
1875	rsr	0.00	1.83
1876	iraD	0.00	0.94
1877	iraC	0.00	1.07
1878	ssaA2	0.00	1.36
1879	nhaC	0.00	2.50
1880	Q5HDQ7	0.00	2.03
1881	Q2FEJ1	0.00	1.65
1882	ssaA3	0.00	2.79
1883	yvcT	0.00	1.47
1884	Q8NV79	0.09	1.07
1885	sagA-lytA	0.00	0.77
1886	Q5HDQ1	0.00	0.00
1887	yrhD	0.00	7.59
1888	fdhL	0.00	6.23
1889	lytR2-lcpC	0.00	0.31
1890	suhB2	0.00	0.88
1891	Q5HDP6	0.14	1.01
1892	Q5HDP4	0.14	1.36
1893	Q5HDP3	0.00	0.30
1894	rpiRC	0.00	0.57
1895	proY	0.00	1.16
1896	Q5HDP0	0.00	2.80
1897	Q5HDN9	0.00	0.00
1898	Q5HDN8	0.00	0.00
1899	glpW	0.00	1.10
1900	yocS	0.00	0.54
1901	Q5HDN5	0.00	0.38
1902	glvC	0.06	3.24
1903	glvR	0.00	2.48
1904	Q5HDN2	0.00	0.79
1905	nhaC2	0.00	100.00
1906	Q5HDN0	0.00	9.77
1907	yhxD-yghA	0.00	1.36
1908	Q5HDM8-yxeP	0.36	2.58
1909	hutI	0.00	1.13
1910	hutU	0.00	0.72
1911	hutR	0.00	1.24
1912	fosB	0.00	100.00
1913	hutG	0.00	2.56
1914	lyrA	0.00	4.68
1915	rpiA	0.00	0.73
1916	yflK-yiiM	0.00	1.53
1917	galM	0.00	0.69
1918	ynfA	0.31	0.61
1919	yhaP	0.00	1.22
1920	yhaQ	0.00	0.78
1921	Q5HDL3-yhaI	0.00	20.34
1922	yxlJ	0.00	1.15
1923	gltS	0.08	2.23
1924	fni-idi2	0.00	0.76
1925	corA	0.00	1.05
1926	Q5HDK8	0.00	1.76
1927	Q5HDK7	0.00	9.05
1928	mlhB	0.00	1.22
1929	Q5HDK5	0.16	1.56
1930	semB	0.05	6.11
1931	semA	0.00	6.33
1932	Q5HDK2	0.18	1.80
1933	tcaB	0.08	0.74
1934	tcaA	0.00	0.94
1935	tcaR	0.00	0.22
1936	Q5HDJ7	0.00	1.14
1937	hrtA	0.15	1.05
1938	hrtB	0.09	1.23
1939	hssR	0.00	1.04
1940	hssS	0.00	1.75
1941	lytT	0.00	0.90
1942	Q5HDJ1	0.00	3.73
1943	mqo	0.00	1.42
1944	lctP-locus2	0.00	1.31

Number/ Position	Gene	RGB-095930 compared to ERR1764920	RGB-095930 compared to NCTC8325
1945	tagF	0.00	0.90
1946	Q5HDI7	0.00	7.14
1947	paiA	0.00	1.74
1948	yhfP-A1KWX9	0.00	1.00
1949	Q5HDI4	0.00	5.22
1950	iruO	0.00	1.16
1951	Q5HDI2	0.00	0.00
1952	pip	0.00	1.10
1953	Q5HDH9-tetR21	0.00	1.12
1954	cobI	0.00	0.21
1955	scrA	0.07	1.32
1956	DUF1722	0.00	2.34
1957	rsp-araC	60.02	60.49
1958	ydaG	0.00	2.36
1959	Q5HDH2	0.00	6.72
1960	gltT	0.00	1.96
1961	DUF3139	0.00	1.09
1962	sarZ	0.22	2.91
1963	hsp20	0.00	5.36
1964	narK	0.00	4.79
1965	nreC	0.00	3.52
1966	nreB	0.10	7.92
1967	nreA	0.00	5.52
1968	narI	0.00	7.96
1969	narJ	0.00	4.57
1970	narH	0.00	7.76
1971	narG	0.00	4.72
1972	nasF	0.00	11.66
1973	nasE	0.00	10.16
1974	nasD	0.00	3.95
1975	nirR-nasR	0.14	12.55
1976	Q5HDF4	0.00	7.08
1977	focA-L2-nirC	0.00	13.70
1978	Q8NV18	0.00	15.30
1979	Q5HDF2	0.00	2.14
1980	zinT	0.00	0.84
1981	yoeB1	0.00	0.75
1982	yefM1	0.00	1.19
1983	dsbA	0.00	1.00
1984	DUF4467	0.00	0.28
1985	fmhA	0.00	4.80
1986	tcyC	0.00	7.79
1987	tcyB	0.00	7.13
1988	tcyA	0.00	2.82
1989	mdeA	0.00	1.94
1990	gpmA2	0.00	0.73
1991	cdf	0.00	0.81
1992	sbi	0.00	5.63
1993	hlgA	0.00	1.08
1994	lukS	0.00	5.27
1995	lukF	0.00	5.73
1996	bioX	0.21	2.35
1997	bioW	0.00	0.87
1998	bioF	0.00	1.97
1999	bioB	0.00	2.18
2000	bioA	0.00	0.66
2001	bioD	0.00	1.75
2002	Q5HDC6-L1	0.00	0.69
2003	msbA3	0.00	0.17
2004	msbA4	0.00	1.42
2005	Q5HDC6-L2	0.00	1.85
2006	ldr_fst-L2	0.00	0.93
2007	gtrA	0.00	2.07
2008	glxK1	0.00	100.00
2009	Q5HDC0	0.00	1.36
2010	bcr	0.00	2.48
2011	aarP	0.00	0.42
2012	Q5HDB7	0.00	0.46
2013	Q5HDB6	0.00	1.73
2014	aapA3	0.07	0.50
2015	nhaK2	0.00	0.63
2016	ydaO	0.00	1.26

Number/ Position	Gene	RGB-095930 compared to ERR1764920	RGB-095930 compared to NCTC8325
2017	flp-fmtA	0.00	4.53
2018	rfbD	0.00	0.94
2019	panE1	0.00	0.85
2020	mmr	0.00	0.93
2021	opuCD	0.00	1.44
2022	opuCC	0.00	2.12
2023	opuCB	0.16	2.52
2024	opuCA	0.00	1.87
2025	ydeI-opuCB	0.17	2.67
2026	ybeC	0.06	1.94
2027	pnbA	0.00	100.00
2028	pbuE	0.00	2.02
2029	yjkA-ybbM	0.13	7.08
2030	yjkB	0.15	13.73
2031	pepA2	0.00	2.69
2032	Q5HD92	0.00	0.66
2033	yerD	0.00	1.90
2034	Q5HD90	0.00	2.93
2035	norD	0.00	1.01
2036	cntF	0.00	0.93
2037	cntD	0.00	3.80
2038	cntC	0.00	1.03
2039	cntB	0.00	0.75
2040	cntA	0.00	0.94
2041	DUF2338-cntM	0.00	2.46
2042	fmrO	0.00	0.49
2043	dapF	0.00	1.46
2044	yxbG	0.00	10.99
2045	Q5HD78	0.00	1.75
2046	aphD	0.00	0.24
2047	bdhA	0.00	1.15
2048	iraB	0.00	0.35
2049	Q5HD71	0.52	0.52
2050	pgcA	0.00	4.21
2051	Q5HD60	0.00	0.37
2052	Q5HD59	0.00	6.15
2053	sasG	0.00	100.00
2054	sarT	0.00	100.00
2055	sarU	0.00	100.00
2056	gtaB	0.00	1.38
2057	fnbB	55.65	13.32
2058	Q5HD52	0.00	3.25
2059	fnbA	99.77	13.28
2060	Q2G207	0.00	1.00
2061	gntP	0.00	1.62
2062	gntK	0.06	3.02
2063	gntR	0.00	0.88
2064	merR3	0.00	2.88
2065	relP	0.00	1.01
2066	DUF2188	0.20	0.82
2067	yccS	0.00	5.68
2068	ycbE	0.00	0.86
2069	dedA	0.00	2.29
2070	stp	0.14	1.44
2071	smp	0.00	1.04
2072	fbp	0.00	10.08
2073	Q1Y5K2	0.00	1.67
2074	Q1Y5K1	0.00	3.20
2075	mhqA-2	0.00	1.86
2076	mhqR	0.00	0.69
2077	Q5HD32	0.00	3.86
2078	catE	0.00	1.24
2079	frp	0.00	1.19
2080	ldhD	0.00	0.70
2081	ywtE	0.00	2.37
2082	srtA	0.00	1.29
2083	ywnH	0.00	9.35
2084	sdaA	0.11	1.11
2085	sdaB	0.00	0.59
2086	ptsEIIC	0.00	0.67
2087	Q5HD18	0.00	11.79
2088	ydeD	0.00	0.76

Number/ Position	Gene	RGB-095930 compared to ERR1764920	RGB-095930 compared to NCTC8325
2089	aes	0.00	1.34
2090	Q5HD15	0.00	1.45
2091	Q5HD14	0.00	1.22
2092	glcB	0.00	100.00
2093	cidC-pox	0.00	2.36
2094	cidB	0.00	3.62
2095	cidA	0.00	1.01
2096	cidR	0.00	1.25
2097	Q2FV82	0.00	0.00
2098	ssaA4	0.00	1.85
2099	mvaA	0.00	5.07
2100	mvaS	0.00	1.20
2101	adaB	0.00	3.26
2102	clpL	2.90	6.17
2103	A8Z3E9	0.00	6.43
2104	feoB	0.00	1.65
2105	feoA	0.00	0.00
2106	mmpL-farE	0.00	7.15
2107	Q8NUR3	0.00	6.74
2108	Q5HCZ7	0.00	1.19
2109	rocA-pruA	0.00	1.23
2110	maa	0.00	5.50
2111	cwrA	0.00	3.13
2112	copA	0.00	0.91
2113	copZ	0.00	0.48
2114	ddh	0.00	0.80
2115	ywfG	0.00	1.90
2116	crtN	0.00	1.06
2117	crtM	0.00	1.62
2118	crtQ	0.00	2.39
2119	crtP	0.00	1.07
2120	crtO	0.20	3.01
2121	ssaA1	0.00	1.56
2122	oatA	0.00	1.43
2123	isaA	0.00	0.14
2124	Q5HCY0	0.00	1.04
2125	acrA	0.00	2.63
2126	Q5HCX8	0.00	4.19
2127	ynzC	0.00	0.00
2128	Q5HCX6	0.00	1.59
2129	nmrA	0.00	1.09
2130	Q5HCX4	0.00	5.00
2131	gbaA	0.00	4.12
2132	gbaB	0.14	5.25
2133	Q5HCX0	0.10	1.19
2134	yraK	0.00	1.32
2135	cobW3	0.00	1.35
2136	Q6GDL8	0.00	1.11
2137	Q5HCW6	0.00	1.54
2138	DUF4176	0.00	1.35
2139	Q8NUP1	0.00	2.17
2140	Q8NUP0	0.00	4.35
2141	Q8NUN9	0.00	3.34
2142	pyrD	0.00	1.88
2143	Q5HCW0	0.00	3.99
2144	DUF208	0.00	1.11
2145	Q5HCV8	0.00	0.89
2146	pepX	0.06	2.08
2147	panD	0.00	1.04
2148	panC	0.00	0.82
2149	panB	0.00	0.73
2150	panE2	0.00	0.81
2151	alsD-L2	0.00	0.85
2152	ldh2	0.00	1.35
2153	yfnA1	0.07	1.24
2154	yhxA-gabT	0.07	1.05
2155	Q5HCU7	0.00	1.68
2156	fda	0.00	3.14
2157	lqo	0.00	0.67
2158	acsA2-bclA	0.00	1.19
2159	Q5HCU3	0.00	2.25
2160	Q5HCU2	0.00	0.00

Number/ Position	Gene	RGB-095930 compared to ERR1764920	RGB-095930 compared to NCTC8325
2161	betA	0.00	1.11
2162	betB	0.00	0.94
2163	A8Z5A6	0.00	1.04
2164	Q79ZX2	0.00	0.71
2165	cudT	0.00	0.68
2166	nrdG	0.00	1.12
2167	nrdD	0.00	1.03
2168	citM	0.00	1.90
2169	cysG	0.00	4.93
2170	cysJ	0.00	0.80
2171	gpxA-L2	0.00	1.00
2172	A6QKB0	0.00	2.78
2173	nsaB	0.00	2.60
2174	nsaA	0.00	1.18
2175	nsaS	0.00	0.79
2176	nsaR	0.00	1.05
2177	Q5HCS2	0.00	1.00
2178	phoB	0.00	1.54
2179	DUF2648	0.00	0.00
2180	slyA	0.00	0.44
2181	estA	0.00	1.31
2182	clfB	4.99	9.15
2183	arcR	0.14	0.85
2184	arcC-L2	0.00	0.53
2185	arcD-L2	0.07	1.47
2186	arcB-L2	0.00	0.49
2187	arcA-L2	0.00	1.13
2188	argR-L2	0.00	6.89
2189	aur	0.13	10.85
2190	isaB	0.00	16.29
2191	Q6G635	0.00	16.45
2192	manR	0.00	2.88
2193	manP	0.00	4.61
2194	manA2-yvyI	0.00	0.75
2195	yhgE	0.00	2.52
2196	scaH-lytZ	0.00	1.34
2197	ywoC1	0.00	0.36
2198	sasF	0.00	2.52
2199	gtfB	0.00	1.40
2200	gtfA	0.00	1.86
2201	secA2	0.00	1.46
2202	asp3	0.00	1.88
2203	asp2	0.00	1.21
2204	asp1	0.00	1.35
2205	secY2	0.00	2.15
2206	sasA	0.01	7.79
2207	A6U540	0.00	9.24
2208	Q5HCP0	0.00	30.69
2209	Q5HCN9	0.00	2.78
2210	ywrF	0.00	2.29
2211	msrA3	0.00	2.31
2212	Q5HCN6	0.00	2.20
2213	capC-L2	0.00	1.56
2214	capB-L2	0.00	2.74
2215	capA-L2	0.00	2.26
2216	icaR	0.18	1.07
2217	icaA	0.00	0.81
2218	icaD	0.00	0.65
2219	icaB	0.00	0.57
2220	icaC	0.00	3.80
2221	lip1	0.00	2.88
2222	hisI	0.00	1.74
2223	hisF	0.00	2.24
2224	hisA	0.00	2.70
2225	hisH	0.00	3.11
2226	hisB	0.00	3.28
2227	hisC1	0.10	3.35
2228	hisD	0.08	4.56
2229	hisG	0.00	0.81
2230	hisZ	0.00	1.10
2231	Q5HCL6	0.00	3.91
2232	Q5HCL5	0.00	78.71

Number/ Position	Gene	RGB-095930 compared to ERR1764920	RGB-095930 compared to NCTC8325
2233	yceI	0.00	4.65
2234	drp35	0.00	4.63
2235	Q5HCK8	0.00	2.93
2236	pcp	0.00	4.07
2237	Q5HCK6	0.00	100.00
2238	padR	0.00	100.00
2239	Q2FUS3	0.00	3.23
2240	cna	49.47	100.00
2241	Q7WS08	0.07	1.90
2242	rarD	0.11	2.75
2243	Q5HCK1	0.17	5.56
2244	nixA	0.00	3.05
2245	Q5HCJ9-nhoA	0.00	13.36
2246	hdeD	0.00	7.02
2247	vraD	0.00	1.32
2248	vraE	0.00	4.68
2249	Q5HCJ5	0.00	7.29
2250	cspC-L3-cspB	0.00	0.50
2251	Q5HCI9-immR	0.00	2.81
2252	DUF3147_L1	0.00	0.52
2253	DUF3147_L2	0.00	1.41
2254	noc	0.00	0.83
2255	rsmG-gidB	0.00	0.56
2256	mnmG-gidA	0.00	0.64
2257	mnmE-trmE	0.00	0.58
2258	rnpA	0.00	0.00
2259	rpmH	0.00	0.00
	MEAN	3.93	3.08
	MEDIAN	0.70	0.00
